# Global, regional, and national burden of traumatic brain injury and spinal cord injury, 1990–2016: a systematic analysis for the Global Burden of Disease Study 2016

**DOI:** 10.1016/S1474-4422(18)30415-0

**Published:** 2019-01

**Authors:** GBD 2016 Traumatic Brain Injury and Spinal Cord Injury Collaborators, Spencer L James, Alice Theadom, Richard G Ellenbogen, Marlena S Bannick, Wcliff Montjoy-Venning, Lydia R Lucchesi, Nooshin Abbasi, Rizwan Abdulkader, Haftom Niguse Abraha, Jose C Adsuar, Mohsen Afarideh, Sutapa Agrawal, Alireza Ahmadi, Muktar Beshir Ahmed, Amani Nidhal Aichour, Ibtihel Aichour, Miloud Taki Eddine Aichour, Rufus Olusola Akinyemi, Nadia Akseer, Fares Alahdab, Animut Alebel, Suliman A Alghnam, Beriwan Abdulqadir Ali, Ubai Alsharif, Khalid Altirkawi, Catalina Liliana Andrei, Mina Anjomshoa, Hossein Ansari, Mustafa Geleto Ansha, Carl Abelardo T Antonio, Seth Christopher Yaw Appiah, Filippo Ariani, Nigus Gebremedhin Asefa, Solomon Weldegebreal Asgedom, Suleman Atique, Ashish Awasthi, Beatriz Paulina Ayala Quintanilla, Tambe B Ayuk, Peter S Azzopardi, Hamid Badali, Alaa Badawi, Shivanthi Balalla, Amrit Banstola, Suzanne Lyn Barker-Collo, Till Winfried Bärnighausen, Neeraj Bedi, Masoud Behzadifar, Meysam Behzadifar, Bayu Begashaw Bekele, Abate Bekele Belachew, Yihalem Abebe Belay, Derrick A Bennett, Isabela M Bensenor, Adugnaw Berhane, Mircea Beuran, Ashish Bhalla, Soumyadeeep Bhaumik, Zulfiqar A Bhutta, Belete Biadgo, Marco Biffino, Ali Bijani, Nigus Bililign, Charles Birungi, Soufiane Boufous, Alexandra Brazinova, Allen W Brown, Mate Car, Rosario Cárdenas, Juan J Carrero, Félix Carvalho, Carlos A Castañeda-Orjuela, Ferrán Catalá-López, Yazan Chaiah, Ana Paula Champs, Jung-Chen Chang, Jee-Young J Choi, Devasahayam J Christopher, Cyrus Cooper, Christopher Stephen Crowe, Lalit Dandona, Rakhi Dandona, Ahmad Daryani, Dragos Virgil Davitoiu, Meaza Girma Degefa, Gebre Teklemariam Demoz, Kebede Deribe, Shirin Djalalinia, Huyen Phuc Do, David Teye Doku, Thomas M Drake, Manisha Dubey, Eleonora Dubljanin, Ziad El-Khatib, Richard Ofori-Asenso, Sharareh Eskandarieh, Alireza Esteghamati, Sadaf Esteghamati, Andre Faro, Farshad Farzadfar, Mohammad Hosein Farzaei, Seyed-Mohammad Fereshtehnejad, Eduarda Fernandes, Garumma Tolu Feyissa, Irina Filip, Florian Fischer, Takeshi Fukumoto, Morasaleh Ganji, Fortune Gbetoho Gankpe, Abadi Kahsu Gebre, Tsegaye Tewelde Gebrehiwot, Kebede Embaye Gezae, Gururaj Gopalkrishna, Alessandra C Goulart, Juanita A Haagsma, Arvin Haj-Mirzaian, Arya Haj-Mirzaian, Randah R Hamadeh, Samer Hamidi, Josep Maria Haro, Hadi Hassankhani, Hamid Yimam Hassen, Rasmus Havmoeller, Caitlin Hawley, Simon I Hay, Mohamed I Hegazy, Delia Hendrie, Andualem Henok, Desalegn Tsegaw Hibstu, Howard J Hoffman, Michael K Hole, Enayatollah Homaie Rad, Seyed Mostafa Hosseini, Sorin Hostiuc, Guoqing Hu, Mamusha Aman Hussen, Olayinka Stephen Ilesanmi, Seyed Sina Naghibi Irvani, Mihajlo Jakovljevic, Sudha Jayaraman, Ravi Prakash Jha, Jost B Jonas, Kelly M Jones, Zahra Jorjoran Shushtari, Jacek Jerzy Jozwiak, Mikk Jürisson, Ali Kabir, Amaha Kahsay, Molla Kahssay, Rizwan Kalani, André Karch, Amir Kasaeian, Getachew Mullu Kassa, Tesfaye Dessale Kassa, Zemenu Yohannes Kassa, Andre Pascal Kengne, Yousef Saleh Khader, Morteza Abdullatif Khafaie, Nauman Khalid, Ibrahim Khalil, Ejaz Ahmad Khan, Muhammad Shahzeb Khan, Young-Ho Khang, Habibolah Khazaie, Abdullah T Khoja, Jagdish Khubchandani, Aliasghar A Kiadaliri, Daniel Kim, Young-Eun Kim, Adnan Kisa, Ai Koyanagi, Kristopher J Krohn, Barthelemy Kuate Defo, Burcu Kucuk Bicer, G Anil Kumar, Manasi Kumar, Ratilal Lalloo, Faris Hasan Lami, Van C Lansingh, Dennis Odai Laryea, Arman Latifi, Cheru Tesema Leshargie, Miriam Levi, Shanshan Li, Misgan Legesse Liben, Paulo A Lotufo, Raimundas Lunevicius, Narayan Bahadur Mahotra, Marek Majdan, Azeem Majeed, Reza Malekzadeh, Ana-Laura Manda, Mohammad Ali Mansournia, Benjamin Ballard Massenburg, Kedar K V Mate, Man Mohan Mehndiratta, Varshil Mehta, Hagazi Meles, Addisu Melese, Peter T N Memiah, Walter Mendoza, Getnet Mengistu, Atte Meretoja, Tuomo J Meretoja, Tomislav Mestrovic, Tomasz Miazgowski, Ted R Miller, GK Mini, Andreea Mirica, Erkin M Mirrakhimov, Babak Moazen, Moslem Mohammadi, Mariam Molokhia, Lorenzo Monasta, Stefania Mondello, Mahmood Moosazadeh, Ghobad Moradi, Mahmoudreza Moradi, Maziar Moradi-Lakeh, Mehdi Moradinazar, Shane Douglas Morrison, Marilita M Moschos, Seyyed Meysam Mousavi, Srinivas Murthy, Kamarul Imran Musa, Ghulam Mustafa, Mohsen Naghavi, Gurudatta Naik, Farid Najafi, Vinay Nangia, Bruno Ramos Nascimento, Ionut Negoi, Trang Huyen Nguyen, Emma Nichols, Dina Nur Anggraini Ningrum, Yirga Legesse Nirayo, Peter S Nyasulu, Felix Akpojene Ogbo, In-Hwan Oh, Anselm Okoro, Andrew T Olagunju, Tinuke O Olagunju, Pedro R Olivares, Stanislav S Otstavnov, Mayowa Ojo Owolabi, Mahesh P A, Smita Pakhale, Achyut Raj Pandey, Konrad Pesudovs, Gabriel D Pinilla-Monsalve, Suzanne Polinder, Hossein Poustchi, Swayam Prakash, Mostafa Qorbani, Amir Radfar, Anwar Rafay, Alireza Rafiei, Afarin Rahimi-Movaghar, Vafa Rahimi-Movaghar, Mahfuzar Rahman, Muhammad Aziz Rahman, Rajesh Kumar Rai, Fatemeh Rajati, Usha Ram, David Laith Rawaf, Salman Rawaf, Robert C Reiner, Cesar Reis, Andre M N Renzaho, Serge Resnikoff, Satar Rezaei, Shahab Rezaeian, Leonardo Roever, Luca Ronfani, Gholamreza Roshandel, Nobhojit Roy, George Mugambage Ruhago, Basema Saddik, Hosein Safari, Saeid Safiri, Mohammad Ali Sahraian, Payman Salamati, Raphael de Freitas Saldanha, Abdallah M Samy, Juan Sanabria, João Vasco Santos, Milena M M Santric Milicevic, Benn Sartorius, Maheswar Satpathy, Kim Savuon, Ione J C Schneider, David C Schwebel, Sadaf G Sepanlou, Hosein Shabaninejad, Masood A Ali Shaikh, Mehran Shams-Beyranvand, Mehdi Sharif, Mahdi Sharif-Alhoseini, Sheikh Mohammed Shariful Islam, Jun She, Aziz Sheikh, Jiabin Shen, Kevin N Sheth, Kenji Shibuya, Mekonnen Sisay Shiferaw, Mika Shigematsu, Rahman Shiri, Ivy Shiue, Haitham Shoman, Soraya Siabani, Tariq J Siddiqi, João Pedro Silva, Dayane Gabriele Alves Silveira, Dhirendra Narain Sinha, Mari Smith, Adauto Martins Soares Filho, Soheila Sobhani, Moslem Soofi, Joan B Soriano, Ireneous N Soyiri, Dan J Stein, Mark A Stokes, Mu'awiyyah Babale Sufiyan, Bruno F Sunguya, Jacob E Sunshine, Bryan L Sykes, Cassandra E I Szoeke, Rafael Tabarés-Seisdedos, Braden James Te Ao, Arash Tehrani-Banihashemi, Merhawi Gebremedhin Tekle, Mohamad-Hani Temsah, Omar Temsah, Roman Topor-Madry, Miguel - - Tortajada-Girbés, Bach Xuan Tran, Khanh Bao Tran, Lorainne Tudor Car, Kingsley Nnanna Ukwaja, Irfan Ullah, Muhammad Shariq Usman, Olalekan A Uthman, Pascual R Valdez, Tommi Juhani Vasankari, Narayanaswamy Venketasubramanian, Francesco S Violante, Fasil Wagnew Shiferaw Wagnew, Yasir Waheed, Yuan-Pang Wang, Kidu Gidey Weldegwergs, Andrea Werdecker, Tissa Wijeratne, Andrea Sylvia Winkler, Grant M A Wyper, Yuichiro Yano, Mehdi Yaseri, Yasin Jemal Yasin, Pengpeng Ye, Ebrahim M Yimer, Paul Yip, Engida Yisma, Naohiro Yonemoto, Seok-Jun Yoon, Michael G Yost, Mustafa Z Younis, Mahmoud Yousefifard, Chuanhua Yu, Zoubida Zaidi, Sojib Bin Zaman, Mohammad Zamani, Zerihun Menlkalew Zenebe, Sanjay Zodpey, Valery L Feigin, Theo Vos, Christopher J L Murray

## Abstract

**Background:**

Traumatic brain injury (TBI) and spinal cord injury (SCI) are increasingly recognised as global health priorities in view of the preventability of most injuries and the complex and expensive medical care they necessitate. We aimed to measure the incidence, prevalence, and years of life lived with disability (YLDs) for TBI and SCI from all causes of injury in every country, to describe how these measures have changed between 1990 and 2016, and to estimate the proportion of TBI and SCI cases caused by different types of injury.

**Methods:**

We used results from the Global Burden of Diseases, Injuries, and Risk Factors (GBD) Study 2016 to measure the global, regional, and national burden of TBI and SCI by age and sex. We measured the incidence and prevalence of all causes of injury requiring medical care in inpatient and outpatient records, literature studies, and survey data. By use of clinical record data, we estimated the proportion of each cause of injury that required medical care that would result in TBI or SCI being considered as the nature of injury. We used literature studies to establish standardised mortality ratios and applied differential equations to convert incidence to prevalence of long-term disability. Finally, we applied GBD disability weights to calculate YLDs. We used a Bayesian meta-regression tool for epidemiological modelling, used cause-specific mortality rates for non-fatal estimation, and adjusted our results for disability experienced with comorbid conditions. We also analysed results on the basis of the Socio-demographic Index, a compound measure of income per capita, education, and fertility.

**Findings:**

In 2016, there were 27·08 million (95% uncertainty interval [UI] 24·30–30·30 million) new cases of TBI and 0·93 million (0·78–1·16 million) new cases of SCI, with age-standardised incidence rates of 369 (331–412) per 100 000 population for TBI and 13 (11–16) per 100 000 for SCI. In 2016, the number of prevalent cases of TBI was 55·50 million (53·40–57·62 million) and of SCI was 27·04 million (24·98–30·15 million). From 1990 to 2016, the age-standardised prevalence of TBI increased by 8·4% (95% UI 7·7 to 9·2), whereas that of SCI did not change significantly (−0·2% [–2·1 to 2·7]). Age-standardised incidence rates increased by 3·6% (1·8 to 5·5) for TBI, but did not change significantly for SCI (−3·6% [–7·4 to 4·0]). TBI caused 8·1 million (95% UI 6·0–10·4 million) YLDs and SCI caused 9·5 million (6·7–12·4 million) YLDs in 2016, corresponding to age-standardised rates of 111 (82–141) per 100 000 for TBI and 130 (90–170) per 100 000 for SCI. Falls and road injuries were the leading causes of new cases of TBI and SCI in most regions.

**Interpretation:**

TBI and SCI constitute a considerable portion of the global injury burden and are caused primarily by falls and road injuries. The increase in incidence of TBI over time might continue in view of increases in population density, population ageing, and increasing use of motor vehicles, motorcycles, and bicycles. The number of individuals living with SCI is expected to increase in view of population growth, which is concerning because of the specialised care that people with SCI can require. Our study was limited by data sparsity in some regions, and it will be important to invest greater resources in collection of data for TBI and SCI to improve the accuracy of future assessments.

**Funding:**

Bill & Melinda Gates Foundation.

## Introduction

Traumatic brain injury (TBI) and spinal cord injury (SCI) are increasingly considered to be important global health priorities.^1^ These injuries not only cause health loss and disability for individuals and their families, but also represent a burden to health-care systems and economies through lost productivity and high health-care costs.^2^ Given that the injuries that lead to TBI and SCI are frequently preventable, there is also value in measuring the extent to which different causes of injury lead to TBI or SCI to help to understand the effect that injury-prevention programmes could have.

Many epidemiological studies have been limited by difficulties in comprehensively measuring the incidence of cross-injury sequelae such as TBI and SCI, and have instead focused on the incidence of the causes of injury, such as falls, road injuries, and interpersonal violence.^3^ As a result, few comprehensive epidemiological assessments have been done across all sources of injury, despite increasing dialogue about the long-term neuropsychological consequences of concussions in young people and professional athletes playing sports and about the risk of TBI from head trauma in bicycle crashes and other causes of injury.^4^, ^5^ Epidemiological studies that have focused specifically on TBI and SCI without estimation of all potential causes of injury have identified substantial burdens, but are often limited by relying on locations where incidence data were available without adopting modelling strategies for estimation of the burden in locations where data were sparse.^6^, ^7^, ^8^, ^9^, ^10^, ^11^, ^12^ Epidemiological assessments have been done in low-income and low-middle-income countries but typically have been limited by poor availability of data.^7^, ^12^, ^13^ Few studies have reported age-standardised incidence rates, which would enable comparison between countries with different populations, and the studies that have reported such data showed that the incidence rates of TBI and SCI vary substantially between countries.^7^, ^12^ These studies have not measured the relative disability caused by different injuries over time; such data are important because, whereas injuries such as fractures might be disabling only in the short term, conditions such as cognitive impairment from TBI or paraplegia from SCI can leave patients with lifelong health loss. In general, measurement of the burden of TBI and SCI in greater geographical and demographic detail—and over time—is of substantial value.

**Research in context**

**Evidence before this study**

Previous epidemiological studies of the incidence and outcomes of traumatic brain injury (TBI) and spinal cord injury (SCI) have been limited by focusing on certain subpopulations, including only select injuries, or by providing estimates only for areas of the world with accessible data. Previous Global Burden of Diseases, Injuries, and Risk Factors (GBD) studies have reported the burden of injury by cause of injury, such as self-harm, road injuries, and falls, but have not reported results by nature of injury sustained as a result of those causes, including TBI and SCI. To date, no studies have systematically measured the burden of TBI and SCI globally for all countries, ages, and sexes through recent years and from all causes of injury. To identify sources of injury data that could inform an assessment of non-fatal burden from TBI and SCI, we used results from the GBD 2016 injuries estimation process, which included systematic reviews of injury incidence data for all causes of injury that were initially done for GBD 2010 and updated as new data and literature studies became available in GBD 2013, GBD 2015, and GBD 2016. Inclusion criteria for the systematic reviews were representative, population-based surveys; reporting of injuries incidence; and clinical records from general hospitals, outpatient primary care facilities, and health insurance claims when such data were available with injury diagnosis codes. In this study, we updated a previous review of injuries data done for the World Bank that contributed to GBD 2010, GBD 2013, and GBD 2015 by searching the Global Health Data Exchange for surveys, hospital datasets, and literature studies in any language that were tagged as having injury-related data up to Dec 31, 2016.

**Added value of this study**

In this study, we used for the first time the GBD 2016 framework to report estimates of the global, regional, and national burden in terms of incidence, prevalence, and years of life lived with disability of TBI and SCI for 195 countries and territories. We have provided these estimates globally, by region, and by Socio-demographic Index quintiles in 2016, as well as the percentage change since 1990. We also provide estimates of the proportions of TBI and SCI caused by different causes of injury for each geographical region in 2016. Although epidemiological assessments that focus on particular populations have been done, no other studies of TBI or SCI have provided estimates in this level of detail for all countries derived from a standardised, systematic approach. We were able to measure uncertainty in our estimates by using the uncertainty propogation methods used throughout the GBD study.

**Implications of all the available evidence**

Our estimates suggest that TBI and SCI are severely disabling injuries. The global burden of TBI increased significantly between 1990 and 2016, whereas that of SCI has not changed significantly over time in terms of age-standardised incidence and prevalence. Age-standardised incidence and prevalence of TBI and SCI were high in central Europe, eastern Europe, and central Asia; the incidence and prevalence of SCI were high in North America and western Europe. Addressing the global burden of these conditions requires improved efforts to decrease the causes of SCI and TBI (eg, fall-prevention strategies, reducing alcohol overuse, and improving road safety, all of which could help to prevent injuries or decrease injury severity) and improved access to, and quality of, medical and social care (which could improve survival and reduce morbidity). People with TBI or SCI can have other medical conditions that require close supervision and might benefit from rehabilitation and medical care to reduce disability. Hence, although injury prevention efforts are key, health-care systems should also anticipate a growing burden from caring for people with TBI and SCI. These conditions could necessitate special focus within health-care systems, because they can be medically complex and burdensome for patients, clinicians, and families. In the future, development of improved methods for surveillance of TBI and SCI will be important, particularly in low-income settings, as will development of methods to identify patients with TBI who do not seek medical care.

The Global Burden of Diseases, Injuries, and Risk Factors (GBD) study is the product of a global research collaboration that quantifies the effects of hundreds of diseases, injuries, and risk factors around the world, producing annual estimates of all-cause mortality, causes of death, non-fatal health outcomes, and risk factors. Within the GBD framework, estimates for TBI and SCI burden have not previously been available as reported results. Instead, these nature-of-injury codes were incorporated as part of the analytic process that computed disability and results were ultimately provided only by cause (eg, falls) rather than by nature of injury (eg, TBI). Here, we describe an approach for estimation of nature-specific non-fatal burden estimates for all injuries, and report the incidence, prevalence, and years of life lived with disability (YLDs) for TBI and SCI, as well as the proportion of TBI and SCI caused by different injuries by region.

## Methods

### Overview

Our approach to measuring TBI and SCI was developed within the GBD 2016 study framework. In the GBD 2016 study, standardised analytic methods were used to estimate incidence, prevalence, and YLDs by age, sex, cause, year, and location. The study was an attempt to use all accessible information about disease and injury occurrence, clinical course, and severity that passed a set of inclusion criteria. The comparability of data was optimised by adjusting for different case definitions, enforcing consistency between data for prevalence, incidence, and cause of death estimates, and predicting estimates for locations with sparse data by borrowing information from other locations and covariates. These methods, data, and criteria are described in more detail in other GBD 2016 reports.^3^, ^14^, ^15^, ^16^, ^17^

Detailed elements of the GBD methods for measurement of TBI and SCI (including case definitions and severity definitions), a flowchart for our TBI and SCI estimation, and overall GBD study methods are in appendix 1. The measurement of TBI and SCI burden had two key deviations from the standard GBD framework. First, the GBD cause hierarchy categorised both TBI and SCI as being a nature of injury as opposed to a cause of injury—ie, these conditions previously had been measured as consequences of causes of injury. For example, a cause, such as a fall, could lead to SCI. Historically falls have been measured and reported but the actual nature of injury (eg, TBI, ankle fracture) that occurred because of the fall has not been directly reported. This aspect of the GBD study design was consistent across other natures of injuries. Second, estimation of TBI and SCI deviated from the GBD study framework in terms of the measures that were reported for the conditions, because we do not estimate death from TBI or SCI. Although TBI and SCI can lead to death, they were not considered causes of death in the GBD 2016 framework. Instead, the cause of injury (eg, falls) that led to a nature of injury such as TBI was considered the cause of death. For example, an individual who had a fall, sustained a TBI, and then died while in hospital after the injury would be considered to have had a death caused by a fall and an incident TBI. In this study, we estimated the non-fatal burden and therefore report incidence, prevalence, and YLDs, but not cause-specific mortality or years of life lost.

### Cause-of-injury estimation

The process for estimation of incidence, prevalence, and YLDs was as follows. First, the incidence of 29 different causes of injury (appendix 1) were modelled with DisMod-MR 2.1, a meta-regression tool that was used extensively throughout the GBD study.^3^ These cause-of-injury models measured the incidence of each cause of injury that required medical care, which included patients who were admitted or seen in an outpatient clinic and received a diagnosis code for a given cause of injury. Receiving an injury diagnosis code did not preclude the possibility of death in the hospital or after discharge. Each of these cause models used an array of data types, including surveillance studies, literature studies, hospital discharge records, and emergency department records. The details of these models have previously been described in more detail.^3^ Although we do not estimate death from TBI or SCI in this study, our modelling strategy also included cause-specific mortality rates from the cause of death ensemble model to inform incidence estimates for cause-of-injury models such as road injuries in data-sparse areas using estimates from data-rich areas.^18^ The outputs from these models were estimates of inpatient (admitted) and outpatient incidence rates of causes of injury and were specific for location, sex, age, and year. The outpatient incidence of each cause was derived from the inpatient incidence on the basis of a regression coefficient for outpatient incidence that was extracted from DisMod-MR 2.1 incidence models in locations that had both inpatient and outpatient data.

### Nature-of-injury estimation

Clinical record data that coded for both cause and nature of injury were used to estimate the proportion of each cause that resulted in each nature of injury. If an injury cause resulted in more than one nature of injury, the most severe was chosen on the basis of a mixed-effects regression model that estimated the disability experienced by an injured individual adjusted for age, sex, and never-injured status, with country and individual random effects. Because SCI was associated with higher disability than TBI (appendix 1), SCI was chosen if both conditions occurred as a result of the same injury. We used this method after finding in a previous GBD study^19^ that statistically assigning multiple injury categories to a single individual was difficult because of a sparsity of data. This process and the severity rankings are described in more detail in appendix 1. These proportions were calculated for each external cause-of-injury–nature-of-injury (cause–nature) combination, such that the proportions of all natures of injury for a given cause of injury sum to 1 because of a Dirichlet regression. The output from this step was incidence for each cause–nature combination.

### Derivation of incidence, prevalence, and YLDs

From the incidence estimates for each cause–nature combination, we separately modelled short-term and long-term estimates using proportions of individuals expected to experience short-term versus long-term disability (the cutoff for long-term disability was 1 year). The proportions estimated to experience permanent health loss generally increased with age and were different for TBI and SCI (appendix 1). The short-term prevalence estimates were then calculated on the basis of average duration of a short-term case, whereas the long-term estimates were considered to be permanent and underwent comorbidity adjustment as described previously.^3^ Cause–nature incidence rates were converted to prevalence with the differential equation solver used in DisMod-MR 2.1. This solver reconciled the incidence rates from the previous steps with standardised mortality ratios derived from literature studies to estimate prevalence, because people with long-term disability due to TBI and SCI die at a higher rate than the background mortality in the population.^20^ The final output from this step was prevalence of each cause–nature combination for each location, year, age, and sex combination.

YLDs were then calculated by multiplying the prevalence by the disability weight. Measurement has been described in more detail previously, but in summary, disability weights were measured through population and internet surveys on the basis of lay descriptions of disabling conditions.^21^ For example, the disability weight for short-term mild TBI and for short-term moderate or severe TBI were 0·110 (95% uncertainty interval [UI] 0·074–0·158) and 0·214 (0·141–0·297), respectively, meaning that the affected people experienced health losses of 11·0% and 21·4%, respectively, compared with a person in full health. All disability weights for different severities of TBI and SCI are provided in appendix 1.

After estimation of YLDs, the prevalence, incidence, and YLDs for TBI and SCI were then summed across all causes to estimate the all-injury prevalence, incidence, and YLDs for TBI and SCI separately. Uncertainty was propagated throughout this process by maintaining distributions of 1000 draws for each estimation stage (including percentage change over time). We use the 25th and 975th sorted values in the draw distributions as the upper and lower UIs for mean estimates and for percentage change, whereby change was judged to be significant if the lower and upper UIs did not overlap zero. This process is consistent with management of uncertainty throughout the GBD study framework.^3^

### Statistical analysis

We grouped countries into quintiles on the basis of their 2016 Socio-demographic Index (SDI) value, which is a composite measure of development derived from income per person, educational attainment, and total fertility rate.^17^ Additionally, we measured the most common causes of TBI and SCI separately in terms of the original cause of injury that led to the disability. Finally, we measured the proportion of TBI that was mild versus the proportion that was moderate or severe and the proportion of SCI that occurred at the neck versus below the neck and present these values at the global level. Analyses were done in Python (version 2.7), Stata (version 13.1), and R (version 3.3). Statistical code used for this study will be made available upon publication of this Article via the Institute for Health Metrics and Evaluation. This study complies with the Guidelines for Accurate and Transparent Health Estimates Report (GATHER) recommendations (appendix 1).

### Role of the funding source

The funder of the study had no role in study design, data collection, data analysis, data interpretation, or the writing of the report. All authors had full access to the data in the study and had final responsibility for the decision to submit for publication.

## Results

We used incidence data for every cause of injury and every GBD region. The number of sources by injury and by region are in appendix 1. Incidence, prevalence, and YLD estimates for every cause of injury by age, sex, and location for 1990–2016 are available through an online results tool.

Table 1 shows the incidence and prevalence of TBI in terms of all-age counts, age-standardised rates (per 100 000 population), and percentage change in age-standardised rates between 1990 and 2016. Table 2 shows the same information for SCI. YLDs from TBI and SCI in terms of all-age counts, age-standardised rates, and total percentage change are in appendix 2, which also includes these estimates by age and sex, and for 1990. Between 1990 and 2016, age-standardised incidence rates significantly increased by 3·6% (95% UI 1·8 to 5·5) for TBI and decreased non-significantly by −3·6% (−7·4 to 4·0) for SCI, leading to age-standardised incidence rates of 369 (331 to 412) per 100 000 for TBI and 13 (11 to 16) per 100 000 for SCI (Table 1, Table 2).

**Table 1:** Incidence and prevalence of traumatic brain injury in 2016, and percentage change in age-standardised rates by location, 1990–2016

		**Incidence**			**Prevalence**		
		2016 counts	2016 age-standardised rates (per 100 000)	Percentage change in age-standardised rates, 1990–2016	2016 counts	2016 age-standardised rates (per 100 000)	Percentage change in age-standardised rates, 1990–2016
**Global**		**27 082 033 (24 302 091 to 30 298 710)**	**369 (331 to 412)**	**3·6 (1·8 to 5·5)**	**55 495 674 (53 400 547 to 57 626 214)**	**759 (731 to 788)**	**8·4 (7·7 to 9·2)**
High SDI		3 682 268 (3 112 645 to 4 394 060)	343 (293 to 403)	−9·4 (−12·2 to −6·2)	8 463 137 (8 121 296 to 8 818 355)	647 (619 to 675)	−7·9 (−8·7 to −7·1)
High-middle SDI		5 550 132 (4 977 125 to 6 205 225)	468 (419 to 523)	−10·7 (−13·3 to −8·4)	13 458 443 (12 951 567 to 13 973 353)	1032 (993 to 1 074)	−5·4 (−6·3 to −4·5)
Middle SDI		7 279 905 (6 580 600 to 8 046 104)	318 (287 to 351)	21·8 (18·8 to 24·9)	16 745 178 (16 127 494 to 17 364 295)	699 (674 to 725)	32·4 (31·0 to 33·9)
Low-middle SDI		8 074 189 (7 244 954 to 8 969 510)	397 (356 to 441)	11·1 (7·6 to 16·5)	13 524 272 (12 971 769 to 14 124 365)	747 (717 to 778)	18·7 (17·7 to 20·0)
Low SDI		2 607 230 (2 291 622 to 2 997 764)	366 (323 to 416)	−9·3 (−14·7 to −6·0)	3 506 690 (3 308 885 to 3 760 344)	669 (633 to 719)	3·3 (1·9 to 4·8)
**High income**		**3 274 760 (2 736 209 to 3 975 372)**	**298 (251 to 354)**	**−9·6 (−13·0 to −6·1)**	**7 330 041 (7 013 363 to 7 655 518)**	**544 (520 to 569)**	**−10·2 (−11·0 to −9·2)**
**High-income North America**		**1 221 494 (1 019 814 to 1 475 250)**	**329 (277 to 392)**	**−4·1 (−8·8 to 1·2)**	**2 603 351 (2 488 042 to 2 726 560)**	**600 (573 to 630)**	**−6·3 (−7·9 to −4·6)**
	Canada	110 332 (92 166 to 133 581)	302 (254 to 361)	−10·4 (−15·2 to −6·0)	253 144 (241 660 to 265 695)	558 (532 to 586)	−11·2 (−12·9 to −9·2)
	Greenland	161 (133 to 197)	321 (267 to 389)	−19·0 (−21·3 to −16·5)	281 (269 to 296)	525 (502 to 553)	−14·6 (−15·9 to −13·2)
	USA	1 110 578 (927 814 to 1 340 515)	333 (280 to 396)	−3·3 (−8·2 to 2·5)	2 349 017 (2 244 955 to 2 461 041)	605 (577 to 635)	−5·7 (−7·5 to −3·9)
**Australasia**		**78 554 (65 710 to 93 741)**	**276 (231 to 327)**	**−13·0 (−18·6 to −7·3)**	**178 663 (170 767 to 187 588)**	**528 (503 to 556)**	**−14·1 (−15·6 to −12·2)**
	Australia	66 020 (55 309 to 78 895)	275 (230 to 327)	−12·1 (−17·8 to −6·3)	150 213 (143 534 to 157 799)	527 (503 to 555)	−13·3 (−14·9 to −11·3)
	New Zealand	12 535 (10 560 to 14 974)	279 (236 to 330)	−17·1 (−23·2 to −11·1)	28 450 (27 152 to 29 828)	534 (508 to 561)	−18·1 (−20·3 to −16·1)
**High-income Asia Pacific**		**563 538 (471 687 to 681 672)**	**276 (231 to 330)**	**−16·9 (−20·6 to −13·1)**	**1 256 353 (1 203 704 to 1 308 375)**	**489 (467 to 511)**	**−14·8 (−16·1 to −13·5)**
	Brunei	1535 (1306 to 1819)	384 (325 to 456)	−20·7 (−24·6 to −16·6)	2708 (2564 to 2857)	673 (640 to 708)	−21·3 (−22·7 to −19·9)
	Japan	382 954 (317 505 to 467 002)	263 (220 to 314)	−15·5 (−19·9 to −11·2)	891 110 (854 680 to 928 073)	474 (452 to 496)	−14·2 (−15·8 to −12·4)
	Singapore	11 193 (9348 to 13 379)	285 (238 to 340)	−4·4 (−10·0 to 0·6)	24 309 (23 165 to 25 421)	516 (491 to 540)	−0·8 (−3·0 to 1·5)
	South Korea	167 856 (141 874 to 199 972)	316 (267 to 377)	−19·4 (−23·8 to −14·7)	338 225 (323 467 to 352 938)	535 (510 to 559)	−18·4 (−20·1 to −17·0)
**Western Europe**		**1 262 700 (1 042 418 to 1 546 907)**	**292 (244 to 351)**	**−13·4 (−17·2 to −9·7)**	**3 021 435 (2 880 245 to 3 154 517)**	**546 (519 to 572)**	**−12·8 (−13·8 to −11·7)**
	Andorra	236 (194 to 292)	300 (249 to 361)	4·1 (0·8 to 7·5)	583 (554 to 611)	565 (536 to 594)	6·2 (4·7 to 7·7)
	Austria	28 255 (23 166 to 35 170)	322 (266 to 388)	−19·9 (−24·5 to −14·8)	66 670 (63 606 to 69 564)	589 (561 to 616)	−17·7 (−19·4 to −16·2)
	Belgium	41 126 (33 848 to 51 024)	344 (287 to 416)	−6·8 (−13·6 to −0·6)	90 487 (86 268 to 94 639)	621 (590 to 652)	−10·1 (−11·7 to −8·1)
	Cyprus	2959 (2503 to 3516)	323 (273 to 381)	−10·0 (−14·2 to −5·6)	6605 (6280 to 6947)	618 (588 to 650)	−9·0 (−10·3 to −7·6)
	Denmark	17 302 (14 208 to 21 444)	301 (249 to 366)	−14·9 (−19·7 to −10·0)	39 756 (37 914 to 41 590)	556 (529 to 584)	−12·5 (−14·0 to −11·0)
	Finland	20 009 (16 226 to 25 470)	344 (284 to 420)	−5·5 (−10·4 to −0·8)	44 056 (42 012 to 46 103)	609 (579 to 637)	−4·5 (−6·3 to −2·8)
	France	209 986 (170 948 to 261 143)	307 (255 to 372)	−19·9 (−24·6 to −15·3)	466 018 (442 758 to 488 496)	564 (535 to 593)	−19·8 (−21·4 to −18·1)
	Germany	236 043 (192 862 to 295 657)	288 (239 to 346)	−12·8 (−18·4 to −7·7)	592 273 (564 640 to 619 584)	535 (508 to 562)	−12·6 (−14·2 to −11·1)
	Greece	33 094 (27 874 to 39 411)	321 (271 to 382)	−9·7 (−14·8 to −4·8)	89 972 (85 526 to 94 453)	627 (594 to 660)	−7·9 (−9·2 to −6·4)
	Iceland	926 (766 to 1120)	282 (233 to 339)	−7·5 (−11·9 to −2·8)	2021 (1925 to 2110)	532 (506 to 557)	−7·1 (−8·8 to −5·3)
	Ireland	13 298 (10 956 to 16 111)	297 (246 to 356)	−6·0 (−11·1 to −0·6)	28 873 (27 464 to 30 268)	550 (523 to 578)	−6·5 (−8·1 to −4·9)
	Israel	22 803 (18 980 to 27 302)	278 (232 to 332)	−4·5 (−11·5 to 1·3)	45 734 (42 915 to 49 699)	556 (521 to 605)	4·0 (1·0 to 8·4)
	Italy	191 527 (158 858 to 231 854)	315 (263 to 377)	−11·7 (−15·7 to −7·3)	491 141 (468 037 to 514 611)	596 (566 to 626)	−10·2 (−11·7 to −8·7)
	Luxembourg	1771 (1461 to 2157)	303 (252 to 367)	−26·5 (−31·5 to −21·9)	3980 (3783 to 4168)	563 (535 to 591)	−25·7 (−27·1 to −24·3)
	Malta	1170 (965 to 1445)	289 (240 to 352)	−8·9 (−12·4 to −5·1)	2870 (2741 to 3000)	539 (514 to 566)	−5·7 (−7·1 to −4·4)
	Netherlands	46 656 (38 792 to 56 158)	275 (233 to 327)	0·0 (−5·4 to 5·8)	112 886 (107 893 to 118 007)	523 (499 to 549)	1·2 (−0·8 to 3·1)
	Norway	15 956 (13 059 to 19 836)	298 (246 to 363)	−5·5 (−10·5 to −0·9)	34 915 (33 251 to 36 587)	547 (519 to 574)	−4·3 (−5·8 to −2·5)
	Portugal	28 078 (23 484 to 33 755)	267 (226 to 316)	−29·3 (−34·6 to −24·6)	70 982 (67 792 to 74 142)	504 (480 to 527)	−28·5 (−30·3 to −26·6)
	Spain	128 447 (107 057 to 155 547)	284 (237 to 339)	−16·4 (−21·3 to −11·4)	328 217 (312 784 to 343 173)	543 (515 to 569)	−16·5 (−18·0 to −14·6)
	Sweden	28 106 (22 808 to 34 819)	282 (233 to 342)	−4·6 (−9·1 to −0·1)	63 463 (60 718 to 66 262)	512 (488 to 536)	−5·9 (−7·6 to −4·2)
	Switzerland	25 123 (20 227 to 31 494)	284 (233 to 343)	−29·1 (−33·8 to −24·9)	54 812 (52 351 to 57 096)	499 (476 to 520)	−29·4 (−30·9 to −27·9)
	UK	168 579 (137 783 to 208 313)	260 (215 to 316)	−5·9 (−9·9 to −2·0)	382 133 (364 581 to 399 049)	478 (454 to 499)	−6·5 (−7·5 to −5·3)
**Southern Latin America**		**148 473 (124 980 to 179 016)**	**225 (190 to 271)**	**11·7 (8·7 to 14·8)**	**270 239 (259 242 to 281 576)**	**392 (376 to 409)**	**15·1 (13·9 to 16·5)**
	Argentina	100 117 (84 470 to 120 244)	228 (192 to 273)	14·0 (9·8 to 18·2)	179 575 (172 271 to 187 296)	401 (384 to 418)	18·0 (16·4 to 19·7)
	Chile	39 688 (32 980 to 47 814)	214 (178 to 257)	4·4 (1·1 to 7·7)	74 082 (71 084 to 77 306)	368 (352 to 384)	8·0 (6·3 to 9·5)
	Uruguay	8662 (7243 to 10 453)	244 (205 to 294)	13·8 (9·4 to 18·3)	16 567 (15 878 to 17 280)	424 (406 to 443)	17·0 (15·2 to 19·0)
**Central Europe, eastern Europe, and central Asia**		**3** **174** **597 (2 813 645 to 3 622 489)**	**740 (657 to 844)**	**−4·2 (−6·3 to −2·2)**	**7 505 017 (7 139 954 to 7 872 426)**	**1539 (1464 to 1614)**	−0·6 (−1·5 to 0·6)
**Eastern Europe**		**1 679 786 (1 495 412 to 1 908 308)**	**772 (688 to 876)**	**−2·4 (−5·2 to 0·3)**	**3 987 022 (3 796 684 to 4 175 331)**	**1546 (1472 to 1623)**	**−1·5 (−3·0 to 0·2)**
	Belarus	85 268 (74 665 to 98 805)	853 (749 to 979)	15·8 (11·5 to 20·3)	203 206 (192 572 to 213 647)	1724 (1632 to 1815)	15·2 (12·9 to 17·9)
	Estonia	9843 (8618 to 11 372)	722 (635 to 827)	−20·2 (−24·0 to −16·0)	25 698 (24 398 to 27 131)	1529 (1445 to 1622)	−14·1 (−16·2 to −11·8)
	Latvia	15 511 (13 648 to 17 822)	743 (654 to 851)	−22·6 (−26·0 to −18·8)	38 996 (37 015 to 40 989)	1519 (1436 to 1601)	−18·4 (−20·2 to −16·6)
	Lithuania	26 381 (23 106 to 30 541)	845 (743 to 972)	−5·5 (−9·4 to −1·3)	64 738 (61 519 to 68 064)	1709 (1618 to 1800)	−3·8 (−5·7 to −1·9)
	Moldova	25 099 (22 243 to 28 672)	609 (537 to 696)	−17·6 (−21·2 to −14·0)	58 867 (55 632 to 62 363)	1251 (1181 to 1327)	−14·2 (−16·4 to −12·0)
	Russia	1 202 502 (1 074 273 to 1 364 131)	799 (715 to 905)	−1·5 (−4·9 to 2·2)	2 810 261 (2 678 210 to 2 939 902)	1589 (1512 to 1668)	−0·6 (−2·5 to 1·6)
	Ukraine	315 182 (278 598 to 360 233)	683 (605 to 779)	−6·5 (−10·0 to −2·8)	785 255 (745 321 to 824 796)	1390 (1317 to 1464)	−6·1 (−8·1 to −4·1)
**Central Europe**		**1 055 830 (916 104 to 1 233 304)**	**857 (750 to 988)**	**−3·0 (−5·9 to −0·4)**	**2 649 259 (2 512 485 to 2 792 424)**	**1797 (1699 to 1895)**	**4·4 (3·3 to 5·5)**
	Albania	19 366 (17 024 to 22 180)	662 (583 to 761)	10·1 (6·5 to 13·9)	48 963 (46 227 to 51 786)	1490 (1405 to 1577)	12·6 (9·7 to 16·1)
	Bosnia and Herzegovina	25 864 (22 525 to 29 894)	688 (599 to 793)	42·4 (37·9 to 46·7)	76 617 (71 724 to 82 170)	1600 (1493 to 1714)	53·9 (47·9 to 62·5)
	Bulgaria	57 125 (49 635 to 66 125)	776 (683 to 897)	−5·3 (−9·4 to −1·3)	157 661 (149 402 to 165 850)	1663 (1572 to 1756)	−1·8 (−3·8 to 0·6)
	Croatia	39 226 (33 726 to 46 071)	801 (704 to 914)	−3·1 (−8·3 to 2·5)	92 177 (87 994 to 96 575)	1627 (1553 to 1703)	0·1 (−2·8 to 3·3)
	Czech Republic	115 120 (98 857 to 135 307)	1022 (885 to 1191)	−5·3 (−9·8 to −0·3)	297 221 (281 750 to 314 241)	2174 (2051 to 2309)	9·1 (6·7 to 11·5)
	Hungary	96 761 (82 027 to 115 438)	865 (744 to 1018)	−19·0 (−23·5 to −14·7)	221 514 (209 001 to 234 108)	1707 (1606 to 1810)	−8·8 (−11·2 to −6·4)
	Macedonia	14 795 (12 858 to 17 014)	714 (621 to 821)	16·0 (11·9 to 20·0)	37 193 (35 009 to 39 447)	1526 (1434 to 1621)	18·3 (15·2 to 21·3)
	Montenegro	4976 (4367 to 5690)	785 (688 to 897)	8·5 (5·3 to 11·7)	12 611 (11 930 to 13 318)	1712 (1615 to 1810)	11·5 (9·3 to 13·9)
	Poland	370 019 (319 509 to 432 391)	893 (782 to 1038)	1·0 (−3·6 to 5·2)	908 548 (860 062 to 960 247)	1856 (1753 to 1964)	9·1 (6·7 to 11·3)
	Romania	169 215 (146 358 to 196 891)	834 (729 to 966)	−7·9 (−12·5 to −3·4)	434 844 (411 374 to 458 018)	1751 (1653 to 1847)	−5·0 (−6·9 to −3·1)
	Serbia	65 967 (57 683 to 75 848)	733 (642 to 840)	13·4 (10·0 to 16·7)	174 644 (164 893 to 184 515)	1604 (1511 to 1705)	17·9 (14·9 to 21·6)
	Slovakia	51 215 (44 217 to 60 241)	889 (775 to 1031)	−9·9 (−13·3 to −6·0)	123 805 (117 261 to 130 731)	1831 (1730 to 1936)	−2·0 (−3·9 to 0·4)
	Slovenia	26 182 (22 098 to 31 703)	1092 (938 to 1294)	−3·8 (−9·3 to 3·1)	63 460 (60 084 to 67 002)	2255 (2133 to 2384)	3·2 (1·3 to 5·5)
**Central Asia**		**438 981 (389 647 to 498 547)**	**495 (439 to 563)**	**0·1 (−2·3 to 2·4)**	**868 736 (823 691 to 916 878)**	**1054 (1002 to 1109)**	**1·9 (0·7 to 3·2)**
	Armenia	14 329 (12 636 to 16 274)	474 (417 to 539)	−12·4 (−16·1 to −8·6)	39 865 (36 019 to 45 144)	1171 (1057 to 1325)	−13·3 (−17·4 to −9·4)
	Azerbaijan	45 533 (40 390 to 51 890)	465 (410 to 530)	−3·4 (−6·9 to 0·1)	101 481 (95 966 to 107 296)	1008 (954 to 1065)	1·6 (−0·9 to 4·5)
	Georgia	20 209 (17 948 to 22 994)	496 (440 to 563)	−4·6 (−8·2 to −1·3)	48 707 (46 371 to 51 143)	1046 (994 to 1100)	−3·0 (−5·6 to −0·1)
	Kazakhstan	108 784 (96 745 to 122 858)	609 (541 to 689)	9·8 (5·7 to 14·2)	217 996 (206 528 to 229 023)	1251 (1187 to 1313)	11·3 (8·8 to 13·9)
	Kyrgyzstan	28 144 (24 869 to 31 895)	470 (417 to 532)	−13·1 (−16·7 to −9·6)	51 346 (48 550 to 54 313)	989 (940 to 1043)	−9·6 (−12·0 to −7·2)
	Mongolia	19 388 (17 259 to 22 037)	634 (563 to 723)	51·3 (43·5 to 58·0)	34 544 (32 803 to 36 466)	1258 (1197 to 1325)	52·1 (47·4 to 56·6)
	Tajikistan	35 891 (31 281 to 41 437)	417 (364 to 482)	−13·3 (−16·9 to −9·8)	62 285 (58 266 to 66 604)	918 (861 to 978)	−6·9 (−10·1 to −2·7)
	Turkmenistan	24 881 (22 055 to 28 455)	448 (398 to 512)	−0·2 (−3·7 to 3·6)	46 815 (44 152 to 49 691)	945 (894 to 999)	3·6 (1·0 to 5·9)
	Uzbekistan	141 821 (125 591 to 160 782)	459 (405 to 521)	4·0 (0·1 to 7·5)	265 697 (251 467 to 282 482)	963 (913 to 1019)	5·4 (3·0 to 7·9)
**Latin America and Caribbean**		**1 845 785 (1 656 712 to 2 074 570)**	**330 (296 to 372)**	**1·1 (−0·9 to 3·3)**	**3 721 363 (3 549 097 to 3 887 453)**	**681 (650 to 710)**	**3·6 (2·2 to 4·9)**
**Central Latin America**		**716 600 (642 948 to 806 959)**	**293 (263 to 330)**	**−9·5 (−11·6 to −7·6)**	**1 412 146 (1 349 779 to 1 474 463)**	**609 (584 to 635)**	**−5·0 (−5·9 to −3·9)**
	Colombia	139 297 (124 785 to 157 283)	294 (263 to 331)	−8·7 (−12·1 to −5·1)	300 952 (287 367 to 315 562)	632 (605 to 663)	−1·7 (−4·0 to 0·3)
	Costa Rica	15 073 (13 458 to 17 031)	316 (281 to 357)	18·3 (13·8 to 23·1)	33 681 (31 961 to 35 326)	677 (643 to 710)	24·8 (21·5 to 28·3)
	El Salvador	19 120 (16 989 to 21 613)	317 (282 to 359)	1·0 (−7·3 to 7·0)	37 724 (35 524 to 40 291)	660 (622 to 705)	5·5 (−0·5 to 9·6)
	Guatemala	45 833 (40 822 to 51 724)	301 (269 to 341)	14·2 (6·5 to 19·9)	73 308 (69 788 to 77 152)	593 (568 to 620)	19·1 (15·0 to 22·4)
	Honduras	20 923 (18 552 to 23 757)	279 (246 to 317)	30·4 (25·3 to 36·2)	37 596 (34 937 to 40 813)	567 (532 to 610)	33·1 (26·4 to 42·1)
	Mexico	341 669 (306 552 to 384 904)	279 (249 to 315)	−19·7 (−21·8 to −17·7)	658 215 (628 280 to 687 951)	565 (540 to 589)	−16·9 (−18·0 to −15·6)
	Nicaragua	15 254 (13 573 to 17 337)	263 (233 to 299)	4·8 (1·1 to 8·7)	29 229 (27 333 to 31 642)	564 (531 to 609)	−0·0 (−5·3 to 4·5)
	Panama	12 357 (11 042 to 13 915)	315 (281 to 354)	19·1 (15·2 to 23·4)	25 833 (24 514 to 27 043)	683 (649 to 715)	24·0 (20·8 to 27·1)
	Venezuela	107 073 (96 052 to 119 963)	348 (313 to 390)	14·0 (9·4 to 18·7)	215 609 (205 565 to 225 532)	742 (709 to 775)	19·6 (16·8 to 22·5)
**Andean Latin America**		**175 372 (157 046 to 195 338)**	**303 (271 to 340)**	**7·9 (4·7 to 11·2)**	**338 065 (320 850 to 354 351)**	**640 (610 to 670)**	**12·1 (10·2 to 14·0)**
	Bolivia	31 106 (27 863 to 34 867)	294 (262 to 329)	−4·0 (−7·0 to −1·2)	55 875 (53 030 to 58 747)	608 (579 to 638)	2·6 (0·2 to 5·1)
	Ecuador	55 753 (49 257 to 63 120)	350 (310 to 398)	16·5 (9·0 to 26·0)	101 553 (96 588 to 106 302)	696 (663 to 728)	11·8 (9·3 to 14·3)
	Peru	88 513 (78 933 to 98 960)	282 (252 to 316)	6·8 (2·3 to 11·4)	180 637 (170 825 to 189 957)	622 (590 to 652)	15·1 (11·8 to 18·3)
**Caribbean**		**145 899 (130 066 to 163 629)**	**320 (285 to 360)**	**21·1 (17·9 to 24·5)**	**322 291 (292 210 to 359 914)**	**706 (640 to 788)**	**29·9 (20·2 to 44·6)**
	Antigua and Barbuda	263 (235 to 295)	291 (259 to 326)	15·4 (12·3 to 18·8)	596 (566 to 625)	640 (607 to 671)	18·5 (15·6 to 21·4)
	The Bahamas	1222 (1097 to 1368)	314 (281 to 352)	6·3 (2·9 to 9·8)	2720 (2591 to 2843)	660 (629 to 690)	11·2 (8·6 to 14·5)
	Barbados	782 (697 to 875)	275 (246 to 309)	19·2 (16·2 to 22·5)	1995 (1899 to 2084)	600 (569 to 628)	23·1 (19·9 to 26·3)
	Belize	1148 (1029 to 1282)	321 (289 to 358)	32·3 (26·4 to 37·1)	1969 (1873 to 2056)	659 (629 to 685)	31·7 (28·6 to 35·3)
	Bermuda	226 (201 to 253)	322 (286 to 361)	−4·0 (−7·5 to −0·2)	515 (490 to 539)	719 (684 to 752)	5·0 (2·4 to 7·7)
	Cuba	41 964 (36 486 to 48 735)	338 (296 to 387)	12·5 (8·0 to 17·7)	96 307 (91 245 to 101 339)	683 (646 to 719)	10·6 (7·6 to 13·9)
	Dominica	210 (188 to 236)	284 (254 to 319)	32·7 (29·1 to 36·5)	458 (434 to 481)	610 (578 to 641)	35·5 (31·6 to 40·0)
	Dominican Republic	32 270 (28 928 to 36 034)	308 (277 to 343)	31·7 (27·7 to 36·2)	65 016 (61 686 to 68 195)	678 (645 to 709)	34·1 (30·7 to 37·7)
	Grenada	333 (296 to 373)	317 (283 to 356)	29·8 (26·2 to 33·5)	643 (609 to 674)	645 (612 to 674)	31·5 (28·5 to 34·6)
	Guyana	2297 (2055 to 2582)	307 (276 to 344)	15·4 (11·7 to 19·3)	4176 (3972 to 4380)	589 (561 to 616)	19·1 (16·5 to 22·0)
	Haiti	31 804 (27 952 to 36 334)	289 (255 to 327)	20·1 (12·3 to 31·0)	71 467 (48 284 to 108 152)	748 (530 to 1087)	69·6 (20·6 to 144·6)
	Jamaica	7721 (6895 to 8611)	268 (239 to 299)	38·9 (35·5 to 42·7)	16 275 (15 350 to 17 055)	580 (548 to 608)	38·2 (33·7 to 42·4)
	Puerto Rico	13 213 (11 678 to 15 024)	346 (307 to 391)	21·8 (17·1 to 27·0)	31 248 (29 568 to 32 767)	731 (690 to 766)	23·4 (19·7 to 27·1)
	Saint Lucia	526 (469 to 592)	291 (260 to 328)	20·1 (16·8 to 23·4)	1199 (1141 to 1258)	632 (602 to 663)	25·2 (21·9 to 28·7)
	Saint Vincent and the Grenadines	342 (305 to 387)	312 (279 to 352)	29·1 (25·3 to 32·8)	698 (661 to 731)	639 (606 to 668)	30·9 (27·2 to 34·7)
	Suriname	1684 (1511 to 1885)	313 (281 to 351)	26·1 (22·2 to 30·2)	3444 (3284 to 3595)	652 (623 to 680)	27·0 (23·5 to 30·3)
	Trinidad and Tobago	4111 (3684 to 4589)	312 (280 to 348)	24·9 (19·7 to 29·9)	9781 (9298 to 10 211)	661 (628 to 691)	33·0 (29·5 to 36·5)
	Virgin Islands	343 (306 to 389)	309 (276 to 348)	15·2 (11·6 to 18·8)	834 (792 to 874)	640 (607 to 672)	16·4 (13·9 to 19·8)
**Tropical Latin America**		**807 914 (720 908 to 913 966)**	**382 (340 to 432)**	**6·2 (3·0 to 9·8)**	**1 648 860 (1 572 072 to 1 728 016)**	**763 (728 to 798)**	**5·4 (3·0 to 7·5)**
	Brazil	786 433 (701 498 to 889 704)	383 (341 to 434)	5·6 (2·4 to 9·2)	1 608 456 (1 533 394 to 1 684 669)	764 (729 to 801)	4·9 (2·4 to 7·0)
	Paraguay	21 481 (18 974 to 24 600)	330 (293 to 376)	36·9 (32·6 to 41·7)	40 404 (38 084 to 42 978)	692 (655 to 731)	33·7 (29·4 to 38·3)
**Southeast Asia, east Asia, and Oceania**		**6 356 051 (5 736 733 to 7 010 413)**	**302 (273 to 332)**	**31·1 (26·8 to 35·5)**	**16 424 025 (15 779 923 to 17 082 254)**	**714 (687 to 743)**	**43·0 (41·0 to 45·4)**
**East Asia**		**4 481 454 (4 033 188 to 4 949 337)**	**312 (282 to 344)**	**33·3 (28·5 to 38·3)**	**12 301 082 (11 843 999 to 12 776 357)**	**739 (712 to 768)**	**43·5 (41·4 to 45·7)**
	China	4 339 654 (3 905 674 to 4 790 917)	313 (283 to 345)	33·1 (28·3 to 38·1)	11 931 974 (11 487 676 to 12 391 509)	742 (715 to 771)	43·6 (41·4 to 45·8)
	North Korea	71 712 (64 835 to 79 836)	267 (241 to 297)	56·4 (49·9 to 63·1)	163 389 (156 812 to 170 561)	590 (566 to 616)	54·1 (50·3 to 57·6)
	Taiwan (province of China)	70 088 (63 129 to 77 594)	296 (267 to 328)	26·2 (21·3 to 31·9)	205 719 (197 291 to 213 773)	708 (679 to 737)	31·1 (28·2 to 34·1)
**Southeast Asia**		**1 843 182 (1 667 459 to 2 039 489)**	**283 (256 to 312)**	**27·1 (20·9 to 32·0)**	**4 070 463 (3 880 114 to 4 273 779)**	**649 (620 to 680)**	**42·8 (40·1 to 46·0)**
	Cambodia	41 142 (37 163 to 45 610)	263 (238 to 291)	25·3 (6·8 to 38·0)	80 281 (73 748 to 91 402)	615 (560 to 713)	24·4 (5·0 to 41·4)
	Indonesia	672 105 (606 726 to 743 145)	264 (238 to 292)	25·2 (21·4 to 29·1)	1 453 365 (1 384 542 to 1 525 952)	595 (569 to 624)	35·8 (32·6 to 39·5)
	Laos	17 757 (16 038 to 19 746)	241 (218 to 268)	17·7 (−7·8 to 35·4)	29 003 (27 652 to 30 510)	523 (500 to 546)	53·6 (49·8 to 57·0)
	Malaysia	100 399 (90 368 to 111 622)	324 (292 to 358)	36·5 (32·2 to 41·5)	219 095 (209 068 to 229 686)	746 (715 to 781)	42·9 (40·1 to 45·4)
	Maldives	772 (699 to 859)	211 (192 to 233)	7·2 (3·6 to 10·5)	1638 (1558 to 1716)	505 (483 to 528)	20·5 (18·3 to 23·3)
	Mauritius	3375 (3049 to 3722)	269 (244 to 297)	43·8 (39·1 to 48·6)	8810 (8434 to 9178)	619 (592 to 646)	52·3 (48·8 to 55·6)
	Myanmar	133 998 (120 772 to 148 898)	250 (225 to 278)	43·3 (38·4 to 48·5)	309 036 (282 463 to 348 950)	598 (549 to 670)	72·3 (60·5 to 88·9)
	Philippines	285 035 (256 711 to 318 916)	275 (249 to 307)	29·7 (21·0 to 38·3)	525 214 (499 001 to 554 225)	589 (562 to 621)	42·4 (38·2 to 45·9)
	Sri Lanka	63 643 (57 818 to 70 087)	309 (281 to 340)	−23·4 (−49·2 to 0·2)	172 628 (158 213 to 195 277)	794 (727 to 897)	37·5 (29·7 to 48·6)
	Seychelles	306 (278 to 339)	312 (283 to 345)	37·7 (33·4 to 42·4)	694 (665 to 723)	692 (663 to 720)	45·6 (43·1 to 48·5)
	Thailand	244 221 (218 841 to 271 818)	352 (317 to 391)	28·2 (23·8 to 32·7)	652 995 (624 934 to 682 082)	812 (776 to 848)	35·6 (32·8 to 38·2)
	Timor-Leste	2689 (2426 to 2970)	235 (213 to 260)	−26·3 (−56·8 to 8·5)	5952 (5049 to 7666)	711 (593 to 930)	39·3 (28·2 to 49·8)
	Vietnam	275 305 (247 741 to 307 255)	291 (262 to 323)	50·4 (46·0 to 55·0)	605 688 (577 863 to 632 572)	644 (616 to 672)	59·2 (55·8 to 62·5)
**Oceania**		**31 414 (28 426 to 34 833)**	**282 (256 to 312)**	**29·1 (24·1 to 33·7)**	**52 480 (49 980 to 55 147)**	**565 (540 to 591)**	**39·8 (37·6 to 42·4)**
	American Samoa	227 (206 to 250)	290 (264 to 320)	13·2 (10·1 to 16·6)	444 (422 to 466)	644 (616 to 674)	17·6 (14·9 to 20·2)
	Federated States of Micronesia	273 (247 to 302)	267 (242 to 295)	28·0 (24·2 to 32·1)	464 (442 to 489)	545 (521 to 571)	31·4 (28·6 to 34·2)
	Fiji	2181 (1982 to 2409)	256 (234 to 282)	40·0 (35·7 to 44·4)	4599 (4402 to 4812)	538 (516 to 562)	43·5 (40·8 to 45·9)
	Guam	566 (514 to 624)	326 (296 to 359)	34·0 (29·6 to 38·6)	1273 (1219 to 1330)	726 (695 to 758)	34·7 (32·6 to 37·1)
	Kiribati	283 (255 to 316)	243 (219 to 271)	42·2 (38·0 to 46·9)	467 (444 to 491)	481 (459 to 503)	48·2 (45·6 to 51·2)
	Marshall Islands	197 (179 to 218)	263 (239 to 289)	31·4 (27·2 to 35·8)	322 (307 to 338)	528 (505 to 552)	31·1 (28·4 to 33·7)
	Northern Mariana Islands	405 (365 to 451)	307 (279 to 338)	10·4 (7·4 to 13·6)	758 (720 to 798)	702 (673 to 734)	11·9 (9·8 to 13·9)
	Papua New Guinea	22 356 (20 209 to 24 797)	288 (260 to 320)	27·0 (21·0 to 32·2)	35 154 (33 432 to 36 990)	566 (540 to 592)	41·4 (38·6 to 44·3)
	Samoa	518 (469 to 571)	261 (237 to 287)	27·3 (23·7 to 31·1)	944 (899 to 996)	583 (558 to 612)	36·5 (33·2 to 40·5)
	Solomon Islands	1593 (1443 to 1765)	269 (245 to 298)	33·4 (29·3 to 37·6)	2481 (2360 to 2611)	538 (515 to 563)	38·5 (36·2 to 41·0)
	Tonga	288 (261 to 320)	268 (244 to 296)	18·4 (14·2 to 22·7)	499 (477 to 523)	565 (542 to 589)	25·4 (23·1 to 27·8)
	Vanuatu	725 (655 to 803)	259 (235 to 287)	40·4 (35·7 to 45·1)	1206 (1148 to 1273)	539 (514 to 566)	46·7 (43·5 to 49·7)
**North Africa and Middle East**		**2 434 103 (1 986 710 to 3 189 781)**	**412 (340 to 528)**	**14·5 (−1·1 to 43·0)**	**3 966 247 (3 679 371 to 4 400 997)**	**782 (730 to 864)**	**1·3 (−0·8 to 3·7)**
	Afghanistan	207 438 (117 298 to 397 313)	564 (331 to 1 046)	63·3 (−0·4 to 166·9)	219 778 (163 909 to 318 642)	953 (670 to 1 458)	−10·5 (−22·8 to 11·3)
	Algeria	124 287 (111 642 to 137 587)	310 (278 to 343)	−7·5 (−10·0 to −4·8)	259 395 (246 483 to 272 456)	710 (677 to 743)	−1·6 (−3·6 to 0·7)
	Bahrain	4748 (4278 to 5231)	339 (305 to 374)	−8·4 (−11·6 to −5·1)	10 818 (10 262 to 11 427)	796 (759 to 835)	−2·1 (−4·8 to 0·8)
	Egypt	262 264 (236 586 to 292 139)	281 (254 to 312)	24·6 (21·1 to 28·6)	484 935 (461 177 to 509 082)	601 (574 to 628)	23·2 (20·3 to 26·4)
	Iran	302 610 (272 173 to 335 983)	372 (335 to 411)	−32·7 (−49·9 to −12·2)	701 593 (652 604 to 773 884)	921 (858 to 1013)	−7·8 (−12·8 to −3·5)
	Iraq	267 248 (165 709 to 466 109)	633 (407 to 1 097)	67·2 (8·7 to 181·8)	314 391 (253 102 to 440 165)	1134 (908 to 1581)	−4·2 (−12·8 to 5·1)
	Jordan	27 961 (22 479 to 37 776)	343 (278 to 457)	4·3 (−12·2 to 41·3)	40 959 (38 745 to 43 436)	646 (616 to 679)	−11·4 (−13·8 to −8·5)
	Kuwait	14 802 (13 329 to 16 386)	376 (339 to 415)	−32·3 (−53·5 to −10·7)	33 516 (31 700 to 35 329)	902 (861 to 943)	−6·4 (−9·7 to −4·0)
	Lebanon	18 765 (16 061 to 22 592)	329 (282 to 395)	−44·6 (−62·5 to −26·6)	63 264 (49 117 to 92 756)	1099 (849 to 1623)	−21·9 (−30·4 to −12·0)
	Libya	26 136 (19 639 to 38 381)	420 (318 to 617)	29·1 (0·5 to 90·0)	47 418 (41 783 to 57 143)	814 (723 to 970)	6·8 (−3·4 to 25·2)
	Morocco	95 064 (85 330 to 105 706)	284 (255 to 316)	0·5 (−2·4 to 3·4)	212 653 (203 045 to 224 043)	647 (618 to 680)	5·9 (3·8 to 8·5)
	Oman	20 498 (18 259 to 22 719)	427 (383 to 475)	−5·5 (−9·0 to −2·0)	41 920 (39 721 to 43 961)	991 (945 to 1036)	−1·1 (−3·0 to 0·9)
	Palestine	16 165 (14 415 to 18 200)	284 (256 to 318)	−2·8 (−18·9 to 9·5)	25 590 (22 870 to 30 171)	714 (629 to 875)	−1·2 (−10·3 to 7·7)
	Qatar	11 778 (10 504 to 13 139)	484 (436 to 535)	−2·3 (−5·5 to 0·8)	24 779 (23 437 to 26 188)	1155 (1102 to 1210)	0·0 (−2·2 to 1·9)
	Saudi Arabia	119 832 (107 918 to 132 057)	380 (341 to 419)	−13·5 (−15·3 to −11·5)	243 943 (233 302 to 255 668)	855 (821 to 892)	−11·1 (−12·5 to −10·0)
	Sudan	126 030 (109 808 to 146 687)	306 (268 to 352)	6·7 (−0·6 to 14·5)	199 309 (187 481 to 215 246)	639 (605 to 686)	12·9 (10·4 to 15·8)
	Syria	262 602 (93 725 to 553 607)	1322 (481 to 2 779)	424·8 (90·8 to 1 029·7)	149 597 (109 414 to 218 593)	917 (696 to 1288)	60·3 (26·7 to 117·7)
	Tunisia	34 436 (30 908 to 38 256)	314 (281 to 349)	4·4 (1·4 to 8·2)	80 306 (76 207 to 84 356)	699 (664 to 734)	7·1 (4·2 to 10·7)
	Turkey	248 553 (222 612 to 277 292)	316 (283 to 352)	−15·7 (−19·3 to −10·7)	557 595 (531 362 to 584 590)	708 (676 to 742)	−13·2 (−16·3 to −10·2)
	United Arab Emirates	46 220 (41 326 to 51 437)	464 (417 to 513)	−6·8 (−9·2 to −4·4)	102 902 (97 674 to 108 666)	1074 (1028 to 1125)	−4·1 (−6·3 to −2·1)
	Yemen	194 241 (130 687 to 327 977)	626 (427 to 1051)	99·0 (35·8 to 234·4)	147 165 (134 214 to 168 397)	708 (659 to 783)	7·8 (3·0 to 15·6)
**South Asia**		**7 039 830 (6 292 303 to 7 812 364)**	**439 (393 to 488)**	**4·4 (2·4 to 6·6)**	**12 366 812 (11 871 688 to 12 866 592)**	**828 (794 to 860)**	**16·7 (15·7 to 17·8)**
	Bangladesh	540 467 (485 923 to 599 958)	343 (309 to 383)	12·9 (9·6 to 16·3)	980 717 (935 604 to 1 031 234)	698 (666 to 732)	29·5 (26·4 to 33·2)
	Bhutan	3214 (2872 to 3589)	426 (379 to 477)	2·1 (−0·5 to 4·7)	5489 (5233 to 5756)	811 (777 to 847)	7·6 (5·2 to 10·0)
	India	5 641 697 (5 039 029 to 6 262 015)	455 (406 to 505)	2·3 (0·1 to 4·6)	9 965 355 (9 558 481 to 10 358 885)	846 (811 to 879)	14·7 (13·8 to 15·7)
	Nepal	108 610 (96 933 to 121 246)	382 (340 to 427)	3·9 (0·9 to 7·1)	181 820 (173 484 to 191 290)	751 (717 to 788)	20·3 (17·3 to 23·7)
	Pakistan	745 843 (669 513 to 830 822)	401 (361 to 446)	19·9 (16·5 to 23·2)	1 233 430 (1 174 231 to 1 291 274)	803 (769 to 837)	26·5 (23·7 to 30·0)
**Sub-Saharan Africa**		**2 956 908 (2 659 347 to 3 286 997)**	**326 (293 to 363)**	**−11·8 (−20·3 to −6·7)**	**4 182 169 (3 987 073 to 4 395 220)**	**621 (594 to 649)**	**0·7 (−0·3 to 1·8)**
**Southern sub-Saharan Africa**		**251 795 (227 351 to 279 669)**	**332 (300 to 368)**	**−14·4 (−18·0 to −12·0)**	**420 050 (401 203 to 441 118)**	**640 (614 to 670)**	**−15·2 (−16·2 to −14·3)**
	Botswana	7864 (7032 to 8785)	351 (316 to 391)	17·1 (14·2 to 20·1)	12 941 (12 262 to 13 600)	675 (643 to 706)	16·0 (14·2 to 18·0)
	eSwatini	4735 (4232 to 5305)	365 (326 to 407)	17·0 (13·3 to 20·8)	6370 (6051 to 6696)	646 (617 to 675)	8·3 (6·5 to 10·2)
	Lesotho	7016 (6310 to 7810)	336 (304 to 372)	25·7 (22·4 to 29·2)	9080 (8651 to 9546)	529 (507 to 555)	9·0 (7·0 to 11·2)
	Namibia	7400 (6646 to 8200)	296 (266 to 326)	−0·2 (−2·8 to 2·4)	11 413 (10 830 to 11 979)	578 (551 to 603)	2·1 (0·7 to 3·6)
	South Africa	185 015 (167 062 to 205 569)	352 (318 to 390)	−18·8 (−22·9 to −15·9)	327 583 (313 064 to 344 174)	680 (652 to 712)	−19·3 (−20·3 to −18·3)
	Zimbabwe	39 766 (35 598 to 44 047)	271 (245 to 300)	2·9 (0·9 to 5·0)	52 663 (50 084 to 55 611)	471 (452 to 492)	0·7 (−1·1 to 2·5)
**Western sub-Saharan Africa**		**1 158 340 (1 040 158 to 1 293 825)**	**316 (284 to 353)**	**−2·6 (−4·9 to −0·7)**	**1 614 512 (1 537 783 to 1 696 546)**	**597 (572 to 622)**	**3·1 (1·9 to 4·4)**
	Benin	33 501 (30 129 to 37 297)	327 (293 to 364)	4·6 (1·7 to 7·6)	47 011 (44 631 to 49 362)	613 (585 to 638)	7·7 (5·2 to 10·1)
	Burkina Faso	53 214 (47 530 to 59 321)	312 (278 to 349)	−0·6 (−3·2 to 2·1)	69 252 (65 694 to 72 704)	567 (542 to 592)	6·3 (4·3 to 8·6)
	Cameroon	70 982 (63 788 to 79 440)	320 (286 to 359)	3·0 (0·0 to 6·2)	95 352 (90 299 to 100 578)	572 (546 to 598)	0·5 (−1·4 to 2·7)
	Cape Verde	1654 (1495 to 1838)	304 (275 to 337)	12·2 (9·7 to 14·8)	2810 (2673 to 2944)	626 (600 to 653)	16·0 (13·5 to 18·6)
	Chad	42 615 (38 197 to 47 636)	315 (282 to 351)	−7·6 (−22·9 to 2·7)	54 599 (51 499 to 58 150)	595 (564 to 631)	5·1 (2·0 to 7·7)
	Côte d'Ivoire	71 655 (64 027 to 80 338)	344 (307 to 385)	−5·3 (−7·9 to −2·6)	99 313 (93 967 to 104 562)	617 (589 to 644)	−3·0 (−5·1 to −0·9)
	The Gambia	5471 (4879 to 6116)	291 (261 to 325)	−9·8 (−11·7 to −8·0)	7266 (6889 to 7683)	552 (528 to 578)	−9·4 (−11·5 to −7·4)
	Ghana	84 274 (75 885 to 93 620)	329 (295 to 367)	12·5 (10·0 to 15·1)	125 387 (119 141 to 131 582)	614 (587 to 640)	15·9 (13·5 to 18·4)
	Guinea	35 050 (31 420 to 39 047)	297 (267 to 331)	−5·1 (−7·2 to −2·8)	48 739 (46 352 to 51 196)	539 (516 to 562)	−4·7 (−6·9 to −2·2)
	Guinea-Bissau	5715 (5146 to 6362)	328 (295 to 365)	−7·8 (−9·5 to −6·1)	7776 (7400 to 8173)	567 (544 to 591)	−4·7 (−6·7 to −2·7)
	Liberia	11 400 (10 217 to 12 762)	270 (242 to 302)	−61·6 (−77·5 to −41·0)	17 961 (16 439 to 20 466)	558 (512 to 634)	−4·3 (−11·0 to 4·7)
	Mali	49 132 (43 999 to 54 775)	292 (263 to 325)	−5·0 (−8·6 to −2·3)	64 365 (61 076 to 68 253)	557 (532 to 586)	6·6 (3·5 to 11·1)
	Mauritania	12 094 (10 836 to 13 462)	321 (288 to 358)	−11·3 (−22·4 to −3·5)	19 189 (18 276 to 20 120)	642 (614 to 670)	5·7 (3·6 to 7·9)
	Niger	52 541 (47 077 to 58 407)	281 (253 to 314)	−11·2 (−14·2 to −8·7)	66 915 (63 772 to 70 348)	521 (498 to 543)	−5·6 (−7·8 to −3·6)
	Nigeria	546 313 (488 793 to 609 609)	319 (286 to 358)	−0·6 (−3·3 to 2·6)	772 539 (731 537 to 814 574)	619 (591 to 648)	3·8 (1·7 to 6·0)
	São Tomé and Príncipe	670 (603 to 748)	360 (324 to 402)	5·9 (3·0 to 8·7)	945 (897 to 992)	705 (673 to 737)	6·3 (3·8 to 8·7)
	Senegal	44 145 (39 611 to 48 997)	314 (280 to 350)	−0·4 (−3·1 to 2·5)	61 131 (58 199 to 64 314)	586 (560 to 612)	2·4 (−0·1 to 4·6)
	Sierra Leone	17 879 (16 024 to 19 972)	299 (268 to 334)	−5·1 (−7·3 to −3·0)	25 965 (24 414 to 28 059)	573 (541 to 620)	0·9 (−3·5 to 8·5)
	Togo	20 024 (17 885 to 22 273)	296 (265 to 330)	−1·9 (−4·4 to 0·8)	27 967 (26 541 to 29 459)	536 (513 to 559)	−1·9 (−3·9 to 0·4)
**Eastern sub-Saharan Africa**		**1 181 878 (1 055 453 to 1 317 176)**	**337 (302 to 377)**	**−19·1 (−33·5 to −9·4)**	**1 649 534 (1 564 002 to 1 748 871)**	**640 (608 to 680)**	**5·3 (3·4 to 7·0)**
	Burundi	37 392 (33 413 to 41 982)	363 (324 to 408)	3·0 (0·5 to 5·6)	51 646 (48 379 to 56 131)	689 (646 to 748)	24·3 (18·8 to 34·5)
	Comoros	2436 (2192 to 2719)	339 (304 to 377)	−22·2 (−24·0 to −20·1)	3746 (3554 to 3940)	661 (632 to 690)	−16·8 (−18·6 to −14·8)
	Djibouti	3282 (2949 to 3671)	364 (326 to 408)	−9·1 (−16·4 to −4·4)	5163 (4902 to 5411)	708 (676 to 738)	−1·2 (−3·1 to 1·0)
	Eritrea	16 867 (15 110 to 18 875)	357 (319 to 400)	−1·4 (−4·3 to 1·4)	24 312 (22 780 to 26 223)	679 (640 to 728)	15·2 (10·5 to 22·6)
	Ethiopia	314 622 (281 760 to 350 817)	343 (307 to 382)	−43·4 (−62·2 to −25·4)	456 884 (430 726 to 485 886)	659 (624 to 703)	2·4 (−2·7 to 6·0)
	Kenya	148 509 (133 090 to 166 370)	349 (313 to 391)	8·8 (7·7 to 9·8)	216 411 (206 413 to 226 979)	669 (642 to 696)	12·9 (12·1 to 13·9)
	Madagascar	70 496 (63 093 to 78 880)	305 (272 to 342)	−5·6 (−8·3 to −2·8)	98 994 (93 993 to 104 236)	567 (540 to 593)	−2·6 (−4·8 to −0·3)
	Malawi	45 111 (40 282 to 50 765)	273 (244 to 307)	−13·1 (−15·3 to −10·8)	56 744 (53 648 to 60 217)	478 (455 to 502)	−9·8 (−12·3 to −7·7)
	Mozambique	86 051 (77 161 to 95 938)	333 (298 to 371)	1·5 (−11·2 to 9·5)	116 779 (109 146 to 126 995)	629 (584 to 697)	4·2 (−5·1 to 11·2)
	Rwanda	33 114 (29 559 to 37 120)	297 (265 to 334)	−35·1 (−50·5 to −24·7)	63 878 (52 403 to 85 437)	782 (633 to 1065)	27·0 (4·1 to 73·2)
	Somalia	38 718 (32 227 to 50 226)	402 (339 to 505)	−11·6 (−17·6 to −6·7)	45 828 (42 334 to 51 096)	645 (598 to 719)	1·2 (−2·3 to 6·6)
	South Sudan	46 491 (41 340 to 52 564)	383 (341 to 434)	−29·0 (−49·5 to −9·0)	63 707 (59 090 to 70 275)	723 (674 to 797)	1·1 (−3·0 to 7·3)
	Tanzania	166 283 (148 670 to 186 579)	332 (298 to 371)	−3·1 (−5·3 to −0·8)	225 251 (213 622 to 237 194)	615 (588 to 643)	1·7 (−0·5 to 4·0)
	Uganda	116 067 (103 532 to 129 901)	316 (283 to 354)	0·3 (−4·1 to 3·8)	149 404 (139 610 to 161 903)	609 (568 to 674)	9·2 (−0·6 to 16·7)
	Zambia	55 681 (49 801 to 62 201)	381 (341 to 424)	7·1 (4·3 to 10·0)	69 654 (66 142 to 73 339)	647 (619 to 675)	4·6 (2·6 to 6·8)
**Central sub-Saharan Africa**		**364 894 (327 646 to 405 768)**	**331 (296 to 369)**	**−7·4 (−11·7 to −4·5)**	**498 074 (473 461 to 526 322)**	**637 (607 to 673)**	**2·5 (0·8 to 4·7)**
	Angola	89 553 (80 374 to 99 729)	378 (338 to 424)	−12·8 (−27·2 to −3·2)	123 421 (115 993 to 132 673)	772 (726 to 841)	6·4 (1·5 to 10·2)
	Central African Republic	14 436 (12 954 to 16 183)	299 (268 to 335)	5·7 (1·6 to 11·2)	18 918 (17 855 to 20 081)	487 (464 to 513)	5·7 (2·6 to 9·9)
	Congo (Brazzaville)	15 810 (14 211 to 17 546)	356 (318 to 397)	−2·4 (−4·4 to −0·2)	23 976 (22 503 to 25 886)	711 (669 to 769)	15·3 (10·3 to 24·3)
	Democratic Republic of the Congo	235 694 (211 454 to 262 391)	315 (283 to 351)	−6·6 (−8·6 to −4·6)	316 583 (300 808 to 333 679)	599 (571 to 627)	−0·5 (−3·1 to 3·0)
	Equatorial Guinea	3144 (2808 to 3501)	402 (359 to 452)	16·1 (12·5 to 20·0)	5148 (4920 to 5371)	799 (765 to 831)	39·4 (36·4 to 42·4)
	Gabon	6257 (5600 to 6956)	370 (331 to 411)	−12·0 (−14·1 to −9·8)	10 028 (9590 to 10 472)	732 (701 to 761)	−4·0 (−5·7 to −2·4)

95% uncertainty intervals are in parentheses. SDI=Socio-demographic Index.

**Table 2:** Incidence and prevalence of spinal cord injury in 2016, and percentage change in age-standardised rates by location, 1990–2016

		**Incidence**			**Prevalence**		
		2016 counts	2016 age-standardised rates (per 100 000)	Percentage change in age-standardised rates, 1990–2016	2016 counts	2016 age-standardised rates (per 100 000)	Percentage change in age-standardised rates, 1990–2016
**Global**		**934 951 (780 963 to 1 155 187)**	**13 (11 to 16)**	**−3·6 (−7·4 to 4·0)**	**27 042 505 (24 976 608 to 30 148 230)**	**368 (340 to 409)**	**−0·2 (−2·1 to 2·7)**
High SDI		276 308 (216 293 to 355 713)	25 (20 to 31)	−3·6 (−6·9 to −0·5)	9 247 664 (8 524 049 to 9 989 539)	760 (698 to 827)	−1·5 (−3·1 to −0·1)
High-middle SDI		155 063 (128 957 to 184 025)	13 (11 to 16)	−13·7 (−18·7 to −10·0)	5 394 307 (4 994 153 to 6 042 158)	420 (389 to 469)	−4·8 (−6·4 to −3·3)
Middle SDI		176 312 (149 499 to 209 614)	8 (7 to 9)	5·8 (−0·9 to 11·7)	5 576 932 (5 244 937 to 5 941 725)	231 (217 to 246)	25·6 (23·4 to 28·0)
Low-middle SDI		242 480 (189 305 to 340 476)	12 (9 to 16)	17·9 (4·2 to 54·0)	5 141 936 (4 571 709 to 6 349 080)	260 (233 to 319)	22·6 (19·5 to 28·1)
Low SDI		89 536 (65 824 to 142 240)	12 (9 to 18)	−20·0 (−33·2 to −10·1)	1 795 869 (1 327 167 to 2 961 901)	304 (220 to 519)	14·2 (8·1 to 25·5)
**High income**		**287 206 (223 675 to 372 032)**	**25 (20 to 32)**	**−4·3 (−7·7 to −1·1)**	**9 699 029 (8 946 042 to 10 481 324)**	**776 (713 to 846)**	**−2·0 (−3·6 to −0·6)**
**High-income North America**		**101 259 (79 044 to 129 722)**	**26 (20 to 33)**	**2·9 (−3·2 to 9·0)**	**2 959 275 (2 725 268 to 3 165 768)**	**709 (650 to 761)**	**−6·5 (−9·9 to −2·6)**
	Canada	9654 (7533 to 12 401)	25 (20 to 31)	−2·5 (−7·3 to 1·9)	324 689 (298 545 to 349 527)	752 (685 to 811)	0·5 (−2·7 to 3·8)
	Greenland	15 (12 to 19)	31 (24 to 39)	−16·5 (−19·0 to −14·0)	388 (357 to 416)	704 (644 to 759)	−2·1 (−5·5 to 1·5)
	USA	91 556 (71 406 to 117 479)	26 (20 to 33)	3·7 (−2·7 to 10·5)	2 633 160 (2 427 190 to 2 818 818)	704 (646 to 756)	−7·3 (−11·1 to −3·0)
**Australasia**		**6612 (5191 to 8442)**	**23 (18 to 29)**	**−2·2 (−8·0 to 2·9)**	**240 093 (220 533 to 259 720)**	**745 (682 to 811)**	**3·3 (−0·3 to 6·9)**
	Australia	5556 (4366 to 7090)	23 (18 to 28)	−1·3 (−7·1 to 4·1)	201 658 (185 041 to 218 197)	742 (679 to 809)	3·9 (−0·1 to 7·9)
	New Zealand	1057 (830 to 1351)	23 (18 to 29)	−6·4 (−12·3 to −0·9)	38 436 (35 415 to 41 681)	759 (695 to 828)	0·3 (−3·5 to 5·4)
**High-income Asia Pacific**		**51 251 (40 229 to 65 487)**	**25 (20 to 32)**	**−9·6 (−13·6 to −5·7)**	**1 831 823 (1 686 204 to 1 996 895)**	**821 (747 to 907)**	**1·1 (−1·3 to 3·9)**
	Brunei	124 (98 to 154)	32 (25 to 40)	−13·8 (−17·7 to −9·9)	4018 (3641 to 4463)	922 (840 to 1021)	−6·9 (−9·8 to −3·9)
	Japan	36 218 (28 255 to 46 493)	25 (20 to 31)	−5·1 (−10·0 to −1·0)	1 306 337 (1 202 409 to 1 424 185)	824 (749 to 913)	5·0 (2·1 to 8·8)
	Singapore	1000 (786 to 1269)	26 (21 to 33)	2·0 (−2·5 to 6·5)	39 555 (36 218 to 43 598)	875 (797 to 969)	15·6 (10·5 to 21·3)
	South Korea	13 909 (10 890 to 17 539)	27 (21 to 34)	−17·2 (−21·3 to −12·3)	481 913 (440 648 to 527 192)	811 (738 to 894)	−8·1 (−11·6 to −4·4)
**Western Europe**		**115 958 (88 458 to 151 615)**	**26 (20 to 33)**	**−6·8 (−10·8 to −3·5)**	**4 297 097 (3 965 806 to 4 706 288)**	**854 (780 to 945)**	**0·3 (−1·6 to 2·5)**
	Andorra	21 (16 to 28)	26 (20 to 33)	4·0 (1·4 to 6·9)	834 (763 to 915)	886 (805 to 985)	7·0 (3·3 to 10·8)
	Austria	2614 (1982 to 3459)	29 (22 to 37)	−14·8 (−19·0 to −10·3)	97 310 (88 649 to 107 961)	937 (853 to 1051)	−5·7 (−8·9 to −1·3)
	Belgium	3784 (2863 to 5077)	30 (23 to 39)	3·7 (−3·3 to 10·2)	121 148 (110 972 to 133 190)	908 (827 to 1008)	3·7 (0·0 to 7·8)
	Cyprus	239 (186 to 303)	27 (21 to 34)	−2·4 (−7·0 to 1·8)	9060 (8244 to 9999)	887 (803 to 984)	5·0 (1·6 to 8·7)
	Denmark	1582 (1199 to 2081)	27 (21 to 35)	−10·9 (−15·6 to −6·0)	57 680 (52 720 to 63 666)	876 (793 to 979)	5·1 (0·6 to 9·2)
	Finland	1963 (1476 to 2638)	32 (25 to 42)	0·5 (−4·3 to 5·4)	64 375 (58 841 to 70 664)	977 (884 to 1084)	11·3 (7·5 to 15·7)
	France	19 918 (14 899 to 26 506)	27 (21 to 35)	−12·4 (−17·1 to −7·7)	643 671 (592 262 to 702 838)	855 (781 to 943)	−5·5 (−8·8 to −1·7)
	Germany	22 047 (16 728 to 28 901)	26 (20 to 33)	−5·6 (−10·7 to −0·9)	837 659 (768 281 to 921 948)	842 (765 to 939)	3·0 (−1·0 to 7·3)
	Greece	2627 (2036 to 3381)	25 (20 to 32)	−9·7 (−13·7 to −5·8)	111 122 (102 610 to 121 509)	860 (787 to 949)	−3·7 (−7·0 to −0·5)
	Iceland	84 (65 to 108)	25 (20 to 32)	−1·2 (−5·6 to 3·2)	3138 (2868 to 3465)	864 (786 to 959)	7·6 (3·3 to 12·4)
	Ireland	1250 (964 to 1625)	28 (21 to 35)	5·4 (−0·8 to 10·7)	48 263 (43 550 to 53 544)	961 (862 to 1073)	13·3 (8·7 to 18·4)
	Israel	1969 (1532 to 2513)	24 (18 to 30)	0·2 (−9·9 to 6·3)	73 637 (63 187 to 94 545)	913 (782 to 1179)	18·9 (10·6 to 32·3)
	Italy	16 889 (13 033 to 21 903)	27 (21 to 34)	−6·0 (−9·9 to −1·8)	657 779 (602 165 to 725 620)	893 (810 to 999)	0·9 (−2·9 to 4·8)
	Luxembourg	158 (121 to 206)	27 (20 to 34)	−21·0 (−25·3 to −16·9)	5725 (5240 to 6266)	854 (778 to 941)	−13·6 (−16·8 to −10·1)
	Malta	109 (84 to 141)	26 (21 to 34)	−7·8 (−11·0 to −4·5)	4474 (4088 to 4920)	905 (820 to 1003)	−0·1 (−4·0 to 3·3)
	Netherlands	4019 (3104 to 5168)	23 (18 to 29)	3·0 (−2·5 to 9·1)	152 583 (141 164 to 164 905)	764 (702 to 833)	8·7 (4·1 to 13·7)
	Norway	1498 (1139 to 1981)	27 (21 to 35)	−0·5 (−4·8 to 4·2)	52 434 (47 800 to 58 063)	876 (795 to 982)	9·7 (5·5 to 14·3)
	Portugal	2425 (1888 to 3137)	22 (18 to 28)	−23·3 (−28·1 to −18·8)	92 650 (85 596 to 100 266)	730 (671 to 797)	−18·2 (−22·0 to −14·4)
	Spain	11 337 (8741 to 14 624)	24 (19 to 31)	−6·4 (−11·9 to −1·0)	464 843 (427 508 to 508 351)	841 (768 to 931)	1·0 (−2·9 to 5·8)
	Sweden	2719 (2083 to 3556)	26 (20 to 34)	2·9 (−1·8 to 7·0)	101 699 (92 140 to 113 327)	903 (811 to 1017)	7·2 (2·8 to 11·4)
	Switzerland	2379 (1793 to 3115)	25 (20 to 33)	−28·3 (−34·0 to −23·3)	79 465 (73 587 to 85 786)	780 (718 to 847)	−23·9 (−28·0 to −19·9)
	UK	16 215 (12 431 to 21 182)	25 (19 to 32)	−0·4 (−4·5 to 2·8)	613 245 (561 180 to 677 232)	833 (754 to 929)	2·5 (0·2 to 4·8)
**Southern Latin America**		**12 125 (9601 to 15 163)**	**18 (15 to 23)**	**9·6 (6·1 to 12·6)**	**370 741 (343 020 to 399 059)**	**548 (507 to 591)**	**19·3 (15·6 to 23·5)**
	Argentina	8086 (6429 to 10 010)	18 (15 to 23)	10·3 (5·5 to 14·5)	246 246 (227 798 to 265 490)	556 (514 to 600)	20·5 (15·8 to 26·0)
	Chile	3362 (2662 to 4286)	18 (14 to 23)	4·9 (1·5 to 8·1)	104 731 (96 919 to 112 518)	534 (493 to 575)	15·2 (11·2 to 19·2)
	Uruguay	677 (533 to 851)	19 (15 to 23)	14·6 (11·1 to 18·3)	19 745 (18 265 to 21 341)	534 (492 to 578)	20·9 (16·5 to 26·4)
**Central Europe, eastern Europe, and central Asia**		**77 852 (63 320 to 94 211)**	**18 (15 to 22)**	**−5·2 (−7·3 to −3·1)**	**2 371 936 (2 210 605 to 2 553 070)**	**513 (477 to 554)**	**2·5 (−0·1 to 7·0)**
**Eastern Europe**		**41 674 (34 038 to 50 176)**	**19 (16 to 23)**	**−4·2 (−7·1 to −1·4)**	**1 222 360 (1 141 216 to 1 306 976)**	**510 (474 to 547)**	**−1·0 (−3·9 to 2·7)**
	Belarus	2111 (1719 to 2578)	22 (18 to 26)	14·2 (10·3 to 18·3)	62 524 (58 133 to 66 705)	568 (527 to 609)	13·8 (10·1 to 17·6)
	Estonia	249 (201 to 304)	19 (15 to 23)	−18·0 (−22·1 to −13·6)	8391 (7794 to 9006)	549 (507 to 592)	−4·0 (−7·6 to −0·4)
	Latvia	383 (310 to 470)	19 (15 to 23)	−20·4 (−24·1 to −16·4)	11 905 (11 114 to 12 653)	516 (480 to 552)	−9·5 (−12·1 to −6·6)
	Lithuania	653 (527 to 798)	21 (18 to 26)	−3·8 (−7·6 to 0·4)	19 428 (18 014 to 20 800)	570 (526 to 614)	1·8 (−2·1 to 5·5)
	Moldova	652 (537 to 776)	16 (13 to 19)	−15·7 (−19·2 to −11·8)	20 939 (19 342 to 22 699)	458 (422 to 497)	−5·0 (−9·0 to 0·3)
	Russia	29 681 (24 253 to 35 758)	20 (16 to 24)	−4·1 (−7·8 to −0·7)	847 799 (789 586 to 907 826)	514 (478 to 552)	−1·3 (−4·7 to 3·1)
	Ukraine	7945 (6532 to 9573)	18 (15 to 21)	−6·3 (−9·7 to −2·8)	251 374 (235 107 to 268 367)	484 (451 to 519)	−2·6 (−6·3 to 1·7)
**Central Europe**		**24 512 (19 365 to 30 562)**	**20 (16 to 25)**	**−3·3 (−6·4 to −0·6)**	**812 242 (750 130 to 881 919)**	**597 (549 to 653)**	**12·1 (8·8 to 17·9)**
	Albania	463 (369 to 567)	16 (13 to 20)	10·4 (5·3 to 15·3)	17 030 (15 238 to 19 542)	537 (480 to 622)	20·0 (11·8 to 36·8)
	Bosnia and Herzegovina	621 (497 to 772)	17 (14 to 21)	32·5 (27·6 to 38·0)	33 351 (25 724 to 49 144)	739 (568 to 1097)	91·8 (50·9 to 184·2)
	Bulgaria	1331 (1055 to 1664)	19 (15 to 23)	−6·1 (−10·4 to −1·8)	47 216 (43 288 to 50 931)	553 (503 to 598)	0·0 (−3·7 to 3·5)
	Croatia	874 (687 to 1101)	18 (15 to 22)	−3·7 (−9·7 to 2·2)	27 754 (24 164 to 34 570)	545 (472 to 684)	13·6 (−0·4 to 46·6)
	Czech Republic	2691 (2131 to 3348)	24 (20 to 30)	−4·5 (−10·0 to 1·1)	91 860 (84 086 to 99 449)	728 (662 to 791)	19·1 (14·6 to 24·6)
	Hungary	2256 (1759 to 2863)	21 (16 to 26)	−17·4 (−22·5 to −12·8)	64 731 (59 089 to 69 980)	554 (504 to 603)	4·1 (−0·6 to 8·5)
	Macedonia	350 (276 to 430)	17 (14 to 21)	17·5 (12·9 to 21·9)	12 171 (11 089 to 13 251)	518 (470 to 567)	21·7 (16·7 to 27·2)
	Montenegro	117 (94 to 144)	19 (15 to 23)	8·0 (4·8 to 11·4)	3952 (3633 to 4262)	564 (515 to 612)	11·6 (8·2 to 15·7)
	Poland	8501 (6733 to 10 627)	21 (17 to 26)	0·9 (−3·6 to 5·0)	267 715 (247 729 to 289 959)	589 (543 to 641)	15·1 (11·2 to 19·5)
	Romania	3972 (3142 to 4942)	20 (16 to 25)	−10·3 (−15·0 to −5·9)	129 774 (119 600 to 139 154)	571 (525 to 617)	−5·5 (−9·3 to −2·0)
	Serbia	1542 (1220 to 1916)	18 (14 to 22)	13·7 (10·3 to 17·4)	60 445 (52 192 to 75 281)	604 (518 to 761)	36·8 (20·9 to 72·7)
	Slovakia	1189 (937 to 1487)	21 (17 to 26)	−9·4 (−13·1 to −5·5)	37 606 (34 645 to 40 508)	588 (538 to 637)	5·7 (2·1 to 9·4)
	Slovenia	605 (465 to 778)	26 (20 to 33)	−0·8 (−6·9 to 7·3)	18 638 (17 160 to 19 922)	732 (670 to 785)	14·5 (10·3 to 18·8)
**Central Asia**		**11 666 (9682 to 13 817)**	**13 (11 to 16)**	**−0·9 (−3·1 to 1·4)**	**337 334 (311 213 to 368 474)**	**391 (361 to 425)**	**5·4 (1·8 to 12·0)**
	Armenia	381 (312 to 456)	13 (11 to 15)	−12·5 (−16·5 to −8·6)	17 219 (13 820 to 22 659)	518 (418 to 678)	−15·9 (−23·6 to −7·2)
	Azerbaijan	1233 (1023 to 1470)	13 (10 to 15)	−3·0 (−6·3 to 0·4)	41 913 (37 773 to 48 658)	407 (368 to 469)	14·1 (5·8 to 30·9)
	Georgia	514 (422 to 615)	13 (11 to 15)	−5·6 (−9·2 to −2·0)	17 389 (15 704 to 20 381)	395 (355 to 464)	4·3 (−4·5 to 22·4)
	Kazakhstan	2777 (2306 to 3283)	15 (13 to 18)	5·8 (1·7 to 10·0)	74 482 (69 375 to 79 913)	419 (391 to 450)	7·5 (4·5 to 10·9)
	Kyrgyzstan	751 (625 to 891)	12 (10 to 15)	−14·3 (−17·8 to −10·9)	19 731 (18 264 to 21 228)	354 (329 to 379)	−6·7 (−11·4 to −2·0)
	Mongolia	496 (410 to 592)	16 (13 to 19)	34·1 (19·4 to 44·4)	12 043 (11 144 to 12 943)	405 (377 to 434)	39·1 (34·1 to 44·5)
	Tajikistan	1012 (836 to 1206)	11 (9 to 14)	−11·5 (−15·0 to −8·1)	31 335 (25 925 to 43 192)	416 (346 to 568)	20·8 (2·2 to 63·7)
	Turkmenistan	671 (558 to 792)	12 (10 to 14)	0·2 (−3·4 to 3·8)	18 298 (16 869 to 19 720)	344 (319 to 369)	8·0 (4·7 to 11·4)
	Uzbekistan	3831 (3173 to 4538)	12 (10 to 15)	3·7 (0·3 to 6·9)	104 924 (97 144 to 113 211)	355 (330 to 383)	8·0 (5·2 to 11·0)
**Latin America and Caribbean**		**44 612 (36 971 to 53 003)**	**8 (7 to 10)**	**−4·4 (−8·6 to −1·1)**	**1 257 730 (1 167 571 to 1 358 261)**	**222 (206 to 239)**	**1·2 (−1·1 to 4·3)**
**Central Latin America**		**16 957 (14 048 to 20 273)**	**7 (6 to 8)**	**−14·8 (−19·8 to −11·6)**	**481 048 (439 582 to 529 653)**	**197 (181 to 216)**	**−5·2 (−7·4 to −2·9)**
	Colombia	3224 (2631 to 3876)	7 (6 to 8)	−12·6 (−19·0 to −7·7)	102 906 (93 911 to 113 136)	210 (192 to 230)	4·6 (0·4 to 10·4)
	Costa Rica	348 (285 to 421)	7 (6 to 9)	16·2 (12·2 to 20·2)	10 475 (9631 to 11 355)	209 (192 to 226)	25·0 (21·9 to 28·1)
	El Salvador	437 (358 to 528)	7 (6 to 9)	−22·1 (−49·3 to −0·6)	16 404 (11 867 to 27 532)	282 (202 to 481)	−10·6 (−22·4 to 4·8)
	Guatemala	1134 (937 to 1355)	7 (6 to 9)	−3·5 (−30·6 to 12·3)	28 342 (24 201 to 36 840)	209 (177 to 279)	6·4 (−6·7 to 17·6)
	Honduras	511 (420 to 615)	7 (6 to 8)	29·8 (23·4 to 36·7)	14 181 (12 288 to 16 888)	193 (168 to 227)	48·5 (33·4 to 73·7)
	Mexico	8221 (6810 to 9858)	7 (6 to 8)	−23·8 (−26·6 to −20·9)	218 025 (202 252 to 234 802)	177 (165 to 190)	−17·0 (−19·5 to −14·2)
	Nicaragua	374 (309 to 449)	6 (5 to 8)	0·9 (−4·9 to 5·7)	14 630 (10 612 to 23 865)	260 (190 to 421)	−11·3 (−23·4 to 3·2)
	Panama	285 (234 to 345)	7 (6 to 9)	14·9 (10·7 to 18·7)	8224 (7588 to 8872)	212 (196 to 228)	21·8 (18·5 to 24·9)
	Venezuela	2424 (1985 to 2928)	8 (7 to 10)	11·0 (6·5 to 16·1)	67 861 (62 472 to 73 699)	223 (207 to 242)	20·8 (16·5 to 27·3)
**Andean Latin America**		**4900 (4107 to 5740)**	**8 (7 to 10)**	**−2·1 (−12·2 to 5·5)**	**134 761 (124 709 to 147 122)**	**241 (224 to 262)**	**10·6 (7·7 to 14·3)**
	Bolivia	858 (719 to 1002)	8 (7 to 9)	−9·7 (−12·8 to −6·6)	21 669 (19 934 to 23 636)	218 (202 to 236)	6·5 (3·8 to 9·4)
	Ecuador	1553 (1249 to 1947)	10 (8 to 12)	23·9 (8·7 to 53·4)	36 335 (33 747 to 39 419)	236 (220 to 255)	11·6 (8·2 to 14·4)
	Peru	2489 (2075 to 2913)	8 (7 to 9)	−11·5 (−25·3 to −1·5)	76 757 (70 427 to 84 881)	251 (231 to 277)	11·3 (7·1 to 16·6)
**Caribbean**		**3748 (3111 to 4449)**	**8 (7 to 10)**	**22·1 (16·4 to 30·7)**	**120 881 (99 280 to 156 635)**	**263 (216 to 341)**	**49·7 (24·2 to 94·0)**
	Antigua and Barbuda	7 (6 to 8)	8 (6 to 9)	11·6 (8·5 to 15·0)	220 (203 to 237)	232 (215 to 250)	15·6 (12·0 to 19·6)
	The Bahamas	30 (25 to 36)	8 (7 to 9)	6·6 (3·2 to 10·0)	906 (842 to 972)	220 (204 to 236)	15·7 (12·2 to 19·3)
	Barbados	20 (16 to 23)	7 (6 to 8)	16·7 (14·1 to 19·7)	662 (616 to 709)	212 (197 to 228)	20·4 (16·9 to 25·3)
	Belize	28 (24 to 34)	8 (7 to 9)	17·2 (−0·7 to 27·1)	711 (657 to 770)	212 (198 to 228)	23·6 (19·2 to 29·8)
	Bermuda	5 (4 to 6)	8 (6 to 9)	−2·5 (−5·7 to 0·9)	174 (161 to 186)	241 (223 to 258)	15·7 (12·3 to 19·4)
	Cuba	1054 (853 to 1310)	9 (7 to 10)	15·2 (9·3 to 21·0)	31 067 (28 803 to 33 423)	236 (218 to 255)	17·8 (13·7 to 23·3)
	Dominica	5 (4 to 6)	7 (6 to 8)	27·6 (24·2 to 31·1)	158 (145 to 170)	208 (192 to 224)	31·1 (26·0 to 38·3)
	Dominican Republic	770 (636 to 920)	7 (6 to 9)	26·2 (22·2 to 30·4)	22 083 (20 428 to 23 753)	220 (204 to 236)	33·8 (29·5 to 40·6)
	Grenada	8 (7 to 10)	8 (7 to 10)	24·6 (20·8 to 28·5)	230 (212 to 248)	220 (204 to 237)	24·9 (20·9 to 29·5)
	Guyana	59 (49 to 70)	8 (7 to 10)	9·5 (5·7 to 13·4)	1433 (1336 to 1527)	191 (179 to 203)	16·6 (13·2 to 19·9)
	Haiti	938 (747 to 1180)	9 (7 to 11)	31·2 (10·5 to 65·2)	37 949 (18 897 to 72 460)	381 (188 to 718)	177·3 (38·9 to 419·8)
	Jamaica	194 (161 to 230)	7 (6 to 8)	31·0 (27·0 to 35·2)	5733 (5309 to 6147)	202 (187 to 216)	30·1 (26·0 to 35·4)
	Puerto Rico	319 (260 to 386)	8 (7 to 10)	22·4 (17·3 to 27·8)	9695 (9034 to 10 384)	239 (222 to 256)	28·9 (25·2 to 33·2)
	Saint Lucia	13 (11 to 15)	7 (6 to 9)	16·6 (13·3 to 19·8)	406 (378 to 436)	213 (198 to 228)	24·3 (21·1 to 28·1)
	Saint Vincent and the Grenadines	9 (7 to 10)	8 (7 to 9)	20·4 (15·8 to 25·1)	242 (224 to 259)	216 (201 to 231)	22·0 (18·6 to 27·7)
	Suriname	41 (35 to 49)	8 (7 to 9)	20·2 (16·6 to 24·3)	1180 (1092 to 1273)	216 (201 to 233)	21·8 (15·8 to 26·5)
	Trinidad and Tobago	98 (81 to 118)	8 (6 to 9)	12·9 (−1·8 to 21·8)	3186 (2963 to 3403)	219 (203 to 234)	31·9 (28·2 to 37·7)
	Virgin Islands	9 (7 to 10)	8 (6 to 9)	13·3 (10·2 to 16·7)	254 (236 to 273)	217 (200 to 233)	16·7 (13·3 to 20·7)
**Tropical Latin America**		**19 006 (15 547 to 22 905)**	**9 (7 to 11)**	**0·4 (−4·0 to 5·0)**	**521 040 (484 365 to 556 898)**	**235 (218 to 251)**	**−2·4 (−4·9 to −0·0)**
	Brazil	18 503 (15 141 to 22 303)	9 (7 to 11)	−0·1 (−4·6 to 4·5)	507 588 (472 136 to 542 766)	235 (219 to 252)	−2·9 (−5·3 to −0·4)
	Paraguay	503 (413 to 606)	8 (6 to 9)	28·4 (24·0 to 33·4)	13 452 (12 233 to 14 741)	216 (198 to 235)	20·0 (15·6 to 24·0)
**Southeast Asia, east Asia, and Oceania**		**147 786 (123 214 to 177 266)**	**7 (6 to 9)**	**8·9 (−0·1 to 17·5)**	**5 355 950 (5 008 161 to 5 755 826)**	**234 (219 to 251)**	**32·3 (28·6 to 36·2)**
**East Asia**		**101 644 (84 599 to 122 719)**	**7 (6 to 9)**	**10·0 (0·2 to 18·6)**	**3 851 775 (3 621 176 to 4 082 414)**	**236 (222 to 250)**	**30·7 (27·4 to 34·0)**
	China	98 226 (81 769 to 118 651)	7 (6 to 9)	9·3 (−0·5 to 18·1)	3 739 610 (3 515 973 to 3 963 481)	237 (223 to 251)	30·6 (27·3 to 33·9)
	North Korea	1767 (1466 to 2124)	7 (5 to 8)	44·4 (33·1 to 59·7)	49 176 (46 203 to 52 190)	179 (168 to 190)	35·0 (31·8 to 38·6)
	Taiwan (province of China)	1650 (1368 to 1999)	7 (6 to 9)	21·6 (14·7 to 29·9)	62 989 (59 405 to 66 948)	227 (213 to 242)	29·3 (25·6 to 33·7)
**Southeast Asia**		**45 349 (38 407 to 54 280)**	**7 (6 to 8)**	**6·4 (−11·2 to 18·2)**	**1 486 699 (1 324 441 to 1 766 643)**	**228 (204 to 271)**	**37·3 (26·7 to 47·9)**
	Cambodia	1020 (860 to 1206)	7 (6 to 8)	−13·9 (−47·8 to 17·1)	49 783 (29 731 to 102 600)	377 (212 to 820)	−15·9 (−29·5 to 17·0)
	Indonesia	16 383 (13 827 to 19 498)	7 (6 to 8)	14·1 (8·9 to 19·3)	525 421 (479 338 to 584 463)	205 (188 to 229)	33·2 (21·5 to 44·5)
	Laos	444 (377 to 528)	6 (5 to 7)	−26·8 (−60·8 to 11·5)	10 096 (9319 to 10 871)	164 (153 to 177)	48·7 (42·8 to 53·3)
	Malaysia	2304 (1920 to 2798)	8 (6 to 9)	23·2 (18·0 to 29·5)	70 211 (66 032 to 74 696)	229 (216 to 243)	33·2 (30·6 to 35·7)
	Maldives	20 (17 to 23)	6 (5 to 6)	−0·8 (−4·6 to 3·1)	632 (585 to 678)	184 (171 to 196)	30·9 (27·0 to 35·4)
	Mauritius	81 (68 to 96)	7 (6 to 8)	30·3 (25·0 to 36·4)	2866 (2698 to 3052)	206 (194 to 220)	41·9 (38·7 to 46·3)
	Myanmar	3367 (2848 to 3916)	6 (5 to 7)	25·6 (14·7 to 34·3)	137 785 (109 122 to 183 939)	256 (203 to 343)	69·4 (31·5 to 124·7)
	Philippines	7921 (6314 to 10 612)	8 (6 to 10)	7·5 (−10·6 to 24·8)	204 930 (178 886 to 256 102)	219 (190 to 279)	28·6 (12·3 to 39·8)
	Sri Lanka	1593 (1350 to 1885)	8 (7 to 9)	−63·7 (−84·1 to −23·6)	94 402 (61 940 to 170 102)	435 (286 to 783)	93·5 (48·8 to 161·0)
	Seychelles	8 (6 to 9)	8 (7 to 9)	24·9 (20·1 to 31·0)	225 (211 to 240)	223 (209 to 237)	35·5 (28·2 to 40·3)
	Thailand	5507 (4550 to 6744)	8 (7 to 10)	16·5 (11·1 to 22·3)	186 063 (175 707 to 197 225)	239 (226 to 254)	29·4 (26·3 to 33·2)
	Timor-Leste	69 (59 to 81)	6 (5 to 7)	−70·0 (−88·0 to −25·0)	5688 (2514 to 13 572)	677 (280 to 1658)	41·3 (21·3 to 65·7)
	Vietnam	6571 (5510 to 7844)	7 (6 to 8)	36·7 (30·7 to 43·2)	196 504 (184 525 to 208 822)	204 (192 to 217)	52·0 (48·1 to 55·7)
**Oceania**		**793 (672 to 941)**	**7 (6 to 9)**	**12·5 (−1·5 to 21·8)**	**17 477 (16 227 to 18 776)**	**172 (160 to 184)**	**39·6 (35·0 to 46·2)**
	American Samoa	6 (5 to 7)	8 (7 to 9)	6·2 (2·5 to 10·0)	172 (159 to 186)	229 (214 to 247)	19·9 (14·8 to 28·4)
	Federated States of Micronesia	7 (6 to 8)	7 (6 to 8)	19·2 (15·2 to 24·0)	161 (150 to 172)	170 (160 to 181)	27·6 (23·6 to 32·7)
	Fiji	56 (47 to 66)	7 (6 to 8)	29·3 (24·6 to 35·0)	1533 (1431 to 1641)	174 (163 to 186)	36·8 (34·1 to 39·5)
	Guam	14 (12 to 17)	8 (7 to 10)	24·7 (20·1 to 29·9)	409 (384 to 434)	233 (219 to 248)	27·8 (23·9 to 33·0)
	Kiribati	7 (6 to 9)	6 (5 to 8)	32·5 (27·6 to 37·7)	160 (148 to 174)	152 (141 to 167)	50·0 (43·8 to 63·0)
	Marshall Islands	5 (4 to 6)	7 (6 to 8)	21·3 (16·8 to 26·6)	108 (101 to 116)	160 (150 to 170)	20·7 (18·0 to 23·3)
	Northern Mariana Islands	10 (8 to 12)	8 (7 to 10)	6·8 (3·9 to 10·1)	288 (268 to 309)	237 (224 to 253)	8·5 (6·1 to 11·9)
	Papua New Guinea	562 (476 to 669)	7 (6 to 9)	8·1 (−9·5 to 19·7)	11 718 (10 834 to 12 664)	168 (156 to 181)	44·9 (38·9 to 54·0)
	Samoa	14 (11 to 16)	7 (6 to 8)	18·4 (14·3 to 23·1)	344 (318 to 377)	199 (185 to 217)	36·1 (30·0 to 45·9)
	Solomon Islands	41 (34 to 48)	7 (6 to 8)	22·3 (17·7 to 28·0)	856 (794 to 919)	164 (153 to 175)	30·9 (27·7 to 34·0)
	Tonga	7 (6 to 9)	7 (6 to 8)	14·1 (8·8 to 19·7)	175 (163 to 186)	184 (173 to 195)	24·5 (22·0 to 27·1)
	Vanuatu	19 (16 to 22)	7 (6 to 8)	27·2 (22·1 to 33·1)	406 (374 to 444)	166 (153 to 182)	38·2 (34·0 to 43·7)
**North Africa and Middle East**		**114 545 (60 192 to 250 395)**	**19 (10 to 40)**	**69·6 (1·2 to 219·0)**	**2 419 341 (1 598 927 to 4 560 625)**	**447 (298 to 843)**	**4·1 (−3·2 to 13·1)**
	Afghanistan	14 304 (4044 to 38 406)	37 (11 to 101)	167·8 (−1·1 to 410·1)	313 721 (101 502 to 865 339)	1367 (392 to 3875)	−15·7 (−26·0 to 35·3)
	Algeria	3284 (2785 to 3845)	8 (7 to 10)	−5·4 (−7·9 to −2·7)	106 241 (96 415 to 124 577)	276 (252 to 319)	12·5 (5·3 to 28·7)
	Bahrain	122 (104 to 145)	9 (8 to 11)	−3·9 (−8·5 to −0·1)	4500 (4186 to 4828)	309 (289 to 331)	13·1 (9·8 to 16·9)
	Egypt	7493 (6276 to 9128)	8 (7 to 10)	24·4 (16·5 to 43·7)	201 767 (184 848 to 225 697)	234 (215 to 263)	24·0 (19·9 to 29·4)
	Iran	7332 (6167 to 8763)	9 (8 to 11)	−56·2 (−76·0 to −21·2)	388 904 (258 799 to 723 827)	482 (322 to 896)	−17·4 (−26·8 to −3·6)
	Iraq	16 663 (5613 to 47 047)	37 (13 to 105)	236·9 (25·5 to 701·4)	388 270 (147 340 to 1 027 960)	1331 (498 to 3581)	−7·7 (−19·3 to 14·3)
	Jordan	1204 (663 to 2460)	14 (8 to 30)	68·3 (−2·7 to 246·7)	16 977 (15 689 to 18 348)	247 (230 to 265)	−2·5 (−6·0 to 2·6)
	Kuwait	365 (308 to 429)	10 (8 to 11)	−64·1 (−86·5 to −18·1)	13 897 (12 640 to 16 107)	342 (314 to 386)	5·9 (2·0 to 13·5)
	Lebanon	669 (449 to 1195)	12 (8 to 21)	−67·9 (−82·0 to −39·3)	91 954 (33 043 to 252 384)	1590 (567 to 4363)	−26·3 (−31·9 to −13·0)
	Libya	1269 (585 to 3231)	20 (10 to 51)	146·1 (19·9 to 512·5)	36 616 (19 378 to 78 295)	571 (313 to 1208)	89·7 (14·6 to 237·8)
	Morocco	2466 (2084 to 2896)	7 (6 to 9)	−1·6 (−7·0 to 1·9)	82 368 (76 645 to 88 686)	242 (226 to 261)	9·8 (5·9 to 13·2)
	Oman	461 (381 to 559)	10 (8 to 12)	−3·3 (−7·0 to 0·1)	14 520 (13 596 to 15 636)	312 (293 to 335)	5·3 (2·6 to 8·2)
	Palestine	504 (400 to 668)	9 (7 to 11)	−24·3 (−53·6 to 7·5)	21 989 (11 872 to 48 698)	613 (304 to 1432)	−13·0 (−22·5 to 8·9)
	Qatar	265 (219 to 320)	11 (10 to 14)	−3·5 (−6·1 to −0·8)	8857 (8206 to 9463)	375 (351 to 399)	3·3 (0·9 to 5·8)
	Saudi Arabia	2948 (2498 to 3469)	9 (8 to 11)	−13·6 (−16·2 to −10·9)	89 085 (83 584 to 94 849)	291 (274 to 308)	−8·2 (−10·2 to −5·7)
	Sudan	4274 (3005 to 6775)	10 (7 to 16)	8·0 (−13·6 to 31·9)	103 888 (78 840 to 166 051)	298 (227 to 477)	27·4 (20·2 to 39·1)
	Syria	27 672 (5097 to 90 873)	136 (25 to 441)	1878·0 (264·0 to 6479·8)	159 497 (64 351 to 401 256)	839 (367 to 2049)	228·8 (71·6 to 485·6)
	Tunisia	966 (809 to 1173)	9 (7 to 11)	9·1 (2·4 to 25·4)	31 176 (28 902 to 33 771)	268 (249 to 290)	13·7 (10·0 to 17·9)
	Turkey	7321 (5931 to 9464)	9 (8 to 12)	−6·6 (−16·3 to 15·1)	231 112 (212 709 to 258 146)	288 (265 to 321)	−1·8 (−7·6 to 9·1)
	United Arab Emirates	1051 (870 to 1261)	11 (9 to 13)	−6·8 (−8·9 to −4·6)	35 473 (33 003 to 37 961)	337 (315 to 360)	−3·1 (−5·8 to −0·3)
	Yemen	13 802 (4761 to 37 003)	42 (15 to 111)	408·7 (84·9 to 1 265·1)	75 800 (55 113 to 129 308)	314 (235 to 509)	20·1 (8·0 to 38·6)
**South Asia**		**180 120 (151 167 to 213 759)**	**11 (10 to 13)**	**−2·1 (−5·9 to 1·5)**	**4 127 359 (3 895 776 to 4 387 308)**	**256 (242 to 272)**	**20·3 (18·3 to 22·7)**
	Bangladesh	14 525 (12 236 to 17 308)	9 (8 to 11)	5·9 (1·6 to 10·5)	368 288 (340 422 to 405 978)	244 (226 to 270)	35·7 (29·9 to 44·2)
	Bhutan	81 (68 to 96)	11 (9 to 13)	0·3 (−2·4 to 3·0)	2043 (1913 to 2192)	276 (259 to 295)	26·6 (23·0 to 31·1)
	India	143 743 (120 391 to 170 991)	12 (10 to 14)	−4·2 (−8·4 to −0·4)	3 252 768 (3 074 402 to 3 448 115)	257 (243 to 272)	17·5 (16·0 to 19·0)
	Nepal	2870 (2421 to 3380)	10 (8 to 12)	0·1 (−3·0 to 3·6)	69 217 (62 575 to 78 982)	260 (236 to 293)	38·9 (29·9 to 56·2)
	Pakistan	18 902 (16 015 to 22 050)	10 (9 to 12)	14·1 (10·3 to 18·3)	435 044 (402 203 to 472 539)	255 (237 to 274)	30·0 (24·4 to 38·7)
**Sub-Saharan Africa**		**82 830 (70 088 to 98 128)**	**9 (7 to 10)**	**−30·4 (−52·9 to −12·0)**	**1 811 159 (1 537 081 to 2 405 237)**	**232 (194 to 319)**	**12·6 (6·2 to 24·2)**
**Southern sub-Saharan Africa**		**6185 (5194 to 7421)**	**8 (7 to 10)**	**−21·0 (−36·3 to −12·2)**	**140 222 (130 205 to 151 679)**	**191 (178 to 209)**	**−13·5 (−15·7 to −11·1)**
	Botswana	190 (158 to 227)	9 (7 to 10)	12·6 (9·2 to 16·6)	4289 (4000 to 4585)	195 (183 to 206)	8·7 (6·7 to 10·8)
	eSwatini	113 (94 to 135)	9 (7 to 10)	13·2 (9·9 to 16·6)	1963 (1826 to 2104)	163 (153 to 173)	−6·5 (−8·3 to −4·6)
	Lesotho	171 (143 to 203)	8 (7 to 10)	18·4 (13·9 to 22·6)	2796 (2594 to 2995)	132 (125 to 141)	−10·6 (−12·9 to −7·8)
	Namibia	185 (155 to 219)	7 (6 to 9)	0·4 (−1·8 to 2·5)	3987 (3697 to 4282)	175 (163 to 186)	5·5 (3·2 to 8·0)
	South Africa	4444 (3702 to 5367)	9 (7 to 10)	−26·5 (−42·9 to −16·0)	107 631 (99 619 to 118 234)	206 (191 to 228)	−16·4 (−18·9 to −13·6)
	Zimbabwe	1084 (920 to 1267)	7 (6 to 8)	2·9 (−0·4 to 5·7)	19 556 (18 025 to 21 070)	140 (131 to 149)	0·4 (−1·2 to 2·0)
**Western sub-Saharan Africa**		**33 433 (28 312 to 39 509)**	**9 (7 to 10)**	**−6·7 (−14·1 to −2·5)**	**648 235 (588 267 to 723 553)**	**202 (186 to 226)**	**10·1 (6·0 to 19·1)**
	Benin	906 (768 to 1067)	9 (7 to 10)	1·8 (−0·9 to 4·6)	17 000 (15 734 to 18 325)	187 (175 to 200)	5·4 (3·1 to 7·6)
	Burkina Faso	1512 (1275 to 1779)	9 (7 to 10)	−2·6 (−5·6 to 1·3)	26 537 (24 449 to 28 527)	179 (167 to 190)	11·1 (8·6 to 13·2)
	Cameroon	2059 (1721 to 2448)	9 (7 to 11)	6·1 (0·9 to 17·1)	36 183 (33 240 to 39 021)	179 (166 to 192)	−1·5 (−4·2 to 1·9)
	Cape Verde	45 (38 to 52)	8 (7 to 10)	9·4 (6·1 to 12·8)	1109 (1029 to 1189)	225 (210 to 239)	16·6 (14·2 to 19·1)
	Chad	1227 (1038 to 1437)	9 (7 to 10)	−32·0 (−61·0 to −2·6)	25 952 (21 330 to 35 887)	247 (194 to 368)	1·3 (−5·7 to 7·2)
	Côte d'Ivoire	1968 (1668 to 2297)	9 (8 to 11)	−4·6 (−7·1 to −1·9)	36 366 (33 388 to 39 550)	186 (173 to 201)	1·7 (−1·2 to 6·8)
	The Gambia	157 (132 to 184)	8 (7 to 9)	−8·2 (−10·1 to −6·3)	3081 (2800 to 3417)	196 (179 to 226)	−6·7 (−11·9 to −3·2)
	Ghana	2301 (1953 to 2690)	9 (7 to 10)	10·5 (8·0 to 13·0)	46 645 (43 313 to 50 073)	196 (183 to 209)	19·4 (15·9 to 23·3)
	Guinea	973 (822 to 1 133)	8 (7 to 9)	−6·8 (−9·1 to −4·4)	18 154 (16 817 to 19 621)	170 (159 to 183)	−5·5 (−8·5 to −1·0)
	Guinea-Bissau	157 (132 to 183)	9 (7 to 10)	−8·5 (−10·4 to −6·7)	2896 (2629 to 3280)	177 (162 to 202)	4·0 (−2·2 to 18·3)
	Liberia	324 (275 to 379)	7 (6 to 9)	−85·7 (−94·6 to −59·7)	12 285 (7581 to 24 974)	339 (203 to 698)	77·5 (15·8 to 189·6)
	Mali	1427 (1206 to 1699)	8 (7 to 10)	−12·4 (−23·3 to −5·9)	28 992 (24 231 to 39 739)	211 (176 to 292)	29·0 (10·1 to 77·9)
	Mauritania	326 (275 to 382)	9 (7 to 10)	−31·1 (−59·6 to −7·6)	7511 (6963 to 8060)	220 (205 to 235)	14·2 (12·0 to 16·6)
	Niger	1545 (1308 to 1825)	8 (7 to 9)	−15·3 (−24·7 to −9·5)	25 726 (23 640 to 27 889)	169 (157 to 182)	−2·5 (−5·1 to 1·0)
	Nigeria	16 220 (13 543 to 19 530)	9 (8 to 11)	3·0 (−2·7 to 16·5)	311 002 (284 603 to 338 096)	210 (194 to 227)	11·0 (7·2 to 17·3)
	São Tomé and Príncipe	18 (15 to 21)	9 (8 to 11)	4·0 (1·4 to 6·5)	363 (336 to 389)	231 (216 to 246)	7·7 (5·4 to 10·1)
	Senegal	1215 (1027 to 1417)	8 (7 to 10)	−3·2 (−8·5 to 0·4)	23 803 (21 961 to 25 655)	191 (178 to 205)	5·4 (2·3 to 9·7)
	Sierra Leone	498 (421 to 582)	8 (7 to 10)	−5·2 (−7·4 to −2·9)	13 960 (10 075 to 23 417)	263 (188 to 451)	51·0 (7·2 to 154·5)
	Togo	555 (468 to 649)	8 (7 to 9)	−2·3 (−4·9 to 0·2)	10 662 (9872 to 11 516)	170 (159 to 182)	−1·6 (−4·1 to 1·9)
**Eastern sub-Saharan Africa**		**33 178 (27 991 to 39 389)**	**9 (8 to 11)**	**−48·2 (−71·0 to −20·4)**	**804 687 (624 381 to 1 220 333)**	**274 (206 to 441)**	**20·4 (13·0 to 33·0)**
	Burundi	1045 (878 to 1240)	10 (8 to 11)	3·1 (−0·6 to 10·1)	27 356 (19 445 to 46 950)	311 (216 to 553)	108·2 (43·3 to 268·0)
	Comoros	63 (53 to 75)	9 (7 to 10)	−20·3 (−22·5 to −18·1)	1394 (1295 to 1495)	212 (199 to 226)	10·7 (7·7 to 14·9)
	Djibouti	87 (73 to 102)	9 (8 to 11)	−24·4 (−47·3 to −8·7)	1908 (1754 to 2107)	230 (212 to 255)	2·8 (−1·9 to 12·1)
	Eritrea	454 (383 to 532)	9 (8 to 11)	−3·4 (−6·2 to −0·4)	13 098 (9461 to 21 689)	301 (217 to 506)	80·4 (29·8 to 197·0)
	Ethiopia	8607 (7299 to 10 139)	9 (8 to 11)	−75·0 (−88·9 to −44·2)	223 360 (172 265 to 347 531)	287 (213 to 474)	13·0 (6·0 to 19·5)
	Kenya	3977 (3363 to 4640)	9 (8 to 11)	7·3 (5·9 to 8·7)	83 265 (77 387 to 89 234)	215 (201 to 228)	17·2 (15·4 to 19·8)
	Madagascar	1943 (1630 to 2280)	8 (7 to 10)	−5·0 (−7·7 to −2·0)	36 761 (33 937 to 39 548)	178 (166 to 190)	2·4 (−0·3 to 5·5)
	Malawi	1280 (1080 to 1504)	7 (6 to 9)	−13·4 (−15·8 to −10·9)	21 452 (19 639 to 23 177)	146 (136 to 156)	−4·7 (−7·5 to −1·7)
	Mozambique	2341 (1972 to 2747)	9 (7 to 10)	−24·0 (−53·9 to 0·4)	60 152 (41 299 to 106 716)	308 (188 to 595)	−18·8 (−29·7 to 0·3)
	Rwanda	908 (761 to 1067)	8 (7 to 9)	−58·4 (−79·8 to −30·3)	85 439 (32 287 to 224 253)	931 (346 to 2464)	453·5 (106·2 to 1 244·1)
	Somalia	1709 (985 to 3437)	16 (10 to 31)	−21·3 (−28·7 to −11·7)	27 548 (17 750 to 53 134)	329 (209 to 637)	33·3 (6·7 to 71·9)
	South Sudan	1455 (1158 to 1935)	11 (9 to 14)	−59·4 (−79·6 to −21·2)	36 330 (23 858 to 66 348)	358 (230 to 667)	35·6 (2·6 to 87·0)
	Tanzania	4584 (3883 to 5363)	9 (7 to 10)	−4·2 (−6·3 to −2·1)	84 663 (78 171 to 91 074)	192 (179 to 205)	6·8 (4·5 to 9·2)
	Uganda	3215 (2700 to 3747)	8 (7 to 10)	−11·7 (−29·0 to −1·6)	76 806 (57 559 to 123 332)	292 (192 to 540)	−7·5 (−24·3 to 23·6)
	Zambia	1487 (1252 to 1747)	10 (8 to 11)	2·5 (−2·1 to 5·9)	24 612 (22 713 to 26 463)	180 (168 to 192)	2·7 (0·3 to 4·8)
**Central sub-Saharan Africa**		**10 034 (8473 to 11 871)**	**9 (8 to 11)**	**−14·3 (−29·1 to −5·7)**	**218 015 (177 254 to 313 214)**	**243 (194 to 357)**	**22·1 (11·0 to 42·6)**
	Angola	2361 (1989 to 2757)	10 (8 to 11)	−38·2 (−66·2 to −9·8)	59 309 (44 235 to 95 957)	339 (234 to 593)	5·2 (−9·3 to 23·8)
	Central African Republic	434 (348 to 551)	9 (7 to 11)	14·8 (3·2 to 42·3)	8061 (6335 to 12 014)	171 (137 to 247)	32·9 (7·6 to 93·0)
	Congo (Brazzaville)	426 (360 to 508)	9 (8 to 11)	−0·5 (−3·8 to 5·8)	11 542 (8420 to 19 317)	302 (218 to 516)	81·9 (31·0 to 207·4)
	Democratic Republic of the Congo	6569 (5542 to 7777)	9 (7 to 10)	−4·5 (−7·3 to −0·3)	133 941 (112 213 to 181 770)	216 (181 to 296)	27·6 (8·5 to 74·1)
	Equatorial Guinea	81 (68 to 94)	10 (9 to 12)	11·8 (6·6 to 16·5)	1697 (1584 to 1805)	234 (219 to 247)	53·4 (49·7 to 57·2)
	Gabon	163 (138 to 191)	10 (8 to 11)	−10·9 (−12·9 to −8·9)	3464 (3219 to 3720)	225 (211 to 240)	3·6 (1·6 to 5·7)

95% uncertainty intervals are in parentheses. SDI=Socio-demographic Index.

**Figure 1:**
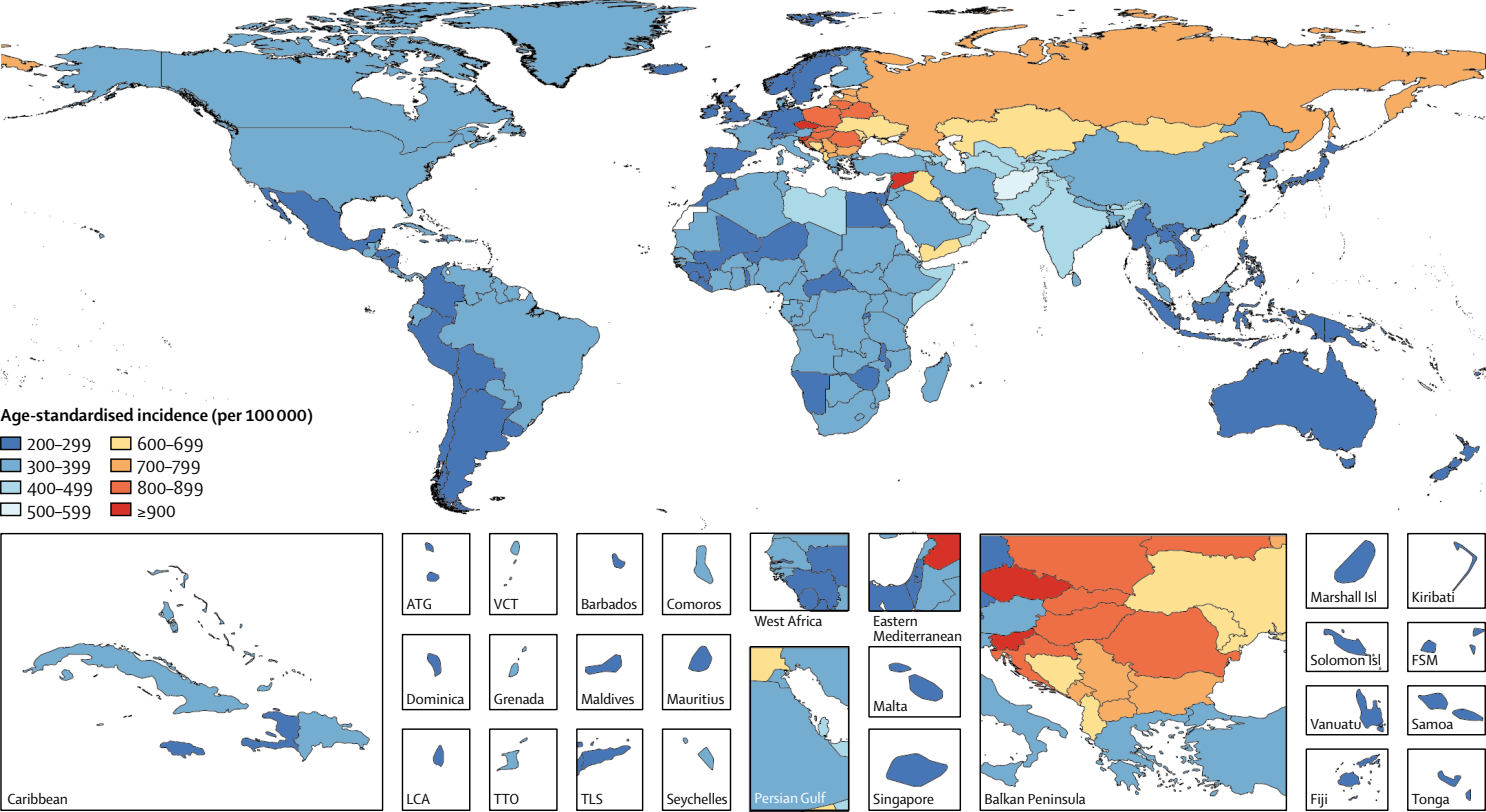
Age-standardised incidence of traumatic brain injury per 100 000 population by location for both sexes, 2016 ATG=Antigua and Barbuda. FSM=Federated States of Micronesia. Isl=Islands. LCA=Saint Lucia. TLS=Timor-Leste. TTO=Trinidad and Tobago. VCT=Saint Vincent and the Grenadines.

**Figure 2:**
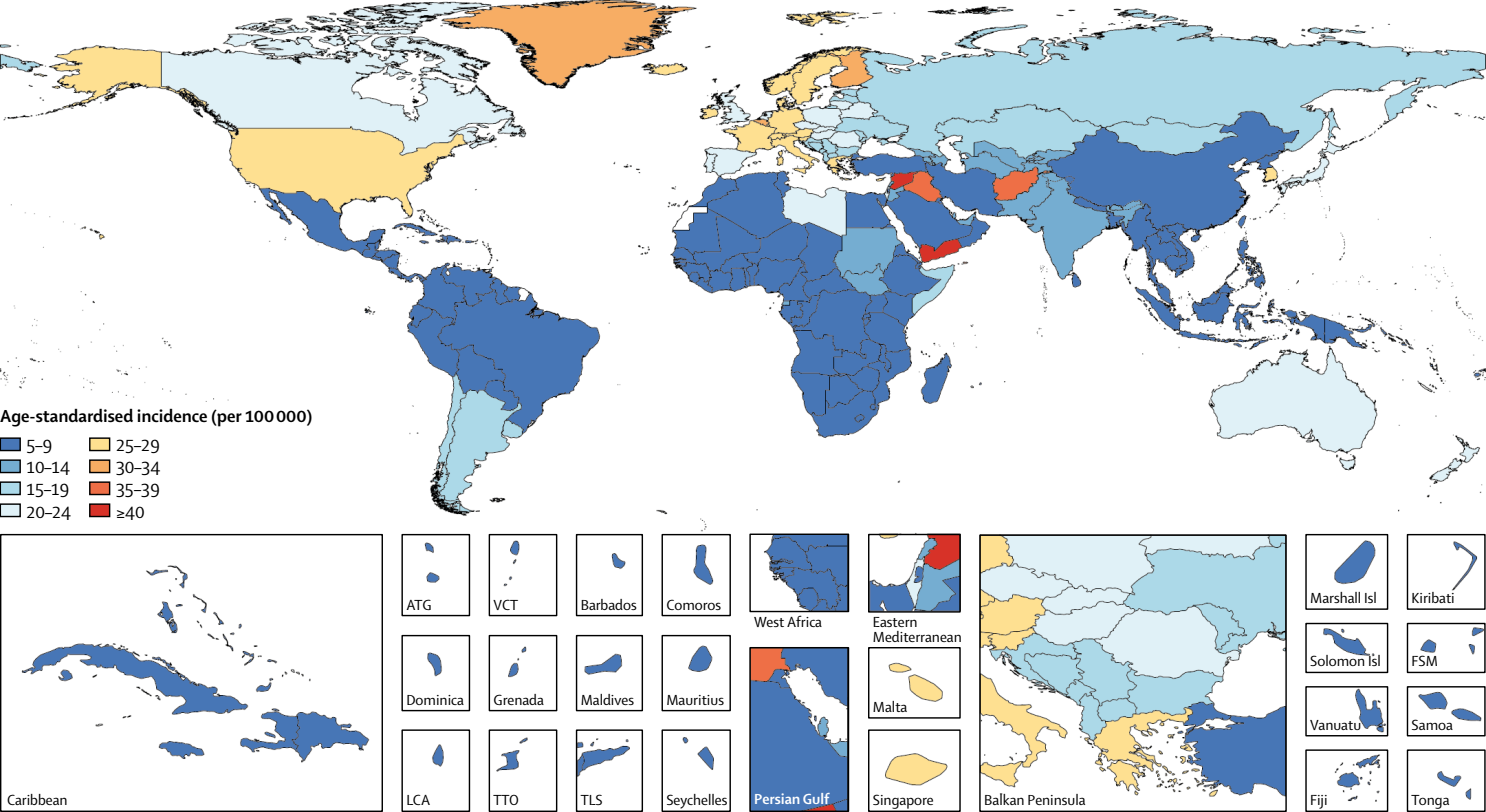
Age-standardised incidence of spinal cord injury per 100 000 population by location for both sexes, 2016 ATG=Antigua and Barbuda. FSM=Federated States of Micronesia. Isl=Islands. LCA=Saint Lucia. TLS=Timor-Leste. TTO=Trinidad and Tobago. VCT=Saint Vincent and the Grenadines.

Figure 1, Figure 2 show the age-standardised incidence by country for 2016 for TBI and SCI, respectively. Central Europe, eastern Europe, and central Asia had substantially higher incidence rates of TBI than the rest of the world; at the regional level, the age-standardised incidence rate was highest in central Europe, at 857 (95% UI 750–988) per 100 000 (table 1). Syria had the highest age-standardised incidence rate of TBI of any country, with 1322 (95% UI 481–2779) cases per 100 000. Slovenia (1092 [938–1294] per 100 000) and the Czech Republic (1022 [885–1191] per 100 000) had the next highest age-standardised incidence rates. The incidence rates for SCI were highest in the high SDI regions high-income North America (26 [20–33] per 100 000) and Western Europe (26 [20–33] per 100 000; table 2). However, at a country level, Syria had the highest age-standardised incidence rate of SCI (136 [25–441] per 100 000), followed by Yemen (42 [15–111] per 100 000), Iraq (37 [13–105] per 100 000), and Afghanistan (37 [11–101]; table 2).

In terms of individuals living with disability from these conditions in 2016, TBI had a global age-standardised prevalence of 759 (95% UI 731–788) per 100 000 (table 1), and SCI had a global age-standardised prevalence of 368 (340–409) per 100 000 (table 2). These estimates corresponded to 55 million (53–58) individuals with TBI and 27 million (25–30) with SCI (for unrounded estimates see table 1). From 1990 to 2016, the age-standardised prevalence of TBI increased by 8·4% (95% UI 7·7 to 9·2; table 1), whereas that of SCI decreased non-significantly by −0·2% (−2·1 to 2·7; table 2). Age-standardised prevalence of TBI was high in the super-region of cental Europe, eastern Europe, and central Asia at 1539 (1464–1614) per 100 000, representing roughly 7·5 million prevalent cases (7·1–7·9). Age-standardised prevalence for SCI was highest in high SDI regions—specifically western Europe (854 [780–945 per 100 000) and high-income Asia Pacific (821 [747–907] per 100 000; table 2).

TBI and SCI caused 8·1 million (95% UI 6·0–10·4) and 9·5 million (6·7–12·4) YLDs, respectively, in 2016. The age-standardised YLD rates were 111 (82–141) per 100 000 for TBI and 130 (90–170) per 100 000 for SCI (appendix 2). The global age-standardised YLD rates per 100 000 population for TBI increased by 8·5% (7·6–9·3) from 1990 to 2016 and those for SCI decreased by 10·0% (7·0–13·3) from 1990 to 2016. At the country level, for TBI, the distribution of YLDs was similar to those of incidence and prevalence. Specifically, countries in central Europe, eastern Europe, and central Asia had the highest age-standardised YLD rates, with country-specific rates ranging from 135 (99–175) per 100 000 in Tajikistan to 335 (241–421) per 100 000 in Slovenia. For SCI, the high-income super-region had the highest age-standardised YLD rates (229 [159–303] per 100 000). Within these locations, Finland (287 [197–381] per 100 000), Ireland (283 [192–373] per 100 000), and Israel (282 [181–396] per 100 000) had the highest age-standardised YLD rates.

**Figure 3:**
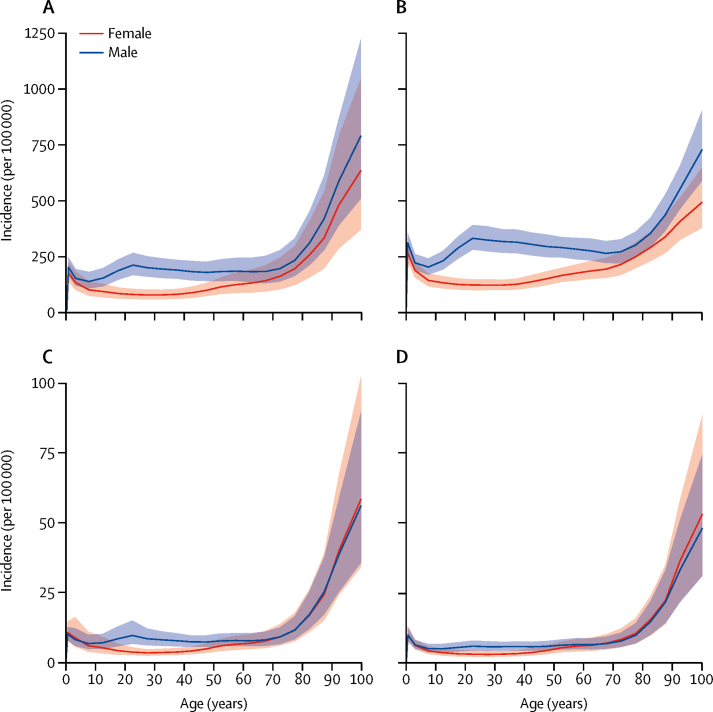
Global incidence of minor (A) and moderate or severe (B) traumatic brain injury, and of spinal cord injury at neck level (C) and below neck level (D), by age and sex, 2016 Shaded regions represent 95% uncertainty intervals.

Figure 3 shows the global age-specific and sex-specific incidence rates per 100 000 for minor TBI, moderate or severe TBI, spinal cord lesions at the neck, and spinal cord lesions below the neck for 2016. For TBI, these figures show divergent patterns between males and females that start in teenage years and extend to ages 50–60 years (figure 3). At older ages (ie, older than 60 years), the sex-specific incidence rates in males and females is similar (figure 3). The incidence is more similar between the sexes for both subtypes of SCI than for TBI, although men have higher incidences than women of spinal cord lesions at the neck level at ages 20–40 years (figure 3).

**Figure 4:**
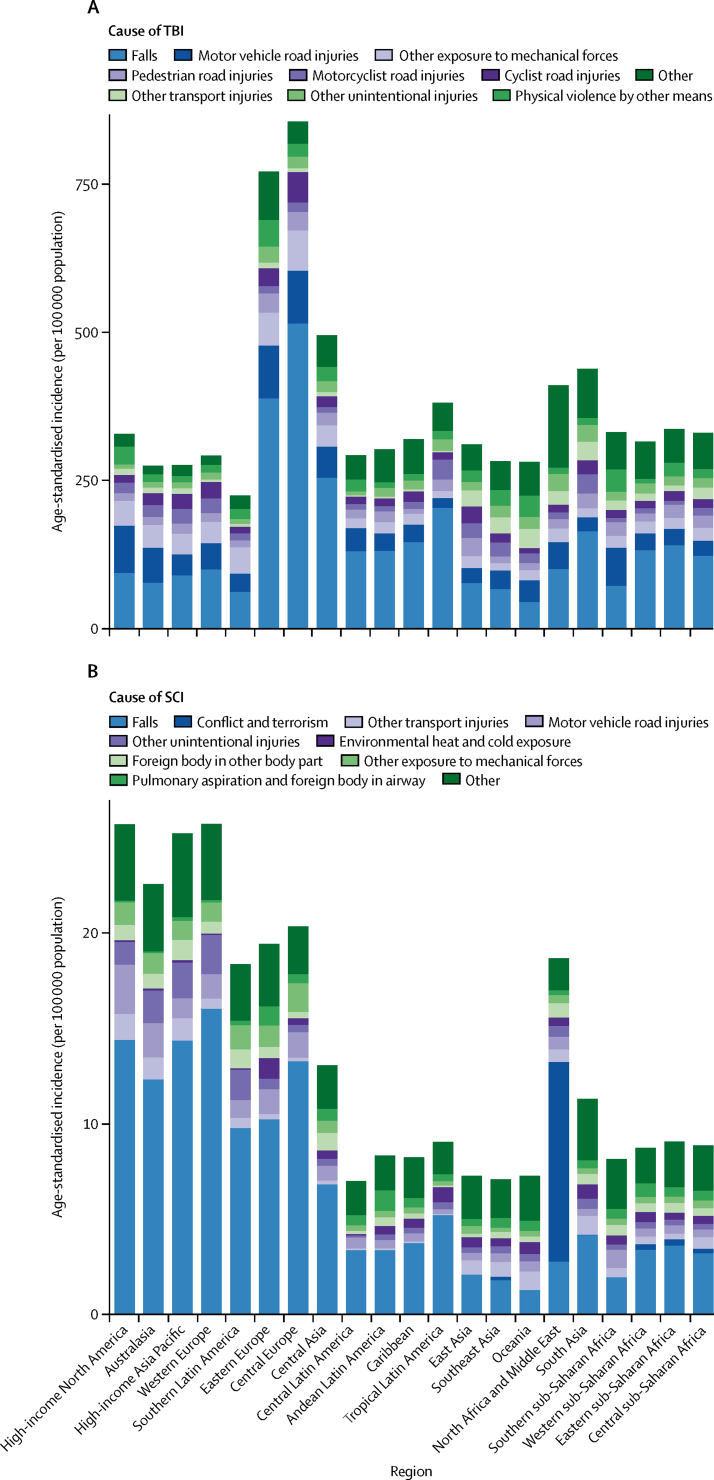
Cause composition of age-standardised incidence of traumatic brain injury (A) and spinal cord injury (B) by Global Burden of Disease region for both sexes, 2016

The proportion of causes leading to TBI and SCI by region are shown in figure 4. In general, falls were the main cause of TBI. In some regions, such as central Europe, more than 50% of the age-standardised incidence of TBI was caused by falls; in other regions, such as Oceania, falls were still the predominant cause but accounted for a smaller proportion of the age-standardised incidence (figure 4). In addition to having high age-standardised incidence, prevalence, and YLDs attributable to TBI, central and eastern Europe also had the highest incidence of TBI caused by falls. The second most common cause of TBI in most regions was motor vehicle road injuries (figure 4A). The main cause of SCI in most regions was also falls, which accounted for more than 50% of age-standardised incidence in nine different GBD regions (figure 4). Conflict and terrorism was the most common cause in North Africa and the Middle East in 2016 (figure 4B).

## Discussion

This study, in which we used the GBD framework to estimate the non-fatal burden of TBI and SCI, is to our knowledge the first effort to quantify the burden of these conditions at global, regional, and national levels for all ages and sexes, and over time, from 1990 to 2016. Globally, these conditions cause non-fatal health loss that is distributed across various levels of income, geographies, and the lifespan, and represent a substantial proportion of global injury burden that could be averted through injury prevention and safety measures.

We identified an increase in global age-standardised incidence, prevalence, and YLDs of TBI between 1990 and 2016. This increase probably reflects the increasing rates of falls and road injuries over this period, which could in turn be due to increased use of motor vehicles, unsafe road conditions, and, in some areas, increased rates of alcohol consumption or unsafe infrastructure.^22^, ^23^, ^24^ By contrast, we noted no significant change in the age-standardised incidence or prevalence of SCI, although with global population growth, the absolute number of people living with the effects of SCI is expected to increase. The increasing global incidence of both TBI and SCI starting approximately at age 70 years also shows the importance of preventive measures for injuries through all years of life—particularly in the context of an ageing global population—and of adequate access to acute medical care resources such as emergency medical services and emergency department care.

Regional patterns differed between TBI and SCI. The highest incidence rates of TBI were in central Europe, eastern Europe, and central Asia, whereas the highest incidence rates of SCI were in high-income North America, western Europe, and high-income Asia Pacific. Conflict-affected countries in the Middle East—ie, Syria, Yemen, and Iraq—and Afghanistan had the highest country-specific incidence of SCI, and Syria also had the highest incidence of TBI. Rates of TBI and SCI were lower in some low SDI countries in regions such as sub-Saharan Africa, which generally corresponded with the geographical patterns of falls and road injuries in those regions as reported in GBD 2016.^3^, ^14^, ^16^ These variations in the underlying causes of TBI and SCI probably explain much of the geographical variation in the incidence of TBI and SCI. Access to health-care resources could also explain some geographical variation. For example, the higher prevalence of SCI in North America and western Europe could be related to survival bias in high SDI areas, whereby medical services have led to successful resuscitation in injury victims who otherwise would have died without receiving a TBI or SCI diagnosis code. The high rates of TBI in central Europe, eastern Europe, and central Asia generally correspond with the high all-injury rate estimated in those regions in GBD 2016.^3^, ^4^, ^16^

Our findings show that, globally, falls and road injuries were the most important cause of non-fatal cases of both TBI and SCI, reflecting the findings for all 328 diseases and injuries from GBD 2016, in which falls were the tenth leading cause of age-standardised YLDs from 1990 to 2016.^3^ This burden of falls was particularly evident in our study for central Europe, eastern Europe, and central Asia, where falls were the second most common cause of disability in 1990 and the third most common cause in 2016.^3^ Although the context in which a fall occurred could not be established in this study because of a lack of International Classification of Diseases (ICD) coding detail, falls can be preventable irrespective of where they occur. Falls leading to SCI have been associated with alcohol use in countries such as Estonia, so risk factor profiles across countries could explain some geographical patterns in this study.^22^ Road injuries were also important causes of these conditions, suggesting that achievement of Sustainable Development Goal 3.6 (“By 2020, halve the number of global deaths and injuries from road traffic accidents”) could reduce the burden of conditions such as TBI and SCI that can result from road injuries.^25^

Our estimates for TBI incidence diverged from estimates in other published literature. Our study relied on cause-of-injury models that by design estimate the incidence of injuries requiring medical care. A limitation of this approach is that some people with TBI, particularly mild TBI, might not seek medical attention after injury and are thus not captured in the analysis, which could lead to underestimation of the global burden of TBI.^26^, ^27^ In a study^28^ done in New Zealand, in which proactive screening methods were used to contact people after an accident to their upper body (including use of broad ICD-10 codes [S00–09] in addition to community-based case-ascertainment sources to identify individuals not seeking medical treatment), the incidence of TBI was 790 per 100 000 (substantially higher than that in our study), and approximately 30% of people with mild TBI did not seek medical attention soon after their TBI. However, this study was done in only one country, and the findings can probably not be generalised to the global population. However, the findings of that study^28^ emphasise the need for other international studies to use a comprehensive community-based approach for case ascertainment to increase the accuracy of GBD estimates.

In general, our study had similar limitations to other GBD studies, but with the added complexity and uncertainty of measuring TBI and SCI within other injury estimates, which has not been done previously in the GBD framework. In terms of TBI-specific and SCI-specific limitations, we used medical record data extensively in our modelling process, which might not be representative of the entire population. This point is pertinent because most of the dual-coded clinical data that was used in the derivation of cause–nature proportions was from high-income countries. Additionally, the derivation of the incidence coefficient that adjusts for injuries receiving outpatient care was based on limited data. These factors could have introduced selection bias, which was addressed to some extent by incorporation of income and health-care access in our modelling process. However, by relying on medical care records, we potentially did not include people with mild TBI who did not seek medical care, which therefore could be a source of detection bias leading to underestimation, although we addressed this issue by using cause-of-injury incidence models for all injuries requiring medical care, followed by a Dirichlet-based modelling approach of cause–nature combinations.^26^, ^27^, ^28^ An additional limitation stems from the studies examining how TBI and SCI can occur together.^29^ A proportion of people can experience an SCI from a traumatic event and also experience TBI, and because of the disability-ranking approach that we used in our cause–nature proportion analysis, these patients would be assigned SCI as their nature of injury. Experiencing both SCI and TBI can also complicate recovery, and presence of non-brain injuries in people with TBI can affect survival,^30^ although estimates of the cumulative effect are outside the scope of this analysis. The ICD codes used to identify SCI cases also include some injuries that do not necessarily lead to paraplegia or tetraplegia, and some such injuries, such as spinal cord contusions, can improve over time. Additionally, emerging evidence about long-term deficits such as dementia, stroke, and increased risk of engagement in antisocial behaviour linked to TBI were not included in our disability computation.^31^, ^32^, ^33^, ^34^ The long-term neurological and psychological sequelae of TBI are poorly understood, and the epidemiological, neuropathological, and psychiatric analyses intended to understand the resultant disabilities will be important to incorporate in future assessments. Similarly, our analysis does not capture cohort effects over time, a limitation that can be addressed in future GBD studies. Overall, the long-term sequelae due to TBI and SCI suggest that further work in terms of measurement of long-term disability is needed to measure the effect of these conditions more accurately, and to ensure that the disability weights accurately reflect the health loss observed in clinical practice and experienced by individuals; such further work could influence future research into disability-weight measurement via health loss surveys. The limitations we describe also show how more research is needed, particularly in low-income areas of the world, to collect comprehensive injury data. Focusing of resources on injury epidemiology data could improve the accuracy of future epidemiological assessments of TBI and SCI.

In conclusion, the age-standardised incidence, prevalence, and YLD of TBI are increasing globally, whereas age-standardised rates of SCI have not changed (although the number of individuals with SCI is likely to be increasing globally). In view of the expense and complexity of managing patients with TBI and SCI, ministries of health, medical systems, and social support infrastructure should focus on development and improvement of injury-prevention strategies, although maintenance of short-term and long-term care pathways to mitigate health loss and improve outcomes among patients with TBI and SCI is also crucial. Finally, measurement of the burden of these conditions could be improved with the establishment of registry systems for patients with TBI and SCI worldwide, which could help to facilitate further research and intervention efforts and improve the accuracy of future epidemiological assessments of these important conditions.

## Supplementary Materials

Supplementary appendix1

Supplementary appendix2

## References

[1] Maas AIR, Menon DK, Adelson PD (2017). Traumatic brain injury: integrated approaches to improve prevention, clinical care, and research. Lancet Neurol.

[2] Te Ao B, Brown P, Tobias M (2014). Cost of traumatic brain injury in New Zealand: evidence from a population-based study. Neurology.

[3] GBD 2016 Disease and Injury Incidence and Prevalence Collaborators (2017). Global, regional, and national incidence, prevalence, and years lived with disability for 328 diseases and injuries for 195 countries, 1990–2016: a systematic analysis for the Global Burden of Disease Study 2016. Lancet.

[4] Burns JF In Europe, echoes of America as concussions spur debate. https://www.nytimes.com/2014/04/06/sports/in-europe-echoes-of-america-as-concussions-spur-debate.html.

[5] Scholten AC, Polinder S, Panneman MJM, van Beeck EF, Haagsma JA (2015). Incidence and costs of bicycle-related traumatic brain injuries in the Netherlands. Accid Anal Prev.

[6] Emanuelson I, Wendt LV (1997). Epidemiology of traumatic brain injury in children and adolescents in south-western Sweden. Acta Paediatr Oslo Nor 1992.

[7] Singh A, Tetreault L, Kalsi-Ryan S, Nouri A, Fehlings MG (2014). Global prevalence and incidence of traumatic spinal cord injury. Clin Epidemiol.

[8] Canadian Institute for Health Information Head injuries in Canada: a decade of change (1994–1995 to 2003–2004). https://secure.cihi.ca/estore/productFamily.htm?pf=PFC1360&lang=fr&media=0.

[9] Rutland-Brown W, Langlois JA, Thomas KE, Xi YL (2006). Incidence of traumatic brain injury in the United States, 2003. J Head Trauma Rehabil.

[10] Bruns J, Hauser WA (2003). The epidemiology of traumatic brain injury: a review. Epilepsia.

[11] Taylor CA (2017). Traumatic brain injury-related emergency department visits, hospitalizations, and deaths—United States, 2007 and 2013. MMWR Surveill Summ.

[12] Chiu W-T, Lin H-C, Lam C, Chu S-F, Chiang Y-H, Tsai S-H (2010). Review paper: epidemiology of traumatic spinal cord injury: comparisons between developed and developing countries. Asia Pac J Public Health.

[13] Rahimi-Movaghar V, Sayyah MK, Akbari H (2013). Epidemiology of traumatic spinal cord injury in developing countries: a systematic review. Neuroepidemiology.

[14] GBD 2016 Causes of Death Collaborators (2017). Global, regional, and national age-sex specific mortality for 264 causes of death, 1980–2016: a systematic analysis for the Global Burden of Disease Study 2016. Lancet.

[15] GBD 2016 Risk Factor Collaborators (2017). Global, regional, and national comparative risk assessment of 84 behavioural, environmental and occupational, and metabolic risks or clusters of risks, 1990–2016: a systematic analysis for the Global Burden of Disease Study 2016. Lancet.

[16] GBD 2016 DALYs and HALE Collaborators (2017). Global, regional, and national disability-adjusted life-years (DALYs) for 333 diseases and injuries and healthy life expectancy (HALE) for 195 countries and territories, 1990–2016: a systematic analysis for the Global Burden of Disease Study 2016. Lancet.

[17] GBD 2016 Mortality Collaborators (2017). Global, regional, and national under-5 mortality, adult mortality, age-specific mortality, and life expectancy, 1970–2016: a systematic analysis for the Global Burden of Disease Study 2016. Lancet.

[18] Foreman KJ, Lozano R, Lopez AD, Murray CJ (2012). Modeling causes of death: an integrated approach using CODEm. Popul Health Metr.

[19] Vos T, Flaxman AD, Naghavi M (2012). Years lived with disability (YLDs) for 1160 sequelae of 289 diseases and injuries 1990–2010: a systematic analysis for the Global Burden of Disease Study 2010. Lancet.

[20] Moorin R, Miller TR, Hendrie D (2014). Population-based incidence and 5-year survival for hospital-admitted traumatic brain and spinal cord injury, Western Australia, 2003–2008. J Neurol.

[21] Salomon JA, Haagsma JA, Davis A (2015). Disability weights for the Global Burden of Disease 2013 study. Lancet Glob Health.

[22] Sabre L, Pedai G, Rekand T, Asser T, Linnamägi U, Kõrv J (2012). High incidence of traumatic spinal cord injury in Estonia. Spinal Cord.

[23] WHO Road traffic injuries. http://www.who.int/news-room/fact-sheets/detail/road-traffic-injuries.

[24] WHO Falls. http://www.who.int/news-room/fact-sheets/detail/falls.

[25] UNDP Goal 3 targets. http://www.undp.org/content/undp/en/home/sustainable-development-goals/goal-3-good-health-and-well-being/targets.html.

[26] Barker-Collo SL, Feigin VL (2009). Capturing the spectrum: suggested standards for conducting population-based traumatic brain injury incidence studies. Neuroepidemiology.

[27] Barker-Collo S, Theadom A, Jones K, Feigin VL, Kahan M (2016). Accuracy of an International Classification of Diseases code surveillance system in the identification of traumatic brain injury. Neuroepidemiology.

[28] Feigin VL, Theadom A, Barker-Collo S (2013). Incidence of traumatic brain injury in New Zealand: a population-based study. Lancet Neurol.

[29] Budisin B, Bradbury CCLB, Sharma B (2016). Traumatic brain injury in spinal cord injury: frequency and risk factors. J Head Trauma Rehabil.

[30] Brown AW, Leibson CL, Mandrekar J, Ransom JE, Malec JF (2014). Long-term survival after traumatic brain injury: a population-based analysis controlled for nonhead trauma. J Head Trauma Rehabil.

[31] Chen Y-H, Kang J-H, Lin H-C (2011). Patients with traumatic brain injury: population-based study suggests increased risk of stroke. Stroke.

[32] Mendez MF (2017). What is the relationship of traumatic brain injury to dementia?. J Alzheimers Dis.

[33] Williams WH, Chitsabesan P, Fazel S (2018). Traumatic brain injury: a potential cause of violent crime?. Lancet Psychiatry.

[34] Fann JR, Ribe AR, Pedersen HS (2018). Long-term risk of dementia among people with traumatic brain injury in Denmark: a population-based observational cohort study. Lancet Psychiatry.

