# Correction to *Lancet Neurol* 2019; 18: 56–87

**DOI:** 10.1016/S1474-4422(21)00383-5

**Published:** 2021-12

*GBD 2016 Traumatic Brain Injury and Spinal Cord Injury Collaborators. Global, regional, and national burden of traumatic brain injury and spinal cord injury, 1990–2016: a systematic analysis for the Global Burden of Disease Study 2016.* Lancet Neurol *2019; **18:** 56–87*—In this Article, Shafiu Mohammed should have been included in the GBD 2016 Traumatic Brain Injury and Spinal Cord Injury Collaborators. The affiliations have also been updated. These corrections have been made to the online version as of Nov 17, 2021.

