# Supplementary material for: Global, regional, and national burden of traumatic brain injury and spinal cord injury, 1990–2016: a systematic analysis for the Global Burden of Disease Study 2016
Source: Lancet Neurol. 2019 Jan;18(1):56–87. doi: 10.1016/S1474-4422(18)30415-0 (PMC6291456; doi:10.1016/S1474-4422(18)30415-0)
Supplement: Supplementary appendix1 [file mmc1.pdf]

THE LANCET
Neurology

Supplementary appendix 1

This appendix formed part of the original submission and has been peer reviewed. We post it as supplied by the authors.

Supplement to: GBD 2016 Traumatic Brain Injury and Spinal Cord Injury Collaborators. Global, regional, and national burden of traumatic brain injury and spinal cord injury, 1990 to 2016: a systematic analysis for the Global Burden of Disease Study 2016. *Lancet Neurol* 2018; published online Nov 26. http://dx.doi.org/10.1016/S1474-4422(18)30415-0.

Appendix 1, Section 1

Case definitions

External injury causes

The injuries estimation process for non-fatal health outcomes encompasses a range of 28 causes, including transport injuries, falls, drowning, self-harm, interpersonal violence, and animal contact. Injury incidence is defined using ICD-9 codes E000-E999 and ICD-10 chapters V to Y. For non-fatal estimation, Chapters S and T in ICD-10 and codes 800-999 in ICD9 are used to estimate morbidity. Each of these 28 causes can result in a variety of physical injury sequelae, including traumatic brain injury and spinal cord injury, which we call the "nature of injury."

Traumatic brain injury

Traumatic brain injury (TBI) is defined by an external force applied to the head causing injury to the brain manifest by different levels of clinical severity. TBI can be differentiated into mild, moderate, and severe based on Glasgow Coma Scale (GCS) and neurological imaging. The ICD-10 codes for TBI are F07.2, F07.8, F07.81, F07.89, F07.9, S06, S07, T90.2, and T90.5. Among these, S06.1 designates minor TBI while the others designate moderate or severe TBI. The ICD-9 codes for TBI are 850 (mild), 852 and 907 (moderate or severe). These codes were identified by physician review.

Spinal cord injury

Spinal cord injury (SCI) is defined as an external injury to the vertebral column which leads to mechanical compression or distortion of the spinal cord leading to diminished or lost function below the spinal cord level of the injury. We separated SCI into lesions at the neck level and below the neck level given the different levels of disability that can be caused by lesions at different levels. The ICD-10 codes for SCI are S14 and T91.3 (neck level) and S24 and S34 (below neck), and the ICD-9 codes are 806.0, 806.1, and 952.0 (at neck), 806.2-806.9, 952.1, 952.2, 952.3, 952.4, 952.8, and 952.9 (below neck). These codes were identified by physician review.

External cause modelling strategy

Overall modelling approach

Each external cause of injury is modelled in DisMod-MR 2.1 as described in the methods section of the manuscript. Additional details on the modelling strategy are provided below, and more information on the overall injuries modelling process including injury-specific details is described in the methods appendix of the GBD 2016 publication on non-fatal outcomes [1].

External cause of injury models

As described in the manuscript, each external cause of injury is modelled using incidence data and cause-specific mortality rate (CSMR) estimates in DisMod-MR 2.1. These models use study-level covariates and location-specific covariates to inform the estimates.

The binary covariates listed below in Appendix Table 1 were used in DisMod-MR 2.1 to account for differences in study populations and metrics among data sources. With DisMod-MR 2.1, we estimated incidence of injuries that warranted inpatient care. Thus, for clinical data, we adjusted outpatient incidence rates, which are systematically higher, to align with inpatient incidence rates. Similarly, for scientific literature sources, we used two covariates that adjusted for treatment availability and the grouping of inpatients and outpatients within one source. The outpatient adjustment was performed in every external cause of injury model as we have outpatient data for all causes. The literature data adjustments were performed depending on the literature data used in each model. After modelling with DisMod-MR 2.1, the multiplier calculated in DisMod-MR 2.1 that adjusts outpatient data was used to recalculate outpatient incidence so that we could ultimately model two severities of injuries: those warranting inpatient care and those warranting outpatient care.

Appendix 1, Section 2 shows a flowchart of the overall measurement approach.

Appendix Table 1: Study covariates used in external cause of injury models in DisMod-MR 2.1

Study covariate	Notation	
	**Less desirable (1)**	**Reference (0)**
**Outpatient**	Outpatient study population	Inpatient study population
**Medcare**	Reported being injured and warranted formal care but did not receive it	Reported being injured and received formal care
**Injury receiving formal care, inpatient and outpatient**	Identifies cases that received formal care, both inpatient and outpatient	Identifies cases that received inpatient care

Input data

Systematic reviews of injuries incidence data for all external causes of injury were conducted for GBD 2010 and updated as new data and literatures were available in GBD 2013, GBD 2015, and GBD 2016. More detailed information on the data-seeking process for injuries is available in past GBD literature [1,2].

Inclusion criteria of the systematic reviews were:

- Representative, population-based surveys
- Reporting of incidence of injuries

Consistent with modelling strategies for other causes in the GBD framework, injuries estimation also utilised hospital and clinic diagnosis codes for locations where these data were available. In these sources, injury incidence is defined using ICD-9 codes E800-E999 and ICD-10 chapters V to Y, except for deaths and cases of drug overdoses and accidental alcohol poisoning, which are classified under drug and alcohol use disorders.

Appendix Table 2a below illustrates the counts of external cause of injury incidence data and Appendix Table 2b show the counts by region. Not shown are 5,264 country-years of cause-specific mortality rate (CSMR) inputs for each external cause that are provided from the central cause of death ensemble model and cause of death correction process used in the cause of death estimation framework in the GBD study. More specific detail on geographical coverage of data is provided elsewhere in GBD literature [2].

Appendix Table 2a: Counts of incidence data sources by external cause

External cause	Sources	Countries/subnational locations
Road injuries	149	199
Other transport injuries	52	93
Falls	119	198
Drowning	86	128
Fire, heat, and hot substances	100	137
Poisonings	96	136
Exposure to mechanical forces	85	128

Adverse effects of medical treatment	59	110
Animal contact	95	134
Pulmonary aspiration and foreign body in airway	75	118
Other unintentional injuries	38	118
Self-harm	89	129
Interpersonal violence	96	135
Pedestrian road injuries by road vehicle	39	96
Cyclist road injuries	38	96
Motorcyclist road injuries	38	94
Motor vehicle road injuries	38	91
Other road injuries	36	89
Unintentional firearm injuries	59	120
Unintentional suffocation	59	120
Other exposure to mechanical forces	59	120
Venomous animal contact	75	117
Non-venomous animal contact	75	120
Foreign body in eyes	74	119
Foreign body in other body part	59	119
Assault by firearm	60	118
Assault by sharp object	60	121
Assault by other means	59	120
Self-harm by firearm	36	89
Self-harm by other specified means	36	89

Appendix Table 2b: Counts of incidence data sources for all causes by region

GBD Region	Sources
East Asia	15
Southeast Asia	16
Oceania	3
Central Asia	4
Central Europe	12

Eastern Europe	6
Australasia	5
Western Europe	36
Southern Latin America	4
High-income North America	20
Caribbean	5
Andean Latin America	1
Central Latin America	11
Tropical Latin America	7
North Africa and Middle East	12
South Asia	16
Central Sub-Saharan Africa	1
Eastern Sub-Saharan Africa	11
Southern Sub-Saharan Africa	6
Western Sub-Saharan Africa	11

TBI and SCI non-fatal outcomes estimation

The results from the external cause models described above then go through the following process.

1. Cause-nature proportions

Cause-nature matrices were applied to the incidence estimates produced with DisMod-MR 2.1. For each cause of injury, we developed matrices for the following categories: inpatient/outpatient and high/low-income countries. To generate the cause-nature matrices, we utilised various dual-coded data sources, which are described in Appendix Table 3, and used Dirichlet regression to estimate what proportion of a certain cause of injury resulted in a specific nature of injury.

Appendix Table 3: Hospital discharge records used for calculating cause-nature matrices

Dual-coded data	Source	Description
Argentina Public Hospital Injury Discharges 2007–2011,	Directorate of Health Statistics and Information, Ministry of Health (Argentina)	Public hospital records aggregated to the country level
China Injury Comprehensive Surveillance Study 2009–2011	Chinese Center for Disease Control and Prevention (CCDC)	Inpatient data collected as part of an injury surveillance study in several subnational sites in China: Chongqing, Dalian, Ningbo, Songjiang, Wuzhong, Zhanjiang, and Zhuhai

China National Injury Surveillance System 2006–2014	Chinese Center for Disease Control and Prevention (CCDC), Ministry of Health (China)	Nationally representative surveillance system of outpatients with injuries
United Kingdom - England Hospital Episode Statistics 2002–2015	NHS England	Records of inpatient, outpatient, and emergency attendances at NHS hospitals in England
Netherlands National Medical Registry (LMR) 1998–2012	Dutch Hospital Data (DHD)	Cases of inpatient care in Dutch hospitals
Netherlands Injury Surveillance System 1998–2012	Consumer Safety Institute (Netherlands)	Emergency department data from a representative sample of private hospitals in the Netherlands
Argentina Injury Surveillance System Tabulations 2008	National Institute of Epidemiology, National Administration of Laboratories and Health Institutes (ANLIS), GBD 2010 Injury Expert Group	Inpatient administrative records
United States National Hospital Discharge Survey 1990–2006	National Center for Health Statistics, Centers for Disease Control and Prevention	Sample of inpatient records selected from a national sample of non-Federal, short-stay hospitals
Bulgaria Hospital Discharge Injury Tabulations 2004	Global Burden of Disease 2010 Injury Expert Group	Inpatient administrative records
Czech Republic Hospital Discharge Injury Tabulations 2004	Global Burden of Disease 2010 Injury Expert Group	Inpatient administrative records
Denmark Hospital Discharge Injury Tabulations 2005	Global Burden of Disease 2010 Injury Expert Group	Inpatient administrative records
Estonia Hospital Discharge Injury Tabulations 2003	Global Burden of Disease 2010 Injury Expert Group	Inpatient administrative records
Hungary Hospital Discharge Injury Tabulations 2004	Global Burden of Disease 2010 Injury Expert Group	Inpatient administrative records
Iceland Hospital Discharge Injury Tabulations 2005	Global Burden of Disease 2010 Injury Expert Group	Inpatient administrative records
Italy Hospital Discharge Injury Tabulations 2003	Global Burden of Disease 2010 Injury Expert Group	Inpatient administrative records
Latvia Hospital Discharge Injury Tabulations 2004	Global Burden of Disease 2010 Injury Expert Group	Inpatient administrative records
Malta Hospital Discharge Injury Tabulations 2005	Global Burden of Disease 2010 Injury Expert Group	Inpatient administrative records
Netherlands Hospital Discharge Injury Tabulations 2004 - 2005	Global Burden of Disease 2010 Injury Expert Group	Inpatient administrative records
Norway Hospital Discharge Injury Tabulations 2004	Global Burden of Disease 2010 Injury Expert Group	Inpatient administrative records
Portugal Hospital Discharge Injury Tabulations 2004	Global Burden of Disease 2010 Injury Expert Group	Inpatient administrative records

Slovenia Hospital Discharge Injury Tabulations 2004	Global Burden of Disease 2010 Injury Expert Group	Inpatient administrative records
Sweden Hospital Discharge Injury Tabulations 2004	Global Burden of Disease 2010 Injury Expert Group	Inpatient administrative records
Macedonia Hospital Discharge Injury Tabulations 2005	Global Burden of Disease 2010 Injury Expert Group	Inpatient administrative records
Spain Hospital Discharge Injury Tabulations 2000–2007	Global Burden of Disease 2010 Injury Expert Group	Inpatient administrative records
Mauritius Hospital Discharge Injury Tabulations 2003–2007	Ministry of Health and Quality of Life (Mauritius), Global Burden of Disease 2010 Injury Expert Group	Inpatient administrative records
Mexico Ministry of Health Hospital Discharge Tabulations 2005	Secretariat of Health (Mexico)	Inpatient administrative records
Brazil Hospital Information System 1997–2014	Rio de Janeiro, Brazil: Ministry of Health (Brazil)	Nationally representative administrative discharge records for inpatients and outpatients
Austria Hospital Inpatient Discharges 2001–2010	Federal Ministry of Health (Austria), Statistics Austria	Inpatient administrative records
Canada Discharge Abstract Database 1994–2009	Canadian Institute for Health Information (CIHI)	Hospital administrative data on inpatient discharges from acute care facilities in all Canadian provinces and territories other than Quebec
Mexico Ministry of Health Hospital Discharges 2003–2011	Secretariat of Health (Mexico)	Discharge database from Mexico's Automated Hospital Discharge System (SAEH)
New Zealand National Minimum Dataset 2000–2014	Ministry of Health (New Zealand)	Hospital discharge data for inpatients and day patients
Chile Hospital Discharges 2001–2011	Santiago, Chile: Ministry of Health (Chile)	Administrative discharge records for inpatients

Within the dual-coded data sources, multiple diagnoses were common. In GBD 2010 [2], we tried to apportion co-occurring injuries using regression methods. However, that ended up with implausibly high amounts of long-term disability being assigned to seemingly trivial injury categories such as open wounds and bruises and therefore pulling away disability from more serious injury outcomes including TBI and SCI. Therefore, we decided to assign one injury case to the most severe of any co-occurring injuries.

2. Severity hierarchies

In order to determine the most severe injury, a nature-of-injury severity hierarchy was applied to the data. The method for deriving the severity hierarchy is described in more detail in the methods appendix of the GBD 2016 non-fatal outcomes literature, but in summary we used follow-up data obtained from a pooled dataset of six follow-up studies from China, the Netherlands, and the US, which followed up patients for at least one year after the injury, and the Medical Expenditure Panel Survey (MEPS)[3–6], in order to synthesise different health status measures that could be mapped to GBD disability weights using survey responses that queried the relationship between SF-12 and GBD health states. Information on these studies is provided in Appendix Table 5.

Among the 47 nature of injury codes, spinal cord lesion at the neck level, spinal cord lesion below the neck level, traumatic brain injury moderate or severe, and traumatic brain injury mild were the first, fourth, 12th, and 16th ranked injuries in terms of severity. We used these nature of injury severities to estimate the resulting disability such that only the most severe injury was used for estimating YLDs. The full list of severity ranking is provided in Appendix Table 4.

Appendix Table 4: Empirically-derived nature of injury rankings

Rank	Nature of injury
1	Spinal cord lesion below neck level
2	Amputation of lower limbs, bilateral
3	Amputation of upper limbs, bilateral
4	Spinal cord lesion at neck level
5	Fracture of hip
6	Fracture of femur, other than femoral neck
7	Amputation of upper limb, unilateral
8	Amputation of lower limb, unilateral
9	Multiple fractures, dislocations, wounds, sprains, and strains
10	Moderate/severe TBI
11	Fracture of patella, tibia or fibula, or ankle
12	Crush injury
13	Nerve injury
14	Internal hemorrhage in abdomen and pelvis
15	Fracture of pelvis
16	Burns, >=20% total burned surface area or >= 10% burned surface area if head/neck or hands/wrist involved without lower airway burns
17	Complications following therapeutic procedures
18	Minor TBI
19	Dislocation of hip
20	Lower airway burns (considered inpatient-only diagnosis)
21	Fracture of skull
22	Amputation of thumb
23	Fracture of vertebral column
24	Severe chest Injury
25	Fracture of hand (wrist and other distal part of hand)
26	Amputation of toe/toes

27	Drowning and nonfatal submersion
28	Amputation of fingers (excluding thumb)
29	Contusion in any part of the body
30	Fracture of foot bones except ankle
31	Dislocation of knee
32	Effect of different environmental factors
33	Fracture of radius and/or ulna
34	Burns, <20% total burned surface area without lower airway burns
35	Fracture of face bones
36	Open wound(s)
37	Fracture of sternum and/or fracture of one or more ribs
38	Foreign body in GI and urogenital system
39	Superficial injury of any part of the body
40	Asphyxiation
41	Muscle and tendon injuries, including sprains and strains lesser dislocations
42	Injury to eyes
43	Poisoning requiring urgent care
44	Foreign body in respiratory system
45	Dislocation of shoulder
46	Foreign body in ear
47	Fracture of clavicle, scapula, or humerus

3. Estimating permanent health loss

These studies were also used to measure the probability of permanent health loss, which we defined as an injury that leads to greater disability than an individual's baseline disability one year after an injury occurred. The difference between the pre-injury health states and health status one year after injury is assumed to be their permanent level of injury-related disability. To assess the probability of permanent health loss we estimated the effects using a logit-linear mixed effects regression:

$$\begin{aligned} Logit(DW)_{im} = \ & \alpha + \beta_1(injuries_{im}) + \beta_2(never\ injured_i) + \beta_3(never\ injured_i * age_i) \\ & + \beta_4(fracture\ of\ pelvis_i) + \beta_5(fracture\ of\ pelvis_i * age_i) + \beta_6(poisoning_i * age_i) \\ & + \beta_7(moderate\ to\ severe\ TBI_i * age_i) + RE_c + RE_i \end{aligned}$$

where we included dummy variables for all the nature-of-injury categories ($injuries_{im}$), with the reference category being no injury from MEPS dataset. We also include a dummy for never injured prior to the current injury, age, interactions between age and never injured status, and interactions with three long-term nature-of-injury categories that were found to significantly vary with age: pelvis fractures, poisonings, and moderate or severe traumatic brain injuries. In notation, subscript m refers to patient-reported outcome measure, i refers to individual and c refers to country. Random effects (RE) were included to control for variation between countries and individuals.

After predicting overall disability at one-year follow-up, we estimated a counterfactual by setting all observations to "no injury," the reference group for $\beta(injuries_{im})$ in our model. The disability attributable to the nature-of-injury at one year was assumed to be the difference between our counterfactual of no injury and predicted disability with injury. The probability of treated long-term outcomes was estimated via the ratio of this attributable disability relative to the long-term disability weight for that injury.

$$Probability\ of\ longterm\ disability = \frac{with\ injury\ disability_{im} - counterfactual\ disability_{im}}{DW_m}$$

We developed estimates of the probability of permanent health loss by nature of injury category, injury severity level (including need for inpatient admission), and age. Moderate-severe TBI and spinal cord lesions only have inpatient injury long-term probabilities.

The probabilities of permanent health loss are provided below in Appendix Table 6.

Appendix Table 5: Probabilities of permanent health loss

Injury	Mild TBI		Mod. or sev. TBI	SCI
Age	Inpatient	Outpatient		
<1	0.051 (0.036-0.073)	0.006 (0.004-0.009)	0.067 (0.047-0.095)	0.987 (0.979-0.995)
1-4	0.052 (0.037-0.074)	0.006 (0.004-0.009)	0.068 (0.048-0.096)	
5-9	0.057 (0.040-0.081)	0.007 (0.005-0.010)	0.072 (0.051-0.102)	
10-14	0.064 (0.045-0.091)	0.008 (0.006-0.011)	0.078 (0.054-0.110)	
15-19	0.072 (0.050-0.102)	0.009 (0.006-0.013)	0.084 (0.059-0.118)	
20-24	0.080 (0.056-0.114)	0.010 (0.007-0.015)	0.090 (0.063-0.127)	
25-29	0.089 (0.063-0.127)	0.012 (0.008-0.017)	0.096 (0.068-0.136)	
30-34	0.100 (0.070-0.142)	0.013 (0.009-0.019)	0.104 (0.073-0.146)	
35-39	0.111 (0.078-0.158)	0.015 (0.010-0.021)	0.111 (0.078-0.157)	
40-44	0.124 (0.087-0.177)	0.017 (0.012-0.024)	0.119 (0.083-0.168)	
45-49	0.139 (0.097-0.197)	0.019 (0.013-0.027)	0.127 (0.089-0.179)	
50-54	0.155 (0.108-0.220)	0.022 (0.015-0.031)	0.136 (0.095-0.191)	
55-59	0.172 (0.121-0.245)	0.025 (0.017-0.035)	0.145 (0.101-0.204)	
60-64	0.192 (0.134-0.273)	0.028 (0.019-0.039)	0.154 (0.108-0.218)	
65-69	0.214 (0.150-0.304)	0.031 (0.022-0.045)	0.164 (0.115-0.232)	
70-74	0.238 (0.167-0.338)	0.035 (0.025-0.050)	0.174 (0.122-0.246)	
75-79	0.264 (0.185-0.375)	0.040 (0.028-0.057)	0.185 (0.130-0.261)	
80-84	0.294 (0.206-0.417)	0.045 (0.032-0.064)	0.196 (0.137-0.276)	
85-89	0.326 (0.228-0.463)	0.051 (0.036-0.072)	0.207 (0.145-0.292)	
90-94	0.362 (0.253-0.513)	0.057 (0.040-0.081)	0.218 (0.153-0.308)	
95+	0.400 (0.280-0.569)	0.064 (0.045-0.091)	0.230 (0.161-0.324)	

Appendix Table 6: Long-term follow-up studies

Dataset	Year	Type of data	Type of patients	Setting	Sample size*
**Guangdong follow-up survey, China**[7]	2006–2007	Follow-up survey among sample of ISS patients	Patients (15+ years) who were hospitalised that had been injured by road traffic[48], fall, blunt or penetrating trauma	Based on three national injury surveillance hospitals in Zhuhai, Guangdong Province in China	998 (response 87%)
**LIS follow-up survey, Netherlands**[7]	2001–2002	Follow-up survey among stratified sample of ISS patients (oversampling less common, severe injuries)	Patients (15+ years) who visited the Emergency Department of a hospital and were discharged to the home environment and patients who were admitted to hospital	Based on 17 public hospitals in the Netherlands	8,564 (response 37%)
**LIS follow-up survey, Netherlands**[8]	2007–2008	Follow-up survey among stratified sample of ISS patients (oversampling less common, severe injuries)	Patients (15+ years) who visited the Emergency Department of a hospital and were discharged to the home environment and patients who were admitted to hospital	Based on 15 public hospitals in the Netherlands	8,057 (response 36%)
**NSCOT – National study on Costs and Outcomes of Trauma, USA**[9]	2001–2002	A prospective cohort study was conducted among a sample of adult trauma patients treated at Level I trauma centers and non-trauma center hospitals	Patients treated for a moderate to severe injury (as defined by at least one injury of an Abbreviated Injury Scale (AIS) score of 3 or greater	Based on 69 hospitals in 12 states in the US	5,191 (response 61%)
**SCTBIFR – South Carolina Traumatic Brain injury Follow-up Registry, USA**[10]	1999–2002	A prospective cohort study was conducted among injured inpatients with a traumatic brain injury-related injury	Patients (15+ years) who were admitted to hospitals and met the CDC case definition of TBI – trauma to the head associated with altered consciousness, amnesia, neurological abnormalities, skull fracture, intracranial lesion, or death	Discharged from all nonfederal in-state acute care hospitals	7,613 (response 28%)
**Burns outcome study, Netherlands**[11]	2003–2006	A multicenter prospective cohort was conducted among adult (severe) burn patients	Injury patients who sustained severe burns	Three public hospitals with specialised burn units.	311 (response 78%)

4. Converting incidence to prevalence

Short-term and long-term prevalence estimates were modelled separately for computational efficiency and to allow a proportion of cases to undergo adjustment for the increased mortality rate expected in this patient population.

Short-term cases

For short-term prevalence estimation, we converted incidence to prevalence by multiplying incidence and the expected duration of a short-term case. Duration of treated short-term cases was estimated using the following method. We analysed patient responses of two Dutch Injury Surveillance System follow-up studies of 2001–2003 and 2007–2008[7,8]. These studies collected data at 2.5, 5, 9, and 12 months post-injury on whether injury patients were still experiencing problems

due to their injury. If not, the patients were asked how many days they had experienced problems. The injury patients that still reported having problems one year after the injury were assumed to be captured in our analysis of permanent disability. The duration for treated cases of short-term injury was estimated for injuries warranting inpatient admission and injuries warranting other health care separately. The estimates were supplemented by expert-driven estimates of short-term duration for nature of injury categories that did not appear in the Dutch dataset and untreated injuries.

Long-term cases

For long-term prevalence, we first estimated the increased mortality expected in individuals with a TBI or SCI using standardised mortality ratios (SMRs) based on literature review[12–25]. These SMRs were measured in a meta-analysis and then converted to excess mortality rates and applied to the prevalence estimation in the ordinary differential equation solver that converts incidence to prevalence. The results from the meta-analysis and the corresponding SMRs are provided in Appendix Table 7. Short-term prevalence rates did not require the same adjustment for mortality.

Appendix Table 7: Standardised mortality ratios for TBI and SCI

		SMR
Mild TBI		No excess mortality
Mod. or sev. TBI		2.18 (1.88-2.52)
SCI at neck	Below 60yrs	5.03 (4.51-5.60)
	Above 60yrs	2.48 (1.58-3.87)
SCI below neck	Below 60yrs	2.72 (2.45-3.03)
	Above 60yrs	1.89 (1.23-2.89)

5. Adjusting for health care access

For both prevalence categories (short-term and long-term), we calculated different disability weights for treated versus untreated injuries using the Healthcare Access and Quality (HAQ) index. Location-years with an index greater than or equal to 75 were assumed to have all injuries treated, and location-years below this were scaled between 10% and 100% treated based on their HAQ index. The specific formula used is as follows:

$$Percent\ treated = 0.1 + 0.9 \times \frac{HAQi_{country,year} - \min\,(HAQi)}{\max(HAQi) - \min\,(HAQi)}$$

6. Calculating years lived with disability

We then multiplied short-term and long-term prevalence of each cause-nature pair by these disability weights to get years lived with disability (YLDs) and then added the YLDs to get total burden due to injuries. The final disability weights stem from lay descriptions of sequelae highlighting the major functional consequences and symptoms and vary by duration, treatment, and condition. The health states are as follows:

Minor TBI (concussion): has headaches, dizziness, nausea and difficulty concentrating.

Traumatic brain injury, long-term consequences, minor (with or without treatment): has episodes of headaches, memory problems, and difficulty concentrating.

Traumatic brain injury, long-term consequences, moderate (with or without treatment): has frequent headaches, memory problems, difficulty concentrating, and dizziness. The person is often anxious and moody.

Traumatic brain injury, long-term consequences, severe (with or without treatment): cannot think clearly and has frequent headaches, memory problems, difficulty concentrating and dizziness. The person is often anxious and moody, and depends on others for feeding, toileting, dressing and walking.

Severe traumatic brain injury, short term (with or without treatment): cannot concentrate and has headaches, memory problems, dizziness, and feels angry.

Spinal cord lesion below neck level (treated): is paralyzed from the waist down, cannot feel or move the legs and has difficulties with urine and bowel control. The person uses a wheelchair to move around.

Spinal cord lesion below neck level (untreated): is paralyzed from the waist down, cannot feel or move the legs and has difficulties with urine and bowel control. Legs are in fixed, bent positions, and the person gets frequent infections and pressure sores.

Spinal cord lesion at neck level (treated): is paralyzed from the neck down, with no feeling or control over any part of the body below the neck, and no urine or bowel control.

Spinal cord lesion at neck level (untreated): is paralyzed from the neck down, with no feeling or control over any part of the body below the neck, and no urine or bowel control. Arms and legs are in fixed, bent positions, and the person gets frequent infections and pressure sores.

The disability weights by severity and duration are provided in Appendix Table 8.

Appendix Table 8: Disability weights by condition, duration, and Frankel-ASIA scale for SCI

Condition	Short-term	Long-term		
Mild TBI	0.110 (0.074-0.158)	0.132 (0.090-0.182)		
Mod. or sev. TBI	0.214 (0.141-0.297)	0.164 (0.112-0.226)		
SCI at neck	0.732 (0.544-0.871)	Treated	A	0.589 (0.415-0.748)
			B	0.463 (0.308-0.615)
			C	0.463 (0.308-0.615)
			D	0.061 (0.040-0.089)
		Untreated	A	0.732 (0.544-0.871)
			B	0.682 (0.497-0.826)
			C	0.682 (0.497-0.826)
			D	0.061 (0.040-0.089)
SCI below neck	0.623 (0.434-0.777)	Treated	A	0.296 (0.198-0.414)
			B	0.057 (0.037-0.085)
			C	0.057 (0.037-0.085)
			D	0.061 (0.040-0.089)

		Untreated	A	0.623 (0.434-0.777)
			B	0.460 (0.310-0.605)
			C	0.460 (0.310-0.605)
			D	0.061 (0.040-0.089)

1 Vos T, Abajobir AA, Abate KH, *et al.* Global, regional, and national incidence, prevalence, and years lived with disability for 328 diseases and injuries for 195 countries, 1990–2016: a systematic analysis for the Global Burden of Disease Study 2016. *The Lancet* 2017; **390**: 1211–59.

2 Vos T, Flaxman AD, Naghavi M, *et al.* Years lived with disability (YLDs) for 1160 sequelae of 289 diseases and injuries 1990–2010: a systematic analysis for the Global Burden of Disease Study 2010. *The Lancet* 2012; **380**: 2163–96.

3 Salomon JA, Haagsma JA, Davis A, *et al.* Disability weights for the Global Burden of Disease 2013 study. *The Lancet Global Health* 2015; **3**: e712–23.

4 NIAAA Publications. https://pubs.niaaa.nih.gov/publications/arh29-2/74-78.htm (accessed May 15, 2018).

5 Medical Expenditure Panel Survey Home. https://meps.ahrq.gov/mepsweb/ (accessed May 15, 2018).

6 Disability weights for diseases in the Netherlands. Rotterdam: Erasmus University of Rotterdam, 2002.

7 China Zhuhai Study 2006-2007 - China CDC | GHDx. http://ghdx.healthdata.org/record/china-zhuhai-study-2006-2007-china-cdc (accessed May 15, 2018).

8 Functional outcome at 2.5, 5, 9, and 24 months after injury in the Netherlands | GHDx. http://ghdx.healthdata.org/record/functional-outcome-25-5-9-and-24-months-after-injury-netherlands (accessed May 15, 2018).

9 Netherlands Injury Surveillance System 2007 | GHDx. http://ghdx.healthdata.org/record/netherlands-injury-surveillance-system-2007 (accessed May 15, 2018).

10 Mackenzie EJ, Rivara FP, Jurkovich GJ, *et al.* The National Study on Costs and Outcomes of Trauma. *J Trauma* 2007; **63**: S54-67; discussion S81-86.

11 CDC, Medical University of South Carolina, South Carolina Department of Disabilities and Special Needs, South Carolina Department of Health and Environmental Control. South Carolina Traumatic Brain Injury Follow-up Registry 1999-2013. USA.

12 Health-Related Quality of Life After Burns: A Prospective Multicentre Cohort Study With 18 Months Follow-Up | GHDx. http://ghdx.healthdata.org/record/health-related-quality-life-after-burns-prospective-multicentre-cohort-study-18-months-follow (accessed May 15, 2018).

13 Strauss D, Shavelle R, DeVivo MJ, Day S. An analytic method for longitudinal mortality studies. *J Insur Med* 2000; **32**: 217–25.

14 Shavelle R, Strauss D. Comparative mortality of adults with traumatic brain injury in California, 1988--97. *J Insur Med* 2000; **32**: 163–6.

15 Baguley IJ, Nott MT, Howle AA, *et al.* Late mortality after severe traumatic brain injury in New South Wales: a multicentre study. *Med J Aust* 2012; **196**: 40–5.

16 Middleton JW, Dayton A, Walsh J, Rutkowski SB, Leong G, Duong S. Life expectancy after spinal cord injury: a 50-year study. *Spinal Cord* 2012; **50**: 803–11.

17 Middleton JW, Dayton A, Walsh J, Rutkowski SB, Leong G, Duong S. Life expectancy after spinal cord injury: a 50-year study. *Spinal Cord* 2012; **50**: 803–11.

18 Brooks JC, Strauss DJ, Shavelle RM, Paculdo DR, Hammond FM, Harrison-Felix CL. Long-term disability and survival in traumatic brain injury: results from the National Institute on Disability and Rehabilitation Research Model Systems. *Arch Phys Med Rehabil* 2013; **94**: 2203–9.

19 Baguley I, Slewa-Younan S, Lazarus R, Green A. Long-term mortality trends in patients with traumatic brain injury. *Brain Inj* 2000; **14**: 505–12.

20 Ratcliff G, Colantonio A, Escobar M, Chase S, Vernich L. Long-term survival following traumatic brain injury. *Disabil Rehabil* 2005; **27**: 305–14.

21 Frankel HL, Coll JR, Charlifue SW, *et al.* Long-term survival in spinal cord injury: a fifty year investigation. *Spinal Cord* 1998; **36**: 266–74.

22 Harrison-Felix CL, Whiteneck GG, Jha A, DeVivo MJ, Hammond FM, Hart DM. Mortality over four decades after traumatic brain injury rehabilitation: a retrospective cohort study. *Arch Phys Med Rehabil* 2009; **90**: 1506–13.

23 Moorin R, Miller TR, Hendrie D. Population-based incidence and 5-year survival for hospital-admitted traumatic brain and spinal cord injury, Western Australia, 2003-2008. *J Neurol* 2014; **261**: 1726–34.

24 Colantonio A, Escobar MD, Chipman M, *et al.* Predictors of postacute mortality following traumatic brain injury in a seriously injured population. *J Trauma* 2008; **64**: 876–82.

25 Flaada JT, Leibson CL, Mandrekar JN, *et al.* Relative risk of mortality after traumatic brain injury: a population-based study of the role of age and injury severity. *J Neurotrauma* 2007; **24**: 435–45.

26 Cameron CM, Purdie DM, Kliewer EV, McClure RJ. Ten-year outcomes following traumatic brain injury: a population-based cohort. *Brain Inj* 2008; **22**: 437–49.

27 Marino RJ, Ditunno JF, Donovan WH, Maynard F. Neurologic recovery after traumatic spinal cord injury: data from the Model Spinal Cord Injury Systems. *Arch Phys Med Rehabil* 1999; **80**: 1391–6.

Appendix 1, Section 2

TBI/SCI Flow Chart

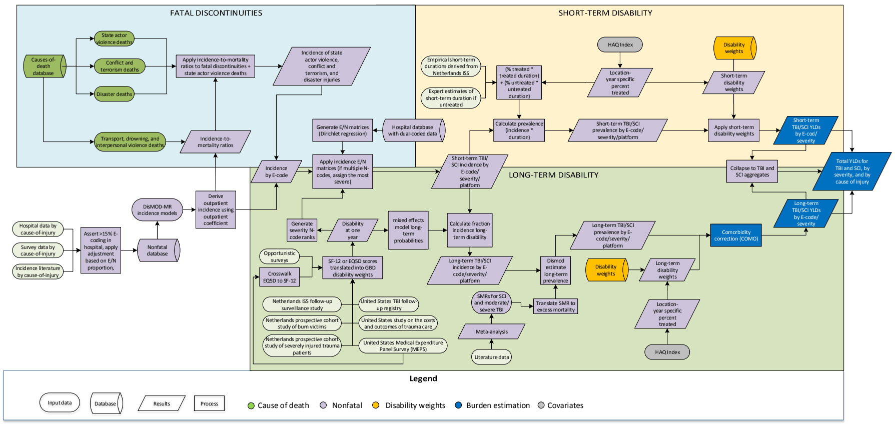

Appendix 1, Section 3

Summary of General Global Burden of Disease Study Methods

The Institute for Health Metrics and Evaluation with a growing collaboration of scientists produces annual updates of the Global Burden of Disease study. Estimates span the period from 1990 to the most recent completed year. By the time of the release of GBD 2016 in September 2017, there were over 2,700 collaborators in 132 countries who contributed to this global public good. Annual updates allow incorporation of new data and method improvements to ensure that the most up-to-date information is available to policy makers in a timely fashion to help make resource allocation decisions. In this analysis, we have aggregated results from GBD 2016 for 15 disease and injury outcomes that are generally cared for by neurological services. These include infectious conditions (tetanus, meningitis, encephalitis), stroke, brain and other nervous system cancers, traumatic brain injury, and spinal cord lesion which are classified outside the more narrowly defined category of neurological disorders in GBD (ie, Alzheimer's disease and other dementias, Parkinson's disease, multiple sclerosis, motor neuron disease, idiopathic epilepsy, migraine, tension-type headache, and a rest category of less common other neurological disorders). Compared to a previous analysis based on GBD 2015,[1] we were able to add the non-fatal outcomes of traumatic brain injury and spinal cord lesion, and medication overuse headache is no longer included as a separate cause but quantified as a consequence of the underlying headache types.

In the methods section of this overview paper we present a summary of the general methods of the global burden of disease. In the accompanying disease-specific papers we concentrate on methods that are specific to each disorder. The guiding principle of GBD is to assess health loss due to mortality and disability comprehensively, where we define disability as any departure from full health. In GBD 2016, estimates were made for 195 countries and territories, and 579 subnational locations, for 27 years starting from 1990, for 23 age groups and both sexes. Deaths were estimated for 264 diseases and injuries, while prevalence and incidence were estimated for 328 diseases and injuries. In order to allow meaningful comparisons between deaths and non-fatal disease outcomes as well as between diseases, the data on deaths and prevalence are summarised in a single indicator, the disability-adjusted life-year (DALY). DALYs are the sum of years of life lost (YLLs) and years lived with disability (YLDs). YLLs are estimated as the multiplication of counts of death and a standard, "ideal", remaining life expectancy at the age of death. The standard life expectancy is derived from the lowest observed mortality rates in any population in the world greater than 5 million.[2] YLDs are estimated as the product of prevalence of individual consequences of disease (or "sequelae") times a disability weight that quantifies the relative severity of a sequela as a number between zero (representing "full health") and 1 (representing death). Disability weights have been estimated in nine population surveys and an open-access internet survey in which respondents are asked to choose the "healthier"[3] between random pairs of health states that are presented with a short description of the main features.

All-cause mortality rates are estimated from vital registration data in countries with complete coverage. For other countries, the probabilities of death before age 5 and between ages 15 and 60 are estimated from censuses and surveys asking mothers to provide a history of children ever born and those still alive, and surveys asking adults about siblings who are alive or have passed away. Using model life tables, these probabilities of death are transformed into age-specific death rates by location, year, and sex. GBD has collated a large database of cause of death data from vital registrations and verbal autopsy surveys in which relatives are asked a standard set of questions to ascertain the likely cause of death, supplemented with police and mortuary data for injury deaths in countries with no other data. For countries with vital registration data, the completeness is assessed with demographic methods based on comparing recorded deaths with population counts between

two successive censuses. The cause of death information is provided in a large number of different classification systems based on versions of the International Classification of Diseases or bespoke classifications in some countries. All data are mapped into the disease and injury categories of GBD. All classification systems contain codes that are less informative because they lack a specific diagnosis (eg, unspecified cancer) or refer to codes that cannot be underlying cause of death (eg, low back pain or senility) or are intermediate causes (eg, heart failure or sepsis). Such deaths are redistributed to more precise underlying causes of death.[4] After these redistributions and corrections for under-registration, the data are analysed in CODEm (cause of death ensemble model), a highly systematised tool that runs many different models on the same data and chooses an ensemble of models that best reflects all the available input data. Models are chosen with variations in the statistical approach ("mixed effects" of spatiotemporal Gaussian Process Regression), in the unit of analysis (rates or cause fractions), and the choice of predictive covariates. The statistical performance of all models is tested by holding out 30% of the data and checking how well a model covers the data that were held out. To enforce consistency from CODEm, the sum of all cause-specific mortality rates is scaled to that of the all-cause mortality rates in each age, sex, location, and year category.

Non-fatal estimates are based on systematic reviews of published papers and unpublished documents, survey microdata, administrative records of health encounters, registries, and disease surveillance systems. Our Global Health Data Exchange (GHDx, http://ghdx.healthdata.org/) is the largest repository of health data globally. We first set a reference case definition and/or study method that best quantifies each disease or injury or consequence thereof. If there is evidence of a systematic bias in data that used different case definitions or methods compared to reference data we adjust those data points to reflect what its value would have been if measured as the reference. This is a necessary step if one wants to use all data pertaining to a particular quantity of interest rather than choosing a small subset of data of the highest quality only. DisMod-MR 2.1, a Bayesian meta-regression tool, is our main method of analyzing non-fatal data. It is designed as a geographical cascade where a first model is run on all the world's data, which produces an initial global fit and estimates coefficients for predictor variables and the adjustments for alternative study characteristics. The global fit adjusted by the values of random effects for each of seven GBD super-regions, the coefficients on sex and country predictors, are passed down as data to a model for each super-region together with the input data for that geography. The same steps are repeated going from super-region to 21 region fits and then to 195 fits by country and where applicable a further level down to subnational units. Below the global fit, all models are run separately by sex and for six time periods: 1990, 1995, 2000, 2005, 2010, and 2016. During each fit all data on prevalence, incidence, remission (ie, cure rate) and mortality are forced to be internally consistent. For most diseases, the bulk of data on prevalence or incidence is at the disease level with fewer studies providing data on the proportions of cases of disease in each of the sequelae defined for the disease. The proportions in each sequela are pooled using DisMod-MR 2.1 or meta-analysis, or derived from analyses of patient-level datasets. The multiplication of prevalent cases for each disease sequela and the appropriate disability weight produces YLD estimates that do not yet take into account comorbidity. To correct for comorbidity, these data are used in a simulation to create hypothetical individuals in each age, sex, location, and year combination who experience no, one, or multiple sequelae simultaneously. We assume that disability weights are multiplicative rather than additive as this avoids assigning a combined disability weight value in any individual to exceed 1, ie, be worse than a "year lost due to death". This comorbidity adjustment leads to an average scaling down of disease-specific YLDs ranging from about 2% in young children up to 17% in oldest ages.

All our estimates of causes of death are categorical: each death is assigned to a single underlying cause. This has the attractive property that all estimates add to 100%. For risks, we use a different,

“counterfactual” approach, ie, answering the question: “what would the burden have been if the population had been exposed to a theoretical minimum level of exposure to a risk”. Thus, we need to define what level of exposure to a risk factor leads to the lowest amount of disease. We then analyse data on the prevalence of exposure to a risk and derive relative risks for any risk-outcome pair for which we find sufficient evidence of a causal relationship. Prevalence of exposure is estimated in DisMod-MR 2.1, using spatiotemporal Gaussian Process Regression, or from satellite imagery in the case of ambient air pollution. Relative risk data are pooled using meta-analysis of cohort, case-control and/or intervention studies. For each risk and outcome pair, we evaluate the evidence and judge if the evidence falls into the categories of “convincing” or “probable” as defined by the World Cancer Research Fund.[5] From the prevalence and relative risk results, population attributable fractions are estimated relative to the theoretical minimum risk exposure level (TMREL). When we aggregate estimates for clusters of risks, eg, metabolic or behavioural risks, we use a multiplicative function rather than simple addition and take into account how much of each risk is mediated through another risk. For instance, some of the risk of high body mass index is directly onto stroke as an outcome but much of its impact is mediated through high blood pressure, high cholesterol, or high fasting plasma glucose, and we would not want to double count the mediated effects when we estimate aggregates across risk factors.[6]

Uncertainty is propagated throughout all these calculations by creating 1,000 values for each prevalence, death, YLL, YLD, or DALY estimate and performing aggregations across causes and locations at the level of each of the 1,000 values for all intermediate steps in the calculation. The lower and upper bounds of the 95% uncertainty interval are the 25th and 975th values of the ordered 1,000 values. For all age-standardised rates, GBD uses a standard population calculated as the non-weighted average across all countries of the percentage of the population in each five-year age group for the years 2010 to 2035 from the United Nations Population Division’s World Population Prospects (2012 revision).[7,8]

GBD uses a composite indicator or sociodemographic development, SDI, which reflects the geometric mean of normalised values of a location’s income per capita, the average years of schooling in the population 15 and over, and the total fertility rate. Countries and territories are grouped into five quintiles of high, high-middle, middle, low-middle, and low SDI based on their 2016 values.[2]

References

1 GBD 2015 Neurological Disorders Collaborator Group. Global, regional, and national burden of neurological disorders during 1990-2015: a systematic analysis for the Global Burden of Disease Study 2015. *Lancet Neurol* 2017; **16**: 877–97.

2 GBD 2016 Mortality Collaborators. Global, regional, and national under-5 mortality, adult mortality, age-specific mortality, and life expectancy, 1970-2016: a systematic analysis for the Global Burden of Disease Study 2016. *Lancet Lond Engl* 2017; **390**: 1084–150.

3 Salomon JA, Haagsma JA, Davis A, *et al.* Disability weights for the Global Burden of Disease 2013 study. *Lancet Glob Health* 2015; **3**: e712-723.

4 GBD 2016 Causes of Death Collaborators. Global, regional, and national age-sex specific mortality for 264 causes of death, 1980-2016: a systematic analysis for the Global Burden of Disease Study 2016. *Lancet Lond Engl* 2017; **390**: 1151–210.

5 American Institute for Cancer Research. Food, nutrition, physical activity, and the prevention of cancer: a global perspective. Washington, DC: American Institute for Cancer Research, 2007.

6 GBD 2016 Risk Factors Collaborators. Global, regional, and national comparative risk assessment of 84 behavioural, environmental and occupational, and metabolic risks or clusters of risks, 1990-2016: a systematic analysis for the Global Burden of Disease Study 2016. *Lancet Lond Engl* 2017; **390**: 1345–422.

7 GBD 2013 Mortality and Causes of Death Collaborators. Global, regional, and national age-sex specific all-cause and cause-specific mortality for 240 causes of death, 1990-2013: a systematic analysis for the Global Burden of Disease Study 2013. *Lancet Lond Engl* 2015; **385**: 117–71.

8 United Nations Department of Economics and Social Affairs Population Division. World Population Prospects: The 2012 Revision. http://esa.un.org/unpd/wpp/Documentation/publications.htm (accessed Nov 4, 2014).

Appendix 1, Section 4

GATHER checklist of information that should be included in reports of global health estimates, with description of compliance and location of information for GBD 2016.

#	GATHER checklist item	Description of compliance	Reference
**Objectives and funding**			
1	Define the indicators, populations, and time periods for which estimates were made.	Narrative provided in paper and appendix describing indicators, definitions, and populations	Main text (Methods) and appendix
2	List the funding sources for the work.	Funding sources listed in paper	Summary (Funding)
**Data Inputs**			
For all data inputs from multiple sources that are synthesised as part of the study:			
3	Describe how the data were identified and how the data were accessed.	Narrative description of data seeking methods provided	Main text (Methods) and appendix
4	Specify the inclusion and exclusion criteria. Identify all ad-hoc exclusions.	Narrative about inclusion and exclusion criteria by data type provided; ad hoc exclusions in cause-specific write-ups	Main text (Methods) and appendix
5	Provide information on all included data sources and their main characteristics. For each data source used, report reference information or contact name/institution, population represented, data collection method, year(s) of data collection, sex and age range, diagnostic criteria or measurement method, and sample size, as relevant.	An interactive, online data source tool that provides metadata for data sources by component, geography, cause, risk, or impairment has been developed	Online data citation tools: http://ghdx.healthdata.org/gbd-2016
6	Identify and describe any categories of input data that have potentially important biases (e.g., based on characteristics listed in item 5).	Summary of known biases by cause included in appendix	Appendix
For data inputs that contribute to the analysis but were not synthesised as part of the study:			

7	Describe and give sources for any other data inputs.	Included in online data source tool	http://ghdx.healthdata.org/gbd-2016
For all data inputs:			
8	Provide all data inputs in a file format from which data can be efficiently extracted (e.g., a spreadsheet as opposed to a PDF), including all relevant meta-data listed in item 5. For any data inputs that cannot be shared due to ethical or legal reasons, such as third-party ownership, provide a contact name or the name of the institution that retains the right to the data.	Downloads of input data available through online tools, including data visualisation tools and data query tools; input data not available in tools will be made available upon request	Online data visualisation tools, data query tools, and the Global Health Data Exchange
**Data analysis**			
9	Provide a conceptual overview of the data analysis method. A diagram may be helpful.	Flow diagrams of the overall methodological processes, as well as cause-specific modelling processes, have been provided	Main text (Methods) and appendix
10	Provide a detailed description of all steps of the analysis, including mathematical formulae. This description should cover, as relevant, data cleaning, data pre-processing, data adjustments and weighting of data sources, and mathematical or statistical model(s).	Flow diagrams and corresponding methodological write-ups for each cause, as well as the databases and modelling processes, have been provided	Main text (Methods) and appendix
11	Describe how candidate models were evaluated and how the final model(s) were selected.	Provided in the methodological write-ups	Appendix
12	Provide the results of an evaluation of model performance, if done, as well as the results of any relevant sensitivity analysis.	Provided in the methodological write-ups	Appendix
13	Describe methods for calculating uncertainty of the estimates. State which sources of uncertainty were, and were not, accounted for in the uncertainty analysis.	Appendix	Appendix
14	State how analytic or statistical source code used to generate estimates can be accessed.	Appendix	http://ghdx.healthdata.org/gbd-2016-code
**Results and Discussion**			

15	Provide published estimates in a file format from which data can be efficiently extracted.	GBD 2016 results are available through online data visualisation tools, the Global Health Data Exchange, and the online data query tool	Main text, and online data tools (data visualisation tools, data query tools, and the Global Health Data Exchange)
16	Report a quantitative measure of the uncertainty of the estimates (e.g. uncertainty intervals).	Uncertainty intervals are provided with all results	Main text, appendix, and online data tools (data visualisation tools, data query tools, and the Global Health Data Exchange)
17	Interpret results in light of existing evidence. If updating a previous set of estimates, describe the reasons for changes in estimates.	Discussion of methodological changes between GBD rounds provided in the narrative of the manuscript and appendix	Main text (Methods and Discussion) and appendix
18	Discuss limitations of the estimates. Include a discussion of any modelling assumptions or data limitations that affect interpretation of the estimates.	Discussion of limitations provided in the narrative of the main paper, as well as in the methodological write-ups in the appendix	Main text (Limitations) and appendix
