# Supplementary material for: Global, regional, and national burden of traumatic brain injury and spinal cord injury, 1990–2016: a systematic analysis for the Global Burden of Disease Study 2016
Source: Lancet Neurol. 2019 Jan;18(1):56–87. doi: 10.1016/S1474-4422(18)30415-0 (PMC6291456; doi:10.1016/S1474-4422(18)30415-0)
Supplement: Supplementary appendix2 [file mmc2.pdf]

THE LANCET
Neurology

Supplementary appendix 2

This appendix formed part of the original submission and has been peer reviewed. We post it as supplied by the authors.

Supplement to: GBD 2016 Traumatic Brain Injury and Spinal Cord Injury Collaborators. Global, regional, and national burden of traumatic brain injury and spinal cord injury, 1990 to 2016: a systematic analysis for the Global Burden of Disease Study 2016. *Lancet Neurol* 2018; published online Nov 26. http://dx.doi.org/10.1016/S1474-4422(18)30415-0.

Appendix Table 1: Incidence, prevalence, and YLDs for 2016 and percentage change of age-standardized rates by location for TBI, males

Location	Incidence (95% UI)			Prevalence (95% UI)			YLDs (95% UI)		
	2016 counts	2016 age-standardised rates per 100,000	Percentage change in age-standardised rates between 1990 and 2016	2016 counts	2016 age-standardised rates per 100,000	Percentage change in age-standardised rates between 1990 and 2016	2016 counts	2016 age-standardised rates per 100,000	Percentage change in age-standardised rates between 1990 and 2016
**Global**	**17 465 449<br>(15 856 986 to 19 228 916)**	**471<br>(427 to 520)**	**4.4<br>(2.4 to 6.4)**	**34 778 162<br>(33 469 326 to 36 112 919)**	**959<br>(923 to 995)**	**9.7<br>(8.8 to 10.5)**	**5 126 307<br>(3 785 215 to 6 577 779)**	**141<br>(103 to 179)**	**9.9<br>(8.8 to 10.8)**
**High SDI**	**2 183 703<br>(1 884 070 to 2 552 912)**	**428<br>(370 to 498)**	**-10.4<br>(-13.3 to -7.2)**	**4 941 166<br>(4 739 105 to 5 142 046)**	**799<br>(765 to 834)**	**-8.4<br>(-9.2 to -7.6)**	**717 969<br>(545 586 to 895 610)**	**118<br>(90 to 148)**	**-8.1<br>(-8.3 to -7.7)**
**High-middle SDI**	**3 578 546<br>(3 237 184 to 3 970 220)**	**598<br>(540 to 664)**	**-12.9<br>(-15.4 to -10.4)**	**8 268 323<br>(7 954 731 to 8 604 337)**	**1 290<br>(1 242 to 1 342)**	**-7.5<br>(-8.6 to -6.4)**	**1 255 251<br>(911 680 to 1 626 220)**	**197<br>(143 to 246)**	**-5.6<br>(-6.7 to -4.9)**
**Middle SDI**	**4 807 736<br>(4 367 618 to 5 281 472)**	**411<br>(374 to 450)**	**23.7<br>(20.2 to 27.2)**	**10 754 385<br>(10 338 024 to 11 154 689)**	**893<br>(859 to 926)**	**35.6<br>(34.1 to 37.2)**	**1 618 791<br>(1 206 335 to 2 046 672)**	**135<br>(99 to 171)**	**33.1<br>(32.5 to 33.6)**
**Low-middle SDI**	**5 259 072<br>(4 749 126 to 5 792 194)**	**504<br>(455 to 556)**	**14.1<br>(10.4 to 19.5)**	**8 664 124<br>(8 306 326 to 9 032 014)**	**951<br>(914 to 990)**	**21.1<br>(19.8 to 22.5)**	**1 306 517<br>(941 302 to 1 647 426)**	**142<br>(107 to 176)**	**19.0<br>(18.3 to 19.5)**
**Low SDI**	**1 704 906<br>(1 518 185 to 1 938 646)**	**480<br>(430 to 539)**	**-9.6<br>(-15.0 to -6.0)**	**2 267 431<br>(2 141 406 to 2 431 723)**	**885<br>(836 to 950)**	**3.3<br>(1.8 to 4.8)**	**347 437<br>(260 249 to 431 892)**	**132<br>(100 to 165)**	**3.9<br>(3.2 to 4.4)**
**High-income**	**1 922 381<br>(1 641 770 to 2 290 662)**	**367<br>(314 to 434)**	**-10.7<br>(-14.0 to -7.1)**	**4 227 439<br>(4 040 591 to 4 407 970)**	**664<br>(634 to 694)**	**-10.6<br>(-11.5 to -9.6)**	**598 227<br>(460 889 to 733 263)**	**95<br>(71 to 120)**	**-10.6<br>(-11.2 to -9.7)**
**High-income North America**	**719 773<br>(615 277 to 848 653)**	**403<br>(347 to 473)**	**-5.7<br>(-9.7 to -1.3)**	**1 537 299<br>(1 467 814 to 1 610 255)**	**744<br>(710 to 780)**	**-6.1<br>(-7.6 to -4.6)**	**218 722<br>(168 677 to 273 375)**	**107<br>(84 to 133)**	**-6.7<br>(-7.2 to -6.2)**
Canada	64 454<br>(54 714 to 76 701)	368<br>(314 to 436)	-13.0<br>(-18.5 to -7.6)	148 983<br>(141 914 to 156 141)	681<br>(648 to 715)	-13.5<br>(-15.6 to -11.1)	21 126<br>(16 323 to 26 086)	98<br>(76 to 122)	-12.5<br>(-13.9 to -11.0)
Greenland	99<br>(83 to 119)	366<br>(309 to 440)	-21.8<br>(-24.8 to -18.8)	176<br>(169 to 186)	591<br>(566 to 623)	-17.0<br>(-18.8 to -15.2)	25<br>(19 to 31)	83<br>(63 to 102)	-14.9<br>(-17.9 to -12.9)
United States	654 969<br>(560 823 to 772 098)	408<br>(350 to 479)	-4.7<br>(-8.9 to 0.0)	1 387 587<br>(1 324 678 to 1 453 685)	752<br>(718 to 789)	-5.2<br>(-6.8 to -3.5)	197 489<br>(150 055 to 242 420)	108<br>(81 to 133)	-6.0<br>(-6.4 to -5.4)
**Australasia**	**47 204<br>(40 060 to 55 682)**	**339<br>(289 to 399)**	**-15.1<br>(-21.5 to -8.6)**	**106 622<br>(101 549 to 111 800)**	**649<br>(617 to 681)**	**-16.0<br>(-17.9 to -13.7)**	**15 303<br>(11 725 to 18 977)**	**94<br>(71 to 115)**	**-14.8<br>(-16.2 to -13.2)**
Australia	39 690<br>(33 580 to 46 828)	337<br>(288 to 398)	-14.2<br>(-21.2 to -7.0)	89 704<br>(85 372 to 94 104)	646<br>(614 to 678)	-15.1<br>(-17.2 to -12.8)	12 874<br>(9 958 to 15 758)	94<br>(73 to 113)	-14.0<br>(-15.3 to -12.2)
New Zealand	7 514<br>(6 402 to 8 887)	347<br>(296 to 408)	-19.3<br>(-26.7 to -12.0)	16 919<br>(16 093 to 17 756)	666<br>(632 to 700)	-20.0<br>(-22.7 to -17.3)	2 429<br>(1 860 to 3 031)	97<br>(75 to 118)	-18.8<br>(-20.4 to -16.4)
**High-income Asia-Pacific**	**357 216<br>(302 499 to 429 496)**	**362<br>(309 to 429)**	**-21.8<br>(-25.9 to -17.4)**	**745 662<br>(712 651 to 777 040)**	**619<br>(589 to 646)**	**-20.7<br>(-22.3 to -19.0)**	**103 831<br>(78 960 to 127 842)**	**88<br>(67 to 111)**	**-20.5<br>(-21.6 to -19.4)**
Brunei	1 060<br>(904 to 1 251)	523<br>(446 to 620)	-19.2<br>(-24.1 to -14.2)	1 832<br>(1 725 to 1 934)	890<br>(842 to 937)	-19.8<br>(-21.8 to -18.1)	261<br>(197 to 326)	126<br>(94 to 158)	-19.1<br>(-19.8 to -18.5)
Japan	240 235<br>(201 884 to 290 437)	340<br>(288 to 402)	-21.8<br>(-26.7 to -16.6)	519 536<br>(497 004 to 540 793)	591<br>(563 to 617)	-21.6<br>(-23.5 to -19.4)	72 115<br>(55 542 to 90 198)	85<br>(66 to 105)	-21.2<br>(-22.3 to -19.9)
Singapore	7 158<br>(6 054 to 8 523)	379<br>(320 to 452)	-10.2<br>(-16.3 to -4.7)	14 988<br>(14 235 to 15 710)	666<br>(633 to 699)	-7.9<br>(-10.4 to -5.3)	2 122<br>(1 634 to 2 593)	95<br>(72 to 117)	-6.9<br>(-8.4 to -5.7)
South Korea	108 763<br>(93 013 to 129 092)	433<br>(369 to 513)	-20.8<br>(-26.3 to -15.3)	209 306<br>(199 372 to 218 543)	706<br>(671 to 738)	-20.0<br>(-21.7 to -18.3)	29 331<br>(22 123 to 35 775)	100<br>(75 to 124)	-20.4<br>(-20.8 to -19.6)
**Western Europe**	**701 636<br>(593 427 to 845 764)**	**347<br>(293 to 413)**	**-13.5<br>(-17.5 to -9.6)**	**1 665 520<br>(1 586 490 to 1 737 880)**	**639<br>(607 to 668)**	**-12.5<br>(-13.6 to -11.4)**	**236 342<br>(175 820 to 293 996)**	**92<br>(69 to 114)**	**-11.8<br>(-12.2 to -11.3)**
Andorra	134<br>(112 to 163)	359<br>(300 to 431)	2.5<br>(-1.5 to 6.5)	321<br>(305 to 337)	660<br>(624 to 694)	3.8<br>(1.7 to 6.0)	45<br>(34 to 56)	94<br>(69 to 118)	4.7<br>(3.8 to 5.3)
Austria	16 172<br>(13 507 to 19 874)	388<br>(324 to 470)	-21.2<br>(-26.6 to -15.3)	37 212<br>(35 428 to 38 870)	695<br>(661 to 729)	-19.2<br>(-21.3 to -17.2)	5 251<br>(3 966 to 6 553)	99<br>(75 to 126)	-18.0<br>(-18.3 to -17.8)
Belgium	22 421<br>(18 905 to 27 047)	405<br>(344 to 479)	-5.2<br>(-12.3 to 1.9)	49 825<br>(47 471 to 52 158)	725<br>(690 to 761)	-7.0<br>(-9.1 to -4.8)	7 075<br>(5 478 to 8 696)	104<br>(80 to 130)	-6.6<br>(-8.3 to -5.0)
Cyprus	1 780<br>(1 517 to 2 097)	401<br>(340 to 472)	-7.5<br>(-12.4 to -2.1)	3 788<br>(3 599 to 3 986)	747<br>(709 to 787)	-5.9<br>(-7.6 to -4.1)	549<br>(424 to 670)	109<br>(83 to 135)	-4.6<br>(-5.6 to -2.9)
Denmark	9 601<br>(8 047 to 11 618)	351<br>(295 to 423)	-11.9<br>(-18.0 to -6.1)	22 015<br>(20 975 to 23 042)	639<br>(606 to 670)	-9.9<br>(-11.6 to -7.7)	3 104<br>(2 382 to 3 856)	91<br>(69 to 114)	-9.1<br>(-9.4 to -8.8)
Finland	11 092<br>(9 171 to 13 730)	409<br>(342 to 493)	-5.4<br>(-10.9 to 0.2)	24 098<br>(22 995 to 25 199)	709<br>(673 to 744)	-3.4<br>(-5.7 to -1.2)	3 418<br>(2 578 to 4 218)	102<br>(78 to 127)	-2.4<br>(-3.4 to -1.7)
France	114 325<br>(95 501 to 138 855)	368<br>(310 to 437)	-17.7<br>(-22.8 to -12.2)	253 751<br>(240 918 to 266 606)	669<br>(633 to 704)	-16.2<br>(-18.0 to -14.5)	36 069<br>(27 636 to 44 237)	97<br>(75 to 118)	-14.9<br>(-16.0 to -13.4)
Germany	128 435<br>(107 715 to 155 780)	337<br>(282 to 401)	-12.6<br>(-19.0 to -6.3)	320 773<br>(306 151 to 335 988)	615<br>(585 to 646)	-11.9<br>(-14.0 to -10.1)	44 955<br>(34 068 to 55 772)	88<br>(66 to 109)	-12.2<br>(-13.2 to -11.6)
Greece	19 886<br>(16 945 to 23 567)	396<br>(337 to 471)	-5.6<br>(-12.8 to 1.1)	51 804<br>(49 213 to 54 430)	762<br>(720 to 803)	-3.8<br>(-5.6 to -1.6)	7 386<br>(5 701 to 9 110)	111<br>(83 to 135)	-3.2<br>(-3.7 to -2.8)
Iceland	537<br>(448 to 650)	330<br>(276 to 398)	-7.5<br>(-12.4 to -2.6)	1 134<br>(1 083 to 1 189)	606<br>(578 to 636)	-7.4<br>(-9.4 to -5.4)	160<br>(120 to 198)	86<br>(64 to 108)	-6.7<br>(-8.3 to -4.5)
Ireland	7 611<br>(6 350 to 9 153)	349<br>(290 to 420)	-7.1<br>(-13.4 to -0.9)	16 016<br>(15 236 to 16 797)	629<br>(597 to 661)	-8.3<br>(-10.4 to -6.2)	2 267<br>(1 734 to 2 763)	90<br>(68 to 112)	-7.8<br>(-9.1 to -6.9)
Israel	13 680<br>(11 517 to 16 207)	339<br>(286 to 401)	-3.2<br>(-11.3 to 3.4)	26 895<br>(25 131 to 29 465)	681<br>(636 to 747)	6.5<br>(2.9 to 11.8)	3 844<br>(2 938 to 4 783)	98<br>(74 to 120)	6.4<br>(5.0 to 7.9)
Italy	106 192<br>(90 101 to 126 380)	380<br>(323 to 450)	-12.7<br>(-17.2 to -7.6)	272 364<br>(259 003 to 285 045)	711<br>(674 to 748)	-10.9<br>(-12.7 to -9.2)	38 693<br>(28 591 to 47 658)	103<br>(76 to 129)	-10.4<br>(-10.7 to -10.2)
Luxembourg	1 013<br>(852 to 1 213)	356<br>(299 to 423)	-28.4<br>(-33.8 to -22.8)	2 248<br>(2 136 to 2 352)	648<br>(615 to 679)	-27.6<br>(-29.3 to -25.8)	321<br>(240 to 401)	93<br>(68 to 118)	-26.0<br>(-28.1 to -24.8)
Malta	676<br>(564 to 819)	345<br>(289 to 416)	-9.8<br>(-14.2 to -5.2)	1 631<br>(1 555 to 1 706)	628<br>(597 to 659)	-7.6<br>(-9.5 to -5.8)	229<br>(174 to 291)	89<br>(68 to 112)	-7.0<br>(-7.9 to -6.2)
Netherlands	24 615<br>(21 032 to 29 105)	305<br>(262 to 359)	-5.2<br>(-11.3 to 1.2)	59 347<br>(56 725 to 62 030)	570<br>(543 to 597)	-4.2<br>(-6.4 to -1.9)	8 250<br>(6 071 to 10 349)	80<br>(60 to 98)	-5.0<br>(-6.7 to -3.8)
Norway	8 938<br>(7 406 to 10 841)	345<br>(287 to 415)	-7.7<br>(-13.0 to -2.3)	19 497<br>(18 560 to 20 420)	623<br>(592 to 654)	-6.5<br>(-8.5 to -4.2)	2 760<br>(2 114 to 3 341)	89<br>(70 to 107)	-5.4<br>(-5.8 to -4.8)
Portugal	15 750<br>(13 420 to 18 703)	324<br>(276 to 381)	-33.0<br>(-39.2 to -26.6)	38 489<br>(36 739 to 40 164)	600<br>(572 to 627)	-31.9<br>(-34.1 to -29.8)	5 509<br>(4 324 to 6 861)	88<br>(67 to 107)	-30.2<br>(-30.4 to -29.8)
Spain	72 012<br>(60 581 to 85 638)	334<br>(282 to 398)	-19.0<br>(-25.0 to -13.2)	179 151<br>(170 408 to 186 656)	624<br>(592 to 653)	-18.6<br>(-20.6 to -16.3)	26 166<br>(20 194 to 32 167)	93<br>(69 to 116)	-16.2<br>(-17.3 to -14.7)
Sweden	16 434<br>(13 578 to 20 083)	343<br>(285 to 416)	-1.8<br>(-6.8 to 2.9)	36 855<br>(35 254 to 38 475)	613<br>(585 to 642)	-2.2<br>(-4.2 to -0.3)	5 176<br>(3 989 to 6 357)	87<br>(66 to 108)	-1.5<br>(-3.1 to -0.5)
Switzerland	14 298<br>(11 765 to 17 408)	344<br>(284 to 413)	-29.3<br>(-34.1 to -25.0)	31 325<br>(29 916 to 32 683)	598<br>(570 to 625)	-29.4<br>(-31.2 to -27.7)	4 487<br>(3 418 to 5 564)	87<br>(66 to 108)	-28.1<br>(-28.9 to -27.2)
United Kingdom	95 321<br>(79 610 to 115 274)	310<br>(259 to 371)	-6.0<br>(-10.2 to -1.9)	215 278<br>(205 005 to 224 536)	562<br>(534 to 588)	-6.5<br>(-7.8 to -5.2)	30 342<br>(23 147 to 37 966)	80<br>(60 to 99)	-6.0<br>(-7.0 to -5.0)
**Southern Latin America**	**96 552<br>(82 071 to 114 460)**	**300<br>(254 to 355)**	**10.9<br>(7.1 to 14.8)**	**172 336<br>(165 504 to 179 722)**	**531<br>(510 to 553)**	**15.5<br>(13.8 to 17.0)**	**24 007<br>(18 764 to 29 412)**	**74<br>(58 to 92)**	**13.8<br>(12.6 to 15.6)**
Argentina	64 880<br>(55 161 to 76 654)	302<br>(257 to 357)	13.5<br>(8.0 to 19.0)	113 467<br>(108 718 to 118 316)	538<br>(516 to 561)	18.6<br>(16.4 to 20.7)	15 877<br>(12 296 to 19 638)	76<br>(58 to 93)	16.5<br>(15.1 to 18.4)
Chile	26 103<br>(22 094 to 31 001)	286<br>(243 to 340)	2.2<br>(-2.2 to 6.5)	48 491<br>(46 558 to 50 512)	505<br>(484 to 526)	6.8<br>(4.9 to 8.7)	6 684<br>(5 073 to 8 347)	70<br>(54 to 87)	6.6<br>(5.6 to 8.2)
Uruguay	5 566<br>(4 694 to 6 618)	331<br>(280 to 394)	14.1<br>(8.4 to 20.3)	10 368<br>(9 916 to 10 827)	581<br>(555 to 608)	18.4<br>(16.3 to 20.8)	1 443<br>(1 085 to 1 769)	81<br>(62 to 100)	16.2<br>(16.1 to 16.4)
**Central Europe, Eastern Europe, and Central Asia**	**2 038 155<br>(1 819 850 to 2 312 271)**	**1 007<br>(897 to 1 144)**	**-5.5<br>(-8.1 to -2.9)**	**4 496 028<br>(4 278 576 to 4 728 193)**	**2 033<br>(1 935 to 2 140)**	**-2.9<br>(-4.0 to -1.5)**	**661 543<br>(492 659 to 838 160)**	**301<br>(219 to 380)**	**-1.2<br>(-1.9 to -0.8)**
**Eastern Europe**	**1 077 351<br>(963 349 to 1 220 888)**	**1 055<br>(943 to 1 190)**	**-4.1<br>(-7.5 to -0.6)**	**2 301 924<br>(2 191 876 to 2 419 169)**	**2 022<br>(1 928 to 2 128)**	**-4.8<br>(-6.5 to -2.8)**	**339 571<br>(246 474 to 437 177)**	**300<br>(220 to 385)**	**-2.5<br>(-3.7 to -1.8)**
Belarus	54 618<br>(48 071 to 62 838)	1 166<br>(1 029 to 1 338)	14.4<br>(8.6 to 19.9)	117 682<br>(111 126 to 124 428)	2 262<br>(2 135 to 2 389)	12.5<br>(10.0 to 15.4)	17 177<br>(12 310 to 21 528)	332<br>(241 to 424)	13.1<br>(11.5 to 14.0)
Estonia	6 208<br>(5 459 to 7 112)	970<br>(858 to 1 112)	-21.9<br>(-26.2 to -16.8)	14 692<br>(13 917 to 15 558)	1 976<br>(1 869 to 2 099)	-16.9<br>(-19.2 to -14.2)	2 164<br>(1 577 to 2 734)	295<br>(211 to 380)	-14.2<br>(-16.1 to -13.0)
Latvia	9 635<br>(8 503 to 11 030)	1 008<br>(893 to 1 149)	-23.2<br>(-27.3 to -18.2)	21 793<br>(20 659 to 22 933)	1 973<br>(1 868 to 2 081)	-20.9<br>(-22.9 to -18.7)	3 226<br>(2 339 to 4 065)	295<br>(212 to 379)	-17.3<br>(-18.6 to -16.4)
Lithuania	16 568<br>(14 624 to 19 117)	1 161<br>(1 027 to 1 334)	-5.8<br>(-10.7 to -0.2)	36 746<br>(34 833 to 38 778)	2 252<br>(2 128 to 2 380)	-5.3<br>(-8.1 to -2.8)	5 365<br>(3 991 to 6 802)	334<br>(241 to 420)	-2.2<br>(-3.6 to -1.4)
Moldova	16 301<br>(14 531 to 18 498)	813<br>(723 to 926)	-18.9<br>(-23.3 to -14.6)	35 221<br>(33 147 to 37 252)	1 611<br>(1 520 to 1 704)	-17.4<br>(-19.8 to -15.0)	5 165<br>(3 734 to 6 528)	237<br>(172 to 303)	-15.0<br>(-15.6 to -13.9)
Russia	769 484<br>(689 721 to 876 352)	1 092<br>(974 to 1 237)	-3.4<br>(-7.8 to 1.2)	1 618 660<br>(1 542 745 to 1 703 738)	2 075<br>(1 977 to 2 187)	-4.1<br>(-6.3 to -1.5)	238 862<br>(175 017 to 300 706)	308<br>(225 to 393)	-1.7<br>(-3.2 to -0.8)
Ukraine	204 537<br>(181 368 to 232 705)	937<br>(829 to 1 067)	-7.9<br>(-12.2 to -3.2)	457 131<br>(433 369 to 481 426)	1 833<br>(1 737 to 1 932)	-8.7<br>(-11.2 to -6.6)	67 628<br>(49 861 to 85 855)	274<br>(195 to 342)	-6.5<br>(-6.8 to -6.1)
**Central Europe**	**661 626<br>(580 546 to 761 741)**	**1 137<br>(1 002 to 1 305)**	**-3.0<br>(-6.3 to 0.2)**	**1 632 650<br>(1 547 004 to 1 726 843)**	**2 365<br>(2 236 to 2 505)**	**3.3<br>(2.1 to 4.7)**	**237 581<br>(177 776 to 297 730)**	**349<br>(266 to 442)**	**4.1<br>(3.7 to 4.5)**
Albania	12 705<br>(11 254 to 14 411)	884<br>(780 to 1 004)	7.5<br>(3.3 to 12.0)	31 456<br>(29 776 to 33 359)	1 981<br>(1 872 to 2 100)	10.9<br>(7.7 to 14.9)	4 608<br>(3 343 to 5 883)	293<br>(215 to 376)	10.7<br>(10.3 to 11.0)
Bosnia and Herzegovina	16 737<br>(14 721 to 19 037)	911<br>(802 to 1 035)	46.5<br>(40.2 to 52.7)	49 459<br>(46 170 to 53 287)	2 108<br>(1 965 to 2 272)	58.7<br>(51.6 to 69.1)	7 168<br>(5 348 to 9 060)	310<br>(230 to 388)	54.9<br>(54.8 to 55.0)
Bulgaria	36 511<br>(32 081 to 41 684)	1 029<br>(914 to 1 180)	-10.5<br>(-15.4 to -5.2)	97 285<br>(91 953 to 102 660)	2 171<br>(2 049 to 2 299)	-7.2<br>(-9.5 to -4.6)	14 178<br>(10 370 to 18 246)	322<br>(229 to 413)	-5.7<br>(-7.8 to -4.6)
Croatia	23 008<br>(20 166 to 26 517)	1 054<br>(935 to 1 197)	-2.3<br>(-7.9 to 3.6)	56 187<br>(53 641 to 58 802)	2 174<br>(2 073 to 2 278)	2.5<br>(-0.7 to 6.4)	8 190<br>(5 910 to 10 350)	322<br>(235 to 412)	2.9<br>(1.7 to 3.9)
Czech Republic	69 759<br>(60 644 to 80 420)	1 319<br>(1 152 to 1 512)	-1.5<br>(-6.5 to 3.9)	181 187<br>(171 174 to 192 569)	2 796<br>(2 633 to 2 980)	10.7<br>(7.7 to 13.6)	26 529<br>(18 979 to 33 243)	415<br>(301 to 521)	12.1<br>(11.2 to 12.7)
Hungary	55 474<br>(47 687 to 65 054)	1 113<br>(961 to 1 296)	-17.7<br>(-22.8 to -12.6)	126 635<br>(119 199 to 133 872)	2 177<br>(2 051 to 2 310)	-8.6<br>(-11.2 to -5.7)	18 540<br>(13 820 to 23 474)	323<br>(236 to 407)	-6.7<br>(-7.0 to -6.4)
Macedonia	9 426<br>(8 331 to 10 725)	927<br>(817 to 1 060)	14.0<br>(9.6 to 18.7)	23 767<br>(22 389 to 25 228)	1 989<br>(1 868 to 2 117)	17.3<br>(13.8 to 21.2)	3 490<br>(2 463 to 4 457)	295<br>(206 to 374)	17.2<br>(15.3 to 18.2)
Montenegro	3 292<br>(2 915 to 3 724)	1 057<br>(934 to 1 202)	5.6<br>(1.8 to 9.4)	8 097<br>(7 666 to 8 549)	2 284<br>(2 156 to 2 414)	8.8<br>(6.1 to 11.6)	1 186<br>(869 to 1 508)	338<br>(249 to 433)	9.6<br>(9.3 to 10.2)
Poland	233 210<br>(203 585 to 271 614)	1 198<br>(1 049 to 1 385)	0.8<br>(-4.9 to 6.4)	561 049<br>(529 882 to 596 395)	2 466<br>(2 324 to 2 628)	7.8<br>(4.9 to 10.7)	81 344<br>(58 204 to 103 599)	362<br>(270 to 462)	7.7<br>(7.7 to 7.7)
Romania	111 240<br>(96 713 to 129 381)	1 139<br>(996 to 1 313)	-10.2<br>(-15.7 to -4.3)	272 941<br>(257 852 to 288 989)	2 342<br>(2 210 to 2 484)	-7.9<br>(-10.4 to -5.5)	39 659<br>(28 990 to 50 343)	345<br>(253 to 433)	-6.6<br>(-7.5 to -4.9)
Serbia	41 607<br>(36 757 to 47 265)	964<br>(851 to 1 096)	11.8<br>(7.7 to 16.3)	108 202<br>(102 031 to 114 664)	2 100<br>(1 979 to 2 234)	16.9<br>(13.0 to 20.9)	15 744<br>(11 602 to 20 242)	310<br>(228 to 392)	15.6<br>(15.0 to 16.1)

Location	Incidence (95% UI)			Prevalence (95% UI)			YLDs (95% UI)		
	2016 counts	2016 age-standardised rates per 100,000	Percentage change in age-standardised rates between 1990 and 2016	2016 counts	2016 age-standardised rates per 100,000	Percentage change in age-standardised rates between 1990 and 2016	2016 counts	2016 age-standardised rates per 100,000	Percentage change in age-standardised rates between 1990 and 2016
Slovakia	33 496 (29 146 to 38 875)	1 213 (1 064 to 1 395)	-9.0 (-13.3 to -4.3)	78 557 (74 276 to 83 214)	2 464 (2 326 to 2 611)	-1.7 (-4.1 to 1.0)	11 442 (8 433 to 14 650)	363 (261 to 466)	-0.7 (-2.1 to 0.2)
Slovenia	15 160 (12 998 to 17 847)	1 380 (1 198 to 1 608)	-7.0 (-12.8 to -1.0)	37 828 (35 841 to 40 087)	2 858 (2 704 to 3 029)	0.1 (-2.4 to 2.5)	5 551 (4 183 to 6 909)	427 (310 to 534)	3.6 (3.1 to 4.2)
**Central Asia**	**299 178 (267 269 to 337 474)**	**685 (611 to 772)**	**-2.1 (-5.0 to 1.1)**	**561 454 (531 673 to 592 519)**	**1 427 (1 356 to 1 502)**	**-0.5 (-1.8 to 0.9)**	**83 732 (61 649 to 105 842)**	**211 (151 to 265)**	**0.9 (0.5 to 1.2)**
Armenia	9 386 (8 358 to 10 582)	670 (595 to 757)	-12.7 (-17.3 to -7.6)	23 418 (21 714 to 25 352)	1 550 (1 438 to 1 679)	-12.5 (-16.2 to -8.9)	3 481 (2 558 to 4 423)	231 (172 to 292)	-10.2 (-10.5 to -9.9)
Azerbaijan	30 481 (27 193 to 34 386)	621 (551 to 704)	-8.8 (-12.6 to -4.6)	65 008 (61 415 to 68 935)	1 328 (1 258 to 1 403)	-4.1 (-7.0 to -1.1)	9 571 (6 893 to 12 315)	195 (141 to 245)	-4.0 (-4.1 to -3.8)
Georgia	13 683 (12 234 to 15 269)	703 (627 to 788)	-5.5 (-10.1 to -1.1)	31 260 (29 685 to 32 808)	1 468 (1 392 to 1 542)	-3.8 (-6.6 to -0.5)	4 535 (3 260 to 5 797)	214 (157 to 275)	-4.0 (-4.4 to -3.8)
Kazakhstan	72 747 (64 955 to 82 007)	845 (752 to 952)	7.9 (2.4 to 13.7)	136 750 (129 222 to 143 832)	1 691 (1 605 to 1 773)	8.8 (6.0 to 11.8)	20 506 (14 302 to 26 516)	250 (175 to 318)	11.4 (11.1 to 11.9)
Kyrgyzstan	19 019 (16 851 to 21 529)	645 (574 to 726)	-16.7 (-20.7 to -12.4)	33 051 (31 267 to 35 145)	1 327 (1 262 to 1 403)	-13.8 (-16.5 to -11.2)	4 938 (3 533 to 6 308)	196 (143 to 249)	-12.5 (-12.9 to -12.1)
Mongolia	13 804 (12 300 to 15 663)	908 (809 to 1 034)	57.6 (49.6 to 65.9)	23 567 (22 221 to 25 029)	1 753 (1 666 to 1 855)	57.2 (51.5 to 63.5)	3 486 (2 440 to 4 475)	256 (182 to 333)	55.0 (53.5 to 55.9)
Tajikistan	25 065 (22 033 to 28 792)	574 (505 to 658)	-17.8 (-22.0 to -13.5)	42 311 (39 450 to 45 247)	1 252 (1 173 to 1 334)	-12.2 (-15.7 to -7.7)	6 330 (4 739 to 8 028)	184 (136 to 237)	-11.9 (-12.6 to -10.7)
Turkmenistan	17 028 (15 205 to 19 252)	615 (550 to 695)	-2.4 (-7.0 to 2.4)	30 694 (28 847 to 32 615)	1 282 (1 210 to 1 356)	1.2 (-1.6 to 4.0)	4 627 (3 319 to 5 953)	191 (138 to 249)	2.2 (1.3 to 2.8)
Uzbekistan	97 966 (86 888 to 110 562)	639 (567 to 724)	3.0 (-2.0 to 7.6)	175 395 (164 718 to 186 438)	1 324 (1 249 to 1 400)	4.1 (1.7 to 7.1)	26 216 (18 765 to 34 176)	196 (140 to 249)	5.4 (4.7 to 6.2)
**Latin America and Caribbean**	**1 322 893 (1 193 782 to 1 476 139)**	**472 (427 to 528)**	**1.8 (-0.2 to 3.8)**	**2 666 456 (2 545 581 to 2 782 055)**	**1 001 (958 to 1 043)**	**6.5 (5.1 to 7.9)**	**394 613 (282 994 to 496 180)**	**148 (106 to 190)**	**6.6 (6.2 to 6.9)**
**Central Latin America**	**514 854 (465 951 to 572 465)**	**416 (376 to 462)**	**-11.9 (-14.1 to -9.9)**	**1 011 898 (964 694 to 1 058 898)**	**893 (854 to 932)**	**-6.7 (-7.8 to -5.5)**	**151 157 (107 186 to 194 283)**	**133 (95 to 170)**	**-5.3 (-5.8 to -4.9)**
Colombia	100 415 (89 721 to 111 860)	425 (381 to 475)	-11.0 (-14.9 to -6.9)	215 235 (205 100 to 226 430)	935 (892 to 981)	-3.5 (-6.1 to -0.8)	32 054 (22 687 to 41 046)	140 (100 to 177)	-3.2 (-5.4 to -1.8)
Costa Rica	10 727 (9 628 to 11 959)	445 (399 to 496)	19.1 (13.9 to 24.8)	24 312 (22 965 to 25 494)	986 (933 to 1 033)	25.4 (21.3 to 29.5)	3 601 (2 552 to 4 634)	147 (107 to 189)	23.6 (23.3 to 24.1)
El Salvador	13 260 (11 935 to 14 803)	463 (417 to 517)	-1.4 (-8.4 to 4.5)	25 500 (24 023 to 27 153)	1 004 (948 to 1 071)	5.3 (-0.3 to 9.6)	3 790 (2 719 to 4 801)	150 (109 to 189)	5.9 (4.8 to 7.4)
Guatemala	33 989 (30 502 to 38 022)	447 (401 to 499)	13.0 (6.1 to 19.2)	52 941 (50 425 to 55 849)	912 (873 to 955)	19.9 (15.6 to 23.7)	7 877 (5 597 to 9 975)	134 (97 to 171)	19.2 (18.4 to 20.2)
Honduras	14 850 (13 248 to 16 669)	378 (340 to 423)	19.9 (15.4 to 24.8)	26 350 (24 621 to 28 373)	798 (751 to 851)	24.5 (18.7 to 32.1)	3 939 (2 943 to 4 921)	119 (90 to 149)	22.8 (20.8 to 26.3)
Mexico	241 915 (218 590 to 269 666)	387 (349 to 432)	-23.2 (-25.3 to -21.2)	468 567 (445 892 to 490 468)	814 (778 to 850)	-19.6 (-20.9 to -18.1)	70 248 (49 161 to 90 618)	122 (89 to 158)	-17.2 (-17.6 to -16.8)
Nicaragua	10 884 (9 758 to 12 211)	369 (332 to 414)	1.0 (-3.1 to 5.2)	20 399 (19 081 to 22 007)	825 (775 to 886)	-2.2 (-7.4 to 1.9)	3 036 (2 187 to 3 894)	123 (87 to 161)	-0.4 (-2.5 to 1.1)
Panama	9 213 (8 300 to 10 309)	465 (418 to 520)	23.0 (18.3 to 28.2)	19 292 (18 297 to 20 224)	1 028 (977 to 1 076)	28.0 (24.6 to 31.7)	2 836 (2 004 to 3 652)	152 (106 to 195)	25.0 (24.4 to 26.2)
Venezuela	79 601 (72 041 to 89 312)	510 (461 to 569)	15.7 (9.8 to 22.0)	159 302 (151 332 to 166 743)	1 112 (1 060 to 1 161)	21.0 (17.6 to 24.2)	23 738 (17 485 to 30 576)	166 (117 to 210)	19.8 (19.6 to 19.9)
**Andean Latin America**	**125 327 (112 663 to 138 842)**	**432 (388 to 479)**	**6.6 (3.4 to 9.8)**	**240 417 (228 179 to 252 469)**	**929 (884 to 972)**	**11.9 (9.6 to 14.3)**	**35 564 (24 946 to 45 027)**	**137 (98 to 176)**	**11.5 (8.8 to 12.9)**
Bolivia	22 349 (20 030 to 25 046)	419 (375 to 468)	-4.8 (-8.4 to -1.3)	39 972 (37 821 to 42 214)	885 (840 to 932)	2.5 (-0.2 to 5.6)	5 908 (4 065 to 7 686)	130 (92 to 169)	2.6 (0.7 to 3.7)
Ecuador	40 415 (36 034 to 45 320)	508 (452 to 569)	13.4 (6.3 to 21.9)	73 547 (69 662 to 77 054)	1 028 (978 to 1 075)	10.7 (7.5 to 14.0)	10 861 (7 658 to 14 094)	152 (111 to 195)	10.0 (8.7 to 10.7)
Peru	62 563 (55 986 to 69 318)	398 (357 to 442)	6.1 (1.8 to 10.8)	126 898 (119 883 to 133 937)	892 (844 to 936)	15.5 (11.1 to 19.7)	18 794 (12 944 to 24 386)	132 (93 to 172)	14.9 (10.7 to 17.4)
**Caribbean**	**100 478 (90 685 to 111 950)**	**447 (402 to 498)**	**20.0 (16.7 to 23.4)**	**222 492 (203 939 to 244 606)**	**998 (917 to 1 095)**	**27.7 (19.5 to 40.2)**	**32 596 (23 258 to 42 509)**	**147 (105 to 189)**	**25.6 (25.0 to 26.0)**
Antigua and Barbuda	184 (165 to 205)	422 (378 to 471)	15.0 (10.9 to 19.4)	416 (392 to 438)	945 (893 to 994)	19.2 (15.7 to 23.0)	62 (43 to 80)	141 (100 to 183)	18.0 (17.4 to 18.4)
The Bahamas	851 (764 to 951)	444 (398 to 496)	5.4 (1.3 to 9.7)	1 902 (1 806 to 2 004)	956 (908 to 1 007)	10.9 (7.4 to 14.7)	282 (204 to 373)	142 (99 to 183)	10.6 (8.6 to 11.8)
Barbados	513 (460 to 570)	387 (347 to 431)	18.9 (14.8 to 23.1)	1 336 (1 266 to 1 405)	867 (820 to 914)	22.8 (18.5 to 27.0)	198 (142 to 251)	130 (91 to 165)	22.0 (21.5 to 22.3)
Belize	858 (771 to 957)	470 (424 to 523)	36.1 (29.9 to 42.4)	1 457 (1 381 to 1 530)	969 (923 to 1 010)	34.7 (30.5 to 39.1)	218 (160 to 279)	144 (104 to 181)	31.3 (30.6 to 32.7)
Bermuda	168 (149 to 188)	479 (427 to 535)	-8.4 (-12.6 to -3.8)	378 (358 to 397)	1 077 (1 023 to 1 130)	0.3 (-2.7 to 3.6)	57 (41 to 73)	162 (115 to 209)	1.5 (-0.9 to 3.0)
Cuba	25 916 (22 828 to 29 485)	442 (389 to 503)	7.7 (2.6 to 13.5)	63 690 (59 951 to 67 123)	925 (870 to 978)	6.2 (2.5 to 10.3)	9 303 (6 405 to 11 785)	137 (97 to 178)	5.9 (4.3 to 6.7)
Dominica	154 (138 to 173)	417 (374 to 467)	36.0 (31.1 to 41.0)	331 (313 to 348)	898 (852 to 944)	37.8 (32.5 to 43.0)	49 (34 to 63)	135 (93 to 175)	36.8 (35.2 to 37.7)
Dominican Republic	23 619 (21 216 to 26 471)	445 (402 to 496)	33.9 (28.8 to 39.8)	47 117 (44 602 to 49 644)	993 (944 to 1 044)	37.2 (32.6 to 41.9)	7 015 (4 812 to 9 104)	148 (99 to 193)	34.9 (31.9 to 36.3)
Grenada	251 (224 to 281)	469 (419 to 525)	29.2 (24.8 to 33.9)	472 (446 to 498)	957 (908 to 1 004)	29.4 (25.2 to 33.1)	70 (50 to 90)	142 (107 to 178)	27.8 (26.1 to 30.6)
Guyana	1 712 (1 530 to 1 922)	447 (402 to 500)	15.7 (11.3 to 20.7)	3 085 (2 927 to 3 244)	867 (825 to 907)	19.9 (16.2 to 23.2)	454 (325 to 580)	127 (93 to 162)	18.2 (18.0 to 18.4)
Haiti	23 180 (20 649 to 26 241)	421 (376 to 472)	20.7 (13.6 to 29.8)	49 678 (35 386 to 70 051)	1 080 (803 to 1 468)	62.9 (21.3 to 120.1)	7 138 (4 707 to 9 524)	154 (107 to 200)	57.4 (52.1 to 60.3)
Jamaica	5 584 (4 997 to 6 230)	386 (346 to 430)	43.2 (38.8 to 48.3)	11 751 (11 094 to 12 363)	850 (804 to 892)	41.9 (36.5 to 47.7)	1 736 (1 223 to 2 229)	126 (91 to 163)	38.5 (35.6 to 43.5)
Puerto Rico	8 894 (7 931 to 9 985)	495 (441 to 554)	19.3 (13.7 to 25.5)	20 812 (19 673 to 21 932)	1 063 (1 004 to 1 122)	22.5 (18.1 to 27.1)	3 053 (2 220 to 3 929)	158 (117 to 199)	20.7 (19.5 to 22.9)
Saint Lucia	378 (337 to 421)	421 (376 to 468)	20.3 (16.0 to 24.6)	850 (806 to 896)	922 (874 to 970)	25.9 (21.9 to 30.3)	126 (93 to 160)	137 (99 to 174)	24.3 (22.0 to 28.6)
Saint Vincent and the Grenadines	257 (230 to 290)	464 (415 to 525)	29.3 (24.2 to 34.1)	519 (490 to 547)	941 (890 to 990)	28.8 (24.3 to 33.4)	76 (52 to 101)	139 (96 to 182)	26.7 (23.5 to 28.7)
Suriname	1 212 (1 087 to 1 353)	448 (403 to 498)	30.4 (25.4 to 35.7)	2 475 (2 350 to 2 596)	944 (898 to 987)	30.2 (25.5 to 35.0)	366 (265 to 475)	139 (100 to 176)	29.0 (25.8 to 30.8)
Trinidad and Tobago	3 000 (2 707 to 3 340)	461 (416 to 512)	28.3 (22.2 to 34.3)	7 081 (6 703 to 7 409)	978 (925 to 1 023)	35.5 (31.2 to 39.8)	1 040 (740 to 1 372)	144 (104 to 186)	35.1 (32.8 to 36.5)
Virgin Islands, U.S.	228 (204 to 256)	448 (400 to 500)	15.4 (10.9 to 19.8)	552 (526 to 580)	930 (881 to 980)	16.4 (13.0 to 20.3)	81 (59 to 100)	138 (98 to 178)	14.8 (14.3 to 15.6)
**Tropical Latin America**	**582 235 (521 847 to 655 384)**	**553 (496 to 624)**	**10.9 (8.1 to 14.0)**	**1 191 650 (1 132 750 to 1 248 736)**	**1 137 (1 083 to 1 190)**	**14.6 (12.0 to 17.1)**	**175 135 (126 061 to 224 232)**	**168 (122 to 212)**	**14.0 (13.7 to 14.4)**
Brazil	566 203 (507 507 to 637 161)	555 (498 to 627)	10.3 (7.5 to 13.5)	1 161 831 (1 104 428 to 1 217 222)	1 141 (1 087 to 1 194)	14.2 (11.6 to 16.7)	170 746 (121 339 to 219 898)	168 (122 to 214)	13.6 (13.4 to 14.1)
Paraguay	16 031 (14 196 to 18 170)	473 (421 to 533)	36.9 (31.6 to 42.4)	29 819 (28 095 to 31 787)	1 007 (952 to 1 066)	34.5 (28.9 to 40.1)	4 381 (3 103 to 5 638)	148 (103 to 190)	31.0 (27.5 to 32.9)
**Southeast Asia, East Asia, and Oceania**	**4 094 677 (3 725 521 to 4 499 463)**	**379 (345 to 415)**	**33.1 (28.1 to 38.3)**	**10 256 604 (9 848 618 to 10 663 956)**	**881 (847 to 916)**	**47.0 (44.8 to 49.4)**	**1 516 411 (1 035 581 to 1 917 976)**	**131 (95 to 168)**	**43.4 (42.7 to 44.1)**
**East Asia**	**2 892 584 (2 625 657 to 3 176 695)**	**388 (353 to 426)**	**34.2 (28.9 to 40.2)**	**7 702 324 (7 406 200 to 7 999 447)**	**906 (871 to 940)**	**46.9 (44.6 to 49.5)**	**1 139 370 (823 395 to 1 445 381)**	**135 (97 to 174)**	**43.6 (42.8 to 44.2)**
China	2 802 814 (2 543 742 to 3 078 818)	389 (355 to 427)	34.1 (28.6 to 40.2)	7 479 129 (7 190 336 to 7 765 791)	909 (874 to 944)	47.1 (44.7 to 49.6)	1 106 391 (784 851 to 1 431 104)	136 (98 to 173)	43.8 (42.9 to 44.4)
North Korea	45 557 (41 418 to 50 435)	343 (312 to 381)	56.0 (49.0 to 63.8)	99 570 (95 143 to 104 062)	748 (715 to 780)	53.7 (49.4 to 57.6)	14 716 (10 527 to 18 900)	111 (81 to 139)	47.7 (46.3 to 50.0)
Taiwan (Province of China)	44 213 (40 077 to 48 889)	369 (335 to 408)	25.8 (20.0 to 32.3)	123 625 (118 691 to 128 604)	863 (827 to 899)	31.3 (27.8 to 35.3)	18 260 (12 823 to 23 433)	129 (92 to 168)	28.2 (27.5 to 28.7)
**Southeast Asia**	**1 181 948 (1 076 577 to 1 302 369)**	**361 (330 to 397)**	**31.8 (24.9 to 37.6)**	**2 521 808 (2 398 235 to 2 645 455)**	**817 (780 to 855)**	**48.9 (45.8 to 52.4)**	**371 661 (274 817 to 469 387)**	**120 (89 to 153)**	**44.0 (42.8 to 45.0)**
Cambodia	25 721 (23 353 to 28 420)	333 (303 to 367)	22.8 (4.1 to 37.1)	48 044 (43 702 to 55 613)	799 (714 to 958)	20.1 (-0.1 to 39.4)	7 055 (5 095 to 9 019)	116 (85 to 149)	19.8 (13.8 to 29.1)
Indonesia	422 153 (383 580 to 465 790)	326 (296 to 359)	34.4 (30.1 to 38.9)	895 737 (853 157 to 939 641)	730 (697 to 764)	45.6 (41.8 to 49.7)	131 770 (94 800 to 165 985)	107 (77 to 138)	40.7 (39.8 to 42.2)
Laos	11 067 (10 048 to 12 242)	302 (274 to 333)	14.8 (-11.9 to 34.8)	17 357 (16 546 to 18 203)	643 (615 to 670)	52.4 (48.0 to 56.6)	2 589 (1 792 to 3 456)	94 (66 to 120)	49.5 (45.4 to 52.0)
Malaysia	65 982 (59 760 to 73 003)	424 (385 to 468)	39.6 (34.2 to 45.5)	138 807 (132 411 to 145 580)	956 (914 to 1 000)	46.4 (43.3 to 49.2)	20 455 (14 535 to 25 911)	141 (104 to 184)	41.8 (40.9 to 42.6)
Maldives	503 (456 to 556)	269 (245 to 296)	10.7 (7.0 to 14.6)	1 024 (973 to 1 073)	635 (606 to 664)	23.2 (20.3 to 26.7)	151 (113 to 190)	94 (70 to 120)	21.0 (19.5 to 22.2)
Mauritius	2 253 (2 043 to 2 480)	358 (324 to 395)	46.2 (40.2 to 52.5)	5 611 (5 362 to 5 856)	805 (769 to 841)	57.7 (53.0 to 62.1)	815 (586 to 1 045)	118 (85 to 152)	51.4 (48.9 to 52.8)
Myanmar	87 874 (79 472 to 97 340)	331 (299 to 366)	48.6 (42.7 to 54.3)	195 075 (177 599 to 218 816)	783 (716 to 872)	76.2 (63.2 to 92.1)	28 703 (20 370 to 36 961)	115 (83 to 148)	72.6 (70.7 to 74.7)
Philippines	191 920 (172 163 to 216 239)	366 (329 to 411)	28.7 (19.9 to 37.8)	338 269 (320 164 to 359 197)	764 (725 to 809)	41.4 (36.8 to 45.2)	50 271 (36 824 to 63 198)	112 (86 to 141)	37.1 (36.6 to 37.7)
Sri Lanka	40 908 (37 350 to 45 066)	414 (378 to 456)	-27.1 (-50.2 to -4.2)	109 230 (98 927 to 124 998)	1 062 (963 to 1 213)	36.3 (27.5 to 49.1)	15 871 (11 737 to 19 995)	155 (117 to 198)	30.3 (29.4 to 31.5)
Seychelles	200 (182 to 221)	402 (367 to 442)	33.5 (28.8 to 38.8)	437 (418 to 456)	864 (828 to 902)	44.1 (41.0 to 47.7)	64 (49 to 82)	127 (94 to 161)	37.7 (36.9 to 39.0)
Thailand	157 510 (142 639 to 174 687)	458 (416 to 506)	35.8 (30.8 to 41.4)	403 367 (385 307 to 421 718)	1 031 (984 to 1 078)	44.3 (40.7 to 48.1)	59 327 (40 875 to 76 782)	153 (109 to 194)	39.5 (39.3 to 39.8)
Timor-Leste	1 730 (1 568 to 1 900)	299 (271 to 330)	-31.1 (-59.1 to 3.7)	3 901 (3 251 to 5 095)	945 (769 to 1 266)	35.6 (24.9 to 46.7)	570 (400 to 737)	136 (96 to 173)	30.1 (25.2 to 39.0)
Vietnam	172 627 (156 433 to 192 663)	365 (331 to 405)	57.1 (52.0 to 63.2)	361 305 (344 204 to 378 804)	794 (758 to 830)	67.0 (62.4 to 70.9)	53 234 (38 342 to 68 676)	117 (87 to 150)	60.4 (60.0 to 61.0)

Location	Incidence (95% UI)			Prevalence (95% UI)			YLDs (95% UI)		
	2016 counts	2016 age-standardised rates per 100,000	Percentage change in age-standardised rates between 1990 and 2016	2016 counts	2016 age-standardised rates per 100,000	Percentage change in age-standardised rates between 1990 and 2016	2016 counts	2016 age-standardised rates per 100,000	Percentage change in age-standardised rates between 1990 and 2016
**Oceania**	**20 145 (18 348 to 22 230)**	**355 (324 to 392)**	**25.8 (21.1 to 30.2)**	**32 472 (30 950 to 34 100)**	**696 (665 to 729)**	**38.4 (35.7 to 40.9)**	**4 816 (3 429 to 6 171)**	**102 (71 to 128)**	**34.3 (31.9 to 35.7)**
American Samoa	143 (131 to 157)	368 (336 to 403)	7.4 (4.0 to 11.0)	268 (254 to 281)	798 (764 to 835)	15.4 (12.6 to 18.4)	40 (29 to 51)	118 (84 to 151)	14.2 (13.5 to 14.6)
Federated States of Micronesia	176 (160 to 194)	335 (306 to 368)	24.6 (20.7 to 29.1)	284 (270 to 300)	664 (635 to 697)	28.7 (25.3 to 31.6)	42 (30 to 53)	98 (71 to 126)	26.8 (24.7 to 30.3)
Fiji	1 394 (1 277 to 1 532)	320 (292 to 350)	36.6 (31.9 to 41.6)	2 809 (2 689 to 2 939)	650 (623 to 680)	41.8 (38.8 to 44.5)	416 (308 to 533)	96 (68 to 120)	38.8 (38.4 to 39.2)
Guam	357 (326 to 391)	406 (371 to 444)	34.7 (29.9 to 39.6)	770 (737 to 806)	873 (836 to 913)	35.8 (33.1 to 38.7)	113 (81 to 145)	129 (94 to 163)	31.4 (30.2 to 33.2)
Kiribati	184 (166 to 207)	319 (288 to 358)	43.7 (38.6 to 49.4)	285 (271 to 299)	610 (582 to 639)	50.2 (46.9 to 53.8)	42 (31 to 54)	89 (64 to 114)	44.5 (43.4 to 46.1)
Marshall Islands	129 (117 to 141)	339 (310 to 372)	28.1 (23.5 to 33.1)	202 (192 to 212)	662 (634 to 694)	29.7 (26.6 to 32.8)	30 (21 to 39)	96 (72 to 120)	27.1 (25.8 to 28.0)
Northern Mariana Islands	251 (228 to 279)	378 (345 to 416)	11.3 (8.0 to 14.9)	480 (456 to 505)	830 (795 to 869)	13.1 (10.5 to 15.8)	73 (53 to 95)	123 (88 to 160)	11.8 (10.7 to 13.8)
Papua New Guinea	14 365 (13 060 to 15 876)	363 (330 to 401)	23.1 (17.2 to 28.3)	21 819 (20 766 to 22 986)	699 (667 to 733)	39.6 (36.3 to 42.9)	3 237 (2 306 to 4 081)	102 (73 to 132)	35.2 (32.6 to 36.9)
Samoa	332 (303 to 364)	326 (298 to 358)	21.8 (18.1 to 25.8)	585 (557 to 618)	710 (679 to 747)	31.7 (28.4 to 35.9)	87 (62 to 112)	105 (77 to 134)	28.6 (28.2 to 29.4)
Solomon Islands	1 027 (938 to 1 131)	344 (314 to 379)	30.3 (26.5 to 34.7)	1 551 (1 478 to 1 632)	674 (645 to 705)	36.7 (34.0 to 39.6)	231 (172 to 291)	99 (73 to 126)	33.4 (32.4 to 35.1)
Tonga	182 (167 to 200)	340 (311 to 375)	21.8 (17.2 to 26.8)	299 (286 to 314)	700 (672 to 731)	28.7 (25.7 to 31.8)	44 (32 to 56)	103 (76 to 130)	26.5 (24.7 to 29.8)
Vanuatu	459 (418 to 503)	326 (298 to 359)	38.6 (33.8 to 43.4)	737 (702 to 778)	660 (631 to 695)	46.9 (43.4 to 50.7)	110 (83 to 138)	97 (73 to 124)	43.4 (41.4 to 46.8)
**North Africa and Middle East**	**1 611 196 (1 329 164 to 2 054 379)**	**532 (443 to 670)**	**13.7 (-0.9 to 37.7)**	**2 582 530 (2 397 364 to 2 872 377)**	**1 003 (933 to 1 113)**	**0.2 (-2.1 to 2.6)**	**381 401 (287 110 to 469 929)**	**147 (110 to 185)**	**1.0 (0.6 to 1.3)**
**North Africa and Middle East**	**1 611 196 (1 329 164 to 2 054 379)**	**532 (443 to 670)**	**13.7 (-0.9 to 37.7)**	**2 582 530 (2 397 364 to 2 872 377)**	**1 003 (933 to 1 113)**	**0.2 (-2.1 to 2.6)**	**381 401 (287 110 to 469 929)**	**147 (110 to 185)**	**1.0 (0.6 to 1.3)**
Afghanistan	136 821 (76 928 to 244 886)	748 (433 to 1 323)	64.0 (-1.0 to 162.4)	145 703 (107 931 to 210 937)	1 287 (887 to 2 033)	-12.3 (-24.4 to 10.2)	20 907 (13 087 to 28 433)	180 (107 to 254)	-11.8 (-14.9 to -3.5)
Algeria	79 589 (71 702 to 87 796)	393 (355 to 433)	-8.2 (-11.3 to -4.6)	164 253 (156 222 to 172 607)	901 (860 to 944)	-2.2 (-4.7 to 0.6)	24 352 (17 861 to 30 450)	133 (101 to 167)	-0.9 (-1.5 to 0.0)
Bahrain	3 439 (3 110 to 3 780)	397 (359 to 438)	-11.2 (-14.8 to -7.4)	7 909 (7 495 to 8 343)	920 (877 to 966)	-4.4 (-7.3 to -1.5)	1 196 (850 to 1 546)	137 (99 to 175)	-3.7 (-5.5 to -2.8)
Egypt	167 184 (150 991 to 185 080)	356 (323 to 394)	25.6 (21.4 to 30.2)	299 587 (284 755 to 314 356)	746 (712 to 781)	23.9 (20.1 to 28.3)	44 926 (32 852 to 57 684)	111 (82 to 137)	24.0 (22.9 to 25.7)
Iran	205 967 (185 771 to 228 273)	496 (448 to 549)	-31.9 (-48.9 to -11.5)	466 826 (433 278 to 517 776)	1 217 (1 128 to 1 355)	-7.6 (-12.9 to -2.8)	69 091 (51 500 to 85 333)	180 (135 to 224)	-4.3 (-6.2 to -2.2)
Iraq	175 479 (108 617 to 303 788)	850 (537 to 1 459)	71.8 (10.2 to 187.7)	205 281 (165 191 to 282 726)	1 541 (1 216 to 2 182)	-4.1 (-12.8 to 5.9)	29 590 (20 666 to 39 570)	217 (143 to 289)	-7.9 (-10.4 to -5.5)
Jordan	18 184 (14 525 to 24 175)	441 (355 to 582)	3.4 (-14.1 to 37.7)	26 037 (24 707 to 27 568)	809 (772 to 849)	-14.3 (-17.3 to -11.3)	3 829 (2 677 to 4 929)	118 (87 to 151)	-13.5 (-14.6 to -11.6)
Kuwait	10 545 (9 521 to 11 652)	469 (425 to 517)	-34.2 (-54.7 to -11.9)	23 740 (22 440 to 24 967)	1 112 (1 058 to 1 162)	-5.5 (-8.8 to -2.7)	3 618 (2 756 to 4 612)	167 (125 to 209)	-2.9 (-4.1 to -0.9)
Lebanon	11 927 (10 210 to 14 410)	411 (352 to 492)	-48.9 (-64.5 to -32.6)	41 467 (31 257 to 61 957)	1 401 (1 052 to 2 101)	-28.1 (-35.4 to -19.0)	5 932 (3 760 to 8 299)	202 (123 to 274)	-24.5 (-28.3 to -13.1)
Libya	17 294 (12 922 to 24 994)	548 (412 to 783)	33.5 (3.1 to 91.7)	30 689 (26 609 to 37 526)	1 053 (924 to 1 270)	10.7 (-1.0 to 31.2)	4 474 (3 305 to 5 670)	154 (117 to 192)	9.8 (5.5 to 15.7)
Morocco	61 280 (55 312 to 67 618)	367 (332 to 405)	-1.2 (-4.7 to 2.7)	131 850 (126 005 to 138 547)	826 (790 to 867)	3.5 (0.7 to 6.1)	19 519 (13 996 to 24 863)	122 (85 to 158)	3.9 (1.3 to 5.4)
Palestine	10 304 (9 258 to 11 584)	367 (331 to 410)	-0.1 (-16.7 to 13.6)	16 291 (14 521 to 19 288)	928 (810 to 1 138)	1.0 (-9.0 to 11.1)	2 433 (1 757 to 3 097)	136 (95 to 178)	1.4 (-0.5 to 4.7)
Oman	15 910 (14 130 to 17 686)	501 (452 to 553)	-8.1 (-12.2 to -3.9)	33 302 (31 457 to 35 005)	1 135 (1 084 to 1 187)	-5.7 (-8.2 to -3.5)	5 032 (3 467 to 6 446)	170 (121 to 217)	-3.8 (-4.4 to -2.6)
Qatar	10 007 (8 926 to 11 197)	568 (513 to 628)	-4.0 (-7.7 to -0.2)	21 026 (19 863 to 22 236)	1 331 (1 270 to 1 396)	-1.5 (-4.1 to 0.6)	3 208 (2 291 to 4 090)	199 (144 to 256)	0.7 (0.4 to 1.2)
Saudi Arabia	86 845 (78 127 to 95 457)	483 (435 to 531)	-12.7 (-14.7 to -10.6)	177 647 (169 893 to 186 397)	1 059 (1 016 to 1 107)	-11.7 (-13.3 to -10.4)	26 751 (19 825 to 33 816)	159 (118 to 198)	-9.6 (-9.8 to -9.4)
Sudan	81 773 (71 591 to 94 327)	400 (351 to 458)	6.4 (-1.6 to 14.2)	126 191 (118 790 to 136 601)	829 (783 to 894)	12.0 (9.0 to 15.8)	18 710 (13 840 to 23 917)	121 (89 to 152)	12.3 (11.5 to 13.8)
Syria	172 849 (61 879 to 344 363)	1 744 (625 to 3 483)	452.9 (99.5 to 1 018.1)	96 153 (69 401 to 139 724)	1 185 (883 to 1 670)	65.9 (28.2 to 125.7)	13 149 (8 488 to 17 816)	164 (111 to 216)	55.6 (34.6 to 73.1)
Tunisia	21 564 (19 555 to 23 982)	394 (357 to 435)	5.5 (1.5 to 9.9)	48 462 (46 075 to 50 912)	864 (822 to 905)	7.5 (4.2 to 10.8)	7 218 (5 222 to 9 429)	129 (93 to 167)	8.7 (5.4 to 10.8)
Turkey	156 542 (140 765 to 173 894)	401 (361 to 444)	-18.0 (-22.0 to -12.9)	335 832 (320 448 to 353 987)	889 (849 to 933)	-14.6 (-17.5 to -11.4)	50 239 (36 496 to 63 760)	133 (97 to 168)	-13.1 (-13.4 to -13.0)
United Arab Emirates	38 020 (34 063 to 42 248)	529 (477 to 582)	-8.1 (-10.9 to -5.2)	87 319 (82 820 to 92 175)	1 180 (1 129 to 1 237)	-7.4 (-10.0 to -5.1)	13 063 (9 277 to 16 941)	175 (125 to 225)	-6.1 (-7.6 to -5.2)
Yemen	128 026 (86 582 to 212 869)	840 (575 to 1 353)	97.2 (35.7 to 216.8)	93 962 (85 382 to 107 344)	931 (864 to 1 033)	3.3 (-1.6 to 10.8)	13 590 (10 277 to 17 452)	134 (102 to 167)	3.2 (0.5 to 6.6)
**South Asia**	**4 522 146 (4 092 824 to 4 998 757)**	**539 (488 to 597)**	**7.6 (5.3 to 10.1)**	**7 834 390 (7 516 828 to 8 137 747)**	**1 024 (983 to 1 063)**	**19.8 (18.6 to 21.2)**	**1 162 059 (841 335 to 1 483 799)**	**151 (112 to 192)**	**18.8 (18.0 to 19.3)**
**South Asia**	**4 522 146 (4 092 824 to 4 998 757)**	**539 (488 to 597)**	**7.6 (5.3 to 10.1)**	**7 834 390 (7 516 828 to 8 137 747)**	**1 024 (983 to 1 063)**	**19.8 (18.6 to 21.2)**	**1 162 059 (841 335 to 1 483 799)**	**151 (112 to 192)**	**18.8 (18.0 to 19.3)**
Bangladesh	343 748 (311 223 to 378 569)	427 (387 to 471)	10.1 (6.2 to 14.4)	619 828 (592 120 to 650 817)	873 (835 to 913)	26.5 (22.9 to 30.7)	91 742 (66 498 to 117 969)	129 (94 to 161)	14.4 (13.2 to 16.2)
Bhutan	2 164 (1 950 to 2 412)	523 (469 to 582)	3.4 (-0.0 to 6.9)	3 595 (3 423 to 3 777)	958 (915 to 1 002)	3.6 (0.6 to 6.4)	537 (399 to 683)	143 (106 to 180)	4.3 (4.2 to 4.4)
India	3 617 027 (3 268 881 to 4 009 746)	555 (502 to 614)	6.2 (3.7 to 8.7)	6 301 981 (6 047 026 to 6 549 351)	1 041 (999 to 1 081)	18.7 (17.6 to 19.9)	933 407 (684 061 to 1 175 945)	153 (115 to 191)	18.6 (17.9 to 19.2)
Nepal	66 479 (60 081 to 73 840)	483 (435 to 536)	4.2 (0.5 to 8.3)	110 004 (104 794 to 116 043)	964 (919 to 1 013)	19.0 (15.4 to 23.0)	16 849 (12 903 to 21 399)	145 (105 to 185)	21.0 (20.6 to 21.6)
Pakistan	492 728 (446 049 to 546 994)	520 (472 to 578)	19.6 (15.7 to 23.6)	798 982 (762 486 to 838 158)	1 028 (984 to 1 073)	25.8 (22.3 to 29.7)	119 438 (86 547 to 154 109)	152 (112 to 192)	24.8 (24.6 to 25.2)
**Sub-Saharan Africa**	**1 954 002 (1 767 785 to 2 165 653)**	**436 (394 to 482)**	**-11.3 (-19.8 to -6.2)**	**2 714 715 (2 583 531 to 2 852 607)**	**832 (794 to 871)**	**1.7 (0.6 to 2.9)**	**405 876 (298 498 to 512 129)**	**121 (89 to 155)**	**2.1 (1.8 to 2.5)**
**Southern sub-Saharan Africa**	**164 606 (149 441 to 182 679)**	**444 (404 to 490)**	**-13.3 (-17.1 to -10.8)**	**259 110 (247 549 to 272 040)**	**832 (799 to 869)**	**-14.6 (-15.8 to -13.5)**	**38 510 (27 154 to 49 172)**	**121 (88 to 153)**	**-15.1 (-15.5 to -14.4)**
Botswana	5 183 (4 676 to 5 766)	466 (422 to 516)	15.2 (11.9 to 18.8)	8 143 (7 703 to 8 547)	860 (819 to 899)	12.7 (10.2 to 15.0)	1 203 (873 to 1 501)	125 (94 to 156)	11.9 (10.0 to 15.3)
Lesotho	4 780 (4 316 to 5 313)	470 (427 to 519)	24.0 (20.0 to 27.9)	5 794 (5 504 to 6 110)	711 (683 to 746)	6.9 (4.8 to 9.5)	856 (652 to 1 074)	103 (77 to 132)	4.3 (2.2 to 7.9)
Namibia	4 849 (4 392 to 5 374)	404 (367 to 445)	-0.9 (-4.0 to 2.2)	7 056 (6 680 to 7 424)	763 (728 to 798)	0.8 (-1.2 to 2.8)	1 059 (772 to 1 364)	112 (81 to 143)	0.3 (-0.5 to 0.7)
South Africa	121 543 (110 435 to 134 978)	471 (429 to 521)	-18.0 (-22.3 to -15.0)	201 847 (192 678 to 211 961)	886 (851 to 927)	-18.8 (-19.9 to -17.6)	29 899 (21 231 to 38 202)	129 (95 to 163)	-19.3 (-19.6 to -19.1)
Swaziland	3 127 (2 823 to 3 483)	496 (448 to 552)	13.9 (9.7 to 17.9)	3 952 (3 758 to 4 153)	837 (802 to 874)	3.9 (1.8 to 6.3)	581 (416 to 753)	119 (88 to 149)	1.0 (-0.1 to 1.9)
Zimbabwe	25 125 (22 622 to 27 843)	345 (312 to 380)	5.3 (2.7 to 8.0)	32 316 (30 630 to 34 157)	595 (570 to 621)	2.2 (-0.4 to 4.4)	4 834 (3 431 to 6 174)	86 (62 to 111)	2.5 (0.5 to 6.4)
**Western sub-Saharan Africa**	**774 434 (698 672 to 860 641)**	**422 (380 to 468)**	**-1.5 (-3.8 to 0.6)**	**1 068 517 (1 015 125 to 1 124 350)**	**803 (766 to 838)**	**3.8 (2.1 to 5.6)**	**160 346 (118 593 to 207 065)**	**118 (87 to 147)**	**4.5 (4.4 to 4.8)**
Benin	22 150 (20 003 to 24 636)	438 (396 to 487)	3.6 (0.1 to 7.4)	30 618 (29 047 to 32 195)	823 (786 to 859)	5.9 (2.6 to 8.5)	4 599 (3 338 to 5 836)	121 (87 to 154)	6.4 (5.6 to 7.0)
Burkina Faso	33 500 (30 140 to 37 304)	398 (358 to 443)	-0.7 (-4.0 to 2.5)	42 598 (40 246 to 44 910)	735 (698 to 768)	6.8 (3.6 to 10.2)	6 416 (4 475 to 8 249)	108 (81 to 138)	8.4 (6.9 to 9.3)
Cameroon	46 750 (42 135 to 52 096)	421 (380 to 469)	5.4 (1.9 to 9.1)	61 711 (58 317 to 65 441)	752 (716 to 787)	1.8 (-1.0 to 4.9)	9 277 (6 733 to 11 928)	110 (80 to 139)	2.6 (2.1 to 3.0)
Cape Verde	1 122 (1 016 to 1 242)	423 (385 to 464)	12.9 (10.1 to 16.0)	1 815 (1 716 to 1 912)	855 (815 to 894)	15.5 (11.9 to 18.7)	274 (192 to 360)	128 (92 to 162)	15.0 (14.7 to 15.2)
Chad	28 262 (25 476 to 31 460)	421 (379 to 468)	-8.3 (-23.0 to 2.5)	35 924 (33 731 to 38 547)	805 (761 to 858)	4.2 (0.8 to 7.4)	5 399 (3 885 to 6 969)	117 (84 to 148)	4.5 (3.3 to 5.3)
Cote d'Ivoire	48 566 (43 523 to 54 085)	454 (407 to 505)	-4.1 (-7.2 to -0.8)	66 947 (63 345 to 70 633)	805 (766 to 843)	-1.3 (-4.3 to 1.4)	10 008 (7 289 to 12 668)	118 (88 to 145)	-0.8 (-1.4 to 0.3)
The Gambia	3 518 (3 156 to 3 919)	376 (338 to 418)	-7.0 (-9.3 to -4.6)	4 593 (4 343 to 4 867)	716 (683 to 752)	-7.2 (-9.8 to -4.6)	696 (499 to 893)	106 (77 to 133)	-6.1 (-6.9 to -5.6)
Ghana	55 881 (50 388 to 62 135)	441 (398 to 492)	18.2 (14.9 to 21.4)	81 489 (77 195 to 85 924)	830 (790 to 868)	20.6 (17.4 to 24.4)	12 175 (8 746 to 15 462)	121 (91 to 153)	20.2 (19.4 to 21.1)
Guinea	23 273 (21 035 to 25 854)	394 (356 to 438)	-4.0 (-6.7 to -1.4)	32 174 (30 558 to 33 887)	724 (691 to 755)	-5.1 (-7.8 to -2.2)	4 843 (3 482 to 6 161)	106 (78 to 135)	-4.5 (-8.3 to -2.0)
Guinea-Bissau	3 751 (3 390 to 4 151)	436 (394 to 483)	-6.2 (-8.6 to -3.8)	5 011 (4 760 to 5 280)	747 (715 to 783)	-4.2 (-7.0 to -1.6)	752 (537 to 969)	110 (81 to 140)	-4.0 (-4.5 to -3.1)
Liberia	7 485 (6 740 to 8 368)	354 (318 to 395)	-63.6 (-78.2 to -44.5)	11 810 (10 748 to 13 457)	755 (689 to 863)	-3.8 (-10.9 to 6.1)	1 739 (1 292 to 2 167)	108 (83 to 137)	1.8 (0.3 to 3.3)
Mali	33 147 (29 950 to 36 754)	398 (361 to 441)	-5.0 (-8.9 to -1.7)	43 020 (40 755 to 45 769)	772 (737 to 815)	5.9 (2.3 to 11.2)	6 497 (4 546 to 8 241)	113 (83 to 143)	7.3 (4.8 to 8.8)
Mauritania	8 324 (7 494 to 9 255)	442 (399 to 490)	-11.4 (-22.7 to -3.1)	13 038 (12 373 to 13 720)	901 (859 to 944)	5.5 (2.9 to 8.6)	1 959 (1 431 to 2 546)	133 (94 to 168)	6.1 (5.2 to 7.8)
Niger	35 149 (31 725 to 38 927)	380 (344 to 421)	-12.7 (-16.0 to -9.8)	44 924 (42 696 to 47 280)	702 (670 to 732)	-8.6 (-11.4 to -5.9)	6 855 (4 780 to 8 909)	104 (73 to 134)	-7.2 (-9.2 to -5.9)
Nigeria	369 616 (332 556 to 411 843)	427 (384 to 476)	1.1 (-2.1 to 4.7)	518 948 (489 740 to 547 292)	834 (791 to 875)	4.6 (1.7 to 7.7)	77 676 (54 520 to 100 173)	122 (87 to 155)	5.4 (4.8 to 6.5)
Sao Tome and Principe	482 (434 to 538)	540 (485 to 604)	5.4 (1.6 to 9.3)	670 (633 to 705)	1 067 (1 012 to 1 117)	6.8 (3.5 to 10.0)	100 (68 to 132)	157 (115 to 202)	6.3 (5.1 to 7.1)

Location	Incidence (95% UI)			Prevalence (95% UI)			YLDs (95% UI)		
	2016 counts	2016 age-standardised rates per 100,000	Percentage change in age-standardised rates between 1990 and 2016	2016 counts	2016 age-standardised rates per 100,000	Percentage change in age-standardised rates between 1990 and 2016	2016 counts	2016 age-standardised rates per 100,000	Percentage change in age-standardised rates between 1990 and 2016
Senegal	28 823<br>(25 989 to 31 868)	425<br>(383 to 472)	0.7<br>(-2.7 to 4.3)	38 758<br>(36 748 to 40 896)	802<br>(765 to 839)	4.7<br>(1.3 to 7.7)	5 857<br>(4 279 to 7 545)	118<br>(86 to 150)	4.8<br>(3.6 to 5.6)
Sierra Leone	11 478<br>(10 336 to 12 796)	382<br>(343 to 425)	-12.8<br>(-15.4 to -10.2)	16 598<br>(15 527 to 18 018)	749<br>(702 to 814)	-4.9<br>(-9.8 to 3.4)	2 491<br>(1 886 to 3 125)	110<br>(84 to 135)	-6.6<br>(-7.4 to -5.5)
Togo	13 148<br>(11 864 to 14 580)	397<br>(357 to 440)	1.5<br>(-1.7 to 5.0)	17 851<br>(16 974 to 18 853)	715<br>(683 to 749)	0.2<br>(-2.6 to 3.8)	2 691<br>(1 940 to 3 456)	105<br>(74 to 135)	0.5<br>(-0.3 to 1.4)
**Eastern sub-Saharan Africa**	**777 542<br>(702 030 to 864 415)**	**449<br>(405 to 497)**	**-19.4<br>(-33.8 to -9.4)**	**1 066 251<br>(1 008 834 to 1 133 715)**	**860<br>(816 to 913)**	**6.4<br>(4.3 to 8.0)**	**159 065<br>(115 990 to 203 211)**	**125<br>(92 to 159)**	**7.0<br>(6.2 to 7.9)**
Burundi	24 194<br>(21 747 to 26 873)	477<br>(427 to 533)	2.8<br>(-0.4 to 6.0)	33 375<br>(31 238 to 36 242)	918<br>(856 to 1 000)	25.4<br>(19.1 to 37.0)	5 014<br>(3 596 to 6 471)	134<br>(99 to 167)	23.1<br>(21.8 to 25.0)
Comoros	1 636<br>(1 478 to 1 815)	448<br>(405 to 496)	-24.7<br>(-27.0 to -22.2)	2 473<br>(2 344 to 2 611)	884<br>(844 to 925)	-19.8<br>(-21.9 to -17.7)	371<br>(265 to 478)	131<br>(97 to 165)	-18.2<br>(-19.0 to -16.8)
Djibouti	2 209<br>(1 996 to 2 454)	487<br>(439 to 544)	-9.9<br>(-16.5 to -4.9)	3 453<br>(3 261 to 3 635)	951<br>(904 to 997)	-1.5<br>(-3.9 to 1.0)	516<br>(370 to 661)	141<br>(102 to 180)	-2.1<br>(-2.3 to -1.6)
Eritrea	11 402<br>(10 255 to 12 739)	487<br>(440 to 542)	-2.5<br>(-5.9 to 0.9)	16 130<br>(15 083 to 17 394)	947<br>(888 to 1 016)	14.8<br>(9.5 to 22.8)	2 436<br>(1 760 to 3 148)	140<br>(104 to 178)	13.9<br>(12.6 to 15.8)
Ethiopia	208 969<br>(188 122 to 232 521)	455<br>(411 to 505)	-45.4<br>(-62.9 to -28.5)	299 554<br>(281 764 to 320 650)	894<br>(843 to 957)	2.3<br>(-2.5 to 6.3)	44 525<br>(32 710 to 56 846)	130<br>(97 to 164)	5.7<br>(3.8 to 7.9)
Kenya	99 212<br>(89 433 to 110 498)	473<br>(428 to 525)	13.9<br>(12.8 to 15.1)	141 288<br>(134 499 to 148 449)	899<br>(861 to 937)	17.3<br>(16.0 to 18.7)	21 020<br>(15 010 to 27 008)	131<br>(94 to 170)	16.3<br>(16.1 to 16.4)
Madagascar	45 642<br>(41 001 to 51 010)	396<br>(356 to 442)	-4.4<br>(-7.8 to -0.8)	63 235<br>(59 617 to 66 886)	741<br>(703 to 778)	-0.9<br>(-3.8 to 1.8)	9 553<br>(6 908 to 12 149)	110<br>(82 to 141)	-0.3<br>(-0.8 to 0.0)
Malawi	28 946<br>(25 894 to 32 477)	354<br>(318 to 395)	-10.1<br>(-12.9 to -7.4)	35 580<br>(33 526 to 37 914)	616<br>(585 to 648)	-8.5<br>(-11.8 to -5.5)	5 317<br>(3 565 to 6 926)	89<br>(65 to 116)	-7.6<br>(-8.0 to -7.3)
Mozambique	56 577<br>(50 846 to 62 915)	459<br>(414 to 509)	1.5<br>(-10.6 to 10.4)	74 620<br>(69 471 to 81 406)	877<br>(808 to 987)	3.6<br>(-6.3 to 11.4)	11 004<br>(7 836 to 14 037)	126<br>(92 to 158)	2.6<br>(0.5 to 5.0)
Rwanda	20 886<br>(18 694 to 23 408)	396<br>(355 to 440)	-34.8<br>(-49.7 to -23.5)	37 754<br>(31 705 to 48 843)	1 047<br>(861 to 1 387)	30.2<br>(8.6 to 71.8)	5 630<br>(3 931 to 7 452)	152<br>(103 to 198)	20.7<br>(11.6 to 27.2)
Somalia	25 303<br>(21 124 to 32 170)	533<br>(450 to 660)	-12.5<br>(-18.3 to -7.5)	29 712<br>(27 306 to 33 432)	872<br>(805 to 976)	1.2<br>(-3.2 to 7.5)	4 407<br>(3 180 to 5 574)	127<br>(95 to 159)	-3.1<br>(-5.8 to -0.8)
South Sudan	30 822<br>(27 522 to 34 530)	514<br>(459 to 576)	-30.6<br>(-50.9 to -10.0)	42 529<br>(39 374 to 47 032)	991<br>(918 to 1 097)	1.2<br>(-3.4 to 8.0)	6 373<br>(4 656 to 8 185)	144<br>(107 to 182)	2.9<br>(2.0 to 4.4)
Tanzania	108 797<br>(97 664 to 121 271)	438<br>(396 to 486)	-1.8<br>(-4.7 to 1.1)	145 423<br>(137 294 to 153 811)	813<br>(774 to 853)	2.4<br>(-0.3 to 5.5)	21 778<br>(15 137 to 28 714)	119<br>(84 to 152)	2.1<br>(1.9 to 2.2)
Uganda	75 334<br>(67 587 to 84 268)	418<br>(376 to 466)	5.1<br>(0.5 to 9.3)	95 240<br>(88 637 to 103 950)	812<br>(751 to 909)	13.3<br>(1.8 to 22.8)	14 257<br>(10 640 to 17 578)	118<br>(88 to 145)	15.6<br>(11.8 to 22.4)
Zambia	37 117<br>(33 297 to 41 440)	513<br>(462 to 570)	14.3<br>(10.6 to 18.1)	45 151<br>(42 776 to 47 617)	861<br>(822 to 902)	7.6<br>(4.8 to 10.5)	6 729<br>(4 921 to 8 738)	124<br>(91 to 157)	7.6<br>(7.1 to 8.3)
**Central sub-Saharan Africa**	**237 419<br>(214 193 to 262 981)**	**444<br>(400 to 493)**	**-8.9<br>(-13.1 to -5.9)**	**320 837<br>(304 288 to 340 203)**	**861<br>(818 to 910)**	**1.7<br>(-0.3 to 4.4)**	**47 808<br>(36 065 to 60 342)**	**125<br>(94 to 157)**	**2.3<br>(2.0 to 2.9)**
Angola	56 580<br>(50 991 to 62 935)	489<br>(442 to 544)	-14.3<br>(-28.4 to -3.8)	77 094<br>(72 309 to 83 142)	1 016<br>(949 to 1 115)	6.9<br>(1.4 to 11.6)	11 552<br>(8 470 to 14 483)	148<br>(112 to 188)	8.3<br>(7.2 to 9.4)
Central African Republic	9 369<br>(8 438 to 10 455)	395<br>(356 to 439)	8.6<br>(4.0 to 14.8)	11 909<br>(11 215 to 12 700)	634<br>(602 to 669)	9.9<br>(5.7 to 15.5)	1 764<br>(1 242 to 2 263)	92<br>(66 to 120)	10.2<br>(7.1 to 12.1)
Congo	9 962<br>(8 976 to 10 999)	450<br>(405 to 498)	-1.1<br>(-3.6 to 1.6)	15 213<br>(14 209 to 16 552)	928<br>(868 to 1 010)	21.7<br>(15.6 to 32.6)	2 268<br>(1 643 to 2 868)	136<br>(98 to 169)	22.4<br>(19.1 to 24.3)
DR Congo	155 603<br>(140 339 to 172 368)	432<br>(390 to 480)	-8.7<br>(-11.0 to -6.1)	207 191<br>(196 520 to 218 785)	825<br>(785 to 865)	-2.5<br>(-5.5 to 1.5)	30 817<br>(23 196 to 38 872)	120<br>(90 to 151)	-2.1<br>(-2.7 to -1.1)
Equatorial Guinea	2 068<br>(1 860 to 2 303)	510<br>(459 to 568)	16.2<br>(12.3 to 20.4)	3 384<br>(3 216 to 3 535)	1 007<br>(962 to 1 050)	39.2<br>(35.3 to 43.1)	503<br>(366 to 645)	147<br>(107 to 186)	38.2<br>(35.1 to 40.3)
Gabon	3 837<br>(3 443 to 4 245)	444<br>(400 to 492)	-17.7<br>(-20.1 to -15.1)	6 047<br>(5 771 to 6 343)	879<br>(841 to 918)	-8.5<br>(-10.7 to -6.3)	899<br>(634 to 1 146)	129<br>(93 to 163)	-7.4<br>(-7.8 to -7.2)

Appendix Table 2: Incidence, prevalence, and YLDs for 2016 and percentage change of age-standardized rates by location for TBI, females

Location	Incidence (95% UI)			Prevalence (95% UI)			YLDs (95% UI)		
	2016 counts	2016 age-standardised rates per 100,000	Percentage change in age-standardised rates between 1990 and 2016	2016 counts	2016 age-standardised rates per 100,000	Percentage change in age-standardised rates between 1990 and 2016	2016 counts	2016 age-standardised rates per 100,000	Percentage change in age-standardised rates between 1990 and 2016
**Global**	**9 616 584<br>(8 449 889 to 10 971 055)**	**264<br>(232 to 301)**	**1.8<br>(-0.1 to 4.1)**	**20 717 512<br>(19 923 947 to 21 543 337)**	**562<br>(540 to 584)**	**6.1<br>(5.3 to 6.9)**	**2 995 985<br>(2 239 649 to 3 811 711)**	**82<br>(60 to 103)**	**6.1<br>(5.4 to 6.6)**
**High SDI**	**1 498 566<br>(1 221 233 to 1 856 149)**	**258<br>(214 to 312)**	**-7.8<br>(-11.3 to -4.3)**	**3 521 971<br>(3 370 374 to 3 687 990)**	**499<br>(477 to 522)**	**-7.6<br>(-8.5 to -6.7)**	**496 720<br>(378 359 to 621 038)**	**73<br>(55 to 91)**	**-7.7<br>(-8.0 to -7.4)**
**High-middle SDI**	**1 971 585<br>(1 730 251 to 2 237 268)**	**333<br>(292 to 376)**	**-8.0<br>(-11.0 to -5.3)**	**5 190 120<br>(4 991 210 to 5 387 775)**	**778<br>(748 to 809)**	**-3.2<br>(-4.4 to -2.1)**	**769 945<br>(566 318 to 988 523)**	**117<br>(86 to 145)**	**-2.1<br>(-2.3 to -2.0)**
**Middle SDI**	**2 472 169<br>(2 197 877 to 2 763 079)**	**222<br>(197 to 248)**	**17.9<br>(15.1 to 20.6)**	**5 990 793<br>(5 766 822 to 6 230 543)**	**506<br>(486 to 526)**	**27.6<br>(26.1 to 29.3)**	**885 520<br>(666 132 to 1 113 763)**	**75<br>(56 to 95)**	**24.3<br>(23.9 to 24.8)**
**Low-middle SDI**	**2 815 117<br>(2 473 873 to 3 197 786)**	**287<br>(252 to 327)**	**6.2<br>(2.7 to 12.5)**	**4 860 148<br>(4 638 251 to 5 099 885)**	**544<br>(520 to 569)**	**15.3<br>(14.1 to 16.6)**	**721 646<br>(522 434 to 910 340)**	**80<br>(61 to 99)**	**14.1<br>(13.4 to 14.7)**
**Low SDI**	**902 325<br>(777 390 to 1 071 292)**	**252<br>(220 to 293)**	**-9.6<br>(-15.6 to -6.3)**	**1 239 259<br>(1 160 662 to 1 338 737)**	**461<br>(436 to 495)**	**2.8<br>(1.1 to 4.7)**	**188 246<br>(144 059 to 231 260)**	**68<br>(53 to 84)**	**3.0<br>(2.4 to 3.9)**
**High-income**	**1 352 379<br>(1 082 021 to 1 711 075)**	**228<br>(187 to 279)**	**-7.9<br>(-12.1 to -3.9)**	**3 102 603<br>(2 961 336 to 3 253 385)**	**428<br>(408 to 449)**	**-9.9<br>(-10.8 to -8.7)**	**426 651<br>(331 180 to 521 728)**	**60<br>(45 to 76)**	**-10.7<br>(-11.3 to -10.1)**
**High-income North America**	**501 721<br>(403 023 to 635 594)**	**254<br>(206 to 313)**	**-1.8<br>(-8.1 to 5.7)**	**1 066 052<br>(1 013 364 to 1 124 325)**	**460<br>(438 to 485)**	**-7.2<br>(-9.6 to -5.0)**	**145 774<br>(113 812 to 180 515)**	**64<br>(50 to 79)**	**-10.2<br>(-11.3 to -9.0)**
Canada	45 878<br>(36 986 to 57 711)	236<br>(193 to 289)	-5.6<br>(-11.3 to -0.5)	104 161<br>(99 131 to 109 718)	437<br>(415 to 462)	-7.6<br>(-9.6 to -5.1)	14 449<br>(11 110 to 17 867)	62<br>(48 to 78)	-7.2<br>(-9.3 to -5.6)
Greenland	63<br>(50 to 79)	272<br>(219 to 343)	-12.4<br>(-15.6 to -9.2)	105<br>(100 to 110)	444<br>(423 to 468)	-9.8<br>(-11.9 to -7.7)	15<br>(11 to 18)	61<br>(48 to 75)	-8.0<br>(-8.8 to -6.9)
United States	455 610<br>(365 111 to 577 758)	256<br>(208 to 316)	-1.3<br>(-7.8 to 6.7)	961 430<br>(913 819 to 1 014 406)	463<br>(440 to 489)	-7.2<br>(-9.8 to -4.8)	131 261<br>(100 406 to 159 176)	65<br>(49 to 79)	-10.4<br>(-11.7 to -8.7)
**Australasia**	**31 351<br>(25 553 to 38 638)**	**211<br>(174 to 256)**	**-9.1<br>(-15.5 to -3.2)**	**72 041<br>(68 683 to 75 983)**	**410<br>(390 to 434)**	**-10.5<br>(-12.3 to -8.7)**	**10 043<br>(7 564 to 12 590)**	**58<br>(43 to 72)**	**-10.5<br>(-12.8 to -9.0)**
Australia	26 330<br>(21 459 to 32 577)	211<br>(173 to 257)	-8.3<br>(-15.1 to -1.7)	60 510<br>(57 701 to 63 924)	410<br>(390 to 434)	-9.7<br>(-11.6 to -7.8)	8 422<br>(6 421 to 10 371)	58<br>(44 to 71)	-9.9<br>(-12.2 to -8.3)
New Zealand	5 020<br>(4 107 to 6 157)	212<br>(175 to 260)	-12.9<br>(-19.1 to -6.4)	11 531<br>(10 977 to 12 166)	410<br>(390 to 434)	-14.1<br>(-16.1 to -12.1)	1 621<br>(1 248 to 2 016)	59<br>(45 to 72)	-13.2<br>(-14.1 to -11.8)
**High-income Asia-Pacific**	**206 322<br>(167 529 to 258 583)**	**198<br>(162 to 242)**	**-8.2<br>(-11.7 to -4.8)**	**510 690<br>(490 654 to 533 436)**	**370<br>(353 to 386)**	**-5.7<br>(-6.9 to -4.4)**	**69 836<br>(53 387 to 85 956)**	**52<br>(40 to 66)**	**-6.0<br>(-7.0 to -5.3)**
Brunei	475<br>(393 to 574)	245<br>(201 to 295)	-20.2<br>(-24.6 to -15.5)	876<br>(832 to 925)	453<br>(432 to 478)	-19.5<br>(-21.2 to -17.6)	124<br>(93 to 156)	64<br>(48 to 81)	-17.9<br>(-18.6 to -17.5)
Japan	142 718<br>(114 780 to 181 104)	194<br>(159 to 237)	-4.3<br>(-7.9 to -0.9)	371 574<br>(356 302 to 388 032)	366<br>(350 to 383)	-2.2<br>(-3.6 to -0.8)	50 559<br>(38 889 to 63 306)	52<br>(40 to 64)	-2.4<br>(-3.4 to -1.8)
Singapore	4 035<br>(3 274 to 5 009)	201<br>(163 to 247)	11.5<br>(7.0 to 16.0)	9 322<br>(8 907 to 9 778)	382<br>(364 to 401)	16.1<br>(13.4 to 18.8)	1 297<br>(978 to 1 602)	54<br>(40 to 67)	16.4<br>(14.6 to 17.5)
South Korea	59 094<br>(47 900 to 72 284)	214<br>(176 to 261)	-16.8<br>(-21.5 to -12.2)	128 919<br>(123 859 to 134 844)	385<br>(369 to 404)	-16.7<br>(-18.8 to -14.7)	17 851<br>(13 244 to 21 934)	55<br>(40 to 68)	-16.9<br>(-17.4 to -16.7)
**Western Europe**	**561 065<br>(444 377 to 711 304)**	**235<br>(191 to 290)**	**-12.8<br>(-17.9 to -8.2)**	**1 355 916<br>(1 288 515 to 1 420 035)**	**454<br>(430 to 477)**	**-13.3<br>(-14.4 to -11.9)**	**187 712<br>(139 255 to 233 422)**	**65<br>(48 to 80)**	**-12.8<br>(-13.5 to -12.3)**
Andorra	103<br>(81 to 132)	239<br>(194 to 296)	6.9<br>(2.3 to 11.8)	261<br>(248 to 276)	472<br>(447 to 499)	9.1<br>(6.9 to 11.1)	36<br>(27 to 45)	66<br>(49 to 84)	9.1<br>(8.0 to 10.6)
Austria	12 083<br>(9 484 to 15 421)	253<br>(204 to 312)	-17.1<br>(-23.7 to -11.0)	29 458<br>(28 045 to 30 817)	485<br>(462 to 509)	-16.2<br>(-18.2 to -14.2)	4 095<br>(3 142 to 5 037)	69<br>(52 to 87)	-15.1<br>(-16.0 to -14.5)
Belgium	18 705<br>(14 680 to 24 204)	280<br>(226 to 347)	-8.8<br>(-16.4 to -0.6)	40 662<br>(38 589 to 42 781)	514<br>(488 to 542)	-13.9<br>(-16.0 to -11.9)	5 601<br>(4 326 to 6 896)	73<br>(55 to 91)	-14.4<br>(-16.1 to -13.2)
Cyprus	1 180<br>(966 to 1 434)	250<br>(206 to 303)	-12.8<br>(-18.7 to -6.5)	2 817<br>(2 671 to 2 962)	500<br>(474 to 527)	-12.2<br>(-14.0 to -10.3)	403<br>(307 to 495)	72<br>(54 to 90)	-11.0<br>(-11.2 to -10.6)
Denmark	7 701<br>(6 107 to 9 833)	249<br>(201 to 308)	-18.2<br>(-23.9 to -12.0)	17 742<br>(16 872 to 18 614)	473<br>(448 to 497)	-15.4<br>(-17.5 to -13.4)	2 445<br>(1 857 to 3 057)	67<br>(51 to 83)	-15.0<br>(-15.7 to -14.4)
Finland	8 918<br>(6 963 to 11 675)	276<br>(221 to 347)	-5.4<br>(-11.5 to 0.8)	19 959<br>(18 987 to 20 972)	507<br>(482 to 532)	-6.5<br>(-8.6 to -4.4)	2 770<br>(2 122 to 3 389)	72<br>(56 to 89)	-5.7<br>(-7.4 to -3.0)
France	95 660<br>(74 730 to 123 458)	245<br>(197 to 305)	-22.5<br>(-29.1 to -16.1)	212 267<br>(201 077 to 223 283)	462<br>(437 to 488)	-23.8<br>(-25.7 to -21.8)	29 427<br>(22 240 to 36 406)	66<br>(50 to 82)	-22.6<br>(-24.1 to -21.7)
Germany	107 607<br>(84 105 to 138 652)	236<br>(190 to 294)	-12.5<br>(-18.8 to -5.6)	271 500<br>(257 388 to 286 273)	454<br>(428 to 479)	-13.4<br>(-15.5 to -11.4)	37 191<br>(28 590 to 45 859)	64<br>(48 to 79)	-13.9<br>(-15.3 to -12.8)
Greece	13 208<br>(10 799 to 16 085)	243<br>(200 to 297)	-15.7<br>(-21.0 to -9.7)	38 168<br>(36 200 to 40 248)	498<br>(471 to 527)	-13.2<br>(-14.8 to -11.3)	5 382<br>(4 173 to 6 619)	72<br>(54 to 88)	-11.9<br>(-12.4 to -11.6)
Iceland	389<br>(313 to 483)	232<br>(188 to 285)	-7.0<br>(-13.0 to -0.7)	886<br>(841 to 933)	458<br>(434 to 483)	-6.3<br>(-8.6 to -3.7)	124<br>(92 to 154)	65<br>(48 to 82)	-5.7<br>(-6.6 to -5.2)
Ireland	5 687<br>(4 584 to 7 087)	245<br>(199 to 302)	-3.4<br>(-10.4 to 3.5)	12 857<br>(12 170 to 13 508)	474<br>(448 to 499)	-3.5<br>(-5.5 to -1.4)	1 796<br>(1 340 to 2 232)	67<br>(49 to 84)	-3.8<br>(-7.1 to -1.6)
Israel	9 123<br>(7 427 to 11 232)	215<br>(176 to 265)	-6.3<br>(-13.3 to -0.5)	18 839<br>(17 677 to 20 427)	436<br>(408 to 473)	0.6<br>(-2.3 to 4.6)	2 633<br>(2 042 to 3 244)	61<br>(47 to 75)	0.4<br>(-2.3 to 4.7)
Italy	85 335<br>(67 919 to 107 373)	248<br>(202 to 304)	-10.0<br>(-15.8 to -4.1)	218 778<br>(207 314 to 229 676)	484<br>(458 to 508)	-9.4<br>(-11.3 to -7.3)	30 173<br>(22 559 to 36 931)	69<br>(51 to 86)	-9.2<br>(-10.5 to -7.4)
Luxembourg	758<br>(608 to 942)	248<br>(201 to 306)	-23.0<br>(-29.4 to -17.1)	1 731<br>(1 639 to 1 823)	476<br>(450 to 503)	-23.4<br>(-25.2 to -21.6)	242<br>(189 to 295)	68<br>(52 to 84)	-22.7<br>(-24.2 to -20.1)
Malta	494<br>(393 to 626)	230<br>(185 to 285)	-8.1<br>(-13.1 to -2.4)	1 240<br>(1 177 to 1 299)	447<br>(423 to 469)	-5.1<br>(-6.8 to -3.0)	172<br>(129 to 221)	63<br>(47 to 81)	-3.5<br>(-6.2 to -1.6)
Netherlands	22 041<br>(17 711 to 27 232)	244<br>(201 to 296)	8.2<br>(0.7 to 15.0)	53 538<br>(51 028 to 56 013)	476<br>(452 to 499)	8.7<br>(6.2 to 11.3)	7 292<br>(5 453 to 9 054)	66<br>(50 to 81)	8.0<br>(7.4 to 8.3)
Norway	7 019<br>(5 521 to 9 042)	246<br>(197 to 309)	-2.0<br>(-8.8 to 4.3)	15 418<br>(14 614 to 16 240)	467<br>(441 to 492)	-1.7<br>(-4.2 to 0.3)	2 140<br>(1 566 to 2 661)	66<br>(50 to 82)	-1.5<br>(-2.7 to -0.7)
Portugal	12 328<br>(9 999 to 15 123)	211<br>(174 to 256)	-22.7<br>(-29.0 to -16.8)	32 494<br>(30 950 to 34 034)	416<br>(396 to 437)	-23.3<br>(-25.4 to -21.1)	4 540<br>(3 575 to 5 645)	60<br>(46 to 73)	-22.4<br>(-23.7 to -20.6)
Spain	56 434<br>(45 331 to 70 630)	231<br>(189 to 284)	-12.6<br>(-19.5 to -5.7)	149 065<br>(141 401 to 156 766)	462<br>(437 to 487)	-14.0<br>(-15.9 to -11.8)	21 079<br>(16 115 to 26 115)	67<br>(50 to 85)	-13.2<br>(-13.7 to -12.8)
Sweden	11 672<br>(9 182 to 15 093)	217<br>(175 to 269)	-8.8<br>(-14.5 to -2.8)	26 608<br>(25 378 to 27 993)	409<br>(389 to 430)	-11.2<br>(-13.3 to -9.1)	3 653<br>(2 828 to 4 503)	57<br>(43 to 71)	-11.7<br>(-12.6 to -10.8)
Switzerland	10 825<br>(8 386 to 14 073)	221<br>(176 to 277)	-28.4<br>(-34.6 to -22.2)	23 487<br>(22 297 to 24 681)	400<br>(380 to 420)	-29.5<br>(-31.5 to -27.8)	3 282<br>(2 453 to 4 131)	57<br>(43 to 73)	-27.8<br>(-29.6 to -26.6)
United Kingdom	73 258<br>(57 616 to 93 989)	210<br>(169 to 261)	-5.2<br>(-10.1 to -0.6)	166 855<br>(158 604 to 175 214)	394<br>(374 to 414)	-6.1<br>(-7.3 to -4.9)	23 032<br>(17 602 to 28 835)	56<br>(41 to 69)	-5.4<br>(-5.9 to -4.6)
**Southern Latin America**	**51 921<br>(41 730 to 64 548)**	**150<br>(121 to 185)**	**12.1<br>(8.7 to 15.7)**	**97 904<br>(93 414 to 102 791)**	**264<br>(252 to 277)**	**14.0<br>(12.1 to 15.9)**	**13 242<br>(10 425 to 16 138)**	**36<br>(28 to 45)**	**12.5<br>(11.4 to 14.2)**
Argentina	35 238<br>(28 542 to 43 572)	154<br>(125 to 190)	14.2<br>(9.3 to 18.9)	66 108<br>(62 969 to 69 471)	273<br>(260 to 287)	17.0<br>(14.2 to 19.3)	9 018<br>(6 974 to 11 148)	38<br>(29 to 46)	15.3<br>(14.2 to 16.8)
Chile	13 585<br>(10 701 to 17 227)	140<br>(111 to 175)	6.6<br>(2.0 to 11.2)	25 591<br>(24 374 to 26 944)	239<br>(227 to 252)	8.3<br>(5.7 to 10.6)	3 379<br>(2 574 to 4 187)	32<br>(25 to 40)	7.5<br>(6.2 to 9.6)
Uruguay	3 096<br>(2 491 to 3 852)	158<br>(128 to 195)	11.6<br>(6.9 to 16.3)	6 199<br>(5 921 to 6 489)	280<br>(267 to 293)	13.6<br>(11.0 to 16.5)	843<br>(641 to 1 027)	39<br>(30 to 48)	12.5<br>(10.6 to 13.9)
**Central Europe, Eastern Europe, and Central Asia**	**1 136 442<br>(981 354 to 1 317 776)**	**489<br>(425 to 564)**	**-1.3<br>(-3.7 to 1.1)**	**3 008 989<br>(2 856 104 to 3 158 671)**	**1 105<br>(1 050 to 1 164)**	**3.0<br>(1.5 to 4.7)**	**436 971<br>(328 772 to 550 128)**	**163<br>(120 to 206)**	**4.5<br>(4.4 to 4.9)**
**Eastern Europe**	**602 435<br>(524 573 to 698 172)**	**514<br>(449 to 587)**	**1.8<br>(-1.2 to 5.1)**	**1 685 098<br>(1 601 086 to 1 768 575)**	**1 144<br>(1 084 to 1 208)**	**3.7<br>(1.1 to 6.2)**	**245 504<br>(180 788 to 313 374)**	**170<br>(125 to 217)**	**5.5<br>(4.6 to 6.0)**
Belarus	30 650<br>(26 345 to 36 025)	565<br>(491 to 655)	18.5<br>(14.2 to 23.4)	85 524<br>(80 659 to 90 008)	1 266<br>(1 193 to 1 335)	18.8<br>(14.3 to 23.9)	12 301<br>(8 913 to 15 331)	186<br>(134 to 238)	18.2<br>(17.2 to 20.0)
Estonia	3 635<br>(3 099 to 4 291)	490<br>(424 to 574)	-17.2<br>(-22.0 to -12.3)	11 007<br>(10 367 to 11 692)	1 140<br>(1 070 to 1 217)	-10.8<br>(-13.9 to -7.8)	1 590<br>(1 190 to 1 978)	169<br>(125 to 215)	-8.6<br>(-9.0 to -7.8)
Latvia	5 876<br>(5 052 to 6 885)	502<br>(437 to 578)	-20.7<br>(-25.3 to -16.3)	17 204<br>(16 211 to 18 150)	1 135<br>(1 068 to 1 205)	-14.8<br>(-17.9 to -11.7)	2 486<br>(1 839 to 3 098)	168<br>(121 to 216)	-12.8<br>(-13.5 to -12.3)
Lithuania	9 813<br>(8 380 to 11 588)	560<br>(482 to 651)	-4.7<br>(-9.6 to 0.1)	27 992<br>(26 439 to 29 522)	1 252<br>(1 185 to 1 327)	-1.9<br>(-4.6 to 0.7)	4 037<br>(3 040 to 5 079)	186<br>(135 to 234)	0.8<br>(-0.2 to 2.8)
Moldova	8 798<br>(7 568 to 10 216)	418<br>(363 to 484)	-15.5<br>(-19.9 to -10.4)	23 646<br>(22 119 to 25 258)	937<br>(876 to 1 003)	-9.9<br>(-13.8 to -6.0)	3 417<br>(2 423 to 4 377)	137<br>(97 to 178)	-7.4<br>(-8.7 to -6.6)
Russia	433 018<br>(376 456 to 499 735)	534<br>(466 to 609)	3.5<br>(-0.3 to 7.4)	1 191 602<br>(1 130 823 to 1 251 428)	1 181<br>(1 120 to 1 245)	5.3<br>(1.8 to 8.9)	173 785<br>(129 203 to 217 096)	176<br>(129 to 224)	7.0<br>(5.7 to 7.8)
Ukraine	110 645<br>(95 806 to 129 445)	448<br>(390 to 519)	-4.1<br>(-8.3 to -0.1)	328 123<br>(309 872 to 347 832)	1 015<br>(956 to 1 080)	-2.4<br>(-6.0 to 0.9)	47 904<br>(35 461 to 60 682)	152<br>(108 to 189)	-0.2<br>(-0.5 to 0.1)
**Central Europe**	**394 204<br>(331 940 to 473 228)**	**575<br>(493 to 675)**	**-2.5<br>(-6.0 to 0.9)**	**1 016 609<br>(957 986 to 1 074 668)**	**1 254<br>(1 181 to 1 328)**	**5.9<br>(4.3 to 7.8)**	**145 899<br>(109 044 to 182 946)**	**185<br>(139 to 236)**	**6.9<br>(6.5 to 7.5)**
Albania	6 661<br>(5 649 to 7 899)	443<br>(378 to 521)	19.6<br>(14.6 to 24.6)	17 507<br>(16 338 to 18 672)	1 027<br>(959 to 1 096)	22.2<br>(17.0 to 27.5)	2 534<br>(1 839 to 3 239)	151<br>(110 to 194)	22.1<br>(20.5 to 23.0)
Bosnia and Herzegovina	9 127<br>(7 646 to 10 879)	459<br>(390 to 544)	35.1<br>(30.7 to 39.8)	27 158<br>(25 218 to 29 186)	1 088<br>(1 012 to 1 168)	43.5<br>(36.6 to 51.3)	3 890<br>(2 940 to 4 887)	159<br>(119 to 198)	41.5<br>(40.9 to 42.2)
Bulgaria	20 614<br>(17 302 to 24 640)	514<br>(442 to 606)	4.8<br>(0.1 to 9.4)	60 376<br>(56 914 to 64 038)	1 160<br>(1 090 to 1 234)	7.8<br>(3.5 to 11.8)	8 657<br>(6 497 to 10 902)	171<br>(126 to 216)	8.3<br>(7.3 to 10.1)
Croatia	16 218<br>(13 565 to 19 586)	543<br>(468 to 638)	-5.2<br>(-12.1 to 1.5)	35 990<br>(34 021 to 38 082)	1 100<br>(1 041 to 1 157)	-5.1<br>(-8.7 to -1.2)	5 111<br>(3 711 to 6 449)	161<br>(117 to 206)	-3.8<br>(-5.2 to -2.5)
Czech Republic	45 361<br>(37 647 to 55 181)	719<br>(611 to 856)	-10.2<br>(-16.2 to -3.9)	116 034<br>(108 742 to 123 385)	1 560<br>(1 461 to 1 662)	6.4<br>(3.1 to 9.9)	16 754<br>(11 849 to 21 113)	231<br>(162 to 294)	9.0<br>(6.7 to 10.2)
Hungary	41 287<br>(33 973 to 51 033)	621<br>(526 to 741)	-20.7<br>(-26.4 to -14.9)	94 879<br>(88 896 to 100 954)	1 267<br>(1 189 to 1 349)	-9.3<br>(-12.6 to -5.4)	13 638<br>(10 090 to 17 338)	187<br>(134 to 240)	-6.9<br>(-8.4 to -6.0)
Macedonia	5 369<br>(4 473 to 6 394)	494<br>(419 to 588)	19.3<br>(13.7 to 25.4)	13 426<br>(12 542 to 14 359)	1 064<br>(992 to 1 144)	19.8<br>(15.4 to 24.7)	1 939<br>(1 427 to 2 428)	156<br>(113 to 194)	19.9<br>(18.7 to 22.3)
Montenegro	1 684<br>(1 436 to 1 996)	511<br>(438 to 597)	14.0<br>(9.9 to 18.3)	4 514<br>(4 234 to 4 798)	1 163<br>(1 090 to 1 237)	15.3<br>(11.6 to 20.0)	656<br>(481 to 836)	172<br>(126 to 222)	15.7<br>(15.3 to 16.0)
Poland	136 810<br>(114 205 to 165 215)	591<br>(504 to 695)	2.0<br>(-3.4 to 7.5)	347 499<br>(326 778 to 368 791)	1 281<br>(1 206 to 1 360)	11.2<br>(7.4 to 14.8)	49 768<br>(35 475 to 63 511)	188<br>(138 to 243)	11.3<br>(10.4 to 12.7)
Romania	57 975<br>(48 935 to 68 843)	533<br>(457 to 625)	-2.7<br>(-8.3 to 2.6)	161 903<br>(152 538 to 171 309)	1 196<br>(1 123 to 1 271)	0.8<br>(-2.5 to 4.6)	23 358<br>(16 673 to 30 069)	177<br>(125 to 227)	2.0<br>(1.7 to 2.2)
Serbia	24 360<br>(20 450 to 29 066)	500<br>(430 to 587)	15.7<br>(10.6 to 20.5)	66 442<br>(62 391 to 70 832)	1 128<br>(1 057 to 1 205)	19.3<br>(14.9 to 24.4)	9 534<br>(7 017 to 12 260)	166<br>(121 to 210)	17.8<br>(16.2 to 20.8)

Location	Incidence (95% UI)			Prevalence (95% UI)			YLDs (95% UI)		
	2016 counts	2016 age-standardised rates per 100,000	Percentage change in age-standardised rates between 1990 and 2016	2016 counts	2016 age-standardised rates per 100,000	Percentage change in age-standardised rates between 1990 and 2016	2016 counts	2016 age-standardised rates per 100,000	Percentage change in age-standardised rates between 1990 and 2016
Slovakia	17 719 (14 867 to 21 353)	570 (483 to 668)	-11.9 (-16.8 to -6.8)	45 248 (42 471 to 47 874)	1 237 (1 158 to 1 311)	-3.3 (-6.7 to 0.6)	6 477 (4 763 to 8 301)	181 (129 to 234)	-2.6 (-4.3 to -1.6)
Slovenia	11 022 (8 933 to 13 825)	789 (657 to 964)	0.3 (-7.6 to 10.8)	25 631 (24 025 to 27 325)	1 639 (1 542 to 1 739)	5.0 (1.9 to 8.5)	3 621 (2 713 to 4 531)	240 (172 to 302)	6.8 (6.0 to 8.4)
**Central Asia**	**139 803 (121 531 to 161 726)**	**314 (272 to 362)**	**4.4 (1.7 to 6.9)**	**307 283 (290 504 to 325 770)**	**720 (680 to 761)**	**5.9 (3.6 to 8.0)**	**45 137 (33 402 to 57 015)**	**106 (76 to 133)**	**7.2 (7.1 to 7.6)**
Armenia	4 943 (4 255 to 5 754)	301 (260 to 349)	-8.2 (-12.8 to -3.4)	16 447 (14 057 to 19 904)	863 (735 to 1 047)	-12.6 (-19.1 to -6.1)	2 396 (1 792 to 3 012)	127 (95 to 161)	-10.7 (-12.9 to -7.0)
Azerbaijan	15 053 (13 022 to 17 428)	309 (268 to 358)	5.1 (0.7 to 9.3)	36 473 (34 077 to 38 772)	711 (665 to 756)	9.7 (5.7 to 15.0)	5 296 (3 827 to 6 750)	104 (75 to 130)	9.8 (7.6 to 11.0)
Georgia	6 526 (5 619 to 7 562)	294 (255 to 341)	-5.3 (-9.2 to -1.6)	17 447 (16 400 to 18 510)	670 (629 to 715)	-3.5 (-8.0 to 1.5)	2 500 (1 782 to 3 211)	98 (71 to 127)	-3.6 (-3.9 to -3.3)
Kazakhstan	36 037 (31 354 to 41 515)	390 (339 to 449)	14.9 (10.5 to 19.3)	81 246 (76 696 to 86 289)	873 (825 to 926)	16.3 (11.5 to 21.0)	11 967 (8 315 to 15 491)	128 (88 to 164)	18.7 (18.1 to 20.0)
Kyrgyzstan	9 126 (7 928 to 10 564)	303 (263 to 349)	-5.7 (-9.7 to -1.7)	18 295 (17 071 to 19 471)	686 (643 to 727)	-2.2 (-6.3 to 2.0)	2 695 (1 941 to 3 431)	100 (74 to 128)	-0.4 (-1.4 to 1.1)
Mongolia	5 584 (4 844 to 6 454)	369 (320 to 427)	37.3 (29.2 to 44.8)	10 977 (10 312 to 11 696)	801 (754 to 850)	44.2 (37.2 to 51.1)	1 616 (1 115 to 2 087)	117 (83 to 153)	44.2 (42.8 to 45.1)
Tajikistan	10 827 (9 265 to 12 709)	259 (222 to 303)	-4.0 (-8.1 to -0.3)	19 974 (18 489 to 21 751)	591 (551 to 636)	2.1 (-3.0 to 7.8)	2 976 (2 154 to 3 833)	87 (63 to 113)	2.6 (1.3 to 3.4)
Turkmenistan	7 853 (6 817 to 9 111)	287 (249 to 332)	4.3 (0.4 to 8.6)	16 122 (15 000 to 17 264)	641 (599 to 682)	8.1 (2.9 to 12.7)	2 384 (1 732 to 3 046)	94 (69 to 121)	9.0 (8.3 to 10.2)
Uzbekistan	43 854 (37 958 to 50 914)	285 (247 to 332)	6.4 (2.5 to 10.4)	90 303 (84 579 to 96 734)	639 (599 to 681)	8.3 (3.8 to 13.7)	13 287 (9 511 to 17 319)	94 (66 to 119)	9.5 (8.6 to 11.1)
**Latin America and Caribbean**	**522 893 (453 440 to 608 627)**	**191 (165 to 222)**	**-1.5 (-4.7 to 1.9)**	**1 054 906 (989 423 to 1 113 950)**	**381 (358 to 403)**	**-2.8 (-4.6 to -0.8)**	**153 221 (112 496 to 190 944)**	**56 (41 to 70)**	**-2.8 (-3.3 to -2.0)**
**Central Latin America**	**201 746 (174 300 to 234 974)**	**172 (148 to 202)**	**-4.4 (-7.4 to -1.5)**	**400 248 (375 596 to 424 312)**	**344 (323 to 364)**	**-0.7 (-2.3 to 1.1)**	**58 780 (42 384 to 74 779)**	**51 (37 to 64)**	**-1.7 (-2.0 to -1.2)**
Colombia	38 882 (33 253 to 45 426)	167 (144 to 197)	-3.3 (-8.2 to 2.2)	85 717 (79 680 to 91 575)	355 (331 to 378)	2.6 (-2.0 to 7.0)	12 569 (8 964 to 16 040)	52 (38 to 66)	3.5 (2.9 to 4.0)
Costa Rica	4 347 (3 672 to 5 157)	185 (156 to 219)	16.3 (10.1 to 22.4)	9 369 (8 722 to 10 078)	375 (349 to 403)	24.3 (18.9 to 30.7)	1 359 (1 008 to 1 712)	55 (41 to 69)	22.5 (21.3 to 24.4)
El Salvador	5 860 (5 069 to 6 847)	190 (164 to 222)	11.0 (-3.6 to 20.4)	12 224 (11 282 to 13 362)	389 (360 to 424)	16.7 (8.2 to 23.7)	1 769 (1 276 to 2 232)	57 (42 to 72)	16.0 (11.6 to 19.7)
Guatemala	11 844 (10 211 to 13 865)	167 (142 to 197)	19.8 (8.7 to 27.8)	20 367 (18 846 to 21 908)	319 (297 to 342)	27.7 (21.7 to 34.8)	2 966 (2 157 to 3 744)	46 (34 to 58)	24.6 (22.7 to 26.1)
Honduras	6 072 (5 187 to 7 149)	181 (153 to 214)	57.4 (47.9 to 68.0)	11 246 (10 116 to 12 734)	344 (314 to 380)	58.0 (47.2 to 72.6)	1 656 (1 198 to 2 108)	50 (38 to 64)	53.0 (50.0 to 55.1)
Mexico	99 755 (85 950 to 117 419)	171 (147 to 202)	-12.7 (-16.1 to -9.3)	189 648 (178 239 to 200 839)	327 (308 to 346)	-11.4 (-13.3 to -9.3)	27 939 (20 144 to 35 519)	48 (36 to 62)	-13.0 (-13.4 to -12.3)
Nicaragua	4 370 (3 724 to 5 151)	160 (136 to 190)	12.7 (7.3 to 18.6)	8 829 (8 008 to 9 815)	334 (306 to 367)	6.8 (-0.8 to 14.1)	1 290 (953 to 1 634)	49 (36 to 63)	7.6 (6.0 to 9.5)
Panama	3 144 (2 710 to 3 695)	164 (141 to 193)	10.0 (5.2 to 15.3)	6 541 (6 079 to 6 966)	346 (322 to 369)	15.9 (10.9 to 21.4)	953 (673 to 1 231)	51 (36 to 65)	14.6 (13.9 to 15.8)
Venezuela	27 472 (23 666 to 32 062)	187 (160 to 220)	10.3 (5.4 to 15.2)	56 307 (52 596 to 60 047)	389 (365 to 415)	18.1 (13.1 to 23.3)	8 265 (6 106 to 10 553)	57 (41 to 72)	17.4 (15.9 to 20.3)
**Andean Latin America**	**50 046 (43 756 to 57 393)**	**176 (153 to 202)**	**10.4 (6.1 to 15.0)**	**97 649 (91 505 to 103 178)**	**365 (344 to 385)**	**12.8 (10.2 to 16.0)**	**14 255 (10 294 to 17 891)**	**53 (39 to 68)**	**12.3 (10.6 to 13.3)**
Bolivia	8 758 (7 634 to 10 033)	169 (147 to 194)	-2.4 (-6.0 to 1.1)	15 903 (14 877 to 16 907)	340 (320 to 361)	2.8 (-0.9 to 6.3)	2 315 (1 632 to 2 965)	49 (36 to 63)	2.3 (0.7 to 3.3)
Ecuador	15 339 (13 167 to 18 031)	195 (169 to 230)	26.1 (15.0 to 41.6)	28 006 (26 279 to 29 765)	379 (357 to 402)	16.3 (13.0 to 19.7)	4 065 (2 960 to 5 173)	55 (41 to 70)	14.6 (12.9 to 17.4)
Peru	25 949 (22 599 to 29 785)	168 (146 to 193)	7.1 (0.5 to 13.1)	53 740 (50 114 to 57 107)	366 (342 to 388)	14.3 (9.9 to 19.9)	7 874 (5 450 to 10 188)	54 (38 to 70)	14.2 (11.8 to 15.6)
**Caribbean**	**45 421 (39 287 to 52 918)**	**195 (169 to 227)**	**22.9 (18.3 to 28.2)**	**99 799 (88 166 to 115 494)**	**425 (375 to 493)**	**35.2 (22.1 to 56.4)**	**14 275 (10 338 to 18 449)**	**61 (45 to 78)**	**32.2 (31.5 to 32.7)**
Antigua and Barbuda	79 (69 to 92)	170 (149 to 196)	17.2 (14.0 to 20.6)	180 (168 to 192)	372 (348 to 395)	20.2 (15.6 to 24.7)	26 (18 to 34)	55 (39 to 71)	19.0 (15.8 to 20.9)
The Bahamas	371 (324 to 427)	186 (162 to 213)	8.0 (4.0 to 12.2)	817 (767 to 862)	386 (362 to 407)	10.9 (7.1 to 15.0)	119 (88 to 154)	57 (40 to 72)	10.8 (10.2 to 11.5)
Barbados	269 (233 to 316)	167 (145 to 193)	19.0 (15.4 to 22.7)	659 (615 to 701)	357 (333 to 380)	20.2 (16.2 to 24.7)	95 (70 to 120)	53 (37 to 67)	19.3 (15.7 to 21.3)
Belize	290 (254 to 330)	174 (153 to 198)	25.8 (19.6 to 30.7)	513 (482 to 541)	354 (334 to 373)	27.6 (23.4 to 33.1)	76 (55 to 97)	52 (37 to 65)	24.3 (21.8 to 25.6)
Bermuda	58 (50 to 68)	167 (145 to 195)	10.5 (6.4 to 14.7)	137 (127 to 146)	377 (349 to 400)	21.3 (16.8 to 25.7)	20 (14 to 26)	56 (39 to 72)	20.4 (17.7 to 22.1)
Cuba	16 049 (13 361 to 19 517)	229 (193 to 274)	22.1 (15.2 to 30.7)	32 617 (30 191 to 35 258)	437 (405 to 469)	19.7 (14.1 to 25.3)	4 630 (3 301 to 5 793)	64 (46 to 81)	18.3 (17.7 to 19.2)
Dominica	56 (49 to 65)	152 (132 to 176)	24.1 (20.8 to 27.8)	127 (118 to 136)	334 (310 to 356)	30.3 (24.9 to 37.1)	19 (13 to 24)	50 (34 to 65)	30.2 (25.5 to 33.1)
Dominican Republic	8 650 (7 547 to 9 969)	171 (150 to 197)	27.3 (23.1 to 31.8)	17 899 (16 773 to 18 877)	374 (351 to 394)	32.8 (28.6 to 37.8)	2 624 (1 896 to 3 320)	55 (39 to 70)	30.7 (29.4 to 33.2)
Grenada	82 (72 to 95)	165 (143 to 190)	26.6 (22.3 to 30.7)	171 (159 to 182)	340 (316 to 363)	28.6 (23.4 to 34.8)	25 (17 to 33)	50 (37 to 64)	28.3 (27.5 to 29.0)
Guyana	585 (509 to 673)	165 (144 to 190)	8.7 (4.4 to 12.8)	1 091 (1 017 to 1 160)	314 (294 to 332)	12.6 (7.6 to 16.6)	159 (111 to 205)	46 (33 to 58)	10.7 (9.9 to 12.1)
Haiti	8 624 (7 285 to 10 342)	162 (137 to 192)	17.4 (6.5 to 34.2)	21 789 (12 873 to 36 914)	442 (278 to 715)	85.4 (18.9 to 197.2)	3 016 (1 690 to 4 325)	61 (37 to 85)	76.4 (47.6 to 93.2)
Jamaica	2 137 (1 865 to 2 465)	151 (131 to 175)	26.7 (23.1 to 30.4)	4 524 (4 208 to 4 847)	319 (297 to 342)	27.3 (21.7 to 32.2)	658 (469 to 840)	47 (34 to 61)	25.5 (24.7 to 26.2)
Puerto Rico	4 319 (3 682 to 5 116)	202 (174 to 236)	25.1 (19.3 to 31.6)	10 436 (9 718 to 11 157)	433 (403 to 462)	25.2 (19.8 to 30.3)	1 525 (1 084 to 1 988)	65 (47 to 83)	25.1 (24.5 to 26.2)
Saint Lucia	149 (130 to 172)	166 (145 to 191)	18.6 (14.9 to 22.7)	349 (326 to 370)	359 (336 to 381)	24.0 (19.1 to 29.2)	51 (36 to 66)	53 (36 to 68)	23.5 (19.6 to 25.7)
Saint Vincent and the Grenadines	85 (74 to 98)	160 (140 to 184)	24.3 (20.8 to 28.4)	179 (167 to 189)	333 (312 to 352)	27.5 (22.9 to 32.5)	26 (18 to 34)	49 (35 to 63)	26.8 (25.9 to 28.3)
Suriname	472 (416 to 537)	179 (158 to 204)	18.4 (14.2 to 22.7)	969 (909 to 1 031)	368 (345 to 391)	20.6 (16.9 to 24.6)	141 (105 to 179)	54 (40 to 67)	19.7 (18.3 to 22.3)
Trinidad and Tobago	1 111 (968 to 1 281)	167 (146 to 191)	17.0 (11.8 to 22.1)	2 700 (2 522 to 2 865)	359 (336 to 381)	27.5 (22.9 to 32.5)	390 (280 to 511)	53 (38 to 68)	27.1 (26.6 to 27.5)
Virgin Islands, U.S.	116 (100 to 134)	182 (160 to 210)	14.9 (10.9 to 18.9)	281 (263 to 300)	386 (361 to 410)	18.4 (14.8 to 22.8)	40 (30 to 50)	57 (41 to 73)	17.0 (16.5 to 17.9)
**Tropical Latin America**	**225 679 (195 625 to 263 184)**	**213 (185 to 249)**	**-6.1 (-10.6 to -0.8)**	**457 211 (430 560 to 482 724)**	**415 (390 to 438)**	**-12.5 (-15.4 to -10.1)**	**65 861 (47 376 to 84 414)**	**60 (44 to 77)**	**-11.6 (-11.9 to -10.9)**
Brazil	220 229 (190 834 to 257 061)	214 (186 to 250)	-6.9 (-11.5 to -1.6)	446 626 (420 736 to 471 555)	416 (391 to 439)	-13.2 (-16.1 to -10.7)	64 320 (45 485 to 83 367)	61 (44 to 77)	-12.2 (-12.5 to -11.7)
Paraguay	5 450 (4 689 to 6 367)	182 (157 to 213)	34.6 (29.3 to 40.4)	10 585 (9 790 to 11 371)	372 (346 to 398)	28.6 (22.6 to 36.1)	1 539 (1 092 to 1 968)	54 (38 to 69)	25.7 (24.8 to 27.4)
**Southeast Asia, East Asia, and Oceania**	**2 261 373 (2 009 035 to 2 528 664)**	**221 (197 to 246)**	**26.9 (22.8 to 30.6)**	**6 167 420 (5 923 691 to 6 420 978)**	**544 (522 to 567)**	**37.4 (35.1 to 39.8)**	**895 254 (616 036 to 1 127 204)**	**80 (58 to 102)**	**33.8 (33.3 to 34.6)**
**East Asia**	**1 588 870 (1 409 873 to 1 777 238)**	**229 (204 to 256)**	**30.6 (26.2 to 34.8)**	**4 598 758 (4 421 995 to 4 779 751)**	**565 (543 to 588)**	**38.8 (36.4 to 41.3)**	**665 234 (484 647 to 839 897)**	**83 (60 to 106)**	**35.2 (34.4 to 36.6)**
China	1 536 840 (1 363 520 to 1 717 731)	230 (205 to 257)	30.3 (25.8 to 34.5)	4 452 845 (4 282 684 to 4 628 904)	568 (546 to 590)	38.7 (36.2 to 41.2)	644 017 (459 933 to 829 158)	83 (60 to 106)	35.1 (34.3 to 36.6)
North Korea	26 155 (23 227 to 29 269)	191 (169 to 214)	53.3 (46.4 to 61.4)	63 819 (61 192 to 66 774)	443 (424 to 464)	48.5 (44.1 to 53.4)	9 289 (6 747 to 11 858)	65 (48 to 81)	43.7 (41.3 to 45.1)
Taiwan (Province of China)	25 875 (22 983 to 29 045)	221 (196 to 249)	28.4 (23.0 to 34.7)	82 094 (78 357 to 85 756)	557 (531 to 585)	34.9 (31.2 to 38.5)	11 929 (8 345 to 15 330)	82 (58 to 107)	32.3 (31.9 to 33.0)
**Southeast Asia**	**661 234 (589 645 to 738 826)**	**205 (183 to 230)**	**19.4 (14.1 to 23.6)**	**1 548 655 (1 477 233 to 1 631 274)**	**489 (467 to 514)**	**33.7 (31.1 to 36.8)**	**226 696 (168 899 to 285 044)**	**72 (53 to 91)**	**30.1 (28.9 to 30.9)**
Cambodia	15 421 (13 659 to 17 224)	197 (175 to 221)	25.8 (7.0 to 37.2)	32 237 (29 867 to 36 094)	465 (431 to 522)	32.8 (14.6 to 46.3)	4 715 (3 379 to 6 052)	67 (51 to 86)	31.1 (26.1 to 37.3)
Indonesia	249 952 (221 371 to 280 110)	201 (179 to 225)	12.5 (8.8 to 16.2)	557 628 (531 432 to 589 634)	462 (441 to 487)	22.4 (19.8 to 25.9)	82 040 (59 737 to 102 765)	68 (49 to 87)	19.7 (18.7 to 20.2)
Laos	6 690 (5 893 to 7 491)	183 (161 to 205)	23.4 (-0.9 to 38.2)	11 647 (11 046 to 12 314)	412 (393 to 432)	56.1 (51.8 to 60.2)	1 739 (1 256 to 2 268)	60 (44 to 75)	52.6 (51.2 to 54.9)
Malaysia	34 417 (30 369 to 38 656)	225 (199 to 251)	32.9 (28.1 to 38.8)	80 288 (76 407 to 84 698)	543 (518 to 571)	39.1 (35.4 to 43.1)	11 707 (8 170 to 14 965)	79 (58 to 104)	34.3 (33.6 to 34.9)
Maldives	269 (238 to 302)	153 (136 to 170)	1.7 (-2.1 to 6.0)	614 (582 to 647)	379 (361 to 398)	22.9 (19.7 to 26.5)	90 (67 to 114)	55 (42 to 71)	22.2 (21.5 to 23.0)
Mauritius	1 122 (999 to 1 258)	181 (161 to 202)	38.7 (33.4 to 44.0)	3 198 (3 061 to 3 346)	441 (422 to 463)	43.7 (39.9 to 47.4)	461 (337 to 585)	64 (47 to 82)	40.1 (39.5 to 40.8)
Myanmar	46 124 (40 897 to 51 660)	173 (153 to 194)	34.3 (29.1 to 40.0)	113 961 (103 828 to 129 669)	430 (394 to 484)	66.8 (55.0 to 84.6)	16 605 (11 596 to 21 411)	63 (45 to 81)	63.7 (57.9 to 67.3)
Philippines	93 114 (83 325 to 104 508)	184 (164 to 206)	31.7 (21.5 to 41.5)	186 945 (177 383 to 197 613)	420 (399 to 441)	43.9 (39.0 to 48.8)	27 505 (19 739 to 34 946)	61 (46 to 77)	38.8 (38.4 to 39.0)
Sri Lanka	22 736 (20 244 to 25 441)	213 (190 to 238)	-10.2 (-43.5 to 18.0)	63 398 (58 864 to 70 483)	554 (514 to 617)	48.3 (40.3 to 59.3)	9 101 (6 691 to 11 478)	80 (60 to 104)	42.3 (41.1 to 44.3)
Seychelles	106 (94 to 118)	220 (196 to 244)	42.6 (37.2 to 48.5)	258 (246 to 270)	518 (494 to 542)	45.2 (41.8 to 49.0)	37 (29 to 47)	76 (56 to 96)	39.6 (37.1 to 43.7)
Thailand	86 711 (76 212 to 98 402)	249 (219 to 280)	15.9 (10.8 to 21.0)	249 628 (239 460 to 260 867)	606 (580 to 634)	23.3 (20.6 to 26.2)	36 468 (25 708 to 46 607)	90 (66 to 112)	20.9 (19.9 to 21.5)
Timor-Leste	959 (843 to 1 074)	169 (150 to 190)	-15.8 (-51.9 to 19.8)	2 051 (1 785 to 2 550)	475 (413 to 597)	46.2 (34.8 to 55.5)	300 (215 to 386)	68 (49 to 86)	41.3 (36.9 to 47.6)
Vietnam	102 679 (90 844 to 115 395)	217 (192 to 243)	39.1 (34.3 to 44.1)	244 383 (233 427 to 256 740)	503 (480 to 528)	47.8 (43.0 to 52.2)	35 426 (25 705 to 45 508)	74 (55 to 94)	42.8 (41.3 to 43.8)

Location	Incidence (95% UI)			Prevalence (95% UI)			YLDs (95% UI)		
	2016 counts	2016 age-standardised rates per 100,000	Percentage change in age-standardised rates between 1990 and 2016	2016 counts	2016 age-standardised rates per 100,000	Percentage change in age-standardised rates between 1990 and 2016	2016 counts	2016 age-standardised rates per 100,000	Percentage change in age-standardised rates between 1990 and 2016
**Oceania**	**11 269 (10 022 to 12 618)**	**207 (184 to 233)**	**35.8 (30.3 to 41.2)**	**20 007 (19 003 to 21 059)**	**436 (416 to 457)**	**43.6 (40.8 to 46.8)**	**2 918 (2 130 to 3 671)**	**63 (45 to 78)**	**39.0 (37.5 to 42.0)**
American Samoa	84 (75 to 94)	214 (191 to 238)	27.0 (22.9 to 31.4)	176 (167 to 187)	499 (475 to 526)	26.7 (23.2 to 30.0)	26 (19 to 33)	73 (53 to 92)	24.7 (24.2 to 25.6)
Federated States of Micronesia	97 (86 to 109)	197 (176 to 222)	34.0 (29.3 to 38.8)	180 (171 to 190)	427 (407 to 450)	36.7 (33.0 to 40.5)	26 (20 to 32)	62 (47 to 78)	34.2 (31.2 to 39.1)
Fiji	787 (704 to 877)	191 (172 to 212)	45.4 (40.3 to 51.5)	1 789 (1 705 to 1 887)	424 (405 to 446)	46.5 (42.1 to 50.3)	261 (196 to 330)	62 (44 to 77)	43.3 (41.3 to 44.5)
Guam	209 (187 to 234)	243 (217 to 273)	36.9 (31.4 to 42.6)	503 (481 to 527)	576 (552 to 603)	36.8 (34.0 to 40.3)	73 (51 to 94)	84 (61 to 107)	32.4 (30.8 to 33.5)
Kiribati	99 (89 to 111)	169 (151 to 190)	39.4 (35.3 to 44.3)	182 (172 to 193)	364 (346 to 384)	46.3 (42.6 to 50.2)	26 (19 to 34)	52 (37 to 68)	42.4 (37.9 to 45.2)
Marshall Islands	69 (61 to 77)	185 (165 to 207)	37.7 (32.3 to 43.4)	120 (114 to 127)	397 (378 to 418)	34.6 (31.0 to 38.3)	18 (12 to 23)	57 (42 to 72)	30.5 (28.6 to 34.2)
Northern Mariana Islands	153 (137 to 174)	235 (211 to 262)	11.0 (7.2 to 15.1)	278 (263 to 294)	550 (524 to 575)	12.6 (10.1 to 15.2)	42 (30 to 55)	81 (58 to 104)	10.9 (9.1 to 14.2)
Papua New Guinea	7 991 (7 092 to 8 948)	212 (188 to 238)	34.2 (27.6 to 40.2)	13 335 (12 637 to 14 059)	435 (415 to 457)	45.2 (41.8 to 49.2)	1 945 (1 422 to 2 420)	63 (46 to 80)	40.2 (37.9 to 44.1)
Samoa	186 (165 to 208)	192 (171 to 215)	36.6 (32.0 to 41.9)	359 (341 to 381)	452 (430 to 478)	41.8 (36.9 to 46.9)	53 (38 to 67)	66 (48 to 84)	36.4 (34.8 to 39.1)
Solomon Islands	566 (504 to 636)	194 (173 to 217)	42.4 (36.7 to 48.5)	930 (880 to 982)	403 (384 to 422)	44.8 (41.1 to 48.5)	137 (103 to 172)	58 (43 to 74)	40.0 (38.0 to 41.1)
Tonga	106 (94 to 118)	197 (176 to 219)	13.2 (8.6 to 17.7)	200 (190 to 211)	439 (419 to 461)	21.0 (18.3 to 24.6)	29 (21 to 37)	64 (46 to 81)	19.5 (19.0 to 19.9)
Vanuatu	266 (235 to 300)	192 (169 to 215)	46.2 (40.2 to 52.3)	469 (445 to 498)	418 (398 to 441)	50.9 (46.5 to 55.1)	69 (53 to 86)	61 (46 to 76)	46.8 (45.3 to 49.1)
**North Africa and Middle East**	**822 906 (657 986 to 1 163 044)**	**285 (230 to 387)**	**14.6 (-2.9 to 51.7)**	**1 383 717 (1 277 564 to 1 545 776)**	**555 (517 to 614)**	**2.0 (-0.0 to 4.5)**	**201 515 (152 455 to 247 733)**	**80 (61 to 101)**	**2.2 (1.9 to 2.9)**
**North Africa and Middle East**	**822 906 (657 986 to 1 163 044)**	**285 (230 to 387)**	**14.6 (-2.9 to 51.7)**	**1 383 717 (1 277 564 to 1 545 776)**	**555 (517 to 614)**	**2.0 (-0.0 to 4.5)**	**201 515 (152 455 to 247 733)**	**80 (61 to 101)**	**2.2 (1.9 to 2.9)**
Afghanistan	70 617 (39 322 to 146 804)	366 (214 to 723)	58.3 (-0.5 to 169.1)	74 075 (55 528 to 111 535)	607 (437 to 941)	-7.8 (-20.2 to 11.7)	10 506 (6 876 to 13 922)	84 (55 to 113)	-7.5 (-10.8 to -1.2)
Algeria	44 698 (39 334 to 50 447)	226 (199 to 254)	-6.4 (-9.1 to -3.5)	95 142 (89 297 to 101 122)	520 (490 to 550)	-1.7 (-5.3 to 2.4)	13 907 (9 870 to 17 694)	76 (56 to 97)	-0.9 (-1.8 to -0.3)
Bahrain	1 309 (1 152 to 1 480)	248 (218 to 280)	-5.8 (-9.4 to -2.1)	2 909 (2 721 to 3 098)	597 (561 to 631)	0.8 (-3.0 to 5.0)	426 (308 to 545)	87 (64 to 110)	0.2 (-1.2 to 2.8)
Egypt	95 079 (84 071 to 107 567)	206 (182 to 232)	22.1 (18.1 to 26.9)	185 347 (174 903 to 196 519)	459 (434 to 484)	20.9 (16.1 to 25.8)	27 326 (19 561 to 35 551)	67 (49 to 84)	20.1 (18.4 to 21.0)
Iran	96 644 (85 871 to 109 253)	244 (217 to 275)	-34.0 (-52.7 to -13.4)	234 767 (217 946 to 258 930)	619 (578 to 677)	-7.8 (-12.6 to -3.7)	34 466 (26 200 to 41 931)	91 (70 to 111)	-5.1 (-7.0 to -2.4)
Iraq	91 768 (56 040 to 174 433)	414 (270 to 764)	57.1 (4.5 to 180.9)	109 110 (87 769 to 151 887)	754 (614 to 1 041)	-2.5 (-10.4 to 5.1)	15 637 (10 544 to 21 414)	106 (71 to 141)	-5.4 (-8.6 to -0.2)
Jordan	9 777 (7 779 to 13 505)	241 (195 to 325)	9.9 (-6.0 to 49.7)	14 923 (13 924 to 15 912)	478 (450 to 505)	-4.4 (-8.3 to -0.3)	2 191 (1 530 to 2 828)	69 (51 to 89)	-3.4 (-6.2 to -1.6)
Kuwait	4 257 (3 753 to 4 780)	256 (226 to 287)	-30.8 (-55.1 to -9.8)	9 775 (9 171 to 10 396)	619 (585 to 653)	-6.1 (-10.9 to -2.3)	1 456 (1 100 to 1 866)	91 (68 to 114)	-5.4 (-6.1 to -4.6)
Lebanon	6 838 (5 803 to 8 249)	247 (209 to 301)	-37.9 (-59.1 to -15.6)	21 797 (17 436 to 31 121)	766 (612 to 1 104)	-16.0 (-27.3 to -3.0)	3 101 (2 065 to 4 172)	109 (70 to 146)	-12.0 (-15.0 to -6.9)
Libya	8 842 (6 667 to 13 156)	293 (222 to 431)	30.6 (1.9 to 102.1)	16 728 (14 908 to 20 026)	579 (520 to 687)	8.0 (-1.3 to 25.0)	2 417 (1 771 to 3 070)	84 (64 to 105)	6.7 (2.0 to 11.7)
Morocco	33 783 (29 787 to 38 104)	205 (181 to 232)	3.7 (0.7 to 6.8)	80 803 (75 658 to 85 753)	479 (450 to 507)	9.0 (5.3 to 14.5)	11 812 (8 726 to 14 839)	70 (51 to 89)	8.9 (8.5 to 9.5)
Palestine	5 860 (5 135 to 6 722)	199 (176 to 227)	-7.7 (-23.4 to 2.8)	9 299 (8 233 to 10 914)	503 (444 to 606)	-4.9 (-12.6 to 2.0)	1 382 (989 to 1 759)	73 (53 to 94)	-4.1 (-6.5 to -0.0)
Oman	4 588 (4 020 to 5 198)	288 (254 to 325)	-12.0 (-15.6 to -8.2)	8 618 (8 136 to 9 090)	685 (652 to 718)	-9.0 (-12.4 to -5.8)	1 290 (899 to 1 642)	101 (72 to 129)	-7.4 (-8.2 to -6.8)
Qatar	1 771 (1 562 to 2 001)	276 (244 to 310)	-2.9 (-5.8 to 0.2)	3 753 (3 520 to 3 987)	685 (649 to 721)	2.2 (-1.1 to 5.5)	568 (398 to 731)	101 (72 to 132)	3.7 (2.3 to 4.8)
Saudi Arabia	32 988 (29 087 to 37 073)	241 (213 to 271)	-14.2 (-16.3 to -12.0)	66 296 (63 007 to 69 800)	569 (543 to 595)	-12.4 (-14.0 to -11.0)	9 835 (7 271 to 12 420)	84 (62 to 105)	-10.2 (-11.4 to -9.6)
Sudan	44 257 (37 978 to 53 625)	213 (184 to 256)	7.2 (0.0 to 15.2)	73 118 (67 893 to 79 007)	456 (427 to 488)	14.9 (11.5 to 18.6)	10 682 (8 074 to 13 477)	66 (50 to 81)	14.2 (13.4 to 15.3)
Syria	89 753 (30 844 to 222 484)	894 (322 to 2 128)	378.2 (72.9 to 1 102.0)	53 444 (40 449 to 79 073)	654 (518 to 929)	53.2 (23.5 to 109.1)	7 325 (4 882 to 9 789)	90 (62 to 118)	44.6 (23.4 to 66.0)
Tunisia	12 872 (11 377 to 14 497)	236 (208 to 266)	3.6 (0.3 to 7.8)	31 844 (29 847 to 33 732)	544 (511 to 576)	7.6 (3.5 to 12.9)	4 666 (3 461 to 6 005)	80 (59 to 103)	8.4 (8.1 to 8.7)
Turkey	92 011 (81 328 to 104 858)	233 (206 to 266)	-12.2 (-16.5 to -6.1)	221 763 (209 060 to 235 982)	543 (512 to 578)	-10.2 (-14.9 to -5.5)	32 365 (24 105 to 40 489)	80 (60 to 99)	-9.5 (-9.9 to -9.0)
United Arab Emirates	8 200 (7 241 to 9 296)	300 (264 to 340)	-10.3 (-13.1 to -7.3)	15 583 (14 643 to 16 583)	718 (681 to 754)	-7.7 (-11.2 to -4.5)	2 319 (1 631 to 3 032)	106 (75 to 136)	-6.8 (-9.0 to -5.4)
Yemen	66 215 (41 408 to 129 206)	410 (270 to 757)	93.1 (27.8 to 253.5)	53 203 (48 208 to 61 204)	494 (459 to 548)	9.6 (4.3 to 18.3)	7 571 (5 682 to 9 785)	70 (53 to 87)	8.8 (7.3 to 11.0)
**South Asia**	**2 517 684 (2 210 913 to 2 830 957)**	**332 (292 to 376)**	**-0.3 (-2.5 to 1.7)**	**4 532 422 (4 324 994 to 4 740 725)**	**625 (598 to 653)**	**12.4 (11.2 to 13.6)**	**659 654 (481 279 to 838 287)**	**91 (68 to 115)**	**12.1 (11.2 to 12.8)**
**South Asia**	**2 517 684 (2 210 913 to 2 830 957)**	**332 (292 to 376)**	**-0.3 (-2.5 to 1.7)**	**4 532 422 (4 324 994 to 4 740 725)**	**625 (598 to 653)**	**12.4 (11.2 to 13.6)**	**659 654 (481 279 to 838 287)**	**91 (68 to 115)**	**12.1 (11.2 to 12.8)**
Bangladesh	196 719 (172 580 to 223 709)	259 (228 to 295)	20.0 (15.8 to 24.1)	360 889 (339 211 to 383 308)	520 (490 to 550)	37.2 (31.5 to 42.9)	52 669 (38 404 to 67 399)	76 (55 to 94)	24.4 (22.7 to 26.3)
Bhutan	1 050 (921 to 1 192)	309 (271 to 352)	-2.6 (-5.9 to 1.0)	1 894 (1 782 to 2 003)	626 (593 to 656)	12.3 (8.5 to 15.6)	274 (202 to 352)	90 (66 to 114)	12.1 (11.1 to 12.8)
India	2 024 670 (1 781 627 to 2 280 404)	347 (305 to 393)	-3.6 (-5.8 to -1.5)	3 663 374 (3 496 494 to 3 832 047)	643 (615 to 673)	8.9 (7.9 to 10.0)	532 099 (392 621 to 666 820)	93 (70 to 116)	9.5 (8.5 to 10.2)
Nepal	42 131 (36 898 to 47 918)	292 (255 to 332)	5.7 (2.0 to 9.5)	71 817 (67 822 to 76 330)	562 (534 to 595)	22.2 (17.5 to 26.9)	10 787 (8 078 to 13 927)	83 (59 to 107)	24.9 (22.4 to 26.3)
Pakistan	253 115 (222 181 to 286 600)	276 (243 to 310)	23.2 (19.4 to 26.8)	434 448 (409 220 to 459 150)	571 (541 to 600)	33.2 (28.7 to 38.4)	63 737 (45 923 to 82 435)	83 (61 to 104)	31.8 (29.2 to 36.5)
**Sub-Saharan Africa**	**1 002 906 (885 983 to 1 130 857)**	**219 (194 to 248)**	**-12.9 (-22.8 to -7.8)**	**1 467 455 (1 387 515 to 1 557 679)**	**424 (404 to 445)**	**-1.2 (-2.3 to 0.1)**	**218 379 (161 958 to 273 841)**	**62 (46 to 78)**	**-0.5 (-1.0 to -0.1)**
**Southern sub-Saharan Africa**	**87 189 (76 948 to 97 534)**	**228 (202 to 255)**	**-15.7 (-19.7 to -13.0)**	**160 940 (153 058 to 169 819)**	**472 (450 to 496)**	**-15.4 (-16.4 to -14.3)**	**23 627 (16 934 to 29 924)**	**69 (50 to 86)**	**-15.7 (-16.4 to -14.4)**
Botswana	2 681 (2 347 to 3 038)	241 (211 to 272)	17.5 (13.8 to 21.0)	4 798 (4 510 to 5 086)	500 (473 to 528)	17.3 (14.5 to 20.4)	704 (513 to 875)	72 (54 to 91)	15.5 (14.2 to 17.6)
Lesotho	2 236 (1 972 to 2 526)	212 (187 to 239)	24.5 (20.4 to 28.4)	3 286 (3 103 to 3 493)	371 (353 to 391)	9.0 (6.2 to 11.9)	480 (360 to 609)	53 (39 to 69)	5.9 (5.3 to 6.4)
Namibia	2 551 (2 249 to 2 875)	198 (175 to 223)	1.4 (-1.8 to 4.7)	4 357 (4 117 to 4 609)	420 (399 to 442)	5.4 (3.3 to 7.8)	646 (465 to 837)	61 (43 to 79)	4.3 (3.7 to 4.6)
South Africa	63 472 (56 203 to 71 205)	241 (213 to 270)	-19.8 (-24.5 to -16.7)	125 735 (119 481 to 132 854)	500 (476 to 526)	-19.4 (-20.5 to -18.2)	18 396 (13 338 to 23 226)	73 (54 to 91)	-19.5 (-20.1 to -18.5)
Swaziland	1 608 (1 409 to 1 819)	243 (212 to 273)	14.5 (10.3 to 18.7)	2 418 (2 276 to 2 561)	475 (451 to 501)	10.2 (7.5 to 12.8)	354 (255 to 456)	68 (50 to 85)	6.4 (5.6 to 7.9)
Zimbabwe	14 641 (12 895 to 16 415)	201 (178 to 225)	0.4 (-2.5 to 3.2)	20 347 (19 188 to 21 667)	356 (339 to 374)	-0.7 (-3.0 to 2.0)	3 001 (2 122 to 3 841)	51 (36 to 67)	-1.5 (-2.0 to -0.6)
**Western sub-Saharan Africa**	**383 905 (338 309 to 433 702)**	**211 (186 to 239)**	**-5.1 (-7.6 to -3.0)**	**545 995 (515 522 to 578 090)**	**399 (379 to 419)**	**1.2 (-0.1 to 2.4)**	**81 664 (60 365 to 105 310)**	**58 (43 to 73)**	**2.4 (1.4 to 3.1)**
Benin	11 351 (9 986 to 12 777)	221 (194 to 248)	2.5 (-0.9 to 6.0)	16 393 (15 385 to 17 422)	418 (396 to 440)	5.3 (2.3 to 8.9)	2 443 (1 797 to 3 076)	61 (44 to 78)	6.2 (5.4 to 6.7)
Burkina Faso	19 714 (17 274 to 22 294)	233 (204 to 264)	-1.8 (-5.0 to 1.4)	26 654 (25 117 to 28 245)	424 (402 to 446)	4.1 (0.8 to 7.2)	3 983 (2 842 to 5 091)	62 (47 to 79)	5.3 (1.6 to 7.9)
Cameroon	24 232 (21 301 to 27 422)	221 (194 to 251)	-2.0 (-5.3 to 1.8)	33 641 (31 667 to 35 774)	399 (379 to 421)	-2.9 (-5.8 to -0.2)	5 031 (3 659 to 6 474)	58 (42 to 73)	-2.1 (-2.4 to -1.9)
Cape Verde	532 (471 to 600)	193 (172 to 218)	3.0 (-0.2 to 6.1)	995 (938 to 1 057)	428 (405 to 451)	9.6 (6.0 to 13.4)	148 (108 to 190)	63 (46 to 79)	9.9 (8.7 to 10.8)
Chad	14 353 (12 615 to 16 238)	211 (186 to 240)	-7.2 (-23.9 to 2.2)	18 675 (17 463 to 20 133)	396 (374 to 422)	5.4 (1.2 to 9.2)	2 799 (2 010 to 3 622)	57 (41 to 72)	6.3 (2.7 to 8.8)
Cote d'Ivoire	23 088 (20 233 to 26 130)	228 (199 to 259)	-4.3 (-7.3 to -1.1)	32 366 (30 429 to 34 358)	411 (391 to 432)	-2.5 (-5.2 to 0.1)	4 804 (3 473 to 6 106)	60 (44 to 75)	-2.4 (-5.0 to -0.8)
The Gambia	1 953 (1 711 to 2 207)	208 (183 to 236)	-11.9 (-14.4 to -9.3)	2 673 (2 512 to 2 851)	396 (375 to 419)	-7.8 (-10.4 to -5.1)	400 (286 to 514)	58 (42 to 72)	-6.9 (-7.7 to -5.7)
Ghana	28 393 (25 083 to 31 916)	223 (196 to 252)	6.1 (3.1 to 9.6)	43 898 (41 584 to 46 337)	419 (399 to 439)	12.0 (8.7 to 15.5)	6 523 (4 689 to 8 278)	61 (46 to 78)	12.0 (9.8 to 13.5)
Guinea	11 776 (10 356 to 13 286)	201 (177 to 227)	-8.2 (-11.1 to -5.2)	16 565 (15 591 to 17 577)	361 (342 to 380)	-5.9 (-9.3 to -2.6)	2 485 (1 820 to 3 126)	53 (39 to 67)	-4.9 (-7.4 to -3.2)
Guinea-Bissau	1 964 (1 736 to 2 212)	224 (198 to 253)	-11.1 (-13.3 to -8.7)	2 765 (2 606 to 2 923)	396 (376 to 416)	-6.4 (-9.3 to -3.6)	412 (292 to 533)	58 (42 to 74)	-6.3 (-7.2 to -5.2)
Liberia	3 915 (3 428 to 4 442)	187 (164 to 212)	-57.8 (-77.1 to -33.3)	6 151 (5 637 to 6 912)	372 (342 to 416)	-5.3 (-12.5 to 2.5)	897 (640 to 1 139)	53 (40 to 68)	0.4 (-0.2 to 1.3)
Mali	15 985 (14 005 to 18 080)	189 (166 to 213)	-7.2 (-11.3 to -3.8)	21 345 (20 036 to 22 876)	357 (338 to 379)	5.6 (1.9 to 10.4)	3 213 (2 240 to 4 079)	52 (39 to 65)	6.6 (5.3 to 7.4)
Mauritania	3 770 (3 304 to 4 261)	203 (178 to 229)	-12.9 (-24.6 to -5.7)	6 151 (5 815 to 6 517)	401 (381 to 422)	3.1 (0.2 to 6.3)	918 (679 to 1 183)	59 (42 to 74)	4.2 (0.7 to 6.4)
Niger	17 392 (15 259 to 19 714)	182 (160 to 207)	-11.8 (-15.0 to -9.0)	21 992 (20 632 to 23 368)	335 (316 to 353)	-6.1 (-9.2 to -3.1)	3 323 (2 343 to 4 291)	49 (35 to 63)	-5.0 (-5.7 to -4.3)
Nigeria	176 697 (154 717 to 201 573)	210 (185 to 238)	-5.0 (-8.2 to -1.7)	253 591 (238 154 to 270 102)	406 (385 to 429)	0.6 (-2.0 to 3.4)	37 961 (26 522 to 49 297)	59 (42 to 76)	2.5 (2.0 to 2.9)
Sao Tome and Principe	187 (165 to 212)	190 (168 to 214)	5.9 (3.4 to 8.5)	275 (257 to 292)	385 (364 to 406)	7.6 (4.5 to 11.0)	41 (29 to 53)	57 (41 to 73)	7.4 (4.6 to 9.3)

Location	Incidence (95% UI)			Prevalence (95% UI)			YLDs (95% UI)		
	2016 counts	2016 age-standardised rates per 100,000	Percentage change in age-standardised rates between 1990 and 2016	2016 counts	2016 age-standardised rates per 100,000	Percentage change in age-standardised rates between 1990 and 2016	2016 counts	2016 age-standardised rates per 100,000	Percentage change in age-standardised rates between 1990 and 2016
Senegal	15 322<br>(13 426 to 17 290)	214<br>(189 to 241)	0.5<br>(-2.6 to 3.8)	22 373<br>(21 076 to 23 726)	405<br>(384 to 426)	3.6<br>(0.6 to 6.7)	3 359<br>(2 458 to 4 333)	59<br>(43 to 76)	4.2<br>(1.6 to 5.8)
Sierra Leone	6 401<br>(5 645 to 7 215)	218<br>(191 to 247)	12.4<br>(9.1 to 15.8)	9 367<br>(8 767 to 10 127)	406<br>(383 to 436)	15.4<br>(10.2 to 22.7)	1 394<br>(1 031 to 1 774)	59<br>(44 to 74)	13.0<br>(11.8 to 14.9)
Togo	6 876<br>(6 011 to 7 776)	201<br>(176 to 229)	-7.2<br>(-10.1 to -4.2)	10 116<br>(9 437 to 10 754)	375<br>(355 to 395)	-4.5<br>(-7.2 to -1.9)	1 511<br>(1 079 to 1 953)	55<br>(39 to 70)	-4.1<br>(-4.3 to -3.9)
**Eastern sub-Saharan Africa**	**404 336<br>(356 100 to 455 925)**	**230<br>(202 to 261)**	**-18.9<br>(-34.9 to -9.3)**	**583 283<br>(547 890 to 623 347)**	**437<br>(413 to 466)**	**3.6<br>(1.0 to 5.8)**	**86 575<br>(65 173 to 108 909)**	**63<br>(47 to 80)**	**4.0<br>(3.1 to 5.2)**
Burundi	13 198<br>(11 591 to 15 007)	254<br>(223 to 290)	1.4<br>(-1.3 to 4.3)	18 271<br>(16 969 to 19 913)	470<br>(442 to 506)	18.4<br>(13.1 to 27.2)	2 752<br>(1 905 to 3 615)	69<br>(50 to 87)	17.4<br>(15.1 to 18.7)
Comoros	800<br>(700 to 908)	230<br>(202 to 260)	-17.7<br>(-20.1 to -14.9)	1 273<br>(1 196 to 1 350)	447<br>(424 to 470)	-11.7<br>(-14.7 to -8.6)	191<br>(133 to 247)	66<br>(48 to 85)	-10.7<br>(-12.6 to -9.6)
Djibouti	1 073<br>(942 to 1 217)	240<br>(211 to 275)	-7.8<br>(-15.6 to -2.5)	1 710<br>(1 603 to 1 807)	470<br>(443 to 494)	-1.8<br>(-4.6 to 1.5)	253<br>(185 to 321)	69<br>(51 to 87)	-1.9<br>(-2.9 to -0.3)
Eritrea	5 465<br>(4 798 to 6 202)	234<br>(206 to 265)	-1.0<br>(-4.4 to 2.6)	8 182<br>(7 574 to 8 888)	440<br>(413 to 473)	14.1<br>(9.1 to 21.0)	1 225<br>(895 to 1 580)	65<br>(48 to 82)	14.6<br>(13.7 to 15.8)
Ethiopia	105 653<br>(92 974 to 119 075)	233<br>(205 to 265)	-39.2<br>(-61.6 to -18.8)	157 331<br>(147 158 to 169 564)	439<br>(414 to 470)	4.5<br>(-2.1 to 8.9)	23 259<br>(17 396 to 29 403)	64<br>(48 to 79)	7.3<br>(5.8 to 8.7)
Kenya	49 297<br>(43 416 to 55 776)	230<br>(202 to 260)	-0.3<br>(-1.5 to 0.9)	75 123<br>(71 180 to 79 297)	452<br>(431 to 472)	6.1<br>(5.0 to 7.2)	11 184<br>(8 107 to 14 215)	66<br>(48 to 85)	5.8<br>(5.1 to 6.3)
Madagascar	24 854<br>(21 859 to 28 374)	216<br>(190 to 245)	-7.3<br>(-10.3 to -4.0)	35 759<br>(33 479 to 37 763)	401<br>(379 to 420)	-3.6<br>(-6.7 to -0.4)	5 350<br>(3 942 to 6 740)	59<br>(45 to 75)	-2.8<br>(-3.3 to -1.9)
Malawi	16 165<br>(14 168 to 18 446)	194<br>(170 to 223)	-18.9<br>(-22.1 to -15.7)	21 164<br>(19 798 to 22 575)	347<br>(328 to 367)	-13.8<br>(-17.1 to -10.1)	3 154<br>(2 248 to 3 990)	51<br>(37 to 65)	-13.3<br>(-14.5 to -12.6)
Mozambique	29 474<br>(25 947 to 33 339)	221<br>(194 to 252)	-1.8<br>(-15.9 to 5.9)	42 159<br>(39 248 to 45 782)	423<br>(394 to 466)	3.4<br>(-5.3 to 9.5)	6 171<br>(4 476 to 7 821)	60<br>(45 to 76)	2.7<br>(-0.3 to 5.3)
Rwanda	12 228<br>(10 755 to 13 895)	214<br>(188 to 243)	-33.2<br>(-49.1 to -23.3)	26 124<br>(20 546 to 37 577)	581<br>(456 to 851)	31.1<br>(3.0 to 91.0)	3 859<br>(2 591 to 5 251)	84<br>(54 to 115)	21.6<br>(3.8 to 34.3)
Somalia	13 415<br>(10 881 to 18 011)	276<br>(230 to 350)	-10.3<br>(-16.5 to -5.4)	16 116<br>(14 818 to 18 020)	433<br>(401 to 477)	1.2<br>(-2.5 to 6.1)	2 366<br>(1 706 to 2 992)	63<br>(46 to 79)	-2.3<br>(-4.4 to -0.5)
South Sudan	15 669<br>(13 743 to 17 981)	254<br>(222 to 292)	-26.4<br>(-50.2 to -7.3)	21 178<br>(19 600 to 23 393)	468<br>(436 to 513)	0.3<br>(-4.0 to 6.1)	3 151<br>(2 430 to 3 908)	68<br>(53 to 83)	1.8<br>(-0.2 to 4.9)
Tanzania	57 485<br>(50 306 to 65 423)	230<br>(202 to 261)	-6.2<br>(-9.4 to -3.1)	79 827<br>(75 139 to 84 279)	426<br>(405 to 447)	-1.4<br>(-4.6 to 2.1)	11 831<br>(8 279 to 15 537)	62<br>(45 to 78)	-1.4<br>(-2.3 to -0.5)
Uganda	40 733<br>(35 416 to 46 378)	218<br>(190 to 249)	-7.8<br>(-12.1 to -4.2)	54 163<br>(50 264 to 58 760)	421<br>(393 to 461)	3.1<br>(-3.7 to 8.3)	8 108<br>(6 104 to 9 940)	62<br>(47 to 75)	5.2<br>(2.5 to 9.9)
Zambia	18 564<br>(16 296 to 21 060)	253<br>(222 to 289)	-5.1<br>(-8.2 to -1.9)	24 502<br>(22 961 to 26 144)	447<br>(423 to 470)	-1.4<br>(-4.2 to 1.4)	3 648<br>(2 663 to 4 739)	65<br>(47 to 82)	-1.5<br>(-2.0 to -0.7)
**Central sub-Saharan Africa**	**127 475<br>(111 979 to 143 529)**	**221<br>(193 to 249)**	**-6.0<br>(-11.2 to -3.1)**	**177 236<br>(166 675 to 189 412)**	**427<br>(405 to 451)**	**1.6<br>(-0.6 to 3.9)**	**26 393<br>(19 908 to 33 437)**	**62<br>(47 to 77)**	**2.5<br>(2.1 to 3.0)**
Angola	32 973<br>(28 979 to 37 132)	271<br>(237 to 306)	-10.7<br>(-26.6 to -1.8)	46 326<br>(43 376 to 49 821)	548<br>(515 to 593)	5.1<br>(0.8 to 8.7)	6 957<br>(4 967 to 8 850)	80<br>(60 to 102)	5.9<br>(4.5 to 6.9)
Central African Republic	5 068<br>(4 428 to 5 735)	206<br>(181 to 233)	-0.1<br>(-4.2 to 5.3)	7 009<br>(6 558 to 7 473)	349<br>(330 to 368)	-1.1<br>(-4.8 to 2.9)	1 034<br>(775 to 1 285)	50<br>(38 to 64)	-0.4<br>(-1.1 to 0.4)
Congo	5 848<br>(5 148 to 6 592)	262<br>(231 to 295)	-5.2<br>(-8.1 to -2.2)	8 763<br>(8 177 to 9 407)	503<br>(472 to 537)	5.0<br>(0.7 to 11.4)	1 293<br>(925 to 1 647)	73<br>(53 to 90)	5.3<br>(4.6 to 5.7)
DR Congo	80 091<br>(70 243 to 90 554)	201<br>(177 to 227)	-4.5<br>(-6.9 to -1.9)	109 392<br>(102 514 to 117 503)	386<br>(365 to 409)	-0.2<br>(-3.2 to 3.0)	16 252<br>(12 150 to 20 539)	56<br>(42 to 70)	0.7<br>(0.0 to 1.5)
Equatorial Guinea	1 076<br>(941 to 1 218)	286<br>(250 to 326)	16.6<br>(12.5 to 21.4)	1 765<br>(1 669 to 1 857)	567<br>(539 to 594)	37.4<br>(33.3 to 41.9)	263<br>(191 to 337)	83<br>(60 to 104)	37.1<br>(35.2 to 38.8)
Gabon	2 420<br>(2 123 to 2 725)	291<br>(256 to 328)	-5.2<br>(-8.0 to -2.0)	3 981<br>(3 770 to 4 185)	581<br>(553 to 608)	-0.6<br>(-3.3 to 1.8)	590<br>(419 to 749)	85<br>(61 to 108)	0.1<br>(-0.3 to 0.7)

Appendix Table 3: Incidence, prevalence, and YLDs for 1990 by location for TBI

Location	Incidence (95% UI)		Prevalence (95% UI)		YLDs (95% UI)	
	1990 counts	1990 age-standardised rates per 100,000	1990 counts	1990 age-standardised rates per 100,000	1990 counts	1990 age-standardised rates per 100,000
**Global**	**18 399 714 (16 583 249 to 20 420 559)**	**356 (320 to 396)**	**31 490 083 (30 266 457 to 32 779 825)**	**701 (675 to 727)**	**4 668 695 (3 465 238 to 5 967 006)**	**103 (76 to 129)**
**High SDI**	**3 370 080 (2 910 569 to 3 917 335)**	**379 (326 to 442)**	**6 790 828 (6 491 410 to 7 095 002)**	**702 (671 to 734)**	**992 030 (752 473 to 1 239 895)**	**103 (78 to 130)**
**High-middle SDI**	**4 597 643 (4 128 730 to 5 146 495)**	**524 (470 to 586)**	**9 077 193 (8 707 385 to 9 473 210)**	**1 092 (1 047 to 1 138)**	**1 366 263 (1 001 366 to 1 758 542)**	**163 (120 to 203)**
**Middle SDI**	**4 638 664 (4 238 433 to 5 088 866)**	**261 (238 to 287)**	**7 853 859 (7 542 639 to 8 204 480)**	**528 (508 to 550)**	**1 229 364 (916 051 to 1 554 962)**	**81 (60 to 103)**
**Low-middle SDI**	**4 494 130 (4 051 503 to 4 996 645)**	**358 (322 to 399)**	**6 301 797 (6 044 648 to 6 584 124)**	**630 (605 to 655)**	**976 700 (706 410 to 1 232 458)**	**95 (72 to 117)**
**Low SDI**	**1 403 939 (1 229 304 to 1 666 541)**	**404 (354 to 469)**	**1 664 187 (1 557 475 to 1 809 304)**	**648 (613 to 695)**	**253 457 (191 779 to 312 428)**	**96 (73 to 119)**
**High-income**	**2 954 460 (2 510 292 to 3 482 957)**	**330 (279 to 390)**	**5 924 156 (5 648 378 to 6 206 551)**	**606 (577 to 635)**	**845 042 (646 144 to 1 037 989)**	**87 (65 to 109)**
**High-income North America**	**940 485 (801 350 to 1 111 711)**	**343 (292 to 408)**	**1 880 065 (1 791 320 to 1 975 678)**	**640 (610 to 673)**	**271 496 (208 103 to 340 427)**	**93 (72 to 115)**
Canada	91 993 (78 048 to 109 057)	337 (286 to 401)	184 586 (175 887 to 194 043)	629 (599 to 661)	26 121 (20 154 to 32 296)	89 (69 to 111)
Greenland	192 (161 to 231)	396 (330 to 479)	264 (252 to 279)	614 (587 to 649)	37 (29 to 46)	84 (65 to 102)
United States	847 875 (722 607 to 1 005 600)	344 (293 to 408)	1 694 427 (1 613 935 to 1 779 880)	642 (611 to 674)	245 217 (185 223 to 302 001)	93 (69 to 115)
**Australasia**	**65 014 (54 706 to 76 780)**	**317 (267 to 374)**	**130 812 (123 797 to 138 521)**	**615 (582 to 650)**	**18 639 (14 145 to 23 408)**	**88 (66 to 108)**
Australia	53 527 (45 112 to 63 095)	313 (263 to 369)	108 238 (102 446 to 114 474)	607 (575 to 642)	15 419 (11 666 to 19 099)	87 (67 to 106)
New Zealand	11 486 (9 714 to 13 608)	336 (284 to 399)	22 574 (21 361 to 24 002)	652 (618 to 693)	3 216 (2 426 to 4 054)	93 (70 to 114)
**High-income Asia-Pacific**	**558 145 (474 065 to 658 277)**	**332 (282 to 393)**	**1 032 289 (982 783 to 1 084 151)**	**574 (546 to 603)**	**147 592 (111 581 to 182 893)**	**82 (62 to 103)**
Brunei	1 150 (982 to 1 363)	485 (415 to 571)	1 579 (1 480 to 1 684)	855 (809 to 903)	228 (171 to 286)	120 (89 to 149)
Japan	385 620 (327 368 to 455 297)	312 (264 to 368)	770 387 (732 849 to 809 879)	553 (525 to 581)	109 511 (83 720 to 137 592)	79 (61 to 97)
Singapore	7 974 (6 776 to 9 375)	298 (254 to 351)	13 506 (12 747 to 14 284)	521 (494 to 548)	1 933 (1 467 to 2 365)	74 (56 to 91)
South Korea	163 401 (138 180 to 193 092)	392 (334 to 462)	246 817 (234 570 to 258 551)	655 (627 to 686)	35 817 (26 707 to 43 974)	94 (69 to 116)
**Western Europe**	**1 293 625 (1 085 990 to 1 550 861)**	**338 (284 to 403)**	**2 726 849 (2 593 526 to 2 864 030)**	**626 (594 to 658)**	**385 435 (285 159 to 479 324)**	**89 (67 to 111)**
Andorra	143 (119 to 174)	288 (242 to 346)	306 (290 to 321)	532 (505 to 560)	43 (32 to 53)	75 (55 to 94)
Austria	31 179 (25 937 to 37 963)	402 (336 to 484)	63 212 (59 961 to 66 587)	716 (678 to 755)	8 851 (6 793 to 10 946)	101 (77 to 127)
Belgium	36 731 (30 719 to 43 729)	369 (310 to 439)	79 453 (75 418 to 83 629)	690 (655 to 726)	11 250 (8 720 to 13 823)	98 (75 to 123)
Cyprus	2 419 (2 054 to 2 866)	359 (305 to 426)	4 499 (4 270 to 4 738)	679 (644 to 715)	645 (490 to 794)	97 (73 to 122)
Denmark	18 835 (15 514 to 22 986)	354 (295 to 428)	38 277 (36 344 to 40 126)	636 (603 to 668)	5 348 (4 086 to 6 661)	90 (68 to 111)
Finland	18 191 (15 237 to 21 870)	364 (305 to 437)	36 100 (34 421 to 37 713)	637 (607 to 666)	5 112 (3 878 to 6 296)	91 (69 to 113)
France	222 732 (184 685 to 269 267)	383 (320 to 459)	447 605 (423 858 to 470 669)	703 (667 to 739)	62 880 (47 809 to 77 298)	100 (76 to 123)
Germany	258 498 (215 423 to 312 328)	330 (275 to 396)	572 826 (541 394 to 604 990)	612 (578 to 646)	81 002 (62 072 to 100 002)	87 (67 to 107)
Greece	35 852 (30 073 to 42 728)	355 (297 to 422)	79 188 (75 265 to 83 191)	681 (646 to 717)	11 300 (8 756 to 13 903)	98 (74 to 119)
Iceland	786 (661 to 933)	304 (256 to 361)	1 453 (1 380 to 1 524)	572 (544 to 600)	205 (152 to 255)	81 (60 to 102)
Ireland	11 299 (9 494 to 13 506)	316 (265 to 377)	20 431 (19 397 to 21 535)	589 (559 to 621)	2 902 (2 223 to 3 544)	84 (64 to 103)
Israel	13 408 (11 244 to 15 927)	291 (246 to 345)	21 836 (20 661 to 23 289)	535 (507 to 569)	3 121 (2 344 to 3 891)	76 (57 to 95)
Italy	205 495 (171 653 to 245 797)	357 (299 to 426)	442 234 (421 269 to 465 246)	663 (630 to 699)	62 845 (46 114 to 77 475)	95 (70 to 119)
Luxembourg	1 559 (1 309 to 1 866)	413 (346 to 492)	3 340 (3 165 to 3 506)	759 (719 to 798)	469 (357 to 579)	107 (80 to 134)
Malta	1 115 (932 to 1 334)	317 (266 to 379)	2 090 (1 990 to 2 187)	571 (544 to 598)	294 (224 to 375)	80 (61 to 100)
Netherlands	41 057 (34 254 to 49 194)	275 (230 to 329)	85 817 (81 403 to 90 529)	517 (491 to 546)	12 096 (9 059 to 15 006)	73 (55 to 88)
Norway	13 722 (11 424 to 16 542)	315 (264 to 377)	27 622 (26 292 to 28 987)	572 (544 to 601)	3 864 (2 915 to 4 724)	81 (63 to 98)
Portugal	37 940 (31 658 to 45 143)	378 (316 to 448)	78 010 (74 155 to 82 063)	704 (668 to 742)	11 039 (8 620 to 13 831)	100 (76 to 124)
Spain	133 239 (112 725 to 157 977)	339 (287 to 403)	279 699 (265 490 to 294 072)	650 (616 to 684)	40 102 (30 589 to 49 731)	94 (69 to 118)
Sweden	25 118 (20 969 to 30 201)	295 (247 to 352)	55 091 (52 601 to 57 571)	545 (518 to 570)	7 691 (5 994 to 9 382)	77 (59 to 94)
Switzerland	27 447 (22 703 to 33 496)	401 (334 to 483)	55 623 (52 799 to 58 394)	707 (670 to 743)	7 809 (5 889 to 9 795)	100 (76 to 125)
United Kingdom	155 598 (130 587 to 187 247)	277 (233 to 331)	329 461 (313 673 to 344 348)	511 (485 to 534)	46 130 (34 949 to 57 870)	72 (54 to 89)
**Southern Latin America**	**97 192 (81 736 to 115 986)**	**202 (169 to 241)**	**154 141 (148 296 to 160 530)**	**341 (328 to 355)**	**21 803 (16 859 to 26 844)**	**48 (37 to 60)**
Argentina	64 261 (54 249 to 76 806)	200 (168 to 239)	103 908 (99 937 to 108 064)	340 (327 to 353)	14 778 (11 423 to 18 298)	48 (37 to 59)
Chile	26 287 (22 028 to 31 711)	205 (171 to 246)	38 632 (37 054 to 40 294)	340 (327 to 355)	5 382 (4 043 to 6 763)	47 (36 to 59)
Uruguay	6 640 (5 570 to 8 051)	215 (180 to 260)	11 594 (11 088 to 12 109)	362 (347 to 379)	1 643 (1 242 to 2 008)	51 (40 to 63)
**Central Europe, Eastern Europe, and Central Asia**	**3 193 193 (2 849 250 to 3 635 952)**	**773 (689 to 881)**	**6 567 482 (6 248 531 to 6 903 450)**	**1 548 (1 474 to 1 627)**	**957 474 (713 921 to 1 212 007)**	**226 (165 to 285)**

Location	Incidence (95% UI)		Prevalence (95% UI)		YLDs (95% UI)	
	1990 counts	1990 age-standardised rates per 100,000	1990 counts	1990 age-standardised rates per 100,000	1990 counts	1990 age-standardised rates per 100,000
**Eastern Europe**	**1 756 643<br>(1 579 829 to 1 983 378)**	**791<br>(710 to 894)**	**3 751 866<br>(3 573 632 to 3 945 214)**	**1 570<br>(1 495 to 1 652)**	**545 589<br>(400 618 to 697 406)**	**229<br>(169 to 292)**
Belarus	75 014<br>(66 599 to 85 672)	737<br>(655 to 840)	162 937<br>(154 897 to 171 428)	1 496<br>(1 423 to 1 576)	23 907<br>(17 207 to 29 907)	220<br>(160 to 281)
Estonia	14 378<br>(12 814 to 16 371)	904<br>(808 to 1 032)	30 589<br>(29 132 to 32 057)	1 781<br>(1 698 to 1 868)	4 412<br>(3 270 to 5 519)	258<br>(189 to 328)
Latvia	26 018<br>(23 114 to 30 150)	960<br>(853 to 1 100)	54 840<br>(52 202 to 57 583)	1 863<br>(1 773 to 1 958)	7 858<br>(5 795 to 9 805)	268<br>(196 to 342)
Lithuania	33 561<br>(29 706 to 38 329)	895<br>(793 to 1 023)	70 081<br>(66 655 to 73 416)	1 778<br>(1 691 to 1 865)	10 089<br>(7 515 to 12 787)	257<br>(186 to 323)
Moldova	32 151<br>(28 675 to 36 782)	739<br>(659 to 844)	62 378<br>(59 059 to 65 505)	1 458<br>(1 382 to 1 530)	8 954<br>(6 471 to 11 369)	209<br>(151 to 267)
Russia	1 200 342<br>(1 075 978 to 1 345 055)	811<br>(727 to 910)	2 525 109<br>(2 406 747 to 2 650 120)	1 598<br>(1 521 to 1 677)	367 434<br>(273 283 to 458 411)	233<br>(173 to 295)
Ukraine	375 178<br>(333 330 to 430 478)	731<br>(650 to 835)	845 932<br>(801 708 to 892 616)	1 479<br>(1 400 to 1 562)	122 915<br>(90 703 to 156 203)	216<br>(154 to 270)
**Central Europe**	**1 093 189<br>(959 883 to 1 260 914)**	**883<br>(776 to 1 021)**	**2 230 915<br>(2 117 674 to 2 352 040)**	**1 721<br>(1 635 to 1 813)**	**326 068<br>(240 850 to 411 735)**	**252<br>(190 to 321)**
Albania	19 789<br>(17 437 to 22 567)	601<br>(529 to 683)	36 324<br>(34 098 to 38 473)	1 324<br>(1 249 to 1 398)	5 440<br>(3 942 to 6 944)	196<br>(145 to 250)
Bosnia and Herzegovina	21 967<br>(19 086 to 25 572)	483<br>(421 to 563)	46 946<br>(43 750 to 50 332)	1 039<br>(970 to 1 113)	7 088<br>(5 257 to 8 984)	156<br>(117 to 194)
Bulgaria	72 573<br>(63 876 to 83 532)	820<br>(723 to 941)	169 505<br>(161 022 to 178 943)	1 694<br>(1 605 to 1 791)	24 639<br>(18 187 to 31 490)	248<br>(180 to 315)
Croatia	40 367<br>(35 599 to 46 105)	827<br>(731 to 940)	87 364<br>(83 044 to 91 855)	1 626<br>(1 544 to 1 712)	12 723<br>(9 178 to 16 102)	239<br>(175 to 306)
Czech Republic	115 526<br>(99 741 to 135 942)	1 079<br>(935 to 1 264)	226 133<br>(213 256 to 239 413)	1 992<br>(1 877 to 2 107)	32 843<br>(23 267 to 41 368)	291<br>(209 to 367)
Hungary	119 145<br>(101 351 to 139 813)	1 068<br>(915 to 1 249)	223 231<br>(211 578 to 236 031)	1 873<br>(1 773 to 1 980)	32 108<br>(23 730 to 40 876)	271<br>(197 to 344)
Macedonia	12 172<br>(10 748 to 13 871)	615<br>(542 to 701)	25 105<br>(23 670 to 26 547)	1 290<br>(1 218 to 1 362)	3 732<br>(2 672 to 4 738)	191<br>(135 to 239)
Montenegro	4 445<br>(3 944 to 5 046)	723<br>(641 to 821)	9 326<br>(8 837 to 9 839)	1 535<br>(1 455 to 1 618)	1 377<br>(998 to 1 760)	226<br>(165 to 291)
Poland	336 567<br>(294 412 to 387 371)	885<br>(776 to 1 020)	666 337<br>(631 702 to 702 077)	1 701<br>(1 613 to 1 791)	98 048<br>(69 308 to 125 675)	250<br>(184 to 321)
Romania	214 369<br>(188 611 to 249 479)	906<br>(795 to 1 055)	456 957<br>(430 461 to 483 422)	1 844<br>(1 737 to 1 951)	66 436<br>(47 125 to 85 902)	269<br>(191 to 342)
Serbia	60 998<br>(53 674 to 69 537)	646<br>(569 to 736)	136 366<br>(129 457 to 143 980)	1 361<br>(1 291 to 1 439)	20 292<br>(14 652 to 26 403)	203<br>(148 to 258)
Slovakia	51 906<br>(45 310 to 60 316)	986<br>(861 to 1 142)	99 523<br>(94 119 to 104 846)	1 867<br>(1 767 to 1 968)	14 532<br>(10 813 to 18 507)	272<br>(199 to 348)
Slovenia	23 365<br>(20 413 to 27 074)	1 135<br>(990 to 1 314)	47 800<br>(45 412 to 50 515)	2 184<br>(2 073 to 2 309)	6 891<br>(5 089 to 8 681)	315<br>(226 to 398)
**Central Asia**	**343 361<br>(305 973 to 390 945)**	**495<br>(442 to 562)**	**584 701<br>(551 866 to 618 259)**	**1 034<br>(981 to 1 088)**	**86 254<br>(63 281 to 109 439)**	**151<br>(108 to 190)**
Armenia	19 355<br>(17 074 to 22 167)	541<br>(478 to 623)	44 440<br>(38 642 to 52 921)	1 351<br>(1 184 to 1 589)	6 492<br>(4 744 to 8 274)	196<br>(145 to 249)
Azerbaijan	34 974<br>(31 105 to 39 706)	481<br>(429 to 547)	59 509<br>(56 317 to 63 117)	992<br>(942 to 1 047)	8 861<br>(6 369 to 11 388)	146<br>(106 to 182)
Georgia	28 423<br>(25 145 to 32 386)	520<br>(461 to 594)	60 904<br>(57 354 to 64 323)	1 079<br>(1 017 to 1 141)	8 913<br>(6 382 to 11 423)	158<br>(116 to 203)
Kazakhstan	92 963<br>(82 755 to 105 599)	555<br>(495 to 630)	166 832<br>(157 815 to 176 564)	1 124<br>(1 066 to 1 188)	24 417<br>(16 925 to 31 660)	163<br>(112 to 208)
Kyrgyzstan	23 952<br>(21 249 to 27 263)	542<br>(483 to 614)	38 637<br>(36 545 to 40 974)	1 095<br>(1 038 to 1 156)	5 680<br>(4 035 to 7 269)	159<br>(116 to 203)
Mongolia	9 534<br>(8 451 to 10 935)	419<br>(372 to 476)	13 632<br>(12 816 to 14 528)	827<br>(782 to 874)	2 040<br>(1 426 to 2 614)	122<br>(87 to 158)
Tajikistan	24 727<br>(21 622 to 28 468)	482<br>(423 to 552)	36 199<br>(33 961 to 38 447)	987<br>(929 to 1 042)	5 422<br>(3 980 to 6 961)	145<br>(105 to 188)
Turkmenistan	16 729<br>(14 896 to 19 031)	449<br>(402 to 510)	25 052<br>(23 507 to 26 620)	913<br>(864 to 962)	3 755<br>(2 692 to 4 835)	135<br>(98 to 174)
Uzbekistan	92 703<br>(81 995 to 106 454)	441<br>(393 to 504)	139 496<br>(131 545 to 148 085)	914<br>(866 to 963)	20 775<br>(14 693 to 27 249)	134<br>(95 to 170)
**Latin America and Caribbean**	**1 248 345<br>(1 120 136 to 1 401 389)**	**327<br>(294 to 366)**	**2 006 681<br>(1 908 744 to 2 105 818)**	**657<br>(630 to 686)**	**301 374<br>(215 718 to 379 700)**	**97<br>(70 to 123)**
**Central Latin America**	**506 693<br>(455 895 to 569 212)**	**324<br>(291 to 363)**	**776 432<br>(741 852 to 812 099)**	**641<br>(616 to 667)**	**117 101<br>(82 832 to 150 037)**	**95<br>(68 to 120)**
Colombia	104 158<br>(93 412 to 116 953)	322<br>(289 to 361)	165 467<br>(156 884 to 174 231)	643<br>(614 to 671)	24 965<br>(17 878 to 31 786)	95<br>(69 to 119)
Costa Rica	7 589<br>(6 702 to 8 618)	267<br>(236 to 303)	12 989<br>(12 239 to 13 651)	542<br>(515 to 567)	1 991<br>(1 402 to 2 572)	82<br>(60 to 105)
El Salvador	15 744<br>(13 912 to 18 026)	314<br>(279 to 356)	24 789<br>(22 661 to 28 165)	626<br>(580 to 700)	3 721<br>(2 659 to 4 704)	93<br>(68 to 116)
Guatemala	22 082<br>(19 514 to 25 106)	264<br>(235 to 299)	30 265<br>(28 428 to 32 597)	498<br>(472 to 530)	4 561<br>(3 211 to 5 843)	74<br>(54 to 93)
Honduras	10 337<br>(9 165 to 11 729)	214<br>(192 to 241)	14 055<br>(13 226 to 14 875)	426<br>(405 to 446)	2 166<br>(1 595 to 2 729)	64<br>(48 to 82)
Mexico	274 147<br>(246 621 to 307 817)	347<br>(312 to 393)	412 303<br>(393 037 to 431 591)	680<br>(651 to 708)	61 949<br>(43 521 to 79 800)	100<br>(73 to 128)
Nicaragua	9 889<br>(8 752 to 11 127)	251<br>(225 to 281)	15 719<br>(14 213 to 18 148)	564<br>(521 to 635)	2 333<br>(1 744 to 2 936)	82<br>(61 to 106)
Panama	6 145<br>(5 460 to 6 906)	264<br>(236 to 297)	10 356<br>(9 783 to 10 909)	551<br>(523 to 577)	1 580<br>(1 104 to 2 047)	83<br>(58 to 106)
Venezuela	56 603<br>(50 311 to 63 879)	306<br>(273 to 343)	90 489<br>(85 708 to 95 027)	621<br>(592 to 648)	13 841<br>(10 177 to 17 825)	93<br>(66 to 118)
**Andean Latin America**	**105 218<br>(94 555 to 117 097)**	**281<br>(253 to 312)**	**165 109<br>(156 490 to 173 400)**	**571<br>(545 to 596)**	**24 796<br>(17 959 to 31 063)**	**84<br>(62 to 107)**
Bolivia	20 265<br>(18 169 to 22 672)	306<br>(274 to 341)	29 985<br>(28 403 to 31 556)	593<br>(564 to 621)	4 474<br>(3 150 to 5 740)	87<br>(63 to 111)
Ecuador	28 846<br>(25 867 to 32 117)	301<br>(270 to 337)	46 481<br>(43 871 to 49 193)	622<br>(592 to 652)	7 018<br>(4 978 to 9 080)	92<br>(68 to 118)
Peru	56 107<br>(50 132 to 62 531)	264<br>(237 to 293)	88 642<br>(84 024 to 93 523)	541<br>(516 to 565)	13 305<br>(9 525 to 16 911)	80<br>(59 to 102)
**Caribbean**	**92 397<br>(82 979 to 103 210)**	**265<br>(238 to 296)**	**163 127<br>(155 100 to 170 308)**	**543<br>(518 to 566)**	**24 535<br>(17 520 to 31 977)**	**81<br>(59 to 104)**

Location	Incidence (95% UI)		Prevalence (95% UI)		YLDs (95% UI)	
	1990 counts	1990 age-standardised rates per 100,000	1990 counts	1990 age-standardised rates per 100,000	1990 counts	1990 age-standardised rates per 100,000
Antigua and Barbuda	154 (138 to 171)	252 (227 to 281)	296 (281 to 311)	540 (514 to 566)	45 (32 to 57)	81 (59 to 104)
The Bahamas	735 (658 to 821)	295 (264 to 330)	1 246 (1 183 to 1 308)	594 (565 to 619)	188 (139 to 245)	88 (63 to 112)
Barbados	593 (532 to 662)	231 (207 to 258)	1 226 (1 163 to 1 288)	487 (462 to 510)	184 (134 to 234)	73 (52 to 93)
Belize	470 (421 to 524)	242 (218 to 268)	654 (619 to 687)	500 (477 to 522)	101 (75 to 129)	76 (55 to 95)
Bermuda	179 (160 to 202)	335 (300 to 376)	324 (308 to 339)	685 (652 to 715)	48 (35 to 62)	102 (73 to 130)
Cuba	31 236 (27 878 to 35 203)	300 (267 to 339)	62 379 (59 178 to 65 334)	618 (586 to 646)	9 305 (6 491 to 11 718)	92 (66 to 118)
Dominica	147 (130 to 165)	214 (191 to 241)	259 (244 to 273)	450 (426 to 472)	39 (28 to 50)	68 (48 to 87)
Dominican Republic	16 574 (14 769 to 18 488)	234 (210 to 259)	27 106 (25 549 to 28 501)	505 (480 to 528)	4 154 (2 894 to 5 348)	76 (53 to 98)
Grenada	237 (212 to 266)	244 (219 to 276)	383 (363 to 403)	490 (466 to 514)	57 (40 to 75)	73 (54 to 93)
Guyana	1 872 (1 687 to 2 101)	266 (240 to 300)	2 783 (2 635 to 2 928)	495 (472 to 518)	422 (297 to 543)	74 (54 to 94)
Haiti	17 311 (15 580 to 19 327)	241 (216 to 269)	23 606 (22 412 to 24 775)	441 (421 to 461)	3 538 (2 513 to 4 543)	65 (47 to 82)
Jamaica	4 809 (4 267 to 5 391)	193 (172 to 217)	8 259 (7 761 to 8 697)	420 (396 to 440)	1 257 (869 to 1 630)	63 (45 to 83)
Puerto Rico	9 997 (8 981 to 11 203)	284 (255 to 318)	20 158 (19 132 to 21 089)	592 (562 to 619)	3 032 (2 162 to 3 947)	89 (65 to 113)
Saint Lucia	345 (309 to 387)	242 (219 to 271)	573 (544 to 601)	505 (481 to 528)	87 (62 to 112)	76 (53 to 98)
Saint Vincent and the Grenadines	267 (237 to 302)	242 (217 to 273)	412 (389 to 433)	488 (463 to 511)	62 (43 to 81)	73 (52 to 94)
Suriname	1 001 (900 to 1 110)	249 (224 to 276)	1 691 (1 597 to 1 772)	513 (487 to 536)	255 (191 to 325)	76 (57 to 95)
Trinidad and Tobago	3 050 (2 733 to 3 419)	250 (225 to 279)	5 174 (4 907 to 5 454)	497 (474 to 522)	774 (556 to 1 016)	74 (54 to 94)
Virgin Islands, U.S.	274 (246 to 307)	268 (242 to 301)	527 (501 to 553)	550 (523 to 575)	80 (58 to 100)	82 (59 to 106)
**Tropical Latin America**	**544 037 (485 710 to 614 427)**	**359 (323 to 405)**	**902 013 (854 964 to 954 879)**	**723 (690 to 760)**	**134 926 (95 506 to 174 211)**	**106 (77 to 135)**
Brazil	533 854 (477 049 to 602 942)	363 (326 to 409)	885 980 (840 100 to 938 000)	729 (695 to 766)	132 490 (92 569 to 171 965)	107 (77 to 137)
Paraguay	10 183 (8 877 to 11 704)	241 (213 to 276)	16 033 (14 989 to 17 070)	518 (487 to 547)	2 443 (1 741 to 3 127)	78 (54 to 100)
**Southeast Asia, East Asia, and Oceania**	**3 815 907 (3 492 618 to 4 177 443)**	**230 (210 to 252)**	**7 137 884 (6 839 021 to 7 481 130)**	**499 (480 to 521)**	**1 094 425 (742 038 to 1 386 885)**	**76 (55 to 97)**
**East Asia**	**2 784 904 (2 543 453 to 3 042 719)**	**234 (214 to 255)**	**5 451 632 (5 229 171 to 5 708 299)**	**515 (496 to 538)**	**835 421 (599 853 to 1 062 302)**	**78 (56 to 101)**
China	2 699 344 (2 465 330 to 2 949 298)	235 (215 to 257)	5 279 703 (5 064 287 to 5 527 243)	517 (498 to 540)	808 950 (569 039 to 1 051 127)	78 (57 to 100)
North Korea	38 242 (34 727 to 42 056)	171 (155 to 188)	71 196 (68 077 to 74 695)	383 (368 to 401)	11 024 (7 945 to 14 082)	59 (43 to 73)
Taiwan (Province of China)	47 318 (42 848 to 52 405)	234 (213 to 259)	100 733 (96 462 to 105 626)	540 (519 to 565)	15 481 (10 831 to 19 897)	82 (58 to 107)
**Southeast Asia**	**1 016 510 (919 885 to 1 123 532)**	**223 (202 to 246)**	**1 666 381 (1 583 764 to 1 764 511)**	**454 (434 to 477)**	**256 179 (190 341 to 323 116)**	**69 (51 to 87)**
Cambodia	17 781 (15 516 to 21 214)	210 (183 to 247)	32 640 (25 985 to 45 891)	494 (401 to 679)	4 816 (3 245 to 6 423)	72 (51 to 96)
Indonesia	375 929 (341 627 to 414 343)	211 (191 to 232)	641 194 (607 615 to 678 609)	438 (418 to 460)	98 320 (70 559 to 124 111)	66 (48 to 85)
Laos	8 901 (7 550 to 11 409)	205 (174 to 264)	10 668 (10 128 to 11 295)	340 (326 to 356)	1 621 (1 154 to 2 133)	51 (36 to 64)
Malaysia	43 385 (39 349 to 47 710)	237 (216 to 260)	74 171 (70 644 to 77 905)	522 (500 to 545)	11 461 (8 138 to 14 508)	79 (59 to 103)
Maldives	389 (354 to 427)	196 (180 to 216)	638 (605 to 673)	419 (401 to 438)	96 (74 to 120)	62 (47 to 79)
Mauritius	2 038 (1 854 to 2 247)	187 (171 to 205)	3 869 (3 679 to 4 068)	407 (388 to 425)	592 (427 to 757)	62 (45 to 78)
Myanmar	71 262 (64 576 to 78 874)	175 (158 to 193)	116 705 (108 642 to 127 373)	347 (326 to 375)	17 670 (12 550 to 22 812)	52 (38 to 66)
Philippines	136 049 (120 281 to 155 719)	212 (188 to 243)	191 437 (179 886 to 206 949)	414 (392 to 441)	29 677 (21 354 to 37 700)	63 (47 to 79)
Sri Lanka	71 330 (52 669 to 109 934)	403 (300 to 614)	85 806 (80 264 to 93 197)	577 (544 to 619)	13 237 (9 661 to 16 874)	88 (66 to 113)
Seychelles	157 (142 to 173)	227 (206 to 250)	272 (258 to 285)	475 (454 to 495)	42 (32 to 54)	73 (53 to 94)
Thailand	157 156 (142 452 to 174 416)	275 (249 to 305)	287 138 (274 339 to 300 431)	599 (573 to 624)	44 419 (31 190 to 56 952)	92 (67 to 115)
Timor-Leste	2 478 (1 640 to 4 381)	319 (215 to 555)	2 921 (2 241 to 4 121)	510 (404 to 700)	445 (293 to 596)	76 (52 to 99)
Vietnam	128 291 (115 503 to 142 626)	193 (174 to 214)	216 551 (206 659 to 228 442)	405 (387 to 424)	33 399 (24 413 to 42 693)	62 (47 to 78)
**Oceania**	**14 494 (13 149 to 16 006)**	**218 (199 to 241)**	**19 871 (18 902 to 20 928)**	**404 (388 to 423)**	**3 049 (2 189 to 3 882)**	**61 (43 to 76)**
American Samoa	125 (114 to 138)	257 (234 to 281)	196 (187 to 206)	547 (523 to 571)	30 (22 to 38)	81 (59 to 103)
Federated States of Micronesia	209 (189 to 230)	209 (190 to 230)	285 (271 to 301)	414 (396 to 433)	43 (31 to 55)	62 (45 to 80)
Fiji	1 366 (1 243 to 1 500)	183 (167 to 201)	2 175 (2 073 to 2 292)	375 (359 to 392)	333 (248 to 425)	56 (40 to 70)
Guam	333 (303 to 367)	243 (222 to 266)	605 (577 to 635)	539 (515 to 564)	93 (66 to 120)	82 (60 to 104)
Kiribati	136 (122 to 151)	171 (154 to 189)	186 (176 to 197)	324 (310 to 340)	29 (21 to 36)	49 (35 to 62)
Marshall Islands	93 (84 to 102)	200 (182 to 218)	134 (127 to 141)	403 (384 to 423)	20 (14 to 27)	60 (44 to 75)
Northern Mariana Islands	140 (127 to 154)	278 (254 to 305)	236 (224 to 249)	628 (600 to 657)	36 (27 to 47)	94 (66 to 123)

Location	Incidence (95% UI)		Prevalence (95% UI)		YLDs (95% UI)	
	1990 counts	1990 age-standardised rates per 100,000	1990 counts	1990 age-standardised rates per 100,000	1990 counts	1990 age-standardised rates per 100,000
Papua New Guinea	9 443 (8 534 to 10 481)	227 (205 to 252)	12 177 (11 577 to 12 829)	400 (383 to 419)	1 871 (1 349 to 2 342)	60 (44 to 77)
Samoa	355 (323 to 390)	205 (187 to 225)	522 (495 to 549)	427 (409 to 447)	81 (56 to 105)	65 (47 to 83)
Solomon Islands	621 (561 to 686)	202 (184 to 223)	859 (811 to 910)	389 (370 to 408)	132 (98 to 166)	58 (43 to 74)
Tonga	222 (201 to 246)	226 (206 to 252)	325 (309 to 340)	450 (431 to 469)	49 (34 to 64)	67 (49 to 85)
Vanuatu	290 (262 to 320)	185 (168 to 202)	404 (383 to 428)	367 (352 to 385)	62 (46 to 79)	55 (41 to 70)
**North Africa and Middle East**	**1 270 208 (1 124 861 to 1 441 125)**	**359 (320 to 404)**	**1 979 982 (1 814 408 to 2 235 170)**	**772 (717 to 853)**	**292 044 (218 161 to 361 347)**	**112 (84 to 141)**
**North Africa and Middle East**	**1 270 208 (1 124 861 to 1 441 125)**	**359 (320 to 404)**	**1 979 982 (1 814 408 to 2 235 170)**	**772 (717 to 853)**	**292 044 (218 161 to 361 347)**	**112 (84 to 141)**
Afghanistan	45 606 (38 341 to 57 282)	346 (294 to 427)	100 742 (56 274 to 185 458)	1 065 (629 to 1 870)	14 132 (7 820 to 20 135)	148 (86 to 211)
Algeria	88 620 (79 414 to 98 732)	335 (303 to 370)	131 921 (125 550 to 139 236)	722 (692 to 755)	19 637 (14 186 to 24 760)	105 (79 to 132)
Bahrain	1 942 (1 750 to 2 153)	370 (335 to 409)	3 350 (3 167 to 3 535)	813 (776 to 851)	505 (364 to 647)	120 (88 to 152)
Egypt	136 872 (122 976 to 151 981)	226 (203 to 250)	221 936 (210 563 to 233 209)	488 (465 to 511)	33 353 (23 913 to 43 234)	72 (53 to 90)
Iran	328 777 (244 170 to 454 239)	553 (415 to 746)	409 170 (363 611 to 481 537)	999 (905 to 1 150)	58 805 (42 715 to 73 250)	143 (107 to 177)
Iraq	69 276 (61 696 to 78 119)	378 (340 to 425)	155 762 (113 668 to 241 511)	1 184 (896 to 1 741)	23 450 (15 585 to 32 479)	173 (113 to 234)
Jordan	11 702 (10 512 to 13 002)	328 (299 to 362)	16 679 (15 846 to 17 631)	729 (697 to 762)	2 468 (1 700 to 3 200)	105 (77 to 136)
Kuwait	11 977 (8 790 to 17 393)	554 (416 to 796)	15 363 (14 506 to 16 337)	964 (920 to 1 014)	2 292 (1 726 to 2 940)	141 (104 to 178)
Lebanon	17 311 (12 084 to 27 703)	593 (419 to 943)	34 412 (23 025 to 58 044)	1 406 (961 to 2 309)	4 708 (2 765 to 6 744)	193 (107 to 270)
Libya	14 475 (12 976 to 16 094)	325 (294 to 360)	24 404 (22 941 to 26 033)	762 (723 to 805)	3 651 (2 675 to 4 730)	112 (83 to 143)
Morocco	75 106 (67 337 to 84 160)	283 (255 to 315)	118 450 (112 402 to 124 972)	611 (584 to 640)	17 731 (12 994 to 22 329)	90 (65 to 114)
Palestine	6 954 (5 882 to 8 712)	292 (248 to 361)	10 744 (8 559 to 14 695)	723 (590 to 961)	1 583 (1 078 to 2 074)	105 (73 to 138)
Oman	8 329 (7 446 to 9 278)	452 (404 to 503)	12 597 (11 958 to 13 264)	1 002 (953 to 1 051)	1 887 (1 291 to 2 427)	147 (104 to 188)
Qatar	2 503 (2 252 to 2 771)	495 (447 to 546)	4 727 (4 474 to 4 987)	1 154 (1 102 to 1 208)	710 (503 to 908)	169 (121 to 218)
Saudi Arabia	71 599 (64 384 to 79 245)	439 (394 to 486)	106 495 (101 635 to 111 937)	962 (924 to 1 003)	15 917 (11 781 to 20 096)	140 (105 to 175)
Sudan	58 654 (51 919 to 66 960)	287 (254 to 325)	81 780 (76 255 to 88 542)	566 (534 to 602)	12 131 (8 971 to 15 533)	82 (61 to 103)
Syria	33 345 (29 745 to 37 077)	252 (227 to 278)	48 364 (45 027 to 52 519)	572 (539 to 613)	7 265 (5 131 to 9 372)	84 (60 to 106)
Tunisia	25 186 (22 457 to 28 053)	300 (270 to 332)	43 174 (41 002 to 45 395)	653 (623 to 682)	6 454 (4 794 to 8 297)	96 (71 to 122)
Turkey	212 727 (191 552 to 239 448)	374 (338 to 418)	370 698 (351 685 to 391 752)	816 (778 to 857)	55 056 (40 816 to 68 969)	120 (89 to 149)
United Arab Emirates	9 736 (8 713 to 10 807)	498 (446 to 552)	16 869 (15 977 to 17 798)	1 119 (1 072 to 1 170)	2 530 (1 807 to 3 276)	164 (119 to 209)
Yemen	38 677 (34 675 to 43 333)	314 (283 to 348)	51 007 (48 173 to 54 310)	657 (626 to 691)	7 494 (5 656 to 9 623)	94 (72 to 117)
**South Asia**	**4 206 093 (3 782 692 to 4 683 722)**	**420 (377 to 470)**	**5 802 124 (5 546 292 to 6 057 023)**	**709 (682 to 738)**	**877 400 (637 343 to 1 117 709)**	**105 (78 to 132)**
**South Asia**	**4 206 093 (3 782 692 to 4 683 722)**	**420 (377 to 470)**	**5 802 124 (5 546 292 to 6 057 023)**	**709 (682 to 738)**	**877 400 (637 343 to 1 117 709)**	**105 (78 to 132)**
Bangladesh	297 592 (267 711 to 331 939)	304 (273 to 340)	404 305 (385 291 to 424 435)	539 (516 to 562)	70 381 (50 240 to 90 513)	87 (63 to 109)
Bhutan	2 004 (1 795 to 2 233)	417 (373 to 469)	2 759 (2 630 to 2 889)	754 (721 to 786)	415 (308 to 529)	111 (83 to 139)
India	3 494 934 (3 143 205 to 3 891 100)	444 (399 to 497)	4 825 966 (4 615 230 to 5 040 754)	738 (709 to 768)	720 628 (528 822 to 905 989)	108 (81 to 134)
Nepal	62 617 (56 058 to 69 805)	368 (328 to 413)	82 429 (78 655 to 86 469)	624 (597 to 652)	12 385 (9 441 to 15 780)	92 (67 to 117)
Pakistan	348 947 (314 631 to 387 678)	335 (301 to 373)	486 665 (464 634 to 508 068)	635 (608 to 661)	73 667 (53 233 to 95 167)	94 (69 to 119)
**Sub-Saharan Africa**	**1 711 506 (1 511 752 to 1 986 189)**	**369 (327 to 422)**	**2 071 773 (1 969 879 to 2 180 916)**	**617 (591 to 644)**	**307 044 (226 289 to 386 756)**	**89 (66 to 114)**
**Southern sub-Saharan Africa**	**200 387 (180 600 to 223 049)**	**388 (353 to 430)**	**300 124 (286 441 to 315 816)**	**755 (724 to 791)**	**44 762 (31 451 to 57 249)**	**111 (80 to 140)**
Botswana	4 047 (3 627 to 4 522)	300 (269 to 333)	5 605 (5 313 to 5 926)	582 (555 to 609)	846 (596 to 1 073)	85 (63 to 109)
Lesotho	4 169 (3 745 to 4 618)	267 (242 to 294)	5 644 (5 358 to 5 938)	485 (463 to 507)	851 (640 to 1 075)	72 (53 to 93)
Namibia	4 141 (3 718 to 4 562)	296 (268 to 327)	5 678 (5 382 to 5 983)	566 (540 to 592)	859 (635 to 1 094)	84 (60 to 106)
South Africa	160 556 (145 344 to 179 479)	433 (394 to 482)	247 558 (236 168 to 260 490)	843 (807 to 883)	36 882 (26 309 to 47 026)	124 (91 to 156)
Swaziland	2 539 (2 289 to 2 811)	312 (281 to 344)	3 371 (3 194 to 3 566)	596 (568 to 625)	508 (365 to 656)	88 (64 to 110)
Zimbabwe	24 935 (22 346 to 27 648)	263 (237 to 291)	32 268 (30 738 to 34 045)	468 (449 to 489)	4 824 (3 342 to 6 243)	68 (47 to 89)
**Western sub-Saharan Africa**	**601 131 (540 080 to 671 059)**	**324 (291 to 361)**	**784 818 (747 359 to 823 263)**	**580 (555 to 603)**	**116 494 (86 017 to 150 417)**	**84 (62 to 105)**
Benin	14 861 (13 323 to 16 545)	313 (281 to 348)	19 413 (18 407 to 20 440)	569 (543 to 593)	2 889 (2 137 to 3 630)	83 (61 to 105)
Burkina Faso	26 567 (23 676 to 29 696)	314 (281 to 352)	31 753 (30 152 to 33 476)	533 (509 to 557)	4 714 (3 455 to 5 934)	77 (60 to 97)
Cameroon	34 852 (31 306 to 38 957)	311 (278 to 347)	46 964 (44 502 to 49 387)	569 (542 to 593)	6 990 (5 043 to 9 012)	83 (60 to 104)
Cape Verde	925 (829 to 1 023)	271 (245 to 301)	1 285 (1 224 to 1 346)	540 (517 to 562)	195 (141 to 252)	81 (59 to 101)

Location	Incidence (95% UI)		Prevalence (95% UI)		YLDs (95% UI)	
	1990 counts	1990 age-standardised rates per 100,000	1990 counts	1990 age-standardised rates per 100,000	1990 counts	1990 age-standardised rates per 100,000
Chad	19 136 (16 362 to 23 363)	340 (291 to 414)	22 738 (21 161 to 24 863)	566 (533 to 610)	3 359 (2 482 to 4 284)	82 (60 to 102)
Cote d'Ivoire	39 596 (35 571 to 44 260)	364 (328 to 407)	52 928 (50 421 to 55 582)	636 (608 to 665)	7 887 (5 738 to 9 992)	92 (69 to 114)
The Gambia	2 698 (2 420 to 3 023)	322 (288 to 362)	3 502 (3 323 to 3 702)	609 (582 to 638)	523 (376 to 672)	89 (65 to 111)
Ghana	39 495 (35 691 to 43 797)	292 (264 to 326)	52 862 (50 305 to 55 616)	530 (507 to 553)	7 930 (5 711 to 10 053)	78 (59 to 98)
Guinea	17 769 (16 028 to 19 907)	313 (281 to 349)	24 347 (23 069 to 25 616)	566 (539 to 591)	3 613 (2 757 to 4 442)	82 (63 to 102)
Guinea-Bissau	3 435 (3 090 to 3 830)	356 (319 to 396)	4 453 (4 240 to 4 656)	595 (570 to 619)	665 (477 to 857)	87 (64 to 111)
Liberia	15 372 (9 781 to 26 921)	702 (453 to 1 218)	8 816 (8 006 to 10 320)	584 (545 to 647)	1 190 (873 to 1 487)	79 (60 to 100)
Mali	25 993 (23 381 to 28 980)	307 (276 to 343)	30 980 (29 501 to 32 580)	522 (499 to 545)	4 589 (3 247 to 5 790)	76 (57 to 94)
Mauritania	6 688 (5 727 to 7 943)	362 (313 to 420)	8 314 (7 892 to 8 757)	608 (581 to 634)	1 240 (918 to 1 599)	89 (64 to 112)
Niger	24 177 (21 763 to 26 983)	316 (284 to 352)	28 915 (27 364 to 30 431)	551 (525 to 575)	4 304 (3 111 to 5 486)	80 (58 to 102)
Nigeria	284 916 (254 743 to 319 128)	321 (287 to 359)	388 507 (368 967 to 408 395)	597 (570 to 623)	57 498 (40 238 to 74 507)	86 (61 to 110)
Sao Tome and Principe	379 (340 to 424)	340 (305 to 381)	515 (490 to 542)	663 (633 to 693)	77 (55 to 99)	98 (73 to 125)
Senegal	22 078 (19 779 to 24 696)	315 (284 to 352)	28 780 (27 331 to 30 291)	572 (547 to 597)	4 313 (3 202 to 5 501)	84 (62 to 106)
Sierra Leone	11 484 (10 343 to 12 895)	315 (283 to 354)	15 755 (14 989 to 16 572)	568 (543 to 593)	2 410 (1 798 to 3 049)	84 (63 to 106)
Togo	10 694 (9 585 to 11 949)	301 (271 to 337)	13 965 (13 213 to 14 759)	546 (521 to 571)	2 092 (1 487 to 2 714)	80 (56 to 103)
**Eastern sub-Saharan Africa**	**732 664 (618 871 to 925 265)**	**417 (355 to 513)**	**761 273 (712 818 to 822 834)**	**608 (576 to 649)**	**112 478 (83 034 to 143 062)**	**88 (65 to 112)**
Burundi	17 587 (15 701 to 19 739)	353 (316 to 398)	20 578 (19 430 to 21 688)	554 (527 to 579)	3 158 (2 301 to 4 038)	82 (61 to 102)
Comoros	1 573 (1 414 to 1 752)	435 (391 to 483)	2 116 (2 017 to 2 221)	795 (762 to 827)	315 (223 to 405)	116 (85 to 147)
Djibouti	2 242 (1 988 to 2 556)	400 (354 to 453)	2 807 (2 668 to 2 949)	717 (686 to 747)	427 (309 to 543)	106 (77 to 136)
Eritrea	9 956 (8 934 to 11 108)	362 (326 to 404)	11 822 (11 208 to 12 475)	589 (562 to 617)	1 797 (1 290 to 2 315)	87 (64 to 112)
Ethiopia	279 310 (203 574 to 427 361)	605 (447 to 897)	218 653 (199 554 to 246 571)	643 (599 to 709)	30 975 (22 968 to 39 364)	91 (68 to 114)
Kenya	67 811 (60 580 to 75 959)	321 (288 to 359)	84 964 (81 064 to 89 200)	592 (568 to 616)	12 863 (9 253 to 16 418)	87 (63 to 112)
Madagascar	35 051 (31 461 to 39 268)	323 (290 to 362)	45 127 (42 786 to 47 482)	582 (554 to 608)	6 772 (4 950 to 8 561)	85 (64 to 109)
Malawi	28 015 (25 131 to 31 548)	314 (282 to 354)	33 693 (31 827 to 35 585)	530 (504 to 554)	5 008 (3 487 to 6 405)	76 (56 to 98)
Mozambique	41 963 (36 319 to 49 550)	328 (286 to 385)	56 186 (48 382 to 69 628)	603 (533 to 730)	8 235 (5 908 to 10 424)	87 (65 to 109)
Rwanda	30 899 (24 990 to 41 480)	458 (379 to 597)	28 322 (26 667 to 30 356)	615 (586 to 647)	4 604 (3 397 to 5 879)	97 (72 to 120)
Somalia	26 500 (21 275 to 36 175)	455 (371 to 612)	28 039 (26 036 to 30 750)	637 (598 to 685)	4 382 (3 184 to 5 496)	96 (72 to 120)
South Sudan	30 777 (22 482 to 48 630)	539 (407 to 807)	28 490 (26 475 to 31 430)	715 (675 to 768)	4 171 (3 038 to 5 351)	102 (76 to 129)
Tanzania	82 315 (74 116 to 92 248)	343 (308 to 383)	102 462 (97 074 to 108 027)	605 (578 to 634)	15 360 (10 958 to 19 976)	89 (63 to 112)
Uganda	50 898 (45 437 to 57 188)	315 (282 to 356)	64 710 (56 062 to 80 213)	558 (494 to 676)	9 474 (6 816 to 11 868)	80 (58 to 99)
Zambia	27 384 (24 552 to 30 562)	355 (319 to 397)	32 915 (31 259 to 34 595)	619 (592 to 645)	4 919 (3 600 to 6 384)	90 (65 to 113)
**Central sub-Saharan Africa**	**177 324 (159 538 to 199 421)**	**357 (321 to 400)**	**225 559 (213 826 to 239 140)**	**621 (594 to 653)**	**33 282 (25 404 to 41 879)**	**90 (68 to 112)**
Angola	45 825 (39 008 to 57 286)	433 (373 to 529)	53 887 (48 824 to 62 650)	726 (668 to 827)	7 926 (5 855 to 9 868)	105 (80 to 132)
Central African Republic	7 891 (7 099 to 8 770)	283 (255 to 314)	10 169 (9 690 to 10 688)	460 (441 to 481)	1 500 (1 105 to 1 878)	67 (50 to 85)
Congo	8 128 (7 308 to 9 041)	365 (327 to 406)	10 535 (10 039 to 11 046)	617 (591 to 643)	1 561 (1 150 to 1 953)	89 (66 to 110)
DR Congo	110 513 (99 249 to 123 060)	338 (303 to 375)	143 702 (136 872 to 150 854)	601 (575 to 628)	21 227 (16 104 to 26 602)	87 (65 to 109)
Equatorial Guinea	1 233 (1 108 to 1 370)	346 (311 to 385)	1 623 (1 547 to 1 703)	574 (550 to 599)	243 (179 to 310)	84 (62 to 105)
Gabon	3 734 (3 368 to 4 135)	420 (379 to 466)	5 642 (5 408 to 5 886)	762 (731 to 793)	828 (597 to 1 044)	111 (80 to 140)

Appendix Table 4: Incidence, prevalence, and YLDs for 2016 and percentage change of rates by age group for TBI

Age group	Incidence (95% UI)			Prevalence (95% UI)			YLDs (95% UI)		
	2016 counts	2016 rates per 100,000	Percentage change in rates between 1990 and 2016	2016 counts	2016 rates per 100,000	Percentage change in rates between 1990 and 2016	2016 counts	2016 rates per 100,000	Percentage change in rates between 1990 and 2016
Early Neonatal	504<br>(325 to 848)	21<br>(13 to 35)	-0.2<br>(-19.1 to 28.8)	57<br>(39 to 96)	2<br>(2 to 4)	-4.0<br>(-20.4 to 22.1)	9<br>(5 to 13)	0<br>(0 to 1)	-37.1<br>(-49.9 to -19.5)
Late Neonatal	3 190<br>(2 396 to 4 639)	44<br>(33 to 64)	-16.4<br>(-27.1 to -1.0)	433<br>(344 to 603)	6<br>(5 to 8)	-18.3<br>(-26.3 to -6.1)	57<br>(39 to 74)	1<br>(1 to 1)	-19.9<br>(-20.8 to -19.5)
Post Neonatal	559 617<br>(487 981 to 646 357)	482<br>(420 to 556)	-20.1<br>(-22.7 to -17.7)	74 439<br>(62 667 to 86 444)	64<br>(54 to 74)	-18.4<br>(-20.5 to -16.3)	12 646<br>(8 759 to 16 761)	11<br>(8 to 14)	-17.3<br>(-17.8 to -16.5)
1 to 4	1 775 144<br>(1 483 493 to 2 124 381)	351<br>(293 to 420)	-3.8<br>(-7.0 to 0.9)	476 131<br>(421 179 to 535 410)	94<br>(83 to 106)	-5.8<br>(-7.6 to -3.6)	77 039<br>(53 071 to 102 448)	15<br>(10 to 20)	-5.9<br>(-7.4 to -5.1)
5 to 9	1 902 937<br>(1 576 232 to 2 314 135)	296<br>(245 to 360)	-2.5<br>(-6.5 to 2.2)	997 202<br>(898 751 to 1 107 122)	155<br>(140 to 172)	-5.4<br>(-7.8 to -3.7)	157 524<br>(109 203 to 205 129)	25<br>(17 to 32)	-5.1<br>(-6.7 to -4.3)
10 to 14	1 895 489<br>(1 545 690 to 2 303 137)	310<br>(253 to 377)	-1.1<br>(-5.6 to 3.5)	1 450 922<br>(1 337 173 to 1 582 737)	237<br>(219 to 259)	-4.0<br>(-6.3 to -2.3)	227 683<br>(160 251 to 293 288)	37<br>(26 to 48)	-4.4<br>(-5.2 to -3.8)
15 to 19	2 059 877<br>(1 710 375 to 2 458 485)	349<br>(290 to 416)	4.0<br>(-0.8 to 8.6)	1 980 663<br>(1 828 105 to 2 133 618)	335<br>(310 to 361)	0.5<br>(-1.1 to 2.0)	308 033<br>(220 104 to 399 136)	52<br>(37 to 68)	0.1<br>(-0.2 to 0.5)
20 to 24	2 285 070<br>(1 935 541 to 2 702 974)	380<br>(322 to 450)	7.0<br>(1.9 to 11.6)	2 751 479<br>(2 569 707 to 2 932 366)	458<br>(428 to 488)	4.1<br>(2.6 to 5.4)	426 932<br>(298 221 to 555 232)	71<br>(50 to 92)	4.6<br>(3.6 to 5.1)
25 to 29	2 255 537<br>(1 920 470 to 2 665 797)	368<br>(313 to 434)	8.4<br>(4.0 to 13.1)	3 627 690<br>(3 398 134 to 3 855 897)	591<br>(554 to 628)	5.5<br>(4.1 to 6.7)	559 426<br>(403 372 to 726 124)	91<br>(66 to 118)	6.7<br>(5.5 to 7.5)
30 to 34	2 027 150<br>(1 696 651 to 2 373 683)	361<br>(302 to 422)	5.9<br>(1.5 to 10.3)	4 052 081<br>(3 815 994 to 4 282 322)	721<br>(679 to 762)	4.4<br>(3.3 to 5.7)	618 067<br>(436 550 to 774 941)	110<br>(78 to 138)	5.0<br>(3.0 to 6.0)
35 to 39	1 809 932<br>(1 523 571 to 2 126 500)	359<br>(302 to 422)	7.0<br>(2.5 to 12.0)	4 317 570<br>(4 092 351 to 4 545 752)	857<br>(812 to 902)	5.7<br>(4.5 to 7.2)	654 190<br>(481 775 to 827 749)	130<br>(96 to 164)	5.9<br>(5.1 to 6.9)
40 to 44	1 751 295<br>(1 452 636 to 2 056 685)	360<br>(298 to 422)	8.0<br>(3.2 to 12.6)	4 834 715<br>(4 607 460 to 5 063 986)	993<br>(946 to 1 040)	6.7<br>(5.7 to 7.8)	726 100<br>(521 689 to 909 619)	149<br>(107 to 187)	6.5<br>(5.5 to 7.3)
45 to 49	1 680 460<br>(1 408 219 to 2 012 062)	364<br>(305 to 436)	9.3<br>(4.7 to 13.8)	5 263 082<br>(5 031 452 to 5 499 438)	1 141<br>(1 091 to 1 192)	10.0<br>(8.9 to 11.2)	784 684<br>(577 461 to 995 154)	170<br>(125 to 216)	11.0<br>(10.2 to 11.8)
50 to 54	1 555 576<br>(1 296 319 to 1 831 876)	378<br>(315 to 446)	5.0<br>(0.7 to 9.0)	5 369 924<br>(5 122 142 to 5 587 200)	1 306<br>(1 246 to 1 359)	7.3<br>(6.2 to 8.5)	791 140<br>(575 973 to 1 022 811)	192<br>(140 to 249)	8.0<br>(7.2 to 8.7)
55 to 59	1 333 448<br>(1 098 094 to 1 614 053)	385<br>(317 to 466)	9.1<br>(4.9 to 13.1)	5 075 079<br>(4 852 656 to 5 289 758)	1 465<br>(1 401 to 1 527)	11.4<br>(10.4 to 12.5)	736 851<br>(540 365 to 936 515)	213<br>(156 to 270)	12.1<br>(11.3 to 12.7)
60 to 64	1 163 777<br>(958 791 to 1 430 685)	388<br>(319 to 476)	5.6<br>(1.0 to 9.5)	4 770 518<br>(4 573 493 to 4 966 905)	1 589<br>(1 523 to 1 654)	10.6<br>(9.6 to 11.6)	680 592<br>(501 103 to 845 319)	227<br>(167 to 282)	11.2<br>(10.2 to 12.0)
65 to 69	881 920<br>(705 176 to 1 104 987)	391<br>(312 to 489)	6.4<br>(2.7 to 10.1)	3 707 278<br>(3 552 103 to 3 880 926)	1 642<br>(1 573 to 1 719)	14.1<br>(13.2 to 15.1)	515 990<br>(391 263 to 644 082)	229<br>(173 to 285)	12.9<br>(11.6 to 14.0)
70 to 74	662 009<br>(519 162 to 840 771)	421<br>(330 to 535)	8.0<br>(3.4 to 12.9)	2 600 034<br>(2 482 798 to 2 724 454)	1 653<br>(1 579 to 1 732)	19.4<br>(18.6 to 20.3)	356 831<br>(270 469 to 441 839)	227<br>(172 to 281)	19.5<br>(17.8 to 20.9)
75 to 79	566 537<br>(446 346 to 726 438)	486<br>(383 to 623)	2.5<br>(-3.5 to 8.3)	2 004 535<br>(1 897 536 to 2 130 695)	1 720<br>(1 628 to 1 828)	19.2<br>(18.2 to 20.3)	267 393<br>(200 523 to 328 245)	229<br>(172 to 282)	21.4<br>(19.0 to 23.3)
80 to 84	434 018<br>(341 672 to 557 234)	598<br>(471 to 768)	-2.1<br>(-7.2 to 4.3)	1 197 655<br>(1 126 107 to 1 270 976)	1 650<br>(1 552 to 1 751)	16.1<br>(14.9 to 17.3)	155 274<br>(120 809 to 190 880)	214<br>(166 to 263)	17.5<br>(14.5 to 20.6)
85 to 89	295 200<br>(229 058 to 385 231)	741<br>(575 to 967)	-2.7<br>(-9.2 to 3.7)	640 168<br>(595 172 to 687 875)	1 607<br>(1 494 to 1 727)	15.0<br>(13.6 to 16.4)	80 163<br>(62 764 to 98 155)	201<br>(158 to 246)	16.3<br>(12.5 to 20.4)
90 to 94	142 892<br>(109 995 to 190 729)	968<br>(745 to 1 292)	4.5<br>(-1.2 to 9.7)	243 498<br>(223 563 to 265 242)	1 649<br>(1 514 to 1 796)	11.9<br>(10.5 to 13.4)	29 401<br>(22 934 to 35 448)	199<br>(155 to 240)	12.8<br>(9.6 to 16.8)
95 plus	40 454<br>(29 609 to 55 838)	1 227<br>(898 to 1 693)	12.9<br>(3.9 to 20.8)	60 521<br>(54 744 to 66 867)	1 835<br>(1 660 to 2 028)	8.5<br>(6.6 to 10.0)	7 150<br>(5 388 to 8 670)	217<br>(163 to 263)	8.6<br>(5.9 to 11.7)

Appendix Table 5: Incidence, prevalence, and YLDs for 2016 and percentage change of age-standardized rates by location for SCI, males

Location	Incidence (95% UI)			Prevalence (95% UI)			YLDs (95% UI)		
	2016 counts	2016 age-standardised rates per 100,000	Percentage change in age-standardised rates between 1990 and 2016	2016 counts	2016 age-standardised rates per 100,000	Percentage change in age-standardised rates between 1990 and 2016	2016 counts	2016 age-standardised rates per 100,000	Percentage change in age-standardised rates between 1990 and 2016
**Global**	**539 719 (450 081 to 657 655)**	**15 (12 to 18)**	**-3.7 (-7.7 to 3.8)**	**15 168 507 (14 043 842 to 16 835 674)**	**412 (382 to 457)**	**0.8 (-1.1 to 3.4)**	**5 353 537 (3 675 209 to 6 939 493)**	**145 (100 to 188)**	**-9.1 (-12.2 to -6.2)**
**High SDI**	**142 099 (112 884 to 178 133)**	**28 (22 to 35)**	**-5.9 (-9.1 to -2.6)**	**4 928 845 (4 539 821 to 5 333 765)**	**848 (775 to 924)**	**-1.4 (-3.0 to 0.2)**	**1 481 397 (1 036 476 to 1 960 635)**	**257 (181 to 338)**	**-0.6 (-1.3 to 0.2)**
**High-middle SDI**	**92 210 (77 773 to 108 996)**	**16 (13 to 19)**	**-16.9 (-21.8 to -13.1)**	**3 038 403 (2 802 507 to 3 388 512)**	**473 (436 to 525)**	**-6.1 (-7.9 to -4.3)**	**970 894 (663 372 to 1 299 245)**	**151 (104 to 200)**	**-16.2 (-19.5 to -13.4)**
**Middle SDI**	**108 023 (91 160 to 127 998)**	**9 (8 to 11)**	**5.5 (-3.2 to 12.9)**	**3 272 340 (3 067 743 to 3 497 348)**	**267 (251 to 285)**	**28.2 (25.8 to 30.9)**	**1 074 398 (754 785 to 1 390 113)**	**88 (61 to 113)**	**6.7 (1.6 to 11.2)**
**Low-middle SDI**	**145 225 (114 699 to 202 654)**	**14 (11 to 19)**	**21.4 (6.7 to 56.3)**	**2 936 968 (2 637 559 to 3 591 076)**	**295 (265 to 358)**	**22.2 (18.9 to 27.2)**	**1 251 876 (882 037 to 1 670 085)**	**126 (87 to 168)**	**13.0 (8.7 to 17.0)**
**Low SDI**	**54 899 (41 046 to 83 075)**	**15 (11 to 21)**	**-21.1 (-34.6 to -10.7)**	**1 051 988 (794 327 to 1 619 170)**	**370 (269 to 599)**	**11.6 (6.0 to 22.0)**	**544 632 (315 606 to 811 605)**	**195 (105 to 298)**	**6.8 (2.1 to 10.2)**
**High-income**	**146 887 (116 359 to 184 613)**	**28 (22 to 35)**	**-6.8 (-10.1 to -3.5)**	**5 160 763 (4 754 348 to 5 587 164)**	**864 (791 to 942)**	**-2.1 (-3.7 to -0.5)**	**1 520 840 (1 060 447 to 1 985 944)**	**255 (178 to 336)**	**-1.4 (-2.2 to -0.6)**
**High-income North America**	**49 939 (39 715 to 62 053)**	**27 (22 to 34)**	**-2.3 (-7.1 to 2.9)**	**1 531 676 (1 407 926 to 1 641 222)**	**769 (705 to 827)**	**-6.6 (-9.7 to -3.3)**	**449 994 (311 844 to 588 296)**	**227 (160 to 300)**	**-6.0 (-7.2 to -4.6)**
Canada	4 880 (3 887 to 6 065)	27 (22 to 33)	-6.2 (-11.5 to -1.1)	175 367 (159 542 to 190 633)	836 (758 to 913)	-0.9 (-5.1 to 3.5)	51 875 (36 123 to 68 206)	249 (171 to 329)	-0.3 (-1.3 to 0.6)
Greenland	8 (7 to 11)	31 (25 to 39)	-20.4 (-23.5 to -17.5)	222 (203 to 240)	728 (668 to 790)	-4.1 (-9.0 to 1.0)	69 (47 to 89)	225 (157 to 297)	-12.6 (-17.4 to -7.9)
United States	45 033 (35 820 to 56 014)	27 (22 to 34)	-1.6 (-6.8 to 4.0)	1 355 533 (1 247 455 to 1 452 427)	761 (698 to 818)	-7.2 (-10.7 to -3.6)	398 419 (277 469 to 524 101)	224 (154 to 294)	-6.7 (-8.1 to -5.1)
**Australasia**	**3 547 (2 831 to 4 458)**	**25 (20 to 32)**	**-4.4 (-11.2 to 1.8)**	**132 833 (121 153 to 145 663)**	**842 (765 to 928)**	**2.8 (-1.9 to 8.1)**	**39 337 (27 560 to 51 753)**	**250 (175 to 331)**	**3.6 (1.9 to 5.2)**
Australia	2 978 (2 370 to 3 742)	25 (20 to 31)	-3.4 (-10.3 to 2.9)	111 379 (101 178 to 122 345)	834 (756 to 922)	3.5 (-1.6 to 9.3)	33 031 (22 905 to 43 390)	247 (172 to 325)	4.3 (2.7 to 5.8)
New Zealand	569 (455 to 714)	26 (21 to 33)	-8.6 (-15.4 to -2.1)	21 454 (19 692 to 23 586)	884 (807 to 976)	-0.3 (-5.0 to 5.5)	6 332 (4 402 to 8 433)	263 (183 to 346)	0.5 (-1.1 to 2.1)
**High-income Asia-Pacific**	**28 846 (22 792 to 36 461)**	**30 (24 to 38)**	**-13.9 (-18.6 to -9.1)**	**970 527 (888 569 to 1 058 135)**	**908 (824 to 1 002)**	**-2.5 (-5.5 to 1.1)**	**286 173 (201 656 to 374 529)**	**269 (186 to 355)**	**-3.0 (-5.4 to -0.7)**
Brunei	79 (63 to 98)	40 (31 to 50)	-12.6 (-17.1 to -8.0)	2 467 (2 232 to 2 760)	1 090 (991 to 1 217)	-7.1 (-11.2 to -3.0)	732 (495 to 951)	322 (225 to 424)	-15.3 (-18.5 to -12.1)
Japan	20 113 (15 890 to 25 389)	29 (23 to 37)	-10.1 (-16.1 to -4.8)	679 506 (623 003 to 740 120)	902 (817 to 999)	1.0 (-2.7 to 5.7)	200 348 (139 425 to 263 917)	267 (187 to 354)	1.3 (-1.2 to 3.7)
Singapore	571 (452 to 720)	31 (24 to 39)	-4.2 (-9.7 to 1.3)	22 183 (20 011 to 24 489)	1 005 (903 to 1 115)	10.7 (5.0 to 18.3)	6 583 (4 589 to 8 573)	299 (211 to 394)	6.9 (3.6 to 9.8)
South Korea	8 083 (6 371 to 10 112)	33 (26 to 42)	-19.1 (-23.8 to -13.5)	266 370 (242 280 to 292 129)	914 (828 to 1 010)	-9.6 (-14.6 to -4.7)	78 555 (54 728 to 103 345)	271 (189 to 356)	-19.1 (-22.4 to -16.2)
**Western Europe**	**57 903 (45 283 to 74 025)**	**29 (22 to 36)**	**-7.4 (-11.1 to -3.7)**	**2 319 699 (2 117 827 to 2 554 802)**	**963 (875 to 1 075)**	**1.3 (-0.9 to 3.8)**	**684 138 (475 517 to 904 203)**	**286 (199 to 374)**	**2.3 (1.1 to 3.4)**
Andorra	11 (9 to 14)	30 (23 to 38)	3.2 (-0.4 to 7.2)	451 (408 to 501)	997 (893 to 1 127)	6.3 (1.3 to 11.8)	133 (93 to 177)	295 (204 to 388)	6.8 (6.4 to 7.2)
Austria	1 371 (1 055 to 1 771)	33 (26 to 43)	-16.7 (-21.8 to -10.9)	54 192 (49 292 to 60 487)	1 084 (978 to 1 223)	-8.0 (-12.4 to -2.7)	15 982 (11 047 to 21 239)	321 (220 to 426)	-7.0 (-8.5 to -5.7)
Belgium	1 796 (1 396 to 2 320)	32 (25 to 41)	2.9 (-3.2 to 9.3)	64 280 (58 486 to 71 595)	1 005 (907 to 1 128)	6.2 (0.6 to 12.5)	18 953 (13 163 to 25 129)	297 (207 to 394)	7.0 (5.7 to 8.2)
Cyprus	132 (105 to 165)	31 (24 to 39)	-0.9 (-5.5 to 3.9)	4 953 (4 486 to 5 496)	1 014 (917 to 1 129)	6.9 (2.3 to 12.2)	1 461 (1 022 to 1 908)	300 (209 to 394)	1.7 (-0.8 to 3.9)
Denmark	794 (617 to 1 025)	29 (23 to 37)	-6.9 (-12.5 to -1.2)	31 563 (28 449 to 35 229)	981 (877 to 1 103)	8.3 (2.3 to 13.9)	9 298 (6 245 to 12 089)	291 (201 to 384)	9.3 (8.2 to 10.5)
Finland	991 (761 to 1 302)	36 (28 to 46)	-0.6 (-6.2 to 4.5)	34 791 (31 678 to 38 619)	1 101 (993 to 1 235)	14.5 (9.3 to 21.4)	10 195 (6 983 to 13 436)	326 (225 to 431)	16.0 (15.0 to 16.9)
France	9 611 (7 443 to 12 385)	30 (24 to 38)	-11.2 (-16.2 to -5.7)	337 985 (307 539 to 373 207)	960 (870 to 1 066)	-2.3 (-7.1 to 3.3)	99 326 (69 792 to 129 853)	284 (196 to 381)	-1.4 (-2.2 to -0.6)
Germany	10 739 (8 311 to 13 716)	28 (22 to 36)	-6.6 (-12.3 to -1.1)	447 863 (404 533 to 496 812)	942 (843 to 1 057)	3.9 (-1.7 to 10.6)	131 335 (91 768 to 174 674)	279 (190 to 372)	4.9 (3.4 to 6.3)
Greece	1 452 (1 138 to 1 848)	29 (23 to 37)	-5.8 (-10.5 to -0.7)	60 742 (55 271 to 66 896)	983 (889 to 1 092)	-0.8 (-5.5 to 3.6)	17 815 (12 266 to 23 609)	290 (205 to 390)	-0.5 (-1.0 to 0.1)
Iceland	47 (37 to 59)	29 (22 to 37)	-1.6 (-6.5 to 3.6)	1 775 (1 599 to 1 977)	983 (883 to 1 102)	7.6 (2.3 to 13.8)	525 (367 to 700)	292 (200 to 390)	8.3 (6.8 to 9.6)
Ireland	676 (527 to 865)	31 (24 to 39)	6.2 (-0.7 to 12.9)	26 628 (23 871 to 29 778)	1 083 (966 to 1 216)	14.8 (8.5 to 21.7)	7 875 (5 441 to 10 478)	321 (222 to 428)	15.7 (14.3 to 17.0)
Israel	1 082 (847 to 1 361)	26 (21 to 33)	1.4 (-9.4 to 8.2)	41 067 (35 093 to 52 747)	1 044 (891 to 1 342)	19.8 (9.2 to 33.4)	12 856 (8 709 to 17 989)	326 (213 to 452)	19.5 (8.1 to 29.7)
Italy	8 300 (6 540 to 10 482)	30 (23 to 38)	-6.7 (-11.3 to -1.7)	355 125 (321 388 to 395 883)	1 018 (916 to 1 148)	1.5 (-4.1 to 6.4)	104 853 (73 456 to 138 915)	303 (212 to 402)	2.6 (1.2 to 3.8)
Luxembourg	83 (65 to 107)	30 (23 to 38)	-23.4 (-28.2 to -18.4)	3 247 (2 950 to 3 587)	970 (877 to 1 080)	-14.1 (-18.5 to -9.3)	961 (660 to 1 271)	288 (200 to 385)	-13.1 (-14.4 to -11.8)
Malta	58 (45 to 74)	30 (23 to 38)	-7.8 (-12.4 to -3.1)	2 524 (2 293 to 2 793)	1 034 (933 to 1 157)	-0.6 (-6.2 to 5.6)	745 (517 to 990)	306 (212 to 403)	-0.5 (-2.1 to 0.2)
Netherlands	1 922 (1 511 to 2 397)	24 (19 to 30)	-1.4 (-8.1 to 5.7)	79 075 (73 098 to 86 038)	810 (745 to 885)	4.6 (-1.8 to 11.8)	23 460 (16 266 to 31 094)	241 (168 to 317)	5.6 (4.1 to 7.2)
Norway	764 (594 to 980)	30 (23 to 38)	-3.6 (-8.8 to 1.9)	29 376 (26 636 to 32 999)	985 (890 to 1 114)	8.3 (2.3 to 14.6)	8 685 (6 063 to 11 506)	293 (202 to 394)	9.3 (7.7 to 10.8)
Portugal	1 231 (977 to 1 542)	26 (20 to 32)	-29.1 (-34.2 to -23.9)	47 839 (43 777 to 52 437)	818 (743 to 905)	-24.0 (-29.0 to -19.3)	14 015 (9 750 to 18 529)	242 (167 to 323)	-28.1 (-29.8 to -26.5)
Spain	5 809 (4 584 to 7 354)	27 (21 to 34)	-10.4 (-16.0 to -4.2)	251 750 (229 800 to 277 845)	946 (856 to 1 056)	0.1 (-5.2 to 6.3)	74 298 (51 022 to 98 129)	281 (194 to 368)	1.3 (-0.1 to 2.5)
Sweden	1 457 (1 135 to 1 875)	31 (24 to 39)	4.9 (0.3 to 9.5)	59 123 (52 938 to 67 239)	1 066 (945 to 1 222)	12.3 (6.2 to 19.0)	17 433 (12 075 to 22 834)	316 (220 to 413)	13.2 (12.3 to 13.9)
Switzerland	1 215 (939 to 1 543)	29 (23 to 37)	-28.5 (-34.0 to -23.3)	45 535 (41 695 to 49 920)	922 (840 to 1 017)	-23.5 (-28.1 to -18.4)	13 476 (9 385 to 17 713)	273 (188 to 360)	-22.7 (-23.5 to -21.7)
United Kingdom	8 302 (6 502 to 10 600)	27 (21 to 35)	0.6 (-3.8 to 4.4)	337 424 (304 847 to 376 622)	945 (848 to 1 063)	5.0 (2.4 to 8.3)	99 246 (69 815 to 131 661)	279 (196 to 371)	5.5 (4.2 to 6.7)
**Southern Latin America**	**6 651 (5 335 to 8 175)**	**21 (17 to 26)**	**8.0 (3.1 to 12.1)**	**206 028 (188 691 to 222 331)**	**630 (577 to 680)**	**20.1 (15.9 to 25.1)**	**63 420 (43 935 to 83 498)**	**194 (136 to 251)**	**6.8 (2.9 to 10.3)**
Argentina	4 429 (3 563 to 5 422)	21 (17 to 26)	9.0 (1.8 to 14.7)	135 699 (124 171 to 147 556)	634 (581 to 689)	21.1 (15.1 to 27.2)	42 351 (29 999 to 54 974)	197 (138 to 257)	9.0 (5.0 to 12.4)
Chile	1 865 (1 490 to 2 304)	21 (17 to 26)	1.7 (-2.5 to 5.9)	59 684 (54 628 to 64 291)	624 (572 to 674)	15.8 (11.1 to 21.1)	17 691 (12 220 to 23 389)	185 (130 to 244)	1.2 (-3.0 to 4.9)
Uruguay	358 (283 to 444)	21 (17 to 26)	14.2 (9.7 to 19.1)	10 634 (9 715 to 11 553)	611 (557 to 666)	21.4 (15.3 to 29.3)	3 226 (2 243 to 4 179)	186 (129 to 240)	9.8 (6.0 to 12.9)
**Central Europe, Eastern Europe, and Central Asia**	**47 271 (38 847 to 56 848)**	**24 (19 to 28)**	**-6.7 (-9.3 to -4.0)**	**1 298 060 (1 207 617 to 1 400 010)**	**599 (557 to 646)**	**0.5 (-2.5 to 5.2)**	**392 515 (274 614 to 515 568)**	**182 (128 to 238)**	**-9.3 (-14.2 to -4.5)**
**Eastern Europe**	**25 472 (21 124 to 30 489)**	**25 (21 to 30)**	**-6.1 (-9.6 to -2.6)**	**633 782 (589 539 to 682 223)**	**577 (536 to 623)**	**-4.8 (-8.2 to -0.9)**	**185 806 (131 104 to 245 753)**	**170 (118 to 224)**	**-11.9 (-15.8 to -7.3)**
Belarus	1 286 (1 056 to 1 551)	28 (23 to 33)	12.7 (7.4 to 18.3)	32 698 (30 282 to 34 891)	649 (599 to 694)	11.5 (7.0 to 16.0)	9 459 (6 603 to 12 448)	188 (131 to 249)	2.7 (0.1 to 4.9)
Estonia	149 (123 to 181)	24 (19 to 28)	-20.0 (-24.4 to -15.1)	4 402 (4 074 to 4 729)	627 (577 to 677)	-5.1 (-9.7 to 0.3)	1 280 (893 to 1 683)	183 (128 to 240)	-9.4 (-11.5 to -7.6)
Latvia	226 (185 to 273)	24 (20 to 29)	-21.4 (-25.7 to -16.3)	6 049 (5 635 to 6 458)	586 (543 to 628)	-12.3 (-15.8 to -8.6)	1 751 (1 213 to 2 291)	171 (118 to 225)	-17.4 (-19.3 to -15.8)
Lithuania	389 (318 to 468)	28 (23 to 33)	-4.5 (-9.4 to 0.9)	9 934 (9 223 to 10 635)	651 (602 to 701)	0.5 (-4.3 to 5.1)	2 874 (1 981 to 3 783)	189 (132 to 248)	-3.9 (-5.6 to -2.4)
Moldova	400 (333 to 472)	20 (17 to 24)	-16.6 (-20.8 to -12.1)	11 235 (10 341 to 12 241)	515 (474 to 561)	-9.4 (-13.4 to -3.8)	3 520 (2 456 to 4 593)	161 (113 to 211)	-15.5 (-20.1 to -10.4)
Russia	18 131 (14 973 to 21 617)	26 (21 to 31)	-6.3 (-10.7 to -1.7)	438 198 (406 571 to 474 699)	580 (537 to 629)	-5.3 (-9.3 to -0.5)	128 736 (89 875 to 169 063)	171 (119 to 224)	-12.5 (-17.1 to -7.4)
Ukraine	4 891 (4 070 to 5 875)	23 (19 to 27)	-7.9 (-12.2 to -3.4)	131 267 (122 292 to 140 649)	553 (514 to 594)	-6.0 (-10.7 to -1.1)	38 613 (27 000 to 50 800)	164 (114 to 216)	-11.8 (-15.7 to -7.0)
**Central Europe**	**14 472 (11 636 to 17 677)**	**25 (20 to 31)**	**-3.4 (-7.0 to -0.1)**	**470 848 (432 271 to 514 381)**	**719 (660 to 788)**	**11.7 (8.0 to 17.8)**	**140 376 (96 822 to 186 402)**	**215 (150 to 284)**	**-0.3 (-5.7 to 4.9)**
Albania	283 (230 to 345)	20 (16 to 24)	7.0 (1.6 to 12.6)	10 113 (8 940 to 11 613)	656 (578 to 760)	18.1 (8.7 to 34.8)	3 067 (2 127 to 4 184)	201 (138 to 272)	4.6 (-7.1 to 18.2)
Bosnia and Herzegovina	377 (305 to 460)	21 (17 to 26)	35.4 (28.4 to 42.7)	20 738 (15 824 to 30 547)	912 (701 to 1 327)	101.8 (57.1 to 195.1)	6 992 (4 026 to 10 946)	307 (166 to 484)	94.0 (24.2 to 179.2)
Bulgaria	802 (648 to 977)	23 (19 to 28)	-11.7 (-17.0 to -6.2)	27 147 (24 635 to 29 431)	658 (598 to 719)	-5.4 (-10.2 to -0.9)	7 927 (5 561 to 10 402)	194 (133 to 256)	-12.1 (-14.4 to -10.1)
Croatia	480 (385 to 592)	22 (18 to 27)	-2.1 (-8.3 to 4.4)	16 534 (14 221 to 20 836)	683 (588 to 859)	21.1 (6.1 to 54.0)	5 175 (3 383 to 7 451)	216 (141 to 309)	28.6 (2.9 to 59.6)
Czech Republic	1 531 (1 239 to 1 884)	30 (24 to 36)	-0.2 (-5.7 to 5.3)	53 162 (48 495 to 57 698)	867 (787 to 946)	21.1 (14.9 to 28.3)	15 686 (11 021 to 20 443)	257 (179 to 335)	19.7 (17.7 to 21.5)
Hungary	1 210 (957 to 1 508)	25 (20 to 31)	-16.0 (-21.7 to -10.9)	35 242 (31 969 to 38 666)	646 (583 to 715)	5.8 (-0.1 to 11.7)	10 322 (7 209 to 13 414)	190 (132 to 252)	-0.4 (-3.3 to 1.9)
Macedonia	209 (169 to 254)	21 (17 to 25)	15.5 (10.6 to 20.5)	7 307 (6 632 to 8 049)	625 (566 to 691)	21.8 (14.9 to 29.8)	2 171 (1 525 to 2 851)	186 (130 to 247)	9.2 (5.1 to 12.9)
Montenegro	73 (60 to 89)	24 (19 to 29)	4.7 (0.8 to 8.8)	2 374 (2 168 to 2 590)	694 (632 to 760)	9.6 (4.9 to 14.7)	699 (481 to 920)	205 (141 to 273)	5.1 (2.2 to 7.7)
Poland	5 062 (4 038 to 6 226)	27 (21 to 32)	0.9 (-4.9 to 6.5)	154 441 (141 901 to 167 554)	710 (650 to 775)	15.0 (9.8 to 20.5)	45 323 (31 757 to 59 688)	210 (146 to 276)	3.6 (0.4 to 6.1)
Romania	2 468 (1 986 to 3 041)	26 (21 to 31)	-12.7 (-18.6 to -7.0)	75 167 (68 819 to 81 212)	689 (628 to 748)	-9.0 (-14.0 to -4.5)	21 964 (15 288 to 28 702)	203 (141 to 265)	-19.5 (-22.4 to -17.1)
Serbia	912 (735 to 1 114)	22 (17 to 26)	12.0 (7.8 to 16.4)	35 555 (30 423 to 44 664)	732 (625 to 915)	38.0 (20.8 to 73.7)	11 103 (7 134 to 15 732)	228 (147 to 323)	33.7 (7.1 to 66.9)

Location	Incidence (95% UI)			Prevalence (95% UI)			YLDs (95% UI)		
	2016 counts	2016 age-standardised rates per 100,000	Percentage change in age-standardised rates between 1990 and 2016	2016 counts	2016 age-standardised rates per 100,000	Percentage change in age-standardised rates between 1990 and 2016	2016 counts	2016 age-standardised rates per 100,000	Percentage change in age-standardised rates between 1990 and 2016
Slovakia	736 (588 to 909)	27 (22 to 33)	-8.6 (-13.0 to -3.5)	22 381 (20 446 to 24 275)	722 (659 to 787)	6.9 (2.1 to 12.8)	6 583 (4 592 to 8 676)	213 (149 to 280)	1.7 (-0.6 to 3.6)
Slovenia	328 (261 to 411)	31 (25 to 38)	-4.3 (-10.2 to 2.6)	10 687 (9 805 to 11 494)	863 (786 to 932)	14.2 (9.2 to 19.4)	3 168 (2 198 to 4 148)	256 (180 to 334)	14.6 (12.6 to 16.2)
**Central Asia**	**7 326 (6 089 to 8 605)**	**17 (14 to 20)**	**-2.9 (-5.8 to 0.2)**	**193 429 (178 778 to 211 820)**	**461 (427 to 504)**	**3.6 (-0.3 to 10.4)**	**64 682 (45 873 to 84 492)**	**153 (109 to 198)**	**-3.6 (-9.4 to 2.6)**
Armenia	226 (187 to 267)	16 (13 to 19)	-14.0 (-18.8 to -8.8)	8 434 (7 317 to 9 873)	565 (490 to 662)	-14.8 (-22.5 to -7.1)	2 580 (1 763 to 3 521)	173 (121 to 232)	-23.8 (-29.5 to -16.8)
Azerbaijan	763 (638 to 896)	16 (13 to 18)	-8.5 (-12.4 to -4.3)	24 214 (21 772 to 28 207)	477 (430 to 552)	9.2 (0.5 to 26.6)	7 901 (5 449 to 10 620)	156 (106 to 207)	0.3 (-12.7 to 14.7)
Georgia	318 (264 to 378)	16 (14 to 20)	-7.2 (-12.0 to -2.5)	9 949 (8 937 to 11 723)	479 (431 to 563)	3.0 (-6.6 to 20.0)	3 192 (2 204 to 4 265)	155 (107 to 209)	2.4 (-12.5 to 19.4)
Kazakhstan	1 733 (1 444 to 2 045)	20 (17 to 24)	4.7 (-0.8 to 10.2)	41 634 (38 617 to 44 787)	490 (455 to 526)	5.8 (1.8 to 10.2)	12 780 (9 048 to 16 640)	149 (104 to 194)	-4.7 (-9.5 to -0.6)
Kyrgyzstan	468 (392 to 552)	16 (13 to 18)	-17.5 (-21.7 to -12.9)	11 354 (10 480 to 12 252)	415 (385 to 447)	-10.6 (-16.8 to -4.5)	3 772 (2 607 to 4 837)	137 (96 to 177)	-15.9 (-19.2 to -12.4)
Mongolia	330 (272 to 392)	21 (18 to 26)	42.4 (27.8 to 53.5)	7 311 (6 727 to 7 877)	492 (455 to 528)	42.1 (35.2 to 49.2)	2 530 (1 802 to 3 250)	169 (120 to 221)	26.1 (20.2 to 31.5)
Tajikistan	650 (537 to 777)	15 (12 to 17)	-15.6 (-20.0 to -11.0)	19 024 (15 773 to 25 268)	513 (426 to 687)	15.8 (-3.3 to 55.7)	7 088 (4 522 to 10 048)	192 (124 to 270)	15.2 (-14.5 to 46.8)
Turkmenistan	418 (349 to 495)	15 (13 to 18)	-1.8 (-6.2 to 3.0)	10 583 (9 726 to 11 410)	404 (374 to 434)	5.5 (1.6 to 10.1)	3 461 (2 431 to 4 509)	131 (93 to 171)	-6.3 (-9.8 to -3.3)
Uzbekistan	2 419 (2 005 to 2 869)	16 (13 to 19)	3.1 (-1.4 to 7.6)	60 928 (55 988 to 66 187)	422 (390 to 457)	6.7 (3.0 to 10.8)	19 742 (13 752 to 25 481)	136 (96 to 178)	-2.9 (-6.7 to 0.6)
**Latin America and Caribbean**	**29 643 (24 762 to 35 192)**	**11 (9 to 13)**	**-4.0 (-7.5 to -0.9)**	**818 410 (762 257 to 878 274)**	**294 (274 to 315)**	**5.3 (3.0 to 8.2)**	**270 671 (187 682 to 353 406)**	**97 (68 to 125)**	**-8.5 (-11.9 to -5.4)**
**Central Latin America**	**11 222 (9 331 to 13 540)**	**9 (8 to 11)**	**-17.7 (-21.7 to -14.6)**	**307 563 (282 911 to 332 816)**	**257 (238 to 278)**	**-7.0 (-8.9 to -5.2)**	**100 595 (70 996 to 132 316)**	**84 (59 to 110)**	**-19.6 (-22.7 to -17.0)**
Colombia	2 156 (1 756 to 2 597)	9 (8 to 11)	-15.4 (-21.0 to -10.5)	66 563 (60 889 to 72 436)	279 (255 to 302)	3.4 (-0.3 to 8.2)	21 163 (14 900 to 27 615)	88 (62 to 117)	-9.1 (-14.3 to -4.0)
Costa Rica	228 (188 to 277)	10 (8 to 12)	16.9 (12.1 to 21.8)	6 917 (6 354 to 7 464)	276 (254 to 298)	25.5 (21.5 to 29.7)	2 091 (1 445 to 2 741)	83 (58 to 110)	13.6 (9.8 to 16.8)
El Salvador	281 (230 to 341)	10 (8 to 12)	-22.8 (-45.2 to -4.6)	9 398 (7 184 to 14 115)	365 (273 to 567)	-11.6 (-23.6 to 2.8)	3 360 (1 816 to 5 340)	130 (73 to 201)	-25.6 (-33.6 to -6.4)
Guatemala	779 (644 to 933)	10 (9 to 12)	-3.5 (-27.6 to 11.0)	17 741 (15 646 to 21 431)	281 (245 to 354)	6.1 (-6.1 to 16.5)	6 507 (4 427 to 8 625)	103 (69 to 138)	-8.5 (-17.2 to 4.5)
Honduras	335 (277 to 400)	9 (7 to 10)	19.1 (14.1 to 24.4)	8 966 (7 843 to 10 412)	248 (218 to 286)	39.5 (26.1 to 58.2)	3 312 (2 304 to 4 387)	91 (64 to 118)	24.1 (10.4 to 38.7)
Mexico	5 332 (4 462 to 6 397)	9 (7 to 10)	-28.1 (-30.7 to -25.3)	139 090 (129 389 to 148 588)	228 (213 to 243)	-19.9 (-22.2 to -17.6)	44 503 (31 370 to 57 726)	73 (51 to 94)	-30.4 (-33.4 to -28.0)
Nicaragua	245 (203 to 294)	8 (7 to 10)	-2.5 (-7.8 to 2.0)	8 644 (6 614 to 12 634)	328 (248 to 496)	-13.2 (-24.5 to -0.5)	3 109 (1 770 to 4 708)	119 (69 to 186)	-24.1 (-31.8 to -9.0)
Panama	196 (161 to 239)	10 (8 to 12)	18.2 (13.3 to 23.0)	5 532 (5 110 to 5 946)	286 (265 to 307)	25.3 (21.3 to 29.1)	1 746 (1 223 to 2 280)	90 (63 to 116)	11.2 (6.0 to 15.9)
Venezuela	1 670 (1 361 to 2 035)	11 (9 to 13)	12.7 (6.7 to 19.5)	44 713 (41 362 to 48 100)	297 (276 to 320)	21.0 (16.4 to 26.5)	14 013 (9 819 to 17 986)	93 (65 to 121)	6.5 (0.5 to 11.8)
**Andean Latin America**	**3 165 (2 660 to 3 715)**	**11 (9 to 13)**	**-1.6 (-10.4 to 5.4)**	**84 423 (78 363 to 91 546)**	**307 (286 to 332)**	**12.3 (9.2 to 16.1)**	**28 402 (19 886 to 36 185)**	**103 (73 to 134)**	**-4.9 (-9.2 to -1.2)**
Bolivia	556 (466 to 651)	10 (9 to 12)	-9.5 (-12.9 to -6.1)	13 641 (12 684 to 14 781)	279 (261 to 300)	6.9 (3.5 to 10.5)	4 925 (3 474 to 6 334)	101 (71 to 129)	-7.0 (-11.8 to -2.6)
Ecuador	1 022 (831 to 1 267)	13 (10 to 16)	20.2 (6.8 to 44.5)	23 348 (21 769 to 25 210)	308 (288 to 332)	10.8 (7.5 to 14.4)	7 647 (5 374 to 9 971)	101 (70 to 130)	-6.2 (-10.5 to -2.3)
Peru	1 588 (1 327 to 1 850)	10 (8 to 12)	-9.9 (-22.7 to -0.4)	47 434 (43 711 to 51 931)	316 (292 to 345)	14.6 (9.7 to 20.1)	15 433 (10 928 to 20 157)	103 (72 to 133)	-3.7 (-9.3 to 1.6)
**Caribbean**	**2 389 (1 996 to 2 815)**	**11 (9 to 13)**	**20.8 (15.6 to 27.9)**	**77 053 (64 348 to 96 673)**	**340 (285 to 426)**	**46.7 (24.5 to 82.5)**	**26 830 (18 494 to 36 042)**	**118 (81 to 157)**	**31.5 (12.5 to 51.5)**
Antigua and Barbuda	4 (4 to 5)	10 (8 to 12)	11.9 (8.0 to 16.2)	141 (129 to 152)	313 (288 to 338)	18.6 (13.3 to 24.8)	44 (30 to 57)	96 (67 to 126)	7.7 (2.8 to 12.0)
The Bahamas	20 (16 to 23)	10 (9 to 12)	5.7 (1.7 to 9.9)	583 (539 to 626)	288 (267 to 309)	17.0 (12.5 to 21.2)	184 (129 to 239)	91 (64 to 118)	8.4 (4.1 to 12.6)
Barbados	12 (10 to 14)	9 (8 to 11)	17.1 (13.8 to 21.0)	416 (386 to 448)	282 (261 to 304)	23.3 (18.3 to 29.5)	127 (88 to 166)	86 (59 to 113)	12.7 (8.1 to 16.9)
Belize	20 (17 to 24)	11 (9 to 13)	22.2 (4.8 to 32.1)	472 (437 to 509)	284 (265 to 304)	26.9 (21.3 to 33.4)	163 (115 to 209)	97 (69 to 127)	19.1 (13.9 to 24.0)
Bermuda	4 (3 to 4)	11 (9 to 13)	-7.5 (-11.3 to -3.2)	116 (107 to 125)	326 (299 to 349)	11.4 (7.1 to 15.6)	35 (24 to 46)	97 (67 to 128)	2.7 (-1.2 to 5.6)
Cuba	601 (493 to 727)	10 (9 to 12)	10.8 (4.3 to 17.4)	19 856 (18 301 to 21 548)	302 (277 to 328)	15.9 (10.7 to 22.0)	5 843 (4 104 to 7 683)	89 (63 to 117)	6.0 (2.6 to 9.0)
Dominica	4 (3 to 4)	10 (8 to 11)	30.8 (26.2 to 35.5)	104 (96 to 112)	278 (255 to 299)	31.7 (25.9 to 38.7)	34 (24 to 44)	91 (64 to 118)	22.7 (17.6 to 27.8)
Dominican Republic	524 (435 to 627)	10 (8 to 12)	28.4 (23.3 to 33.7)	14 598 (13 458 to 15 767)	293 (272 to 316)	35.3 (29.7 to 41.7)	4 831 (3 386 to 6 259)	97 (67 to 126)	15.2 (9.6 to 20.5)
Grenada	6 (5 to 7)	11 (9 to 13)	24.6 (20.0 to 29.2)	154 (143 to 167)	294 (272 to 317)	23.0 (17.6 to 28.9)	52 (36 to 67)	98 (71 to 127)	13.2 (8.6 to 17.9)
Guyana	41 (35 to 49)	11 (9 to 13)	10.4 (5.9 to 15.2)	959 (891 to 1 021)	254 (237 to 270)	16.6 (12.5 to 20.8)	341 (242 to 437)	90 (64 to 115)	7.9 (4.2 to 11.2)
Haiti	627 (511 to 773)	11 (9 to 14)	29.8 (12.3 to 57.9)	23 507 (12 535 to 41 847)	487 (258 to 865)	154.7 (35.5 to 349.4)	9 479 (5 039 to 15 568)	198 (104 to 316)	137.1 (29.4 to 255.5)
Jamaica	131 (109 to 154)	9 (8 to 11)	36.2 (31.6 to 41.3)	3 831 (3 517 to 4 127)	272 (251 to 293)	33.2 (27.3 to 40.4)	1 256 (882 to 1 636)	89 (62 to 116)	23.1 (16.7 to 29.2)
Puerto Rico	198 (164 to 237)	11 (9 to 13)	19.7 (13.8 to 26.0)	6 043 (5 604 to 6 453)	318 (294 to 340)	31.3 (26.3 to 36.5)	1 773 (1 235 to 2 336)	94 (65 to 124)	24.1 (20.8 to 26.5)
Saint Lucia	9 (7 to 10)	10 (8 to 12)	17.0 (12.9 to 21.1)	265 (246 to 284)	283 (263 to 303)	25.4 (21.0 to 30.5)	86 (60 to 111)	91 (63 to 117)	13.0 (8.7 to 16.9)
Saint Vincent and the Grenadines	6 (5 to 7)	11 (9 to 13)	19.9 (14.6 to 25.7)	164 (152 to 176)	289 (268 to 309)	18.9 (14.5 to 25.0)	55 (39 to 71)	97 (69 to 125)	12.2 (8.6 to 15.4)
Suriname	28 (23 to 33)	10 (9 to 12)	24.7 (20.1 to 29.7)	771 (715 to 827)	282 (263 to 302)	24.5 (18.3 to 29.9)	268 (188 to 343)	98 (68 to 126)	13.5 (8.4 to 18.9)
Trinidad and Tobago	67 (56 to 80)	11 (9 to 13)	16.7 (3.8 to 25.8)	2 109 (1 956 to 2 250)	292 (271 to 312)	33.4 (28.3 to 39.3)	676 (471 to 873)	93 (66 to 121)	21.3 (16.5 to 25.2)
Virgin Islands, U.S.	5 (4 to 6)	10 (9 to 12)	14.3 (10.8 to 18.6)	152 (141 to 163)	282 (261 to 303)	17.1 (12.5 to 22.2)	45 (31 to 58)	83 (58 to 110)	2.9 (-1.9 to 7.4)
**Tropical Latin America**	**12 866 (10 633 to 15 311)**	**12 (10 to 15)**	**4.8 (0.9 to 8.9)**	**349 371 (324 349 to 372 924)**	**321 (298 to 342)**	**9.2 (6.5 to 12.0)**	**113 423 (78 235 to 146 937)**	**104 (73 to 134)**	**-3.1 (-6.1 to -0.5)**
Brazil	12 515 (10 332 to 14 893)	12 (10 to 15)	4.3 (0.5 to 8.6)	340 283 (315 859 to 363 194)	322 (299 to 343)	9.0 (6.2 to 11.8)	110 052 (77 407 to 142 398)	104 (73 to 134)	-3.4 (-6.7 to -0.7)
Paraguay	351 (288 to 425)	10 (9 to 12)	27.9 (22.7 to 33.7)	9 087 (8 182 to 9 918)	287 (261 to 312)	19.9 (13.6 to 25.4)	3 112 (2 204 to 4 017)	98 (68 to 127)	8.9 (4.5 to 12.6)
**Southeast Asia, East Asia, and Oceania**	**89 309 (74 486 to 106 785)**	**8 (7 to 10)**	**8.0 (-4.2 to 18.6)**	**3 110 967 (2 893 184 to 3 343 031)**	**266 (248 to 286)**	**35.2 (31.1 to 39.2)**	**1 028 704 (721 672 to 1 332 336)**	**88 (61 to 116)**	**13.6 (8.6 to 18.6)**
**East Asia**	**61 457 (51 202 to 73 769)**	**8 (7 to 10)**	**7.8 (-6.0 to 19.9)**	**2 242 884 (2 093 827 to 2 408 771)**	**267 (249 to 286)**	**33.6 (30.1 to 37.5)**	**668 455 (467 975 to 875 899)**	**80 (55 to 105)**	**6.3 (0.1 to 11.5)**
China	59 446 (49 502 to 71 371)	8 (7 to 10)	7.2 (-6.9 to 19.4)	2 181 134 (2 035 736 to 2 344 026)	268 (250 to 287)	33.7 (30.0 to 37.6)	650 647 (455 193 to 857 979)	80 (56 to 105)	6.1 (0.1 to 11.5)
North Korea	1 045 (872 to 1 246)	8 (7 to 9)	43.7 (30.1 to 58.6)	27 602 (25 896 to 29 469)	205 (192 to 218)	36.2 (31.9 to 40.7)	9 653 (6 845 to 12 368)	71 (50 to 91)	32.9 (27.5 to 38.0)
Taiwan (Province of China)	966 (806 to 1 160)	8 (7 to 10)	20.5 (12.0 to 30.1)	34 148 (32 119 to 36 184)	249 (233 to 264)	27.9 (23.1 to 33.4)	10 135 (7 040 to 13 176)	74 (52 to 96)	15.2 (10.8 to 19.2)
**Southeast Asia**	**27 368 (23 241 to 32 480)**	**9 (7 to 10)**	**8.4 (-11.1 to 22.3)**	**857 892 (762 457 to 1 016 904)**	**266 (236 to 317)**	**39.9 (27.5 to 51.4)**	**319 521 (223 860 to 421 673)**	**100 (69 to 131)**	**24.1 (15.1 to 35.2)**
Cambodia	598 (510 to 702)	8 (7 to 9)	-18.6 (-52.7 to 14.8)	28 336 (16 973 to 56 194)	486 (262 to 1 021)	-19.7 (-31.1 to 8.3)	12 239 (4 707 to 22 426)	210 (68 to 405)	-27.9 (-36.1 to 30.9)
Indonesia	9 682 (8 241 to 11 388)	8 (6 to 9)	21.6 (16.0 to 27.9)	302 914 (276 325 to 337 119)	235 (214 to 262)	37.5 (23.7 to 50.2)	113 989 (80 375 to 146 983)	89 (63 to 114)	23.7 (11.9 to 36.6)
Laos	262 (224 to 308)	7 (6 to 8)	-31.1 (-63.6 to 9.6)	5 655 (5 223 to 6 094)	188 (175 to 203)	44.7 (38.2 to 50.3)	2 222 (1 588 to 2 836)	74 (52 to 95)	29.6 (23.7 to 35.8)
Malaysia	1 428 (1 189 to 1 720)	9 (8 to 11)	26.2 (20.1 to 32.8)	40 315 (38 033 to 42 731)	265 (251 to 281)	35.8 (32.0 to 39.4)	12 680 (8 910 to 16 453)	83 (58 to 107)	14.7 (9.7 to 19.5)
Maldives	12 (10 to 14)	7 (6 to 8)	2.4 (-1.6 to 6.6)	367 (341 to 392)	215 (201 to 228)	29.1 (24.6 to 34.2)	113 (78 to 147)	66 (46 to 86)	2.5 (-4.6 to 9.5)
Mauritius	50 (42 to 59)	8 (7 to 10)	33.3 (27.3 to 40.5)	1 631 (1 541 to 1 733)	237 (223 to 252)	48.5 (44.1 to 54.4)	505 (352 to 659)	73 (51 to 95)	34.0 (28.5 to 38.9)
Myanmar	2 033 (1 726 to 2 372)	8 (7 to 9)	31.0 (18.1 to 40.3)	80 109 (62 821 to 107 077)	308 (241 to 412)	69.1 (30.6 to 124.2)	31 374 (20 011 to 43 390)	121 (78 to 171)	44.2 (14.0 to 99.0)
Philippines	5 037 (3 990 to 6 738)	10 (8 to 13)	7.6 (-11.4 to 25.5)	121 802 (104 890 to 151 783)	261 (222 to 333)	27.2 (11.4 to 39.9)	44 523 (31 246 to 60 001)	96 (65 to 134)	15.6 (4.1 to 33.0)
Sri Lanka	959 (815 to 1 126)	10 (8 to 12)	-66.2 (-84.4 to -29.6)	57 704 (36 797 to 102 116)	556 (356 to 983)	99.2 (50.5 to 166.8)	20 736 (8 980 to 34 986)	199 (85 to 355)	87.0 (16.4 to 134.7)
Seychelles	5 (4 to 6)	9 (8 to 11)	21.3 (15.6 to 27.7)	130 (123 to 138)	252 (237 to 268)	37.3 (29.0 to 43.5)	42 (29 to 54)	80 (56 to 105)	19.0 (11.3 to 26.5)
Thailand	3 369 (2 791 to 4 101)	10 (8 to 12)	22.5 (16.3 to 29.8)	105 914 (100 062 to 112 089)	277 (261 to 294)	36.5 (32.4 to 40.6)	32 952 (23 173 to 43 403)	86 (61 to 114)	14.7 (8.8 to 20.1)
Timor-Leste	42 (36 to 49)	7 (6 to 8)	-72.8 (-88.7 to -31.5)	3 582 (1 609 to 8 117)	883 (368 to 2 082)	37.7 (17.1 to 62.5)	1 546 (365 to 3 333)	399 (76 to 783)	26.4 (18.8 to 31.3)
Vietnam	3 856 (3 246 to 4 590)	8 (7 to 10)	44.0 (37.3 to 51.3)	108 259 (101 561 to 115 097)	228 (214 to 242)	59.7 (54.2 to 65.6)	35 988 (25 540 to 46 137)	75 (53 to 97)	36.0 (28.4 to 42.8)

Location	Incidence (95% UI)			Prevalence (95% UI)			YLDs (95% UI)		
	2016 counts	2016 age-standardised rates per 100,000	Percentage change in age-standardised rates between 1990 and 2016	2016 counts	2016 age-standardised rates per 100,000	Percentage change in age-standardised rates between 1990 and 2016	2016 counts	2016 age-standardised rates per 100,000	Percentage change in age-standardised rates between 1990 and 2016
**Oceania**	**484 (413 to 567)**	**9 (7 to 10)**	**9.6 (-4.1 to 19.0)**	**10 191 (9 482 to 10 930)**	**198 (185 to 212)**	**39.5 (34.5 to 46.4)**	**3 996 (2 832 to 5 063)**	**77 (55 to 98)**	**32.9 (25.9 to 40.3)**
American Samoa	4 (3 to 4)	10 (8 to 11)	-0.4 (-4.0 to 3.5)	99 (90 to 110)	268 (247 to 296)	21.1 (14.1 to 34.5)	34 (24 to 44)	91 (63 to 118)	14.0 (3.0 to 25.5)
Federated States of Micronesia	4 (4 to 5)	8 (7 to 10)	16.7 (12.5 to 21.6)	92 (85 to 98)	191 (179 to 204)	25.6 (21.1 to 30.9)	35 (24 to 44)	72 (50 to 91)	14.9 (7.4 to 22.7)
Fiji	33 (29 to 39)	8 (7 to 9)	28.1 (23.2 to 33.8)	864 (809 to 924)	192 (180 to 205)	37.4 (34.0 to 40.8)	310 (219 to 399)	69 (49 to 89)	31.7 (23.2 to 40.9)
Guam	8 (7 to 10)	10 (8 to 11)	24.6 (20.0 to 30.0)	232 (217 to 248)	261 (245 to 280)	28.9 (23.5 to 37.1)	72 (51 to 94)	82 (58 to 106)	23.8 (16.0 to 32.2)
Kiribati	4 (4 to 5)	8 (6 to 9)	33.9 (28.1 to 39.9)	87 (80 to 95)	169 (156 to 188)	50.3 (42.2 to 65.4)	36 (26 to 46)	70 (51 to 90)	46.5 (35.1 to 58.9)
Marshall Islands	3 (3 to 4)	8 (7 to 10)	19.1 (14.5 to 24.4)	62 (59 to 67)	182 (172 to 194)	20.8 (17.5 to 24.2)	23 (16 to 30)	68 (48 to 87)	13.3 (7.8 to 17.7)
Northern Mariana Islands	6 (5 to 7)	9 (8 to 11)	7.7 (4.5 to 11.3)	168 (157 to 180)	263 (248 to 280)	9.4 (6.4 to 14.0)	51 (36 to 67)	79 (55 to 103)	0.6 (-5.5 to 6.7)
Papua New Guinea	345 (293 to 405)	9 (8 to 11)	4.4 (-12.6 to 16.0)	6 900 (6 389 to 7 437)	196 (181 to 211)	44.5 (37.9 to 53.8)	2 782 (1 949 to 3 537)	79 (56 to 100)	38.0 (29.0 to 47.8)
Samoa	8 (7 to 10)	8 (7 to 10)	14.1 (10.1 to 18.8)	199 (185 to 217)	225 (209 to 244)	35.4 (29.2 to 44.7)	72 (52 to 93)	81 (58 to 105)	27.0 (18.6 to 36.5)
Solomon Islands	25 (21 to 29)	9 (7 to 10)	20.4 (15.9 to 25.8)	496 (461 to 532)	191 (178 to 204)	30.2 (26.8 to 33.6)	198 (142 to 252)	76 (54 to 96)	25.8 (19.4 to 32.5)
Tonga	4 (4 to 5)	8 (7 to 10)	16.7 (10.4 to 23.5)	96 (89 to 102)	204 (193 to 216)	25.5 (22.3 to 29.0)	34 (24 to 44)	72 (51 to 93)	16.8 (10.5 to 22.9)
Vanuatu	11 (9 to 13)	8 (7 to 9)	26.5 (21.5 to 32.5)	231 (213 to 251)	189 (175 to 207)	39.0 (34.0 to 44.7)	92 (65 to 117)	75 (53 to 95)	35.8 (27.9 to 43.7)
**North Africa and Middle East**	**70 806 (37 655 to 152 329)**	**23 (12 to 48)**	**69.9 (3.5 to 204.3)**	**1 418 064 (954 155 to 2 500 646)**	**520 (347 to 917)**	**2.4 (-5.6 to 11.3)**	**555 149 (261 494 to 932 763)**	**204 (93 to 342)**	**-7.8 (-10.6 to -4.6)**
**North Africa and Middle East**	**70 806 (37 655 to 152 329)**	**23 (12 to 48)**	**69.9 (3.5 to 204.3)**	**1 418 064 (954 155 to 2 500 646)**	**520 (347 to 917)**	**2.4 (-5.6 to 11.3)**	**555 149 (261 494 to 932 763)**	**204 (93 to 342)**	**-7.8 (-10.6 to -4.6)**
Afghanistan	8 820 (2 613 to 22 743)	46 (14 to 120)	173.3 (-1.9 to 426.1)	183 794 (62 817 to 456 108)	1 672 (496 to 4 409)	-17.9 (-27.7 to 33.7)	89 777 (12 223 to 201 129)	808 (80 to 1 813)	-21.5 (-25.3 to 17.2)
Algeria	1 903 (1 621 to 2 230)	9 (8 to 11)	-5.9 (-9.0 to -2.6)	60 330 (54 573 to 70 644)	314 (285 to 364)	14.2 (6.1 to 30.9)	20 334 (14 194 to 27 083)	105 (73 to 136)	2.1 (-10.2 to 16.1)
Bahrain	83 (70 to 97)	10 (8 to 12)	-7.0 (-12.2 to -2.8)	3 079 (2 868 to 3 300)	331 (310 to 353)	11.3 (7.7 to 15.8)	945 (653 to 1 237)	101 (70 to 134)	-4.8 (-10.7 to 0.4)
Egypt	4 399 (3 703 to 5 313)	9 (8 to 11)	25.7 (17.0 to 45.7)	111 941 (102 921 to 125 098)	259 (238 to 291)	24.4 (19.8 to 30.8)	38 399 (26 579 to 50 299)	89 (62 to 115)	6.5 (0.2 to 11.5)
Iran	4 667 (3 924 to 5 563)	11 (9 to 13)	-55.4 (-74.9 to -20.7)	229 586 (155 778 to 404 245)	580 (387 to 1 032)	-18.9 (-28.8 to -4.1)	81 521 (37 897 to 147 671)	205 (91 to 352)	-30.6 (-36.7 to -11.2)
Iraq	10 256 (3 462 to 27 476)	48 (17 to 127)	249.5 (28.4 to 679.9)	225 968 (90 737 to 554 546)	1 665 (648 to 4 159)	-9.0 (-20.4 to 12.7)	99 021 (15 086 to 204 659)	729 (102 to 1 612)	-12.9 (-17.4 to 17.4)
Jordan	740 (406 to 1 480)	18 (10 to 35)	69.6 (-3.7 to 243.6)	9 768 (9 065 to 10 526)	278 (261 to 298)	-6.8 (-10.5 to -1.6)	3 047 (2 121 to 3 995)	86 (59 to 113)	-19.2 (-25.0 to -13.6)
Kuwait	241 (203 to 286)	11 (9 to 13)	-66.3 (-87.2 to -21.2)	9 011 (8 220 to 10 287)	386 (355 to 434)	6.4 (1.9 to 13.5)	2 773 (1 876 to 3 688)	117 (80 to 155)	0.7 (-5.5 to 7.0)
Lebanon	403 (265 to 726)	14 (9 to 25)	-70.7 (-83.4 to -45.5)	56 605 (20 597 to 136 433)	1 886 (682 to 4 545)	-31.7 (-36.6 to -20.6)	22 223 (2 491 to 48 738)	743 (85 to 1 623)	-38.6 (-41.5 to -5.2)
Libya	793 (363 to 1 935)	25 (12 to 60)	159.7 (26.7 to 516.4)	22 170 (11 425 to 46 473)	689 (367 to 1 419)	101.6 (19.6 to 259.2)	8 425 (2 507 to 17 541)	259 (78 to 522)	103.7 (-16.5 to 191.3)
Morocco	1 447 (1 227 to 1 691)	9 (7 to 10)	-3.7 (-8.8 to 0.7)	45 317 (42 207 to 48 710)	274 (255 to 294)	7.2 (3.0 to 11.1)	15 497 (10 843 to 20 337)	93 (66 to 120)	-6.7 (-11.5 to -2.3)
Palestine	296 (236 to 390)	10 (8 to 14)	-23.7 (-52.7 to 12.2)	12 461 (6 914 to 25 744)	726 (361 to 1 606)	-13.6 (-23.4 to 11.2)	4 970 (1 617 to 9 539)	300 (79 to 571)	-19.1 (-24.1 to 5.2)
Oman	335 (275 to 408)	11 (9 to 13)	-5.7 (-9.6 to -1.8)	10 810 (10 131 to 11 636)	331 (312 to 356)	1.8 (-1.2 to 4.9)	3 234 (2 255 to 4 289)	98 (69 to 129)	-14.1 (-18.0 to -10.5)
Qatar	216 (179 to 262)	13 (11 to 15)	-5.1 (-8.1 to -1.9)	7 170 (6 638 to 7 680)	409 (382 to 435)	1.5 (-1.5 to 4.4)	2 153 (1 499 to 2 825)	122 (84 to 159)	-10.8 (-14.8 to -7.2)
Saudi Arabia	1 963 (1 661 to 2 322)	11 (9 to 13)	-11.4 (-14.4 to -8.5)	58 376 (54 918 to 61 970)	322 (304 to 341)	-6.9 (-9.5 to -4.0)	17 431 (12 034 to 22 901)	96 (67 to 125)	-22.7 (-26.8 to -19.2)
Sudan	2 560 (1 813 to 3 896)	12 (9 to 18)	7.7 (-14.3 to 33.5)	58 880 (44 935 to 91 767)	349 (264 to 552)	25.3 (17.9 to 36.7)	23 623 (13 128 to 36 438)	137 (80 to 217)	15.4 (2.5 to 23.6)
Syria	17 389 (3 414 to 52 925)	170 (34 to 524)	2 048.2 (321.7 to 6 687.1)	96 297 (39 123 to 236 466)	1 023 (439 to 2 407)	249.0 (82.5 to 513.7)	40 239 (6 410 to 87 857)	409 (77 to 861)	255.8 (2.2 to 396.6)
Tunisia	554 (466 to 677)	10 (9 to 12)	9.9 (2.4 to 26.0)	16 655 (15 460 to 18 006)	291 (270 to 314)	12.4 (8.7 to 16.7)	5 216 (3 641 to 6 785)	91 (64 to 118)	-3.7 (-9.6 to 1.7)
Turkey	4 271 (3 418 to 5 590)	11 (9 to 14)	-7.8 (-18.6 to 14.7)	125 717 (115 646 to 140 377)	322 (297 to 358)	-0.4 (-6.8 to 10.4)	38 469 (26 500 to 52 281)	98 (68 to 130)	-18.7 (-26.7 to -10.6)
United Arab Emirates	823 (682 to 987)	12 (10 to 14)	-7.4 (-10.0 to -4.7)	28 515 (26 588 to 30 555)	352 (330 to 376)	-4.7 (-8.1 to -1.7)	8 818 (6 194 to 11 516)	108 (76 to 140)	-18.2 (-23.5 to -13.3)
Yemen	8 576 (3 140 to 21 093)	53 (20 to 127)	421.0 (95.8 to 1 156.8)	43 955 (32 193 to 71 641)	377 (283 to 588)	16.1 (3.3 to 34.4)	18 223 (9 798 to 28 733)	155 (88 to 241)	8.9 (-10.7 to 22.0)
**South Asia**	**104 338 (88 380 to 123 098)**	**13 (11 to 15)**	**-0.1 (-4.0 to 4.3)**	**2 285 834 (2 164 545 to 2 421 743)**	**276 (262 to 292)**	**20.2 (17.9 to 22.9)**	**872 774 (628 736 to 1 100 508)**	**105 (75 to 133)**	**8.8 (5.6 to 11.9)**
**South Asia**	**104 338 (88 380 to 123 098)**	**13 (11 to 15)**	**-0.1 (-4.0 to 4.3)**	**2 285 834 (2 164 545 to 2 421 743)**	**276 (262 to 292)**	**20.2 (17.9 to 22.9)**	**872 774 (628 736 to 1 100 508)**	**105 (75 to 133)**	**8.8 (5.6 to 11.9)**
Bangladesh	8 328 (7 133 to 9 748)	11 (9 to 12)	2.0 (-2.7 to 7.2)	204 120 (188 757 to 225 376)	269 (249 to 300)	29.9 (23.4 to 38.8)	74 036 (52 392 to 94 835)	98 (69 to 126)	7.4 (0.8 to 14.0)
Bhutan	50 (42 to 58)	12 (10 to 14)	0.6 (-2.9 to 4.2)	1 234 (1 157 to 1 316)	303 (285 to 323)	21.9 (17.8 to 26.6)	451 (315 to 578)	110 (78 to 140)	3.0 (-2.6 to 8.3)
India	83 052 (70 378 to 97 780)	13 (11 to 15)	-1.7 (-6.1 to 2.9)	1 793 497 (1 697 963 to 1 895 600)	275 (260 to 290)	18.3 (16.5 to 20.2)	680 589 (488 014 to 864 916)	104 (73 to 132)	7.1 (3.8 to 9.9)
Nepal	1 579 (1 339 to 1 842)	11 (10 to 13)	-0.5 (-4.1 to 4.0)	36 688 (33 301 to 41 354)	294 (269 to 330)	34.5 (24.9 to 51.5)	14 319 (9 991 to 18 620)	114 (82 to 148)	22.5 (10.2 to 36.3)
Pakistan	11 329 (9 644 to 13 245)	12 (10 to 14)	13.4 (9.0 to 18.3)	250 296 (232 835 to 271 572)	290 (271 to 311)	25.8 (19.8 to 34.2)	98 088 (69 442 to 125 494)	113 (80 to 144)	18.9 (11.5 to 26.8)
**Sub-Saharan Africa**	**51 466 (44 031 to 60 311)**	**11 (9 to 13)**	**-31.2 (-51.8 to -13.2)**	**1 076 409 (929 027 to 1 361 202)**	**286 (241 to 378)**	**11.5 (6.2 to 20.6)**	**447 296 (308 746 to 607 182)**	**118 (80 to 166)**	**5.1 (-3.2 to 11.4)**
**Southern sub-Saharan Africa**	**3 829 (3 227 to 4 565)**	**10 (9 to 12)**	**-20.9 (-36.6 to -11.5)**	**80 463 (74 754 to 86 891)**	**225 (210 to 246)**	**-13.2 (-15.3 to -11.1)**	**29 887 (21 112 to 38 311)**	**83 (58 to 106)**	**-17.0 (-19.3 to -15.0)**
Botswana	119 (100 to 143)	11 (9 to 13)	11.8 (7.9 to 16.1)	2 472 (2 315 to 2 637)	223 (211 to 237)	7.4 (4.8 to 9.8)	867 (613 to 1 132)	78 (55 to 102)	-3.4 (-14.7 to 7.4)
Lesotho	110 (92 to 130)	11 (9 to 13)	18.4 (14.3 to 22.6)	1 640 (1 523 to 1 746)	157 (148 to 166)	-10.9 (-13.6 to -7.9)	655 (463 to 830)	62 (44 to 79)	-12.3 (-18.0 to -6.4)
Namibia	113 (95 to 134)	9 (8 to 11)	-1.1 (-3.6 to 1.5)	2 214 (2 060 to 2 372)	201 (189 to 214)	3.0 (0.6 to 5.9)	818 (592 to 1 040)	74 (53 to 93)	-8.1 (-15.3 to -0.9)
South Africa	2 780 (2 325 to 3 341)	11 (9 to 13)	-26.5 (-43.2 to -15.6)	62 151 (57 382 to 68 075)	245 (227 to 271)	-16.3 (-18.6 to -13.8)	22 291 (15 686 to 28 796)	87 (62 to 112)	-21.8 (-24.7 to -19.3)
Swaziland	71 (59 to 84)	11 (9 to 14)	11.3 (7.7 to 15.2)	1 113 (1 038 to 1 188)	187 (177 to 198)	-10.8 (-13.1 to -8.6)	421 (297 to 540)	70 (49 to 89)	-16.7 (-23.8 to -9.6)
Zimbabwe	635 (543 to 737)	9 (7 to 10)	5.1 (1.6 to 8.2)	10 873 (10 083 to 11 693)	159 (150 to 169)	3.0 (0.9 to 5.3)	4 381 (3 130 to 5 584)	63 (45 to 81)	7.3 (0.7 to 15.4)
**Western sub-Saharan Africa**	**20 908 (17 804 to 24 655)**	**11 (9 to 13)**	**-6.2 (-13.7 to -1.8)**	**395 869 (363 045 to 437 892)**	**250 (231 to 277)**	**9.5 (5.2 to 17.8)**	**158 394 (113 239 to 203 232)**	**100 (71 to 130)**	**3.1 (-3.7 to 10.1)**
Benin	561 (478 to 659)	11 (9 to 13)	0.8 (-2.3 to 4.4)	10 163 (9 470 to 10 926)	228 (215 to 244)	3.1 (0.2 to 5.9)	4 115 (2 900 to 5 220)	92 (65 to 116)	-3.5 (-6.3 to -0.7)
Burkina Faso	892 (758 to 1 042)	10 (9 to 12)	-2.8 (-6.3 to 1.1)	15 229 (14 049 to 16 308)	213 (199 to 226)	11.1 (7.5 to 14.4)	6 197 (4 466 to 7 789)	86 (62 to 109)	2.9 (-0.4 to 6.3)
Cameroon	1 268 (1 067 to 1 501)	11 (9 to 13)	8.7 (2.9 to 20.4)	21 632 (19 978 to 23 305)	217 (203 to 231)	-1.3 (-4.6 to 2.9)	8 698 (6 217 to 11 079)	87 (63 to 112)	-6.5 (-10.5 to -2.3)
Cape Verde	29 (24 to 33)	11 (9 to 12)	10.5 (6.8 to 14.2)	656 (610 to 703)	272 (255 to 289)	15.9 (12.7 to 19.1)	226 (160 to 291)	93 (66 to 120)	3.2 (-1.0 to 7.3)
Chad	764 (649 to 893)	11 (9 to 13)	-33.8 (-62.8 to -3.9)	15 805 (13 146 to 21 138)	313 (246 to 451)	-0.8 (-7.3 to 5.4)	6 835 (4 557 to 9 568)	136 (83 to 201)	-4.9 (-9.0 to 1.0)
Cote d'Ivoire	1 249 (1 065 to 1 454)	11 (10 to 13)	-3.9 (-6.9 to -0.8)	22 245 (20 552 to 24 096)	224 (210 to 242)	3.0 (-0.5 to 8.5)	9 200 (6 501 to 11 670)	92 (65 to 117)	-0.9 (-5.3 to 3.6)
The Gambia	94 (80 to 111)	10 (8 to 11)	-5.8 (-8.2 to -3.5)	1 778 (1 634 to 1 953)	234 (214 to 266)	-7.7 (-13.1 to -3.5)	706 (503 to 907)	93 (65 to 119)	-13.0 (-17.5 to -8.1)
Ghana	1 426 (1 213 to 1 670)	11 (9 to 13)	15.4 (12.1 to 18.7)	27 832 (25 943 to 29 854)	241 (226 to 257)	22.1 (17.6 to 26.6)	10 669 (7 688 to 13 652)	92 (65 to 118)	12.5 (7.6 to 17.2)
Guinea	604 (513 to 703)	10 (8 to 12)	-6.5 (-9.3 to -3.7)	11 082 (10 311 to 11 936)	211 (198 to 227)	-8.6 (-12.0 to -4.1)	4 625 (3 343 to 5 789)	88 (63 to 111)	-13.1 (-16.9 to -9.1)
Guinea-Bissau	96 (82 to 112)	11 (9 to 13)	-7.2 (-9.6 to -5.0)	1 715 (1 565 to 1 922)	214 (196 to 243)	4.1 (-3.2 to 18.4)	734 (522 to 938)	91 (64 to 118)	-0.4 (-10.5 to 10.6)
Liberia	199 (170 to 231)	9 (8 to 11)	-86.9 (-94.5 to -64.0)	7 523 (4 646 to 14 173)	435 (259 to 842)	79.1 (16.7 to 184.2)	3 336 (1 370 to 5 825)	195 (73 to 357)	83.0 (-15.0 to 169.7)
Mali	901 (765 to 1 062)	10 (9 to 12)	-13.4 (-23.9 to -6.7)	17 945 (15 047 to 24 193)	271 (226 to 370)	25.6 (7.0 to 70.6)	7 390 (4 807 to 10 485)	111 (72 to 156)	17.5 (-9.1 to 51.1)
Mauritania	210 (179 to 246)	11 (9 to 13)	-32.2 (-59.2 to -8.5)	4 717 (4 392 to 5 065)	284 (266 to 304)	12.8 (10.2 to 15.5)	1 812 (1 265 to 2 324)	108 (77 to 136)	2.3 (-3.2 to 7.1)
Niger	968 (828 to 1 132)	10 (9 to 12)	-17.4 (-26.9 to -11.4)	15 871 (14 686 to 17 134)	211 (197 to 226)	-6.8 (-9.8 to -2.5)	6 532 (4 653 to 8 317)	87 (62 to 109)	-13.1 (-16.9 to -9.6)
Nigeria	10 255 (8 635 to 12 315)	11 (10 to 14)	4.7 (-1.6 to 18.2)	193 123 (178 063 to 208 992)	261 (243 to 280)	10.4 (6.0 to 16.7)	73 355 (50 530 to 94 828)	98 (69 to 125)	1.4 (-4.8 to 7.4)
Sao Tome and Principe	12 (10 to 14)	13 (11 to 15)	3.5 (-0.0 to 7.0)	234 (218 to 251)	313 (294 to 332)	6.0 (3.3 to 9.1)	90 (64 to 114)	120 (86 to 150)	-2.5 (-7.0 to 1.7)

Location	Incidence (95% UI)			Prevalence (95% UI)			YLDs (95% UI)		
	2016 counts	2016 age-standardised rates per 100,000	Percentage change in age-standardised rates between 1990 and 2016	2016 counts	2016 age-standardised rates per 100,000	Percentage change in age-standardised rates between 1990 and 2016	2016 counts	2016 age-standardised rates per 100,000	Percentage change in age-standardised rates between 1990 and 2016
Senegal	742 (629 to 866)	11 (9 to 13)	-3.1 (-8.5 to 1.1)	13 849 (12 858 to 14 839)	236 (221 to 253)	5.3 (1.5 to 9.9)	5 629 (3 996 to 7 127)	96 (68 to 121)	0.6 (-3.4 to 4.4)
Sierra Leone	299 (254 to 349)	10 (8 to 11)	-12.9 (-15.5 to -10.5)	8 298 (6 035 to 13 416)	329 (232 to 554)	48.7 (4.7 to 149.6)	3 585 (1 876 to 5 751)	144 (74 to 235)	52.4 (-15.8 to 135.4)
Togo	340 (288 to 399)	10 (8 to 12)	0.5 (-2.6 to 3.8)	6 166 (5 747 to 6 627)	203 (191 to 217)	-3.1 (-6.4 to 0.8)	2 477 (1 767 to 3 086)	81 (58 to 102)	-8.9 (-12.5 to -5.2)
**Eastern sub-Saharan Africa**	**20 530 (17 438 to 23 973)**	**11 (10 to 13)**	**-49.7 (-71.3 to -22.9)**	**470 147 (375 372 to 663 558)**	**336 (256 to 505)**	**17.2 (11.4 to 26.0)**	**201 878 (126 993 to 289 996)**	**144 (85 to 219)**	**9.7 (2.2 to 13.6)**
Burundi	635 (535 to 749)	12 (10 to 14)	3.1 (-1.3 to 10.1)	16 204 (11 656 to 26 353)	393 (270 to 685)	116.7 (48.0 to 277.4)	7 166 (3 724 to 11 479)	176 (89 to 293)	119.9 (16.9 to 248.9)
Comoros	40 (34 to 47)	11 (9 to 13)	-23.1 (-25.4 to -20.5)	851 (794 to 915)	262 (246 to 280)	19.5 (14.5 to 25.4)	342 (247 to 431)	105 (75 to 132)	7.1 (2.1 to 12.0)
Djibouti	55 (47 to 64)	12 (10 to 14)	-25.8 (-47.2 to -9.9)	1 169 (1 075 to 1 281)	284 (262 to 313)	2.7 (-2.3 to 12.4)	467 (331 to 599)	113 (80 to 146)	-3.2 (-10.7 to 5.3)
Eritrea	288 (244 to 336)	12 (10 to 14)	-5.0 (-8.1 to -1.7)	7 991 (5 855 to 12 640)	389 (283 to 618)	81.7 (31.1 to 191.7)	3 523 (1 937 to 5 480)	172 (92 to 265)	78.4 (4.5 to 162.4)
Ethiopia	5 417 (4 622 to 6 326)	11 (10 to 14)	-76.2 (-88.9 to -48.9)	136 024 (107 341 to 201 662)	368 (274 to 578)	10.3 (3.7 to 17.0)	58 973 (34 592 to 88 253)	161 (90 to 259)	1.3 (-2.2 to 5.9)
Kenya	2 489 (2 106 to 2 908)	12 (10 to 14)	12.3 (10.7 to 13.8)	49 356 (46 080 to 52 744)	260 (245 to 277)	19.3 (16.8 to 22.3)	18 987 (13 345 to 24 138)	99 (69 to 126)	14.4 (8.9 to 19.4)
Madagascar	1 183 (1 002 to 1 380)	10 (8 to 12)	-4.0 (-7.2 to -0.2)	21 695 (20 090 to 23 257)	215 (201 to 229)	2.8 (-0.2 to 6.5)	8 850 (6 401 to 11 116)	87 (63 to 111)	-2.3 (-6.5 to 1.7)
Malawi	770 (652 to 901)	9 (8 to 11)	-10.5 (-13.6 to -7.8)	12 431 (11 404 to 13 372)	171 (160 to 182)	-5.6 (-9.1 to -2.0)	4 964 (3 589 to 6 345)	68 (48 to 85)	-13.0 (-17.9 to -8.6)
Mozambique	1 436 (1 216 to 1 668)	11 (9 to 13)	-25.0 (-52.2 to 0.1)	34 606 (24 161 to 59 570)	396 (238 to 791)	-21.1 (-31.2 to -2.1)	15 211 (7 892 to 25 493)	182 (73 to 334)	-28.5 (-34.2 to -3.4)
Rwanda	537 (454 to 629)	10 (8 to 12)	-59.7 (-79.8 to -30.8)	41 230 (18 180 to 91 988)	1 024 (437 to 2 329)	402.9 (115.2 to 967.1)	19 015 (4 020 to 37 663)	471 (87 to 987)	426.3 (2.1 to 910.4)
Somalia	1 060 (627 to 2 034)	21 (13 to 38)	-22.4 (-29.5 to -13.0)	16 476 (10 840 to 29 236)	421 (269 to 757)	32.5 (6.5 to 69.0)	7 836 (3 547 to 13 682)	201 (87 to 339)	32.8 (-8.1 to 51.1)
South Sudan	914 (726 to 1 198)	14 (12 to 18)	-60.8 (-79.4 to -23.2)	22 698 (15 184 to 38 723)	467 (300 to 833)	35.5 (2.5 to 85.0)	10 152 (4 798 to 16 760)	212 (94 to 359)	39.0 (-14.8 to 73.7)
Tanzania	2 811 (2 396 to 3 256)	11 (9 to 13)	-2.8 (-5.7 to -0.0)	49 970 (46 384 to 53 730)	233 (218 to 247)	6.4 (3.2 to 9.9)	19 814 (14 286 to 25 108)	92 (66 to 116)	-1.1 (-4.9 to 2.6)
Uganda	1 954 (1 644 to 2 290)	10 (9 to 12)	-8.7 (-26.4 to 2.7)	44 669 (33 407 to 69 997)	364 (229 to 673)	-8.2 (-25.3 to 27.1)	19 111 (10 655 to 29 374)	161 (71 to 289)	-16.5 (-28.7 to 27.0)
Zambia	928 (783 to 1 082)	12 (10 to 14)	9.4 (4.3 to 13.5)	14 462 (13 395 to 15 578)	215 (201 to 229)	1.5 (-1.9 to 4.5)	5 907 (4 217 to 7 521)	87 (61 to 110)	-2.8 (-7.8 to 2.3)
**Central sub-Saharan Africa**	**6 199 (5 275 to 7 287)**	**11 (10 to 13)**	**-15.9 (-30.7 to -7.2)**	**129 931 (106 455 to 178 634)**	**306 (245 to 442)**	**22.0 (11.1 to 41.2)**	**55 190 (35 775 to 78 739)**	**132 (82 to 192)**	**18.2 (0.7 to 28.7)**
Angola	1 429 (1 212 to 1 657)	12 (10 to 14)	-40.2 (-67.2 to -11.0)	34 848 (26 218 to 54 045)	429 (292 to 739)	5.2 (-9.8 to 26.6)	14 969 (8 663 to 23 235)	187 (93 to 317)	-4.2 (-13.9 to 16.6)
Central African Republic	269 (218 to 336)	11 (9 to 14)	18.6 (6.2 to 47.7)	4 782 (3 747 to 7 042)	208 (166 to 297)	42.0 (13.6 to 107.4)	2 179 (1 318 to 3 242)	94 (59 to 135)	46.1 (2.8 to 95.5)
Congo	257 (218 to 303)	11 (10 to 13)	1.2 (-2.6 to 8.1)	7 011 (5 093 to 11 393)	381 (270 to 641)	101.6 (43.1 to 239.9)	2 979 (1 610 to 4 726)	163 (85 to 261)	103.3 (18.2 to 208.7)
DR Congo	4 098 (3 474 to 4 816)	11 (10 to 13)	-6.9 (-9.9 to -2.9)	80 286 (67 698 to 105 269)	275 (230 to 370)	25.9 (6.5 to 69.1)	33 890 (21 792 to 46 481)	116 (77 to 161)	25.6 (-3.8 to 60.6)
Equatorial Guinea	51 (43 to 59)	13 (11 to 15)	11.5 (6.2 to 16.5)	1 033 (967 to 1 100)	278 (261 to 295)	54.2 (49.8 to 58.8)	367 (259 to 479)	98 (69 to 128)	23.4 (10.8 to 35.6)
Gabon	96 (81 to 111)	11 (9 to 13)	-16.0 (-18.3 to -13.5)	1 972 (1 842 to 2 110)	254 (240 to 271)	2.7 (0.3 to 5.7)	749 (538 to 956)	96 (68 to 123)	-5.0 (-9.7 to -0.8)

Appendix Table 6: Incidence, prevalence, and YLDs for 2016 and percentage change of age-standardized rates by location for SCI, females									
Location	Incidence (95% UI)			Prevalence (95% UI)			YLDs (95% UI)		
	2016 counts	2016 age-standardised rates per 100,000	Percentage change in age-standardised rates between 1990 and 2016	2016 counts	2016 age-standardised rates per 100,000	Percentage change in age-standardised rates between 1990 and 2016	2016 counts	2016 age-standardised rates per 100,000	Percentage change in age-standardised rates between 1990 and 2016
**Global**	**395 232<br>(323 555 to 494 723)**	**11<br>(9 to 14)**	**-3.1<br>(-6.9 to 5.2)**	**11 873 998<br>(10 933 399 to 13 353 964)**	**321<br>(296 to 362)**	**-1.0<br>(-3.2 to 2.7)**	**4 179 510<br>(2 927 186 to 5 482 561)**	**113<br>(80 to 146)**	**-10.8<br>(-14.2 to -7.1)**
**High SDI**	**134 209<br>(102 209 to 177 230)**	**22<br>(17 to 28)**	**-0.1<br>(-4.0 to 3.6)**	**4 318 819<br>(3 987 106 to 4 653 363)**	**670<br>(615 to 728)**	**-1.4<br>(-3.4 to 0.8)**	**1 271 907<br>(889 144 to 1 659 159)**	**200<br>(138 to 263)**	**-0.7<br>(-1.3 to -0.1)**
**High-middle SDI**	**62 853<br>(51 195 to 76 594)**	**11<br>(9 to 13)**	**-9.6<br>(-15.6 to -5.7)**	**2 355 904<br>(2 170 872 to 2 678 799)**	**365<br>(336 to 414)**	**-3.3<br>(-4.9 to -1.6)**	**761 793<br>(525 062 to 1 020 325)**	**118<br>(82 to 158)**	**-14.1<br>(-17.4 to -11.0)**
**Middle SDI**	**68 289<br>(56 751 to 82 400)**	**6<br>(5 to 8)**	**6.0<br>(0.2 to 10.9)**	**2 304 592<br>(2 156 099 to 2 462 847)**	**194<br>(181 to 207)**	**22.5<br>(20.1 to 25.0)**	**749 717<br>(535 566 to 984 375)**	**63<br>(44 to 82)**	**2.0<br>(-3.3 to 6.4)**
**Low-middle SDI**	**97 255<br>(75 656 to 138 684)**	**10<br>(8 to 13)**	**13.2<br>(0.0 to 54.3)**	**2 204 968<br>(1 934 343 to 2 788 914)**	**225<br>(199 to 280)**	**23.4<br>(20.3 to 28.8)**	**964 510<br>(654 180 to 1 306 069)**	**97<br>(67 to 131)**	**14.3<br>(9.6 to 18.4)**
**Low SDI**	**34 637<br>(24 651 to 58 352)**	**9<br>(7 to 14)**	**-18.9<br>(-32.9 to -9.2)**	**743 881<br>(529 173 to 1 376 089)**	**241<br>(170 to 455)**	**18.1<br>(10.6 to 31.5)**	**431 068<br>(221 983 to 696 676)**	**137<br>(67 to 216)**	**15.6<br>(4.8 to 21.4)**
**High-income**	**140 319<br>(106 461 to 185 480)**	**22<br>(17 to 29)**	**-0.6<br>(-4.7 to 3.4)**	**4 538 266<br>(4 191 872 to 4 892 541)**	**686<br>(632 to 746)**	**-1.7<br>(-3.7 to 0.5)**	**1 324 379<br>(918 637 to 1 730 759)**	**202<br>(140 to 268)**	**-1.0<br>(-1.8 to -0.4)**
**High-income North America**	**51 321<br>(38 980 to 67 901)**	**24<br>(19 to 32)**	**9.4<br>(1.7 to 17.4)**	**1 427 599<br>(1 300 605 to 1 543 991)**	**645<br>(589 to 700)**	**-6.4<br>(-11.2 to -0.9)**	**416 238<br>(289 985 to 545 968)**	**188<br>(132 to 249)**	**-6.1<br>(-7.2 to -5.1)**
Canada	4 773<br>(3 638 to 6 294)	23<br>(17 to 29)	2.7<br>(-3.0 to 7.9)	149 322<br>(136 803 to 161 086)	665<br>(605 to 723)	2.8<br>(-2.1 to 7.7)	43 644<br>(30 531 to 57 757)	195<br>(135 to 257)	3.5<br>(2.6 to 4.4)
Greenland	7<br>(5 to 9)	30<br>(23 to 39)	-9.8<br>(-13.7 to -5.7)	166<br>(151 to 181)	675<br>(611 to 736)	2.5<br>(-2.4 to 7.8)	52<br>(36 to 67)	209<br>(146 to 275)	-7.0<br>(-12.3 to -2.2)
United States	46 523<br>(35 367 to 61 398)	24<br>(19 to 32)	10.3<br>(1.9 to 19.1)	1 277 627<br>(1 163 895 to 1 385 886)	643<br>(587 to 697)	-7.4<br>(-12.6 to -1.4)	371 284<br>(254 768 to 488 148)	188<br>(131 to 244)	-7.1<br>(-8.3 to -6.0)
**Australasia**	**3 065<br>(2 333 to 3 989)**	**20<br>(15 to 25)**	**1.1<br>(-5.2 to 6.3)**	**107 260<br>(98 755 to 116 207)**	**648<br>(593 to 706)**	**4.7<br>(0.4 to 9.8)**	**31 369<br>(21 744 to 40 675)**	**190<br>(132 to 250)**	**5.7<br>(4.6 to 6.8)**
Australia	2 578<br>(1 962 to 3 353)	20<br>(15 to 25)	2.0<br>(-4.8 to 7.5)	90 279<br>(82 907 to 98 013)	650<br>(593 to 710)	5.2<br>(0.4 to 10.6)	26 417<br>(18 436 to 34 813)	191<br>(133 to 247)	6.1<br>(5.1 to 7.3)
New Zealand	487<br>(372 to 634)	20<br>(15 to 25)	-3.0<br>(-10.0 to 2.7)	16 981<br>(15 560 to 18 477)	640<br>(583 to 700)	2.2<br>(-3.4 to 9.6)	4 950<br>(3 468 to 6 544)	188<br>(132 to 249)	3.4<br>(1.8 to 4.9)
**High-income Asia-Pacific**	**22 405<br>(17 202 to 29 026)**	**21<br>(17 to 27)**	**-3.7<br>(-6.9 to -0.5)**	**861 296<br>(790 850 to 943 217)**	**734<br>(665 to 814)**	**5.4<br>(2.7 to 8.5)**	**252 704<br>(176 237 to 330 431)**	**216<br>(148 to 286)**	**4.9<br>(3.0 to 6.7)**
Brunei	45<br>(35 to 56)	24<br>(19 to 30)	-13.5<br>(-17.9 to -9.2)	1 551<br>(1 396 to 1 722)	745<br>(672 to 824)	-3.9<br>(-8.3 to 0.6)	456<br>(317 to 609)	218<br>(152 to 287)	-12.2<br>(-15.5 to -8.9)
Japan	16 105<br>(12 430 to 20 924)	21<br>(17 to 27)	1.4<br>(-2.1 to 4.3)	626 831<br>(577 298 to 686 184)	745<br>(678 to 830)	9.8<br>(6.1 to 13.9)	183 894<br>(129 150 to 241 840)	220<br>(153 to 293)	10.3<br>(8.9 to 11.9)
Singapore	429<br>(332 to 550)	21<br>(17 to 28)	13.5<br>(9.2 to 18.1)	17 372<br>(15 869 to 19 269)	751<br>(680 to 838)	24.1<br>(16.8 to 32.5)	5 118<br>(3 582 to 6 782)	222<br>(153 to 291)	19.3<br>(15.7 to 22.6)
South Korea	5 827<br>(4 483 to 7 501)	22<br>(17 to 28)	-13.9<br>(-18.7 to -8.4)	215 542<br>(195 513 to 237 282)	707<br>(637 to 787)	-5.5<br>(-10.3 to -0.4)	63 422<br>(43 901 to 83 705)	209<br>(146 to 274)	-15.7<br>(-19.4 to -12.5)
**Western Europe**	**58 054<br>(43 036 to 77 770)**	**23<br>(17 to 30)**	**-5.0<br>(-9.8 to -1.0)**	**1 977 398<br>(1 816 260 to 2 160 612)**	**742<br>(680 to 819)**	**-0.3<br>(-2.7 to 1.9)**	**577 935<br>(402 923 to 757 024)**	**218<br>(154 to 286)**	**0.7<br>(-0.3 to 1.8)**
Andorra	10<br>(8 to 14)	23<br>(17 to 29)	5.9<br>(2.4 to 9.5)	382<br>(350 to 418)	771<br>(700 to 854)	8.2<br>(2.5 to 14.2)	112<br>(78 to 148)	226<br>(155 to 298)	8.3<br>(7.9 to 8.7)
Austria	1 243<br>(917 to 1 680)	24<br>(18 to 32)	-10.3<br>(-16.1 to -4.7)	43 118<br>(39 044 to 47 751)	789<br>(711 to 889)	-1.8<br>(-6.7 to 4.4)	12 569<br>(8 784 to 16 719)	231<br>(159 to 303)	-0.8<br>(-2.4 to 0.7)
Belgium	1 988<br>(1 453 to 2 758)	27<br>(21 to 37)	5.4<br>(-3.8 to 14.1)	56 868<br>(51 505 to 62 313)	804<br>(724 to 893)	1.1<br>(-3.9 to 6.2)	16 564<br>(11 467 to 21 787)	235<br>(162 to 311)	2.2<br>(1.0 to 3.6)
Cyprus	107<br>(81 to 139)	22<br>(17 to 29)	-4.1<br>(-9.8 to 1.5)	4 108<br>(3 704 to 4 545)	769<br>(689 to 856)	3.4<br>(-2.4 to 8.1)	1 201<br>(836 to 1 592)	225<br>(155 to 300)	-1.5<br>(-4.4 to 1.1)
Denmark	787<br>(579 to 1 060)	24<br>(18 to 31)	-13.7<br>(-19.4 to -7.9)	26 117<br>(23 689 to 29 059)	766<br>(687 to 860)	2.2<br>(-4.2 to 8.5)	7 600<br>(5 307 to 10 013)	224<br>(155 to 300)	3.2<br>(1.2 to 5.0)
Finland	972<br>(706 to 1 336)	28<br>(21 to 37)	3.0<br>(-3.5 to 9.1)	29 584<br>(26 788 to 32 501)	844<br>(761 to 940)	8.0<br>(2.2 to 13.2)	8 627<br>(6 061 to 11 451)	247<br>(170 to 325)	8.9<br>(7.5 to 10.2)
France	10 306<br>(7 522 to 14 043)	24<br>(18 to 31)	-12.7<br>(-19.1 to -6.4)	305 686<br>(278 558 to 333 591)	748<br>(677 to 826)	-8.4<br>(-12.9 to -3.5)	89 323<br>(63 209 to 117 706)	219<br>(153 to 290)	-7.5<br>(-8.9 to -6.2)
Germany	11 308<br>(8 297 to 15 222)	23<br>(17 to 29)	-2.7<br>(-9.1 to 2.9)	389 795<br>(354 715 to 428 685)	738<br>(669 to 820)	2.8<br>(-2.5 to 8.2)	113 326<br>(79 826 to 148 233)	216<br>(148 to 287)	3.7<br>(2.1 to 5.4)
Greece	1 174<br>(881 to 1 544)	21<br>(16 to 27)	-14.5<br>(-19.0 to -9.7)	50 380<br>(46 176 to 55 128)	736<br>(668 to 816)	-7.1<br>(-12.2 to -2.3)	14 629<br>(10 125 to 19 182)	215<br>(149 to 284)	-6.5<br>(-7.3 to -5.8)
Iceland	37<br>(28 to 49)	22<br>(16 to 28)	-0.1<br>(-5.7 to 5.3)	1 363<br>(1 236 to 1 502)	744<br>(672 to 824)	8.1<br>(2.2 to 14.9)	398<br>(279 to 533)	218<br>(154 to 285)	9.0<br>(7.9 to 10.1)
Ireland	574<br>(436 to 756)	24<br>(18 to 31)	5.5<br>(-0.6 to 11.5)	21 635<br>(19 548 to 23 876)	841<br>(754 to 936)	12.2<br>(5.5 to 19.9)	6 340<br>(4 442 to 8 427)	247<br>(170 to 327)	13.6<br>(12.1 to 15.0)
Israel	887<br>(675 to 1 157)	20<br>(16 to 26)	-1.5<br>(-11.6 to 5.3)	32 570<br>(27 791 to 42 549)	785<br>(668 to 1 037)	17.7<br>(8.3 to 33.1)	10 119<br>(6 650 to 15 045)	242<br>(163 to 348)	17.7<br>(6.5 to 29.6)
Italy	8 588<br>(6 415 to 11 443)	23<br>(17 to 30)	-4.3<br>(-9.2 to 1.3)	302 654<br>(273 776 to 332 908)	766<br>(682 to 855)	0.4<br>(-4.9 to 6.2)	88 118<br>(60 196 to 114 351)	224<br>(154 to 295)	1.1<br>(-0.2 to 2.3)
Luxembourg	75<br>(56 to 99)	23<br>(17 to 30)	-16.9<br>(-22.1 to -11.9)	2 478<br>(2 262 to 2 721)	732<br>(664 to 809)	-12.4<br>(-16.5 to -8.1)	724<br>(503 to 961)	214<br>(148 to 285)	-11.6<br>(-12.7 to -10.6)
Malta	51<br>(38 to 68)	23<br>(17 to 29)	-8.3<br>(-12.0 to -4.4)	1 950<br>(1 777 to 2 145)	767<br>(691 to 854)	-1.3<br>(-5.7 to 3.9)	569<br>(394 to 762)	225<br>(156 to 298)	-1.3<br>(-2.7 to -0.7)
Netherlands	2 096<br>(1 566 to 2 758)	22<br>(17 to 28)	9.9<br>(3.3 to 16.9)	73 507<br>(67 477 to 79 881)	716<br>(654 to 786)	14.9<br>(8.8 to 23.0)	21 372<br>(15 028 to 28 085)	209<br>(146 to 276)	15.6<br>(14.6 to 16.6)
Norway	734<br>(541 to 992)	24<br>(18 to 31)	4.5<br>(-1.3 to 10.4)	23 057<br>(20 936 to 25 320)	760<br>(684 to 842)	11.7<br>(6.0 to 18.6)	6 748<br>(4 735 to 8 927)	223<br>(155 to 295)	12.6<br>(11.7 to 13.5)
Portugal	1 194<br>(903 to 1 565)	19<br>(15 to 25)	-13.7<br>(-20.1 to -7.8)	44 811<br>(41 186 to 48 557)	646<br>(590 to 705)	-9.7<br>(-15.5 to -4.4)	13 019<br>(9 087 to 17 192)	189<br>(132 to 251)	-14.6<br>(-16.7 to -12.7)
Spain	5 528<br>(4 121 to 7 359)	21<br>(16 to 27)	-0.9<br>(-8.6 to 5.8)	213 093<br>(195 245 to 232 531)	733<br>(666 to 810)	2.2<br>(-3.3 to 8.1)	62 164<br>(43 291 to 81 005)	215<br>(148 to 285)	3.3<br>(2.0 to 4.6)
Sweden	1 262<br>(935 to 1 692)	22<br>(17 to 28)	0.4<br>(-5.7 to 5.6)	42 577<br>(38 753 to 46 908)	732<br>(659 to 816)	0.5<br>(-4.9 to 6.7)	12 434<br>(8 648 to 16 489)	216<br>(150 to 286)	1.2<br>(0.6 to 1.9)
Switzerland	1 165<br>(847 to 1 583)	22<br>(16 to 29)	-27.2<br>(-34.8 to -20.2)	33 930<br>(31 059 to 36 649)	634<br>(581 to 690)	-24.1<br>(-30.4 to -18.1)	9 944<br>(6 935 to 13 056)	187<br>(132 to 249)	-23.4<br>(-24.2 to -22.6)
United Kingdom	7 913<br>(5 926 to 10 623)	22<br>(16 to 28)	-0.5<br>(-4.7 to 3.0)	275 821<br>(252 605 to 303 554)	720<br>(655 to 799)	0.1<br>(-3.1 to 2.9)	80 389<br>(55 434 to 105 098)	211<br>(148 to 278)	0.6<br>(-0.4 to 1.8)
**Southern Latin America**	**5 474<br>(4 242 to 7 050)**	**16<br>(12 to 20)**	**11.1<br>(7.3 to 15.4)**	**164 714<br>(152 325 to 178 937)**	**466<br>(428 to 508)**	**18.1<br>(13.5 to 23.3)**	**50 397<br>(35 282 to 66 287)**	**143<br>(100 to 187)**	**5.0<br>(1.4 to 8.0)**
Argentina	3 658<br>(2 852 to 4 678)	16<br>(12 to 20)	11.9<br>(6.6 to 17.4)	110 547<br>(101 634 to 120 560)	477<br>(437 to 522)	19.8<br>(13.9 to 26.7)	34 405<br>(24 106 to 44 651)	149<br>(104 to 193)	7.9<br>(4.2 to 11.1)
Chile	1 497<br>(1 147 to 1 958)	15<br>(12 to 20)	7.7<br>(2.9 to 12.9)	45 047<br>(41 581 to 48 904)	444<br>(407 to 485)	13.6<br>(8.9 to 19.0)	13 283<br>(9 222 to 17 551)	131<br>(91 to 171)	-1.1<br>(-4.8 to 2.4)
Uruguay	319<br>(246 to 415)	16<br>(12 to 20)	14.3<br>(9.7 to 18.7)	9 111<br>(8 406 to 9 950)	455<br>(417 to 501)	19.5<br>(13.9 to 26.3)	2 760<br>(1 924 to 3 633)	138<br>(95 to 178)	8.3<br>(4.4 to 11.4)
**Central Europe, Eastern Europe, and Central Asia**	**30 581<br>(24 410 to 37 809)**	**14<br>(11 to 17)**	**-2.1<br>(-4.5 to 0.3)**	**1 073 876<br>(995 842 to 1 151 614)**	**431<br>(399 to 464)**	**5.5<br>(2.7 to 10.2)**	**324 240<br>(226 471 to 430 508)**	**131<br>(91 to 171)**	**-5.1<br>(-9.9 to -0.3)**
**Eastern Europe**	**16 202<br>(13 112 to 20 082)**	**14<br>(12 to 17)**	**-0.2<br>(-3.3 to 3.0)**	**588 578<br>(547 359 to 628 487)**	**444<br>(411 to 477)**	**4.0<br>(0.6 to 8.4)**	**172 709<br>(120 779 to 227 407)**	**132<br>(92 to 172)**	**-3.9<br>(-8.1 to 0.7)**
Belarus	826<br>(656 to 1 035)	16<br>(13 to 19)	16.6<br>(12.4 to 21.5)	29 827<br>(27 551 to 32 119)	489<br>(451 to 531)	16.1<br>(10.7 to 22.2)	8 695<br>(6 080 to 11 483)	144<br>(99 to 189)	7.1<br>(4.3 to 9.4)
Estonia	99<br>(79 to 124)	14<br>(11 to 17)	-14.9<br>(-20.3 to -9.7)	3 989<br>(3 671 to 4 320)	473<br>(431 to 517)	-2.7<br>(-8.6 to 2.7)	1 164<br>(817 to 1 510)	139<br>(95 to 183)	-7.4<br>(-9.5 to -5.6)
Latvia	157<br>(125 to 196)	14<br>(11 to 17)	-18.0<br>(-23.0 to -13.1)	5 855<br>(5 417 to 6 265)	449<br>(414 to 483)	-5.8<br>(-10.0 to -1.0)	1 704<br>(1 171 to 2 238)	132<br>(90 to 174)	-11.3<br>(-13.2 to -9.6)
Lithuania	264<br>(209 to 328)	16<br>(13 to 19)	-2.4<br>(-7.5 to 2.6)	9 494<br>(8 749 to 10 207)	492<br>(451 to 535)	3.3<br>(-2.2 to 8.6)	2 755<br>(1 936 to 3 586)	144<br>(100 to 190)	-1.1<br>(-3.1 to 0.7)
Moldova	252<br>(204 to 306)	12<br>(10 to 15)	-14.2<br>(-18.7 to -9.2)	9 705<br>(8 880 to 10 590)	404<br>(369 to 440)	0.0<br>(-5.3 to 7.0)	3 038<br>(2 110 to 3 977)	127<br>(88 to 167)	-6.6<br>(-11.4 to -1.2)
Russia	11 550<br>(9 318 to 14 250)	15<br>(12 to 18)	0.5<br>(-3.4 to 4.5)	409 601<br>(380 552 to 438 701)	450<br>(416 to 484)	4.3<br>(-0.1 to 9.5)	120 666<br>(83 661 to 157 865)	133<br>(93 to 176)	-3.8<br>(-8.6 to 1.8)
Ukraine	3 054<br>(2 474 to 3 764)	13<br>(10 to 16)	-3.6<br>(-7.8 to 0.5)	120 107<br>(110 730 to 129 191)	418<br>(383 to 452)	1.3<br>(-2.9 to 6.3)	35 426<br>(24 549 to 46 304)	124<br>(86 to 164)	-5.1<br>(-8.9 to -0.0)
**Central Europe**	**10 040<br>(7 707 to 12 900)**	**15<br>(12 to 19)**	**-2.6<br>(-6.2 to 0.8)**	**341 394<br>(314 349 to 372 434)**	**474<br>(435 to 522)**	**12.7<br>(8.8 to 18.9)**	**100 920<br>(70 038 to 132 157)**	**141<br>(98 to 186)**	**0.2<br>(-5.1 to 5.8)**
Albania	179<br>(140 to 226)	12<br>(10 to 15)	19.0<br>(13.3 to 24.5)	6 917<br>(6 194 to 7 986)	426<br>(380 to 494)	27.8<br>(17.2 to 48.5)	2 095<br>(1 445 to 2 806)	129<br>(90 to 173)	12.4<br>(1.1 to 27.0)
Bosnia and Herzegovina	244<br>(187 to 312)	13<br>(10 to 16)	28.3<br>(23.5 to 32.8)	12 613<br>(9 818 to 19 198)	561<br>(429 to 871)	77.3<br>(38.8 to 176.5)	4 218<br>(2 411 to 6 789)	189<br>(106 to 299)	70.7<br>(12.7 to 147.6)
Bulgaria	529<br>(412 to 673)	14<br>(11 to 17)	3.3<br>(-1.5 to 7.8)	20 069<br>(18 374 to 21 738)	442<br>(402 to 485)	7.3<br>(1.8 to 12.6)	5 861<br>(4 093 to 7 758)	130<br>(89 to 172)	-0.7<br>(-3.0 to 1.4)
Croatia	394<br>(300 to 513)	14<br>(11 to 17)	-6.5<br>(-13.8 to 0.4)	11 220<br>(9 824 to 14 162)	408<br>(352 to 534)	3.8<br>(-10.4 to 36.2)	3 473<br>(2 259 to 4 832)	127<br>(81 to 181)	9.1<br>(-11.5 to 39.8)
Czech Republic	1 160<br>(886 to 1 497)	19<br>(15 to 24)	-8.5<br>(-15.4 to -1.8)	38 698<br>(35 329 to 42 119)	586<br>(532 to 643)	16.8<br>(9.8 to 24.6)	11 381<br>(7 919 to 14 989)	173<br>(120 to 227)	15.1<br>(13.2 to 16.9)
Hungary	1 047<br>(794 to 1 372)	17<br>(13 to 21)	-18.7<br>(-24.9 to -13.1)	29 489<br>(26 893 to 32 131)	459<br>(414 to 504)	2.0<br>(-2.9 to 7.6)	8 610<br>(5 963 to 11 392)	135<br>(97 to 179)	-4.3<br>(-7.0 to -2.1)
Macedonia	141<br>(108 to 179)	13<br>(10 to 17)	20.0<br>(14.2 to 25.5)	4 863<br>(4 392 to 5 336)	407<br>(367 to 449)	21.1<br>(11.8 to 29.5)	1 437<br>(985 to 1 898)	120<br>(82 to 159)	8.5<br>(4.4 to 12.4)
Montenegro	44<br>(34 to 55)	14<br>(11 to 17)	13.8<br>(9.7 to 18.0)	1 577<br>(1 435 to 1 713)	437<br>(396 to 478)	14.3<br>(7.8 to 22.0)	462<br>(323 to 605)	129<br>(89 to 170)	9.2<br>(6.4 to 11.8)
Poland	3 438<br>(2 621 to 4 446)	15<br>(12 to 19)	1.8<br>(-3.3 to 6.8)	113 275<br>(103 513 to 123 481)	467<br>(424 to 514)	15.7<br>(9.2 to 23.2)	33 239<br>(23 173 to 44 000)	138<br>(96 to 183)	4.0<br>(0.7 to 6.7)
Romania	1 503<br>(1 162 to 1 945)	14<br>(11 to 18)	-5.6<br>(-11.2 to -0.2)	54 607<br>(49 862 to 59 377)	454<br>(414 to 498)	-0.0<br>(-5.2 to 5.3)	16 004<br>(11 058 to 21 161)	134<br>(93 to 177)	-11.7<br>(-14.7 to -9.2)
Serbia	630<br>(484 to 807)	13<br>(10 to 17)	16.0<br>(11.5 to 20.6)	24 890<br>(21 531 to 31 010)	477<br>(407 to 612)	35.2<br>(18.1 to 76.7)	7 618<br>(4 945 to 10 563)	147<br>(98 to 208)	30.5<br>(7.1 to 63.2)

Location	Incidence (95% UI)			Prevalence (95% UI)			YLDs (95% UI)		
	2016 counts	2016 age-standardised rates per 100,000	Percentage change in age-standardised rates between 1990 and 2016	2016 counts	2016 age-standardised rates per 100,000	Percentage change in age-standardised rates between 1990 and 2016	2016 counts	2016 age-standardised rates per 100,000	Percentage change in age-standardised rates between 1990 and 2016
Slovakia	453 (345 to 583)	15 (12 to 19)	-10.8 (-16.1 to -5.5)	15 225 (13 945 to 16 511)	455 (413 to 496)	3.7 (-1.4 to 9.1)	4 467 (3 140 to 5 886)	134 (93 to 177)	-1.7 (-4.0 to 0.0)
Slovenia	278 (203 to 374)	21 (16 to 27)	3.9 (-4.9 to 15.6)	7 951 (7 281 to 8 573)	592 (543 to 641)	13.8 (8.0 to 20.1)	2 339 (1 620 to 3 076)	175 (122 to 233)	13.6 (11.7 to 15.1)
**Central Asia**	**4 339 (3 540 to 5 224)**	**10 (8 to 12)**	**2.4 (-0.2 to 4.9)**	**143 904 (131 355 to 157 976)**	**325 (297 to 356)**	**7.9 (3.2 to 15.7)**	**48 027 (33 672 to 62 883)**	**108 (75 to 140)**	**-0.0 (-5.5 to 6.4)**
Armenia	155 (124 to 189)	10 (8 to 12)	-7.4 (-11.9 to -2.8)	8 785 (6 431 to 12 806)	476 (351 to 687)	-17.2 (-26.6 to -4.4)	2 684 (1 688 to 3 908)	145 (92 to 210)	-26.0 (-33.2 to -13.2)
Azerbaijan	470 (380 to 568)	10 (8 to 12)	5.2 (0.9 to 9.3)	17 700 (15 831 to 20 656)	339 (304 to 392)	20.8 (11.6 to 38.8)	5 757 (3 935 to 7 731)	110 (77 to 147)	10.4 (-1.6 to 25.9)
Georgia	196 (157 to 238)	9 (7 to 11)	-4.4 (-8.1 to -0.6)	7 440 (6 643 to 8 747)	315 (279 to 372)	5.6 (-3.0 to 26.6)	2 385 (1 582 to 3 273)	102 (70 to 136)	4.2 (-8.9 to 21.8)
Kazakhstan	1 044 (850 to 1 262)	11 (9 to 14)	8.7 (4.3 to 13.2)	32 849 (30 464 to 35 470)	352 (326 to 380)	10.6 (5.8 to 16.1)	10 093 (7 032 to 13 199)	108 (77 to 141)	-0.3 (-5.2 to 4.0)
Kyrgyzstan	282 (231 to 341)	9 (7 to 11)	-8.7 (-12.8 to -4.7)	8 377 (7 646 to 9 145)	296 (271 to 322)	-0.7 (-6.1 to 4.4)	2 771 (1 907 to 3 577)	98 (68 to 127)	-6.7 (-10.3 to -3.2)
Mongolia	167 (136 to 201)	11 (9 to 13)	19.9 (3.9 to 30.9)	4 733 (4 331 to 5 166)	320 (293 to 348)	34.3 (27.7 to 41.7)	1 639 (1 166 to 2 127)	110 (78 to 144)	20.0 (14.6 to 25.1)
Tajikistan	362 (293 to 438)	8 (7 to 10)	-4.5 (-8.4 to -0.7)	12 311 (10 029 to 18 033)	322 (266 to 458)	28.8 (9.0 to 83.8)	4 576 (2 880 to 6 791)	119 (75 to 170)	26.9 (-2.8 to 69.3)
Turkmenistan	253 (207 to 302)	9 (7 to 11)	3.6 (-0.4 to 7.7)	7 715 (7 047 to 8 429)	286 (263 to 313)	11.7 (7.5 to 17.0)	2 509 (1 745 to 3 259)	93 (65 to 121)	-0.5 (-4.5 to 2.8)
Uzbekistan	1 412 (1 151 to 1 692)	9 (7 to 11)	5.1 (1.5 to 9.0)	43 996 (40 361 to 48 182)	292 (269 to 319)	10.6 (6.8 to 14.6)	14 218 (9 997 to 18 353)	94 (66 to 123)	0.9 (-3.1 to 4.3)
**Latin America and Caribbean**	**14 969 (12 043 to 18 177)**	**5 (4 to 7)**	**-6.2 (-12.0 to -1.8)**	**439 320 (398 488 to 486 581)**	**153 (139 to 169)**	**-5.4 (-8.2 to -1.3)**	**144 500 (101 699 to 186 357)**	**50 (36 to 66)**	**-17.8 (-21.3 to -14.7)**
**Central Latin America**	**5 735 (4 600 to 7 035)**	**5 (4 to 6)**	**-10.0 (-17.4 to -5.4)**	**173 485 (153 641 to 198 174)**	**140 (125 to 159)**	**-1.6 (-4.9 to 1.8)**	**56 728 (39 258 to 75 383)**	**46 (31 to 60)**	**-15.1 (-18.6 to -11.9)**
Colombia	1 069 (861 to 1 316)	5 (4 to 6)	-7.4 (-17.3 to -0.4)	36 343 (32 269 to 41 084)	146 (129 to 165)	6.8 (0.4 to 15.5)	11 504 (7 999 to 15 222)	46 (32 to 61)	-6.6 (-13.4 to 1.0)
Costa Rica	120 (95 to 149)	5 (4 to 6)	14.5 (9.0 to 19.8)	3 558 (3 179 to 3 960)	142 (127 to 158)	24.8 (19.6 to 30.3)	1 062 (722 to 1 392)	42 (29 to 57)	12.9 (8.8 to 16.3)
El Salvador	156 (124 to 194)	5 (4 to 6)	-19.2 (-54.7 to 11.1)	7 006 (4 693 to 13 054)	218 (145 to 407)	-5.3 (-19.8 to 15.7)	2 584 (1 197 to 4 570)	80 (36 to 148)	-20.3 (-29.9 to 6.6)
Guatemala	355 (285 to 437)	5 (4 to 6)	-2.5 (-38.8 to 19.3)	10 601 (8 459 to 15 158)	147 (117 to 213)	11.3 (-7.0 to 27.4)	3 943 (2 503 to 5 931)	55 (34 to 83)	-4.5 (-16.4 to 14.8)
Honduras	176 (140 to 217)	5 (4 to 6)	50.5 (40.2 to 62.9)	5 216 (4 342 to 6 546)	139 (118 to 172)	68.1 (46.3 to 106.1)	1 906 (1 311 to 2 564)	51 (35 to 69)	50.3 (27.2 to 73.8)
Mexico	2 889 (2 311 to 3 567)	5 (4 to 6)	-16.6 (-20.5 to -12.6)	78 935 (71 237 to 86 680)	128 (116 to 139)	-12.1 (-15.5 to -7.7)	25 067 (17 607 to 32 687)	40 (29 to 53)	-23.8 (-27.3 to -20.6)
Nicaragua	129 (103 to 160)	5 (4 to 6)	6.1 (-2.8 to 13.4)	5 986 (3 944 to 11 304)	201 (137 to 371)	-7.3 (-21.0 to 11.6)	2 236 (1 017 to 4 135)	74 (36 to 133)	-18.6 (-27.0 to 3.7)
Panama	88 (70 to 110)	5 (4 to 6)	8.4 (3.8 to 13.4)	2 692 (2 433 to 2 976)	139 (125 to 153)	16.4 (11.6 to 21.1)	843 (584 to 1 104)	43 (30 to 57)	3.3 (-1.6 to 8.1)
Venezuela	754 (606 to 929)	5 (4 to 6)	7.8 (2.5 to 12.6)	23 148 (20 673 to 25 898)	152 (136 to 169)	21.6 (15.3 to 30.8)	7 208 (4 973 to 9 432)	47 (33 to 62)	7.9 (1.2 to 14.8)
**Andean Latin America**	**1 735 (1 420 to 2 069)**	**6 (5 to 7)**	**-3.3 (-16.0 to 6.7)**	**50 338 (44 882 to 56 445)**	**177 (159 to 198)**	**8.1 (4.1 to 13.2)**	**16 770 (11 957 to 21 651)**	**59 (41 to 76)**	**-8.7 (-13.6 to -4.4)**
Bolivia	303 (247 to 365)	6 (5 to 7)	-10.2 (-14.3 to -6.0)	8 028 (7 122 to 8 949)	157 (140 to 174)	6.1 (2.8 to 10.3)	2 864 (2 041 to 3 677)	56 (39 to 72)	-8.0 (-13.1 to -3.5)
Ecuador	531 (412 to 686)	7 (5 to 9)	32.1 (11.9 to 74.0)	12 987 (11 799 to 14 384)	166 (152 to 184)	13.8 (8.8 to 18.3)	4 208 (2 957 to 5 477)	54 (38 to 70)	-4.3 (-9.2 to 0.1)
Peru	901 (739 to 1 087)	6 (5 to 7)	-14.9 (-31.6 to -3.8)	29 323 (25 805 to 33 448)	190 (168 to 215)	6.6 (1.4 to 13.7)	9 424 (6 527 to 12 455)	61 (41 to 81)	-10.5 (-16.3 to -5.1)
**Caribbean**	**1 359 (1 094 to 1 650)**	**6 (5 to 7)**	**23.7 (15.8 to 35.6)**	**43 828 (34 743 to 60 555)**	**188 (149 to 261)**	**55.3 (24.4 to 114.2)**	**15 193 (10 019 to 20 904)**	**65 (44 to 90)**	**40.4 (10.4 to 73.9)**
Antigua and Barbuda	2 (2 to 3)	5 (4 to 6)	11.8 (8.1 to 16.0)	78 (72 to 86)	160 (146 to 175)	13.2 (9.5 to 17.8)	24 (17 to 31)	49 (34 to 64)	2.7 (-2.0 to 7.0)
The Bahamas	11 (9 to 13)	5 (4 to 7)	7.8 (3.9 to 11.9)	323 (295 to 353)	154 (140 to 168)	13.4 (9.4 to 19.2)	101 (71 to 134)	48 (34 to 62)	5.0 (0.7 to 8.8)
Barbados	8 (6 to 10)	5 (4 to 6)	15.7 (12.2 to 19.7)	246 (225 to 269)	147 (134 to 162)	14.8 (11.0 to 21.7)	74 (51 to 96)	44 (31 to 59)	4.8 (0.2 to 8.9)
Belize	9 (7 to 10)	5 (4 to 6)	9.5 (-11.8 to 20.7)	239 (214 to 265)	143 (129 to 157)	19.7 (14.8 to 28.4)	82 (57 to 106)	49 (35 to 63)	12.8 (7.4 to 18.2)
Bermuda	2 (1 to 2)	5 (4 to 6)	9.6 (6.1 to 13.6)	58 (52 to 63)	158 (144 to 173)	25.2 (21.6 to 29.1)	17 (12 to 22)	46 (32 to 61)	14.8 (10.9 to 17.8)
Cuba	454 (353 to 583)	7 (5 to 8)	21.5 (13.8 to 29.8)	11 211 (10 149 to 12 249)	167 (151 to 183)	20.6 (15.5 to 28.3)	3 276 (2 270 to 4 289)	49 (34 to 64)	11.1 (7.5 to 14.4)
Dominica	2 (1 to 2)	4 (4 to 5)	20.9 (17.5 to 24.7)	54 (49 to 59)	141 (127 to 155)	29.6 (22.9 to 39.8)	17 (12 to 23)	46 (32 to 59)	20.9 (15.3 to 26.8)
Dominican Republic	246 (200 to 298)	5 (4 to 6)	22.2 (18.0 to 26.6)	7 485 (6 803 to 8 238)	148 (135 to 163)	33.9 (28.2 to 43.8)	2 452 (1 710 to 3 176)	48 (34 to 63)	14.2 (8.0 to 19.4)
Grenada	2 (2 to 3)	5 (4 to 6)	21.5 (17.4 to 25.6)	76 (68 to 84)	146 (131 to 160)	24.2 (20.0 to 32.1)	25 (18 to 33)	48 (34 to 63)	14.4 (9.7 to 19.4)
Guyana	18 (14 to 21)	5 (4 to 6)	3.9 (-0.3 to 8.0)	474 (432 to 514)	127 (116 to 137)	13.1 (9.9 to 16.8)	168 (118 to 219)	45 (32 to 58)	4.8 (1.1 to 8.3)
Haiti	311 (231 to 430)	6 (4 to 8)	33.0 (4.7 to 86.9)	14 442 (6 292 to 29 914)	283 (123 to 587)	221.1 (39.4 to 567.5)	5 957 (2 497 to 10 733)	118 (45 to 207)	207.9 (27.8 to 412.4)
Jamaica	63 (52 to 77)	4 (4 to 5)	20.5 (16.6 to 24.4)	1 902 (1 725 to 2 072)	133 (120 to 145)	23.4 (19.1 to 30.2)	619 (427 to 808)	43 (30 to 57)	14.5 (8.4 to 20.1)
Puerto Rico	121 (96 to 151)	6 (5 to 7)	24.8 (19.2 to 31.0)	3 652 (3 350 to 3 953)	166 (152 to 181)	23.7 (19.2 to 28.2)	1 067 (742 to 1 385)	49 (34 to 65)	16.6 (13.7 to 19.0)
Saint Lucia	4 (3 to 5)	5 (4 to 6)	15.0 (11.3 to 19.1)	142 (129 to 155)	146 (133 to 160)	22.7 (18.4 to 29.0)	46 (32 to 59)	47 (33 to 61)	10.6 (6.3 to 14.4)
Saint Vincent and the Grenadines	2 (2 to 3)	5 (4 to 6)	18.8 (15.0 to 23.1)	77 (70 to 85)	141 (128 to 154)	24.0 (19.2 to 30.8)	26 (18 to 33)	47 (33 to 61)	17.2 (13.4 to 21.0)
Suriname	14 (11 to 17)	5 (4 to 6)	13.3 (9.1 to 17.1)	408 (372 to 450)	151 (137 to 166)	17.2 (11.4 to 22.6)	141 (99 to 185)	52 (37 to 68)	6.7 (1.2 to 11.8)
Trinidad and Tobago	31 (25 to 38)	5 (4 to 6)	5.7 (-13.1 to 16.0)	1 077 (977 to 1 175)	148 (134 to 161)	29.3 (24.6 to 38.2)	342 (244 to 448)	47 (33 to 61)	17.8 (13.1 to 21.8)
Virgin Islands, U.S.	3 (3 to 4)	5 (4 to 6)	11.6 (8.0 to 15.6)	102 (93 to 111)	159 (145 to 174)	17.0 (13.1 to 21.5)	30 (21 to 39)	47 (33 to 62)	2.9 (-1.8 to 7.6)
**Tropical Latin America**	**6 140 (4 855 to 7 646)**	**6 (5 to 7)**	**-9.6 (-15.6 to -3.3)**	**171 669 (157 634 to 187 040)**	**153 (140 to 166)**	**-19.4 (-22.2 to -17.0)**	**55 493 (38 471 to 72 284)**	**49 (34 to 64)**	**-28.6 (-30.8 to -26.6)**
Brazil	5 988 (4 727 to 7 443)	6 (5 to 7)	-10.3 (-16.3 to -4.0)	167 305 (153 766 to 182 285)	153 (140 to 166)	-20.1 (-22.9 to -17.6)	53 830 (37 791 to 69 993)	49 (35 to 65)	-29.2 (-31.6 to -27.1)
Paraguay	152 (120 to 190)	5 (4 to 6)	27.6 (21.9 to 33.9)	4 364 (3 904 to 4 905)	142 (127 to 158)	19.0 (13.5 to 23.4)	1 486 (1 045 to 1 926)	48 (34 to 62)	8.3 (4.0 to 11.8)
**Southeast Asia, East Asia, and Oceania**	**58 477 (48 293 to 72 186)**	**6 (5 to 7)**	**9.9 (2.5 to 16.7)**	**2 244 984 (2 106 614 to 2 417 740)**	**200 (188 to 215)**	**28.9 (25.3 to 32.8)**	**734 986 (514 607 to 943 560)**	**66 (46 to 85)**	**8.4 (3.7 to 12.7)**
**East Asia**	**40 186 (33 113 to 49 336)**	**6 (5 to 7)**	**12.7 (6.3 to 20.0)**	**1 608 891 (1 517 846 to 1 707 666)**	**203 (191 to 217)**	**27.2 (23.5 to 30.5)**	**473 195 (333 129 to 618 329)**	**60 (42 to 79)**	**0.8 (-5.0 to 5.8)**
China	38 781 (31 866 to 47 665)	6 (5 to 7)	12.1 (5.5 to 19.4)	1 558 476 (1 470 653 to 1 653 747)	204 (192 to 218)	27.1 (23.2 to 30.4)	458 750 (318 866 to 599 366)	60 (42 to 78)	0.6 (-5.3 to 5.7)
North Korea	722 (594 to 876)	5 (4 to 6)	43.3 (32.5 to 60.3)	21 574 (20 127 to 23 014)	153 (143 to 164)	31.6 (27.7 to 35.8)	7 470 (5 322 to 9 627)	53 (37 to 68)	28.3 (23.4 to 33.4)
Taiwan (Province of China)	684 (561 to 834)	6 (5 to 7)	24.2 (17.8 to 31.8)	28 841 (27 068 to 30 769)	206 (192 to 221)	32.2 (27.8 to 37.3)	8 449 (5 833 to 11 063)	60 (42 to 79)	18.4 (13.9 to 22.3)
**Southeast Asia**	**17 982 (15 046 to 21 793)**	**6 (5 to 7)**	**3.6 (-12.6 to 13.2)**	**628 807 (559 418 to 750 021)**	**192 (171 to 228)**	**34.0 (25.2 to 43.5)**	**233 400 (159 999 to 311 623)**	**71 (50 to 96)**	**19.4 (12.7 to 26.8)**
Cambodia	422 (353 to 507)	5 (5 to 7)	-9.4 (-47.1 to 18.6)	21 447 (12 746 to 46 616)	295 (170 to 649)	-6.9 (-24.5 to 31.5)	9 224 (3 721 to 17 759)	129 (50 to 246)	-18.0 (-29.3 to 39.7)
Indonesia	6 701 (5 570 to 8 160)	6 (5 to 7)	5.2 (-0.0 to 10.2)	222 507 (201 912 to 250 350)	176 (160 to 197)	27.6 (18.2 to 38.3)	83 121 (59 393 to 107 431)	66 (46 to 84)	15.6 (7.5 to 24.3)
Laos	182 (152 to 220)	5 (4 to 6)	-19.2 (-57.0 to 15.4)	4 440 (4 093 to 4 827)	142 (132 to 153)	54.7 (48.4 to 59.5)	1 731 (1 223 to 2 203)	55 (40 to 70)	38.6 (32.3 to 44.9)
Malaysia	876 (718 to 1 091)	6 (5 to 7)	20.0 (14.6 to 26.6)	29 895 (27 809 to 32 228)	194 (181 to 208)	30.7 (27.3 to 33.9)	9 295 (6 621 to 11 973)	60 (43 to 79)	10.0 (4.9 to 14.4)
Maldives	8 (6 to 9)	4 (4 to 5)	-5.7 (-10.1 to -1.4)	265 (243 to 286)	154 (142 to 165)	38.3 (32.9 to 44.1)	81 (57 to 107)	47 (33 to 61)	10.3 (2.6 to 17.4)
Mauritius	31 (26 to 38)	5 (4 to 6)	25.8 (19.8 to 32.3)	1 236 (1 152 to 1 324)	176 (163 to 189)	34.8 (31.0 to 39.4)	379 (266 to 497)	54 (38 to 71)	21.1 (15.8 to 25.5)
Myanmar	1 334 (1 122 to 1 583)	5 (4 to 6)	18.5 (9.1 to 26.4)	57 676 (45 687 to 79 021)	208 (166 to 286)	70.7 (34.2 to 128.3)	22 544 (15 065 to 31 756)	81 (53 to 112)	47.8 (17.4 to 95.4)
Philippines	2 884 (2 312 to 3 884)	6 (5 to 8)	7.4 (-13.6 to 25.5)	83 128 (72 855 to 103 912)	177 (155 to 222)	31.4 (15.3 to 41.1)	30 334 (20 478 to 41 028)	65 (44 to 88)	19.4 (8.8 to 32.5)
Sri Lanka	634 (528 to 763)	6 (5 to 7)	-57.1 (-83.3 to -5.6)	36 698 (24 908 to 66 831)	326 (220 to 596)	91.3 (48.3 to 161.4)	12 826 (6 308 to 22 542)	114 (51 to 206)	77.1 (16.1 to 130.2)
Seychelles	3 (2 to 4)	6 (5 to 7)	28.9 (23.1 to 35.3)	95 (89 to 102)	192 (179 to 206)	32.8 (26.9 to 37.9)	30 (21 to 39)	61 (42 to 80)	14.7 (8.1 to 20.4)
Thailand	2 138 (1 732 to 2 689)	6 (5 to 8)	7.9 (2.4 to 13.8)	80 149 (75 096 to 85 657)	203 (190 to 217)	21.1 (17.5 to 24.9)	24 694 (17 154 to 31 866)	63 (44 to 81)	1.3 (-4.1 to 6.1)
Timor-Leste	27 (23 to 33)	5 (4 to 6)	-64.1 (-86.9 to -11.6)	2 106 (916 to 5 385)	467 (197 to 1 217)	49.4 (29.6 to 73.7)	953 (235 to 2 058)	209 (46 to 455)	36.5 (28.6 to 43.0)
Vietnam	2 715 (2 246 to 3 296)	6 (5 to 7)	26.9 (20.9 to 33.5)	88 246 (82 208 to 94 462)	180 (168 to 193)	43.0 (38.1 to 46.9)	29 124 (20 716 to 37 963)	59 (42 to 76)	21.5 (15.0 to 27.6)

Location	Incidence (95% UI)			Prevalence (95% UI)			YLDs (95% UI)		
	2016 counts	2016 age-standardised rates per 100,000	Percentage change in age-standardised rates between 1990 and 2016	2016 counts	2016 age-standardised rates per 100,000	Percentage change in age-standardised rates between 1990 and 2016	2016 counts	2016 age-standardised rates per 100,000	Percentage change in age-standardised rates between 1990 and 2016
**Oceania**	**309 (258 to 372)**	**6 (5 to 7)**	**17.7 (2.8 to 27.2)**	**7 286 (6 746 to 7 902)**	**145 (135 to 156)**	**40.2 (35.6 to 47.0)**	**2 838 (2 013 to 3 628)**	**56 (40 to 71)**	**33.3 (27.0 to 40.0)**
American Samoa	2 (2 to 3)	6 (5 to 8)	19.1 (14.7 to 24.0)	73 (68 to 79)	192 (179 to 207)	20.7 (16.2 to 26.8)	24 (17 to 32)	64 (44 to 83)	11.0 (4.5 to 17.1)
Federated States of Micronesia	3 (2 to 3)	6 (5 to 7)	23.2 (18.2 to 29.0)	69 (64 to 74)	149 (140 to 160)	30.2 (25.7 to 35.9)	26 (18 to 33)	56 (39 to 71)	19.0 (11.2 to 27.0)
Fiji	22 (19 to 27)	6 (5 to 7)	30.5 (24.6 to 37.3)	669 (617 to 720)	154 (143 to 166)	35.6 (32.1 to 39.3)	239 (171 to 307)	55 (39 to 71)	29.5 (21.3 to 38.3)
Guam	6 (5 to 7)	7 (5 to 8)	27.4 (21.9 to 33.4)	177 (166 to 189)	204 (191 to 218)	28.5 (24.3 to 32.6)	55 (38 to 71)	63 (44 to 82)	21.9 (16.3 to 27.1)
Kiribati	3 (2 to 3)	5 (4 to 6)	30.1 (25.3 to 35.5)	73 (68 to 80)	136 (126 to 148)	49.9 (43.8 to 61.2)	31 (22 to 39)	57 (40 to 72)	45.8 (36.8 to 55.3)
Marshall Islands	2 (2 to 2)	5 (4 to 6)	24.8 (19.4 to 31.0)	46 (42 to 49)	137 (128 to 147)	20.9 (17.6 to 24.4)	17 (12 to 22)	51 (36 to 65)	13.1 (7.6 to 17.8)
Northern Mariana Islands	4 (3 to 5)	7 (6 to 8)	7.0 (3.7 to 10.7)	120 (110 to 130)	207 (192 to 221)	9.2 (6.1 to 13.0)	36 (25 to 47)	61 (43 to 81)	-0.2 (-6.3 to 6.0)
Papua New Guinea	217 (181 to 262)	6 (5 to 7)	14.4 (-5.2 to 26.1)	4 818 (4 444 to 5 260)	140 (130 to 152)	46.4 (40.0 to 55.5)	1 934 (1 382 to 2 464)	56 (39 to 71)	39.8 (30.6 to 49.3)
Samoa	5 (5 to 6)	6 (5 to 7)	24.6 (19.2 to 31.3)	145 (133 to 160)	172 (159 to 189)	35.4 (29.0 to 46.4)	52 (37 to 67)	62 (44 to 80)	26.5 (17.9 to 35.9)
Solomon Islands	16 (13 to 19)	5 (5 to 7)	27.3 (21.5 to 34.8)	360 (332 to 389)	137 (127 to 148)	33.1 (29.1 to 37.0)	144 (102 to 182)	54 (39 to 69)	28.6 (22.2 to 35.7)
Tonga	3 (3 to 4)	6 (5 to 7)	10.9 (6.2 to 16.3)	79 (73 to 85)	165 (153 to 175)	23.1 (19.3 to 26.9)	28 (20 to 36)	58 (42 to 75)	14.6 (8.3 to 20.4)
Vanuatu	7 (6 to 9)	5 (4 to 7)	30.0 (23.7 to 37.7)	175 (160 to 193)	143 (131 to 158)	38.5 (33.3 to 44.8)	70 (50 to 89)	57 (40 to 73)	35.2 (28.0 to 42.5)
**North Africa and Middle East**	**43 739 (21 925 to 105 705)**	**15 (8 to 35)**	**67.2 (-6.4 to 246.8)**	**1 001 277 (645 202 to 2 078 155)**	**372 (244 to 754)**	**6.6 (0.1 to 16.1)**	**403 586 (163 027 to 755 911)**	**147 (65 to 278)**	**-3.4 (-10.2 to 0.9)**
**North Africa and Middle East**	**43 739 (21 925 to 105 705)**	**15 (8 to 35)**	**67.2 (-6.4 to 246.8)**	**1 001 277 (645 202 to 2 078 155)**	**372 (244 to 754)**	**6.6 (0.1 to 16.1)**	**403 586 (163 027 to 755 911)**	**147 (65 to 278)**	**-3.4 (-10.2 to 0.9)**
Afghanistan	5 485 (1 471 to 16 480)	27 (8 to 79)	154.5 (-2.0 to 395.3)	129 928 (37 052 to 423 077)	1 055 (272 to 3 458)	-11.5 (-22.2 to 33.4)	64 755 (8 255 to 156 607)	526 (55 to 1 365)	-15.3 (-18.1 to 4.8)
Algeria	1 380 (1 155 to 1 630)	7 (6 to 8)	-4.7 (-7.7 to -1.5)	45 911 (41 405 to 54 281)	238 (216 to 278)	10.1 (3.1 to 26.3)	15 200 (10 267 to 20 696)	79 (55 to 104)	-1.9 (-13.0 to 11.9)
Bahrain	40 (33 to 47)	8 (6 to 9)	-0.3 (-5.0 to 4.3)	1 421 (1 309 to 1 546)	272 (252 to 295)	15.0 (10.3 to 20.8)	431 (299 to 566)	82 (57 to 107)	-2.1 (-7.9 to 3.8)
Egypt	3 093 (2 560 to 3 757)	7 (5 to 8)	22.1 (14.1 to 42.3)	89 827 (81 706 to 101 465)	210 (192 to 236)	23.3 (18.2 to 29.3)	30 557 (21 196 to 40 182)	71 (50 to 94)	5.5 (-0.9 to 11.0)
Iran	2 665 (2 218 to 3 245)	7 (6 to 8)	-57.3 (-78.1 to -21.5)	159 318 (101 549 to 325 468)	382 (251 to 749)	-15.0 (-24.6 to -2.1)	57 546 (22 560 to 108 931)	135 (62 to 246)	-27.5 (-33.6 to -12.1)
Iraq	6 407 (1 995 to 19 505)	27 (9 to 79)	213.4 (17.7 to 666.3)	162 302 (53 899 to 478 455)	1 029 (342 to 3 000)	-3.6 (-15.0 to 17.9)	72 642 (10 863 to 179 621)	469 (67 to 1 112)	-7.8 (-11.4 to 5.0)
Jordan	464 (255 to 995)	11 (6 to 23)	69.7 (1.3 to 270.1)	7 210 (6 589 to 7 890)	214 (197 to 231)	4.8 (0.2 to 10.1)	2 219 (1 549 to 2 924)	65 (45 to 85)	-9.2 (-15.4 to -3.0)
Kuwait	123 (103 to 147)	8 (6 to 9)	-61.6 (-87.0 to -13.0)	4 886 (4 395 to 5 752)	281 (257 to 319)	6.0 (1.5 to 13.9)	1 483 (1 002 to 2 021)	84 (57 to 114)	0.7 (-6.1 to 7.8)
Lebanon	266 (182 to 465)	10 (7 to 18)	-64.2 (-80.9 to -27.7)	35 349 (11 718 to 109 681)	1 233 (402 to 3 770)	-24.5 (-30.8 to -6.2)	14 455 (1 839 to 36 646)	501 (64 to 1 255)	-33.4 (-38.0 to 3.2)
Libya	476 (215 to 1 282)	16 (7 to 42)	138.0 (15.2 to 537.4)	14 446 (7 789 to 32 714)	455 (254 to 1 008)	81.7 (11.5 to 233.2)	5 625 (1 836 to 11 243)	166 (55 to 339)	81.7 (-15.3 to 173.2)
Morocco	1 018 (852 to 1 195)	6 (5 to 7)	1.6 (-4.4 to 5.4)	37 051 (34 058 to 40 765)	213 (196 to 234)	13.0 (6.9 to 17.5)	12 540 (8 660 to 16 374)	72 (50 to 94)	-1.7 (-6.9 to 2.8)
Palestine	209 (164 to 280)	7 (6 to 9)	-25.4 (-54.9 to 1.6)	9 528 (4 877 to 22 924)	504 (241 to 1 250)	-11.3 (-20.3 to 6.4)	3 869 (1 186 to 7 984)	207 (56 to 447)	-16.3 (-20.1 to -4.1)
Oman	126 (104 to 152)	8 (6 to 10)	-6.0 (-10.3 to -1.9)	3 710 (3 421 to 4 050)	265 (246 to 288)	3.4 (-0.4 to 7.2)	1 088 (752 to 1 427)	77 (54 to 102)	-12.9 (-17.0 to -9.2)
Qatar	48 (40 to 59)	8 (6 to 9)	-3.0 (-5.7 to -0.3)	1 687 (1 555 to 1 839)	281 (261 to 305)	7.5 (4.7 to 10.4)	496 (347 to 652)	82 (57 to 108)	-5.9 (-10.3 to -1.9)
Saudi Arabia	985 (827 to 1 167)	7 (6 to 9)	-17.0 (-19.7 to -14.1)	30 709 (28 460 to 33 049)	244 (227 to 261)	-11.3 (-13.5 to -8.7)	9 032 (6 282 to 11 920)	71 (50 to 93)	-26.7 (-30.6 to -23.1)
Sudan	1 713 (1 196 to 2 922)	8 (6 to 13)	8.6 (-12.1 to 32.4)	45 008 (33 512 to 76 132)	249 (189 to 414)	30.9 (23.2 to 44.0)	17 874 (9 949 to 29 898)	98 (56 to 160)	20.4 (8.0 to 29.2)
Syria	10 283 (1 593 to 36 314)	100 (16 to 353)	1 632.9 (174.9 to 6 471.1)	63 200 (24 350 to 184 162)	654 (281 to 1 813)	201.5 (55.2 to 470.1)	25 941 (4 834 to 61 675)	269 (58 to 627)	217.1 (5.6 to 389.8)
Tunisia	412 (342 to 505)	8 (6 to 9)	8.6 (2.0 to 25.9)	14 521 (13 336 to 15 930)	246 (226 to 270)	15.5 (9.9 to 20.7)	4 483 (3 131 to 5 911)	76 (54 to 101)	-1.2 (-6.9 to 4.4)
Turkey	3 051 (2 448 to 3 918)	8 (6 to 10)	-5.4 (-15.0 to 16.8)	105 395 (96 495 to 117 800)	256 (235 to 286)	-3.4 (-10.1 to 7.6)	31 786 (22 180 to 42 388)	77 (53 to 103)	-21.8 (-29.6 to -13.2)
United Arab Emirates	228 (189 to 276)	8 (7 to 10)	-9.3 (-12.1 to -6.5)	6 957 (6 369 to 7 618)	282 (260 to 306)	-4.0 (-7.8 to -0.3)	2 129 (1 473 to 2 791)	85 (60 to 112)	-17.9 (-23.4 to -12.9)
Yemen	5 226 (1 609 to 16 218)	30 (10 to 94)	372.8 (57.1 to 1 389.2)	31 846 (22 842 to 58 425)	254 (191 to 439)	22.9 (11.0 to 41.3)	13 157 (6 878 to 22 465)	103 (57 to 171)	14.4 (-4.0 to 29.1)
**South Asia**	**75 783 (62 567 to 92 173)**	**10 (8 to 12)**	**-4.6 (-8.0 to -1.4)**	**1 841 525 (1 714 971 to 1 980 627)**	**235 (220 to 252)**	**20.5 (18.4 to 23.1)**	**700 868 (494 701 to 890 625)**	**89 (63 to 113)**	**9.2 (6.2 to 12.1)**
**South Asia**	**75 783 (62 567 to 92 173)**	**10 (8 to 12)**	**-4.6 (-8.0 to -1.4)**	**1 841 525 (1 714 971 to 1 980 627)**	**235 (220 to 252)**	**20.5 (18.4 to 23.1)**	**700 868 (494 701 to 890 625)**	**89 (63 to 113)**	**9.2 (6.2 to 12.1)**
Bangladesh	6 197 (5 026 to 7 725)	8 (7 to 10)	12.6 (7.2 to 18.0)	164 168 (150 528 to 182 026)	219 (201 to 243)	45.0 (38.2 to 53.7)	59 485 (42 447 to 76 152)	79 (55 to 102)	19.9 (13.2 to 26.2)
Bhutan	31 (26 to 37)	9 (7 to 11)	-1.5 (-4.9 to 2.0)	809 (743 to 880)	241 (223 to 260)	32.4 (27.6 to 37.9)	293 (205 to 376)	87 (62 to 112)	11.8 (5.5 to 17.9)
India	60 691 (49 926 to 73 900)	10 (9 to 13)	-7.5 (-11.5 to -4.1)	1 459 271 (1 363 384 to 1 568 657)	239 (223 to 256)	16.6 (14.8 to 18.3)	550 970 (392 783 to 699 587)	90 (64 to 115)	5.6 (2.5 to 8.3)
Nepal	1 291 (1 063 to 1 553)	9 (7 to 11)	2.7 (-0.8 to 6.4)	32 529 (29 099 to 37 224)	230 (207 to 259)	45.1 (35.6 to 63.9)	12 622 (8 927 to 16 286)	89 (63 to 114)	31.8 (20.6 to 45.0)
Pakistan	7 573 (6 263 to 9 042)	8 (7 to 10)	16.5 (12.4 to 21.0)	184 748 (168 933 to 203 193)	219 (202 to 240)	38.8 (31.7 to 48.8)	72 072 (50 484 to 91 797)	85 (61 to 110)	30.8 (23.2 to 39.9)
**Sub-Saharan Africa**	**31 365 (26 122 to 37 669)**	**7 (5 to 8)**	**-29.3 (-53.9 to -10.8)**	**734 750 (604 261 to 1 047 535)**	**181 (148 to 262)**	**14.3 (6.4 to 30.3)**	**307 378 (194 104 to 442 409)**	**76 (50 to 107)**	**8.4 (-3.7 to 20.3)**
**Southern sub-Saharan Africa**	**2 356 (1 955 to 2 854)**	**6 (5 to 7)**	**-20.6 (-37.5 to -11.8)**	**59 759 (55 074 to 65 316)**	**159 (148 to 175)**	**-13.7 (-16.2 to -10.4)**	**22 035 (15 747 to 28 509)**	**58 (41 to 75)**	**-17.6 (-20.4 to -15.1)**
Botswana	70 (58 to 86)	6 (5 to 8)	11.6 (7.6 to 15.5)	1 817 (1 677 to 1 962)	166 (155 to 178)	8.9 (6.0 to 12.3)	633 (439 to 831)	58 (41 to 76)	-2.2 (-13.6 to 9.2)
Lesotho	61 (50 to 73)	6 (5 to 7)	15.1 (10.0 to 20.4)	1 156 (1 068 to 1 252)	109 (101 to 116)	-13.0 (-16.1 to -9.7)	459 (328 to 584)	43 (31 to 54)	-14.7 (-20.3 to -8.8)
Namibia	71 (59 to 86)	5 (5 to 7)	3.0 (0.1 to 5.8)	1 773 (1 627 to 1 924)	151 (139 to 162)	8.3 (5.0 to 12.3)	650 (470 to 829)	55 (39 to 70)	-3.3 (-11.4 to 4.4)
South Africa	1 663 (1 370 to 2 042)	6 (5 to 8)	-25.9 (-45.3 to -15.4)	45 480 (41 854 to 50 674)	170 (157 to 191)	-16.4 (-19.3 to -12.7)	16 222 (11 446 to 21 212)	61 (43 to 79)	-22.0 (-25.0 to -19.1)
Swaziland	42 (34 to 51)	6 (5 to 8)	10.1 (6.4 to 14.3)	849 (782 to 919)	140 (131 to 150)	-4.6 (-7.1 to -1.8)	318 (221 to 411)	52 (37 to 67)	-10.9 (-18.8 to -3.2)
Zimbabwe	448 (373 to 530)	6 (5 to 7)	0.8 (-2.9 to 4.1)	8 683 (7 928 to 9 424)	122 (113 to 131)	-2.5 (-4.4 to -0.4)	3 479 (2 484 to 4 440)	49 (34 to 62)	1.5 (-4.9 to 9.3)
**Western sub-Saharan Africa**	**12 525 (10 427 to 14 960)**	**7 (6 to 8)**	**-7.7 (-15.7 to -3.4)**	**252 366 (225 944 to 288 488)**	**155 (140 to 175)**	**10.7 (6.5 to 20.9)**	**100 883 (71 631 to 129 395)**	**61 (43 to 79)**	**3.9 (-2.4 to 11.7)**
Benin	345 (287 to 412)	7 (5 to 8)	0.8 (-2.4 to 3.9)	6 837 (6 264 to 7 511)	147 (136 to 159)	5.7 (2.6 to 8.8)	2 751 (1 956 to 3 484)	59 (42 to 75)	-1.1 (-4.1 to 1.7)
Burkina Faso	620 (512 to 740)	7 (6 to 8)	-3.3 (-7.0 to 1.1)	11 308 (10 336 to 12 392)	149 (138 to 160)	9.9 (7.0 to 14.0)	4 579 (3 263 to 5 789)	60 (43 to 76)	1.4 (-1.8 to 4.8)
Cameroon	791 (648 to 955)	7 (6 to 8)	1.8 (-3.3 to 12.4)	14 551 (13 117 to 15 994)	142 (130 to 155)	-2.6 (-5.7 to 1.5)	5 835 (4 216 to 7 544)	57 (41 to 73)	-7.7 (-12.0 to -3.2)
Cape Verde	16 (14 to 19)	6 (5 to 7)	2.4 (-1.2 to 6.0)	453 (418 to 491)	181 (168 to 195)	12.0 (9.0 to 15.2)	155 (109 to 199)	62 (44 to 80)	-0.1 (-4.2 to 3.9)
Chad	463 (382 to 552)	7 (5 to 8)	-29.4 (-61.9 to -0.9)	10 147 (8 019 to 14 983)	184 (142 to 286)	4.6 (-2.7 to 10.6)	4 437 (2 763 to 6 778)	80 (49 to 118)	0.3 (-3.3 to 5.1)
Cote d'Ivoire	719 (596 to 852)	7 (6 to 8)	-3.4 (-6.4 to -0.1)	14 121 (12 765 to 15 682)	146 (133 to 159)	2.4 (-0.8 to 8.1)	5 835 (4 170 to 7 421)	60 (43 to 76)	-1.5 (-5.8 to 3.8)
The Gambia	62 (51 to 75)	6 (5 to 8)	-10.0 (-12.6 to -7.3)	1 303 (1 169 to 1 467)	161 (145 to 187)	-1.8 (-7.4 to 2.1)	516 (360 to 661)	64 (45 to 83)	-7.0 (-12.1 to -2.0)
Ghana	875 (725 to 1 036)	7 (6 to 8)	5.5 (2.2 to 9.0)	18 813 (17 222 to 20 543)	154 (142 to 166)	18.1 (13.6 to 22.8)	7 179 (5 017 to 9 260)	58 (42 to 74)	8.6 (4.1 to 13.3)
Guinea	369 (307 to 440)	6 (5 to 7)	-8.1 (-11.2 to -4.8)	7 072 (6 407 to 7 820)	130 (119 to 142)	-1.7 (-5.8 to 3.8)	2 942 (2 078 to 3 759)	53 (38 to 68)	-6.3 (-10.6 to -1.9)
Guinea-Bissau	60 (50 to 72)	7 (6 to 8)	-10.7 (-12.9 to -8.1)	1 181 (1 053 to 1 363)	142 (128 to 163)	3.5 (-3.1 to 19.5)	503 (352 to 657)	60 (43 to 77)	-1.3 (-9.8 to 9.5)
Liberia	126 (104 to 151)	6 (5 to 7)	-83.6 (-94.5 to -48.3)	4 763 (2 805 to 10 394)	247 (146 to 544)	74.8 (14.0 to 205.7)	2 137 (876 to 4 203)	113 (42 to 222)	82.8 (-8.5 to 185.2)
Mali	526 (436 to 639)	6 (5 to 7)	-12.1 (-23.8 to -5.3)	11 047 (9 048 to 16 222)	154 (128 to 222)	32.0 (12.7 to 88.0)	4 560 (2 938 to 6 650)	62 (41 to 89)	22.7 (-3.5 to 58.4)
Mauritania	116 (97 to 139)	6 (5 to 7)	-30.0 (-59.1 to -6.5)	2 794 (2 543 to 3 069)	158 (146 to 173)	14.6 (11.3 to 18.1)	1 060 (744 to 1 374)	60 (42 to 77)	3.5 (-2.1 to 8.4)
Niger	577 (479 to 697)	6 (5 to 7)	-13.7 (-24.0 to -7.8)	9 854 (8 892 to 10 870)	126 (115 to 137)	1.1 (-2.9 to 5.2)	4 033 (2 874 to 5 122)	51 (37 to 65)	-5.7 (-9.8 to -1.8)
Nigeria	5 965 (4 867 to 7 381)	7 (6 to 8)	-0.5 (-6.3 to 13.0)	117 879 (105 846 to 132 013)	158 (144 to 174)	11.1 (6.9 to 18.4)	44 432 (31 013 to 56 856)	59 (42 to 77)	2.0 (-4.5 to 8.9)
Sao Tome and Principe	6 (5 to 7)	6 (5 to 7)	4.1 (1.3 to 6.7)	129 (117 to 141)	156 (143 to 169)	11.0 (7.6 to 13.9)	49 (35 to 63)	59 (43 to 76)	2.1 (-2.5 to 6.3)

Location	Incidence (95% UI)			Prevalence (95% UI)			YLDs (95% UI)		
	2016 counts	2016 age-standardised rates per 100,000	Percentage change in age-standardised rates between 1990 and 2016	2016 counts	2016 age-standardised rates per 100,000	Percentage change in age-standardised rates between 1990 and 2016	2016 counts	2016 age-standardised rates per 100,000	Percentage change in age-standardised rates between 1990 and 2016
Senegal	473<br>(392 to 560)	6<br>(5 to 8)	-1.6<br>(-7.1 to 2.4)	9 955<br>(9 096 to 10 896)	152<br>(140 to 164)	8.5<br>(4.7 to 14.1)	4 019<br>(2 862 to 5 101)	61<br>(43 to 77)	3.6<br>(-0.4 to 8.2)
Sierra Leone	199<br>(164 to 239)	7<br>(5 to 8)	9.0<br>(5.4 to 12.6)	5 662<br>(3 975 to 10 255)	201<br>(143 to 359)	56.4<br>(11.9 to 174.5)	2 464<br>(1 282 to 4 107)	87<br>(47 to 146)	58.6<br>(-3.2 to 155.8)
Togo	215<br>(178 to 257)	6<br>(5 to 7)	-6.0<br>(-9.0 to -2.9)	4 496<br>(4 097 to 4 955)	140<br>(129 to 151)	0.9<br>(-2.1 to 4.7)	1 799<br>(1 277 to 2 294)	56<br>(39 to 70)	-4.9<br>(-8.4 to -1.4)
**Eastern sub-Saharan Africa**	**12 649<br>(10 516 to 15 286)**	**7<br>(6 to 8)**	**-45.8<br>(-72.1 to -16.9)**	**334 541<br>(248 501 to 565 231)**	**216<br>(156 to 373)**	**25.9<br>(14.7 to 44.4)**	**145 234<br>(81 923 to 237 980)**	**95<br>(51 to 155)**	**19.1<br>(2.3 to 29.8)**
Burundi	410<br>(338 to 492)	7<br>(6 to 9)	1.8<br>(-2.3 to 9.9)	11 152<br>(7 660 to 20 467)	233<br>(160 to 430)	92.9<br>(34.1 to 257.9)	5 048<br>(2 609 to 8 528)	102<br>(53 to 171)	92.3<br>(12.4 to 211.5)
Comoros	23<br>(19 to 28)	7<br>(5 to 8)	-16.0<br>(-18.7 to -13.0)	543<br>(496 to 593)	162<br>(150 to 176)	-1.1<br>(-4.3 to 3.1)	217<br>(158 to 279)	65<br>(46 to 82)	-8.5<br>(-12.1 to -4.7)
Djibouti	32<br>(27 to 39)	7<br>(6 to 8)	-21.9<br>(-47.0 to -6.3)	740<br>(672 to 830)	177<br>(162 to 198)	2.5<br>(-2.5 to 12.2)	295<br>(210 to 379)	70<br>(49 to 91)	-3.6<br>(-10.9 to 4.9)
Eritrea	166<br>(137 to 198)	7<br>(6 to 8)	-2.1<br>(-5.4 to 1.5)	5 107<br>(3 540 to 9 540)	219<br>(156 to 398)	77.5<br>(27.2 to 220.4)	2 290<br>(1 164 to 3 916)	98<br>(51 to 163)	78.3<br>(7.4 to 175.9)
Ethiopia	3 190<br>(2 656 to 3 809)	7<br>(6 to 8)	-72.8<br>(-89.7 to -34.8)	87 336<br>(64 853 to 151 165)	211<br>(153 to 378)	19.3<br>(10.8 to 27.6)	38 837<br>(21 114 to 63 186)	93<br>(50 to 152)	8.8<br>(4.8 to 13.9)
Kenya	1 489<br>(1 237 to 1 756)	7<br>(6 to 8)	-0.1<br>(-2.0 to 1.6)	33 909<br>(31 068 to 36 902)	171<br>(158 to 184)	14.2<br>(12.4 to 16.9)	12 948<br>(9 227 to 16 633)	65<br>(46 to 82)	9.6<br>(4.2 to 14.5)
Madagascar	761<br>(627 to 907)	6<br>(5 to 8)	-6.3<br>(-9.6 to -2.6)	15 066<br>(13 673 to 16 567)	143<br>(131 to 155)	2.3<br>(-1.5 to 6.7)	6 124<br>(4 423 to 7 848)	58<br>(41 to 73)	-2.9<br>(-7.0 to 1.4)
Malawi	509<br>(421 to 610)	6<br>(5 to 7)	-17.8<br>(-21.2 to -14.4)	9 022<br>(8 172 to 9 877)	121<br>(111 to 132)	-4.9<br>(-8.5 to -0.7)	3 587<br>(2 541 to 4 548)	48<br>(34 to 61)	-12.2<br>(-17.0 to -7.7)
Mozambique	906<br>(750 to 1 082)	6<br>(5 to 8)	-24.4<br>(-56.1 to -1.3)	25 546<br>(17 038 to 50 639)	236<br>(145 to 503)	-15.5<br>(-27.5 to 2.8)	11 249<br>(5 623 to 19 583)	107<br>(47 to 197)	-23.5<br>(-29.8 to -1.3)
Rwanda	371<br>(306 to 441)	6<br>(5 to 7)	-55.1<br>(-79.3 to -26.6)	44 209<br>(13 809 to 129 873)	866<br>(262 to 2 513)	541.2<br>(94.5 to 1 648.1)	21 604<br>(2 892 to 50 885)	429<br>(53 to 992)	626.1<br>(2.1 to 1 401.7)
Somalia	649<br>(359 to 1 435)	12<br>(7 to 24)	-19.6<br>(-27.8 to -9.5)	11 073<br>(6 746 to 23 541)	242<br>(149 to 517)	35.2<br>(6.0 to 77.5)	5 296<br>(2 299 to 10 266)	113<br>(48 to 209)	34.9<br>(-5.7 to 64.7)
South Sudan	541<br>(421 to 750)	8<br>(6 to 11)	-57.0<br>(-79.7 to -17.3)	13 632<br>(8 590 to 28 264)	252<br>(159 to 527)	35.4<br>(0.8 to 92.9)	6 168<br>(2 758 to 11 359)	115<br>(51 to 209)	39.7<br>(-11.0 to 86.1)
Tanzania	1 773<br>(1 471 to 2 126)	7<br>(6 to 8)	-6.7<br>(-10.1 to -3.8)	34 693<br>(31 498 to 38 013)	153<br>(141 to 165)	6.7<br>(3.8 to 9.8)	13 690<br>(9 888 to 17 270)	60<br>(43 to 77)	-0.8<br>(-4.6 to 3.1)
Uganda	1 261<br>(1 038 to 1 499)	6<br>(5 to 8)	-16.0<br>(-33.2 to -7.2)	32 137<br>(23 594 to 55 298)	225<br>(151 to 435)	-4.8<br>(-21.3 to 19.4)	13 980<br>(7 652 to 22 847)	99<br>(48 to 172)	-13.0<br>(-23.5 to 14.5)
Zambia	560<br>(464 to 668)	7<br>(6 to 9)	-7.4<br>(-12.1 to -3.6)	10 150<br>(9 217 to 11 180)	146<br>(134 to 160)	2.9<br>(-0.4 to 6.1)	4 117<br>(2 966 to 5 251)	59<br>(42 to 76)	-1.3<br>(-6.6 to 3.9)
**Central sub-Saharan Africa**	**3 835<br>(3 178 to 4 618)**	**6<br>(5 to 8)**	**-12.7<br>(-30.5 to -3.7)**	**88 083<br>(70 420 to 133 226)**	**183<br>(144 to 281)**	**21.1<br>(9.8 to 42.7)**	**38 206<br>(23 820 to 57 449)**	**78<br>(48 to 118)**	**17.1<br>(0.4 to 31.4)**
Angola	932<br>(771 to 1 118)	7<br>(6 to 9)	-35.2<br>(-66.6 to -7.0)	24 461<br>(17 919 to 41 306)	257<br>(178 to 471)	6.0<br>(-5.8 to 19.9)	10 469<br>(5 781 to 17 132)	112<br>(56 to 190)	-3.2<br>(-10.1 to 9.2)
Central African Republic	165<br>(131 to 214)	6<br>(5 to 8)	8.4<br>(-2.2 to 37.5)	3 279<br>(2 555 to 5 079)	134<br>(108 to 196)	20.7<br>(-0.5 to 76.2)	1 475<br>(895 to 2 194)	60<br>(38 to 87)	24.8<br>(-7.2 to 70.9)
Congo	170<br>(140 to 204)	7<br>(6 to 9)	-3.4<br>(-7.2 to 2.3)	4 531<br>(3 277 to 7 816)	223<br>(161 to 392)	56.0<br>(13.3 to 171.2)	1 916<br>(1 046 to 3 201)	94<br>(50 to 159)	56.0<br>(-2.7 to 143.0)
DR Congo	2 472<br>(2 053 to 2 961)	6<br>(5 to 7)	-2.0<br>(-5.2 to 2.6)	53 656<br>(44 322 to 76 465)	161<br>(134 to 228)	28.2<br>(8.6 to 81.0)	22 486<br>(14 674 to 33 039)	68<br>(44 to 99)	27.9<br>(-0.6 to 64.9)
Equatorial Guinea	30<br>(25 to 36)	8<br>(6 to 9)	12.4<br>(5.9 to 17.7)	664<br>(608 to 716)	185<br>(170 to 198)	52.0<br>(45.3 to 57.6)	235<br>(164 to 308)	65<br>(46 to 85)	21.3<br>(8.7 to 33.2)
Gabon	67<br>(55 to 80)	8<br>(6 to 10)	-5.6<br>(-8.2 to -2.9)	1 492<br>(1 369 to 1 618)	195<br>(180 to 210)	2.9<br>(0.1 to 5.6)	565<br>(403 to 726)	73<br>(53 to 93)	-4.9<br>(-9.6 to -0.7)

Appendix Table 7: Incidence, prevalence, and YLDs for 1990 by location for SCI

Location	Incidence (95% UI)		Prevalence (95% UI)		YLDs (95% UI)	
	1990 counts	1990 age-standardised rates per 100,000	1990 counts	1990 age-standardised rates per 100,000	1990 counts	1990 age-standardised rates per 100,000
**Global**	**673 482 (569 730 to 799 303)**	**13 (11 to 16)**	**17 347 936 (15 971 534 to 19 260 964)**	**368 (342 to 402)**	**6 805 450 (4 823 262 to 8 731 434)**	**144 (102 to 185)**
**High SDI**	**228 034 (180 626 to 284 627)**	**26 (20 to 32)**	**7 297 623 (6 726 617 to 7 904 398)**	**772 (711 to 837)**	**2 168 918 (1 500 402 to 2 853 967)**	**230 (161 to 299)**
**High-middle SDI**	**135 555 (113 534 to 160 034)**	**16 (13 to 18)**	**3 792 173 (3 481 729 to 4 281 330)**	**441 (407 to 494)**	**1 377 953 (967 583 to 1 787 496)**	**159 (110 to 205)**
**Middle SDI**	**131 477 (113 743 to 154 405)**	**7 (6 to 9)**	**2 997 355 (2 791 322 to 3 226 200)**	**184 (172 to 196)**	**1 186 351 (837 729 to 1 513 325)**	**72 (51 to 92)**
**Low-middle SDI**	**126 688 (107 781 to 148 361)**	**10 (8 to 12)**	**2 488 295 (2 186 519 to 3 135 058)**	**212 (191 to 258)**	**1 185 463 (826 456 to 1 614 245)**	**98 (68 to 130)**
**Low SDI**	**54 677 (37 344 to 92 552)**	**15 (10 to 24)**	**866 891 (603 463 to 1 559 475)**	**266 (197 to 441)**	**515 813 (262 669 to 832 341)**	**152 (81 to 233)**
**High-income**	**233 705 (184 378 to 291 441)**	**26 (21 to 33)**	**7 566 804 (6 974 147 to 8 202 030)**	**793 (729 to 860)**	**2 213 591 (1 537 186 to 2 901 777)**	**232 (160 to 308)**
**High-income North America**	**69 107 (55 707 to 84 917)**	**25 (20 to 31)**	**2 198 588 (2 022 133 to 2 373 364)**	**758 (697 to 820)**	**641 698 (447 987 to 850 037)**	**222 (155 to 292)**
Canada	6 930 (5 505 to 8 650)	25 (20 to 32)	218 725 (201 304 to 235 630)	748 (688 to 807)	64 132 (44 271 to 84 382)	219 (154 to 285)
Greenland	17 (13 to 21)	37 (29 to 46)	342 (313 to 370)	719 (660 to 775)	117 (83 to 151)	245 (173 to 314)
United States	62 130 (50 178 to 76 204)	25 (20 to 31)	1 978 566 (1 818 865 to 2 139 272)	759 (697 to 822)	577 335 (396 153 to 760 812)	222 (154 to 295)
**Australasia**	**4 688 (3 708 to 5 857)**	**23 (18 to 29)**	**153 018 (140 048 to 166 214)**	**721 (660 to 784)**	**44 848 (30 997 to 59 261)**	**212 (148 to 277)**
Australia	3 859 (3 048 to 4 825)	23 (18 to 29)	126 888 (115 891 to 138 157)	714 (652 to 778)	37 124 (25 613 to 48 414)	209 (145 to 275)
New Zealand	829 (656 to 1 036)	25 (19 to 31)	26 130 (23 925 to 28 460)	757 (692 to 825)	7 637 (5 317 to 10 203)	221 (153 to 291)
**High-income Asia-Pacific**	**45 671 (36 318 to 56 707)**	**28 (22 to 35)**	**1 458 757 (1 342 163 to 1 598 224)**	**812 (746 to 892)**	**434 747 (304 110 to 567 180)**	**242 (167 to 318)**
Brunei	87 (69 to 109)	37 (29 to 46)	2 182 (1 971 to 2 441)	990 (911 to 1 088)	709 (500 to 925)	319 (224 to 420)
Japan	31 469 (25 074 to 39 282)	26 (21 to 32)	1 064 467 (978 909 to 1 158 865)	784 (720 to 858)	313 496 (217 302 to 415 655)	231 (162 to 302)
Singapore	669 (532 to 832)	25 (20 to 32)	21 084 (19 175 to 23 264)	757 (693 to 830)	6 512 (4 486 to 8 503)	233 (163 to 305)
South Korea	13 446 (10 614 to 16 765)	33 (26 to 41)	371 024 (335 783 to 414 110)	882 (806 to 976)	123 281 (86 943 to 160 962)	292 (205 to 378)
**Western Europe**	**106 210 (82 309 to 136 481)**	**28 (21 to 35)**	**3 542 072 (3 257 999 to 3 876 661)**	**851 (781 to 934)**	**1 032 757 (723 494 to 1 363 852)**	**249 (172 to 327)**
Andorra	12 (10 to 16)	25 (19 to 32)	455 (414 to 502)	828 (751 to 920)	133 (93 to 175)	243 (169 to 322)
Austria	2 641 (2 019 to 3 438)	34 (26 to 44)	83 855 (76 464 to 92 096)	994 (902 to 1 099)	24 406 (16 838 to 32 135)	290 (201 to 379)
Belgium	2 910 (2 240 to 3 751)	29 (22 to 37)	96 159 (88 081 to 105 494)	876 (799 to 967)	27 928 (19 627 to 36 907)	256 (178 to 342)
Cyprus	182 (143 to 232)	27 (21 to 34)	5 614 (5 127 to 6 166)	844 (771 to 927)	1 740 (1 216 to 2 263)	261 (184 to 346)
Denmark	1 642 (1 254 to 2 157)	30 (23 to 39)	47 701 (43 629 to 52 483)	834 (761 to 924)	13 861 (9 539 to 18 436)	243 (170 to 321)
Finland	1 613 (1 250 to 2 069)	32 (25 to 41)	48 145 (43 981 to 52 906)	877 (799 to 970)	13 976 (9 720 to 18 328)	255 (177 to 338)
France	18 417 (14 100 to 23 860)	31 (24 to 40)	553 501 (507 960 to 606 628)	905 (826 to 995)	161 285 (110 829 to 212 660)	264 (184 to 348)
Germany	21 519 (16 578 to 27 720)	27 (21 to 35)	728 321 (664 723 to 799 157)	818 (744 to 904)	211 468 (147 895 to 278 722)	239 (165 to 312)
Greece	2 778 (2 142 to 3 562)	28 (22 to 36)	99 253 (91 448 to 108 921)	893 (819 to 985)	29 009 (19 969 to 38 333)	261 (182 to 344)
Iceland	66 (51 to 83)	26 (20 to 32)	2 042 (1 858 to 2 258)	803 (732 to 887)	597 (416 to 778)	235 (164 to 309)
Ireland	925 (717 to 1 186)	26 (20 to 33)	29 475 (26 845 to 32 531)	848 (773 to 935)	8 601 (6 036 to 11 310)	248 (169 to 326)
Israel	1 091 (853 to 1 396)	24 (18 to 30)	32 459 (28 698 to 38 448)	768 (682 to 903)	10 122 (7 128 to 13 566)	238 (164 to 323)
Italy	16 100 (12 399 to 20 818)	28 (22 to 36)	560 175 (513 832 to 614 715)	885 (808 to 977)	163 096 (115 659 to 213 712)	259 (179 to 338)
Luxembourg	127 (99 to 161)	34 (26 to 42)	4 189 (3 861 to 4 565)	988 (908 to 1 085)	1 218 (856 to 1 604)	288 (201 to 379)
Malta	100 (78 to 128)	29 (22 to 37)	3 329 (3 036 to 3 689)	906 (825 to 1 004)	980 (680 to 1 290)	268 (186 to 353)
Netherlands	3 304 (2 540 to 4 223)	22 (17 to 28)	113 888 (104 009 to 124 667)	703 (641 to 772)	33 332 (23 409 to 43 607)	206 (144 to 270)
Norway	1 214 (937 to 1 570)	27 (21 to 34)	36 778 (33 546 to 40 546)	799 (726 to 884)	10 725 (7 476 to 14 051)	234 (162 to 307)
Portugal	2 905 (2 259 to 3 685)	29 (23 to 37)	95 188 (86 609 to 104 297)	892 (811 to 981)	29 503 (20 736 to 38 236)	278 (191 to 365)
Spain	9 992 (7 795 to 12 714)	26 (20 to 33)	346 795 (317 276 to 380 043)	833 (760 to 915)	100 918 (71 234 to 132 816)	243 (168 to 322)
Sweden	2 212 (1 722 to 2 831)	26 (20 to 33)	78 844 (71 397 to 87 510)	842 (757 to 942)	22 996 (16 111 to 30 379)	247 (172 to 327)
Switzerland	2 486 (1 912 to 3 248)	36 (28 to 46)	77 284 (70 311 to 85 107)	1 025 (927 to 1 136)	22 597 (15 671 to 29 613)	300 (207 to 394)
United Kingdom	13 869 (10 815 to 17 641)	25 (19 to 31)	495 133 (453 214 to 545 669)	812 (740 to 899)	144 617 (99 684 to 187 966)	238 (166 to 315)
**Southern Latin America**	**8 030 (6 480 to 9 994)**	**17 (13 to 21)**	**214 370 (198 116 to 233 368)**	**460 (426 to 499)**	**74 220 (53 190 to 95 582)**	**159 (112 to 204)**
Argentina	5 349 (4 329 to 6 648)	17 (13 to 21)	144 097 (132 833 to 157 314)	461 (426 to 503)	49 980 (35 825 to 63 696)	160 (113 to 204)
Chile	2 175 (1 739 to 2 704)	17 (14 to 22)	56 415 (51 795 to 61 226)	464 (429 to 500)	19 214 (13 517 to 24 662)	157 (110 to 205)
Uruguay	505 (402 to 629)	16 (13 to 20)	13 848 (12 803 to 15 019)	441 (408 to 479)	4 647 (3 333 to 6 052)	148 (105 to 193)
**Central Europe, Eastern Europe, and Central Asia**	**80 232 (65 936 to 96 111)**	**19 (16 to 23)**	**2 112 350 (1 966 468 to 2 255 713)**	**501 (466 to 535)**	**711 897 (510 486 to 920 412)**	**169 (122 to 217)**

Location	Incidence (95% UI)		Prevalence (95% UI)		YLDs (95% UI)	
	1990 counts	1990 age-standardised rates per 100,000	1990 counts	1990 age-standardised rates per 100,000	1990 counts	1990 age-standardised rates per 100,000
**Eastern Europe**	**44 849 (37 043 to 53 381)**	**20 (17 to 24)**	**1 203 537 (1 124 524 to 1 285 125)**	**515 (481 to 550)**	**383 936 (269 916 to 499 488)**	**165 (117 to 215)**
Belarus	1 907 (1 571 to 2 274)	19 (16 to 22)	53 080 (49 307 to 56 931)	499 (463 to 536)	16 822 (11 802 to 21 806)	158 (111 to 205)
Estonia	361 (298 to 434)	23 (19 to 27)	9 522 (8 865 to 10 190)	572 (532 to 613)	2 927 (2 064 to 3 834)	176 (123 to 229)
Latvia	637 (523 to 771)	24 (19 to 28)	16 211 (15 104 to 17 277)	571 (531 to 609)	5 029 (3 506 to 6 546)	177 (125 to 233)
Lithuania	832 (685 to 1 010)	22 (18 to 27)	21 611 (20 201 to 22 998)	559 (522 to 596)	6 572 (4 571 to 8 505)	171 (118 to 223)
Moldova	836 (695 to 994)	19 (16 to 23)	20 942 (19 480 to 22 424)	482 (449 to 517)	7 044 (4 945 to 9 067)	162 (114 to 209)
Russia	30 720 (25 428 to 36 513)	21 (17 to 25)	808 909 (755 405 to 864 278)	521 (485 to 556)	259 026 (182 367 to 337 924)	167 (118 to 216)
Ukraine	9 556 (7 955 to 11 408)	19 (16 to 22)	273 261 (254 465 to 292 025)	497 (462 to 533)	86 082 (59 878 to 111 870)	157 (111 to 205)
**Central Europe**	**25 957 (20 768 to 31 818)**	**21 (17 to 26)**	**682 814 (630 620 to 731 925)**	**533 (492 to 572)**	**228 284 (160 366 to 292 359)**	**178 (126 to 228)**
Albania	488 (395 to 596)	15 (12 to 18)	13 060 (11 867 to 14 230)	448 (410 to 486)	4 532 (3 174 to 5 850)	155 (108 to 200)
Bosnia and Herzegovina	580 (470 to 705)	13 (10 to 16)	17 915 (16 191 to 19 652)	386 (349 to 422)	6 300 (4 429 to 8 049)	135 (95 to 174)
Bulgaria	1 728 (1 396 to 2 130)	20 (16 to 24)	52 831 (48 696 to 56 995)	553 (507 to 598)	16 652 (11 603 to 21 691)	176 (124 to 227)
Croatia	909 (730 to 1 112)	19 (15 to 23)	25 035 (23 169 to 27 025)	480 (443 to 520)	7 426 (5 169 to 9 710)	143 (99 to 189)
Czech Republic	2 716 (2 120 to 3 379)	26 (20 to 32)	67 541 (62 176 to 72 779)	612 (563 to 661)	20 191 (14 095 to 26 611)	184 (129 to 239)
Hungary	2 776 (2 158 to 3 498)	25 (20 to 31)	60 534 (55 520 to 65 472)	532 (487 to 577)	18 883 (13 329 to 24 709)	166 (118 to 216)
Macedonia	290 (236 to 353)	15 (12 to 18)	8 527 (7 807 to 9 298)	425 (389 to 463)	2 831 (1 962 to 3 651)	141 (99 to 184)
Montenegro	106 (86 to 129)	17 (14 to 21)	3 115 (2 847 to 3 382)	506 (463 to 549)	961 (664 to 1 269)	156 (109 to 204)
Poland	7 903 (6 335 to 9 763)	21 (17 to 26)	199 829 (183 631 to 215 250)	511 (470 to 551)	65 632 (45 620 to 84 322)	168 (119 to 217)
Romania	5 255 (4 237 to 6 404)	22 (18 to 27)	147 002 (134 637 to 158 412)	604 (552 to 652)	48 976 (34 890 to 63 162)	201 (141 to 258)
Serbia	1 448 (1 167 to 1 765)	15 (12 to 19)	43 835 (40 097 to 47 376)	441 (403 to 478)	14 052 (9 858 to 18 312)	142 (99 to 184)
Slovakia	1 222 (976 to 1 508)	23 (19 to 29)	29 763 (27 516 to 31 994)	556 (515 to 598)	9 228 (6 508 to 11 978)	173 (122 to 224)
Slovenia	534 (420 to 668)	26 (21 to 33)	13 827 (12 727 to 14 920)	639 (588 to 690)	4 092 (2 871 to 5 318)	190 (132 to 248)
**Central Asia**	**9 426 (7 852 to 11 163)**	**13 (11 to 16)**	**225 999 (208 542 to 244 705)**	**371 (345 to 399)**	**81 272 (57 360 to 104 948)**	**133 (94 to 171)**
Armenia	527 (436 to 630)	15 (12 to 18)	20 998 (15 363 to 29 920)	615 (454 to 875)	7 228 (4 477 to 10 302)	213 (131 to 308)
Azerbaijan	964 (807 to 1 135)	13 (11 to 15)	23 011 (21 354 to 24 687)	356 (331 to 380)	8 194 (5 863 to 10 536)	126 (90 to 161)
Georgia	735 (603 to 880)	14 (11 to 16)	21 138 (19 599 to 22 764)	378 (351 to 408)	6 888 (4 802 to 8 940)	123 (88 to 161)
Kazakhstan	2 481 (2 060 to 2 917)	15 (12 to 17)	61 047 (56 594 to 65 647)	390 (362 to 417)	20 824 (14 758 to 27 076)	132 (94 to 170)
Kyrgyzstan	659 (548 to 781)	14 (12 to 17)	14 508 (13 405 to 15 679)	379 (352 to 408)	5 108 (3 620 to 6 551)	133 (94 to 171)
Mongolia	281 (233 to 340)	12 (10 to 14)	5 417 (4 949 to 5 864)	291 (269 to 312)	2 109 (1 515 to 2 669)	113 (79 to 143)
Tajikistan	701 (578 to 835)	13 (11 to 15)	14 047 (12 900 to 15 194)	345 (320 to 370)	5 302 (3 798 to 6 795)	130 (92 to 164)
Turkmenistan	469 (393 to 552)	12 (10 to 14)	9 818 (9 032 to 10 570)	319 (296 to 341)	3 610 (2 575 to 4 597)	116 (82 to 149)
Uzbekistan	2 610 (2 167 to 3 104)	12 (10 to 14)	56 015 (51 535 to 60 651)	329 (304 to 353)	19 885 (14 390 to 25 500)	116 (82 to 149)
**Latin America and Caribbean**	**32 730 (27 537 to 38 636)**	**8 (7 to 10)**	**756 199 (700 374 to 819 471)**	**219 (204 to 235)**	**288 300 (206 219 to 366 114)**	**83 (58 to 106)**
**Central Latin America**	**13 137 (11 014 to 15 561)**	**8 (7 to 10)**	**292 651 (264 339 to 332 918)**	**208 (191 to 231)**	**111 785 (78 320 to 145 472)**	**79 (56 to 102)**
Colombia	2 589 (2 148 to 3 104)	8 (7 to 9)	58 525 (53 282 to 64 782)	200 (185 to 219)	21 189 (15 052 to 27 300)	72 (51 to 93)
Costa Rica	182 (148 to 219)	6 (5 to 8)	4 400 (4 005 to 4 830)	167 (154 to 181)	1 466 (1 023 to 1 885)	55 (39 to 71)
El Salvador	497 (353 to 789)	9 (7 to 14)	15 691 (8 961 to 32 584)	316 (196 to 619)	6 828 (2 493 to 12 733)	135 (57 to 241)
Guatemala	692 (520 to 1 001)	8 (6 to 11)	14 990 (11 057 to 25 023)	196 (154 to 301)	6 465 (3 473 to 10 245)	84 (49 to 128)
Honduras	257 (212 to 308)	5 (4 to 6)	4 923 (4 489 to 5 337)	130 (120 to 139)	2 034 (1 449 to 2 572)	53 (38 to 67)
Mexico	7 160 (6 031 to 8 409)	9 (7 to 11)	148 614 (137 871 to 160 283)	213 (200 to 228)	54 735 (38 745 to 69 733)	78 (56 to 100)
Nicaragua	259 (215 to 309)	6 (5 to 8)	11 140 (6 195 to 23 392)	294 (190 to 549)	4 809 (1 785 to 9 289)	122 (55 to 213)
Panama	148 (123 to 179)	6 (5 to 8)	3 651 (3 324 to 3 990)	174 (160 to 188)	1 300 (909 to 1 662)	62 (43 to 80)
Venezuela	1 354 (1 113 to 1 626)	7 (6 to 9)	30 718 (27 916 to 33 639)	185 (171 to 200)	10 873 (7 658 to 14 033)	65 (47 to 84)
**Andean Latin America**	**3 387 (2 801 to 4 045)**	**9 (7 to 10)**	**72 969 (65 809 to 82 078)**	**218 (200 to 241)**	**29 024 (20 581 to 37 232)**	**86 (62 to 109)**
Bolivia	619 (518 to 725)	9 (7 to 10)	11 908 (10 796 to 13 111)	204 (188 to 222)	4 925 (3 521 to 6 238)	84 (61 to 106)
Ecuador	783 (660 to 910)	8 (7 to 9)	18 044 (16 533 to 20 091)	211 (196 to 232)	6 993 (4 931 to 8 886)	82 (58 to 104)
Peru	1 986 (1 610 to 2 472)	9 (7 to 11)	43 017 (37 890 to 49 947)	226 (203 to 255)	16 667 (11 572 to 21 582)	87 (62 to 112)
**Caribbean**	**2 372 (1 988 to 2 816)**	**7 (6 to 8)**	**56 671 (52 681 to 60 556)**	**176 (164 to 187)**	**21 939 (15 701 to 27 978)**	**68 (49 to 87)**

Location	Incidence (95% UI)		Prevalence (95% UI)		YLDs (95% UI)	
	**1990 counts**	**1990 age-standardised rates per 100,000**	**1990 counts**	**1990 age-standardised rates per 100,000**	**1990 counts**	**1990 age-standardised rates per 100,000**
Antigua and Barbuda	4 (3 to 5)	7 (6 to 8)	116 (106 to 124)	201 (186 to 216)	39 (27 to 51)	68 (48 to 87)
The Bahamas	18 (15 to 22)	7 (6 to 9)	446 (414 to 476)	190 (178 to 202)	152 (109 to 195)	64 (45 to 83)
Barbados	15 (13 to 18)	6 (5 to 7)	455 (420 to 487)	176 (163 to 188)	152 (107 to 197)	59 (41 to 75)
Belize	14 (11 to 17)	7 (6 to 8)	258 (238 to 280)	172 (160 to 184)	95 (68 to 120)	63 (44 to 80)
Bermuda	4 (3 to 5)	8 (6 to 9)	106 (98 to 113)	208 (194 to 222)	34 (24 to 44)	67 (47 to 87)
Cuba	767 (635 to 922)	7 (6 to 9)	20 877 (19 437 to 22 295)	200 (187 to 213)	6 715 (4 660 to 8 692)	64 (45 to 83)
Dominica	4 (3 to 5)	6 (5 to 7)	98 (91 to 105)	159 (148 to 170)	34 (24 to 44)	56 (39 to 72)
Dominican Republic	418 (350 to 495)	6 (5 to 7)	9 842 (9 104 to 10 596)	164 (153 to 176)	3 807 (2 690 to 4 875)	63 (46 to 80)
Grenada	7 (5 to 8)	7 (5 to 8)	146 (135 to 157)	176 (163 to 189)	53 (38 to 68)	64 (45 to 81)
Guyana	52 (44 to 61)	7 (6 to 9)	1 067 (988 to 1 150)	164 (153 to 175)	412 (296 to 524)	63 (45 to 80)
Haiti	487 (409 to 576)	7 (6 to 8)	8 429 (7 779 to 9 117)	138 (128 to 147)	3 696 (2 645 to 4 625)	60 (43 to 75)
Jamaica	130 (108 to 155)	5 (4 to 6)	3 239 (2 980 to 3 484)	155 (143 to 165)	1 147 (800 to 1 485)	55 (39 to 70)
Puerto Rico	241 (199 to 288)	7 (6 to 8)	6 430 (5 937 to 6 912)	186 (172 to 200)	2 005 (1 405 to 2 591)	58 (41 to 76)
Saint Lucia	9 (8 to 11)	6 (5 to 7)	209 (194 to 224)	171 (160 to 183)	75 (53 to 96)	61 (43 to 79)
Saint Vincent and the Grenadines	7 (6 to 9)	7 (6 to 8)	165 (152 to 177)	177 (165 to 189)	59 (41 to 75)	63 (45 to 81)
Suriname	26 (22 to 31)	6 (5 to 8)	649 (593 to 731)	178 (164 to 197)	248 (179 to 322)	68 (48 to 88)
Trinidad and Tobago	83 (69 to 102)	7 (6 to 8)	1 881 (1 738 to 2 025)	166 (154 to 178)	663 (470 to 848)	58 (41 to 75)
Virgin Islands, U.S.	7 (6 to 8)	7 (6 to 8)	188 (175 to 201)	186 (173 to 198)	63 (44 to 81)	63 (44 to 80)
**Tropical Latin America**	**13 833 (11 519 to 16 509)**	**9 (7 to 11)**	**333 908 (305 408 to 362 022)**	**241 (222 to 259)**	**122 522 (87 708 to 156 079)**	**88 (63 to 112)**
Brazil	13 571 (11 300 to 16 190)	9 (8 to 11)	327 757 (299 800 to 355 332)	242 (224 to 260)	119 716 (84 566 to 153 109)	88 (62 to 113)
Paraguay	262 (214 to 318)	6 (5 to 7)	6 151 (5 532 to 6 806)	180 (163 to 197)	2 310 (1 637 to 2 968)	67 (49 to 86)
**Southeast Asia, East Asia, and Oceania**	**108 027 (92 365 to 127 667)**	**7 (6 to 8)**	**2 734 809 (2 516 137 to 2 996 879)**	**177 (164 to 192)**	**1 074 827 (754 250 to 1 382 918)**	**69 (49 to 88)**
**East Asia**	**77 464 (66 489 to 91 858)**	**7 (6 to 8)**	**2 043 621 (1 896 529 to 2 204 399)**	**180 (169 to 194)**	**765 036 (542 391 to 975 630)**	**67 (48 to 86)**
China	75 322 (64 590 to 89 330)	7 (6 to 8)	1 983 131 (1 839 640 to 2 139 561)	181 (169 to 195)	744 414 (530 352 to 946 424)	68 (48 to 87)
North Korea	1 007 (859 to 1 188)	5 (4 to 5)	26 033 (24 291 to 27 814)	133 (124 to 141)	9 316 (6 563 to 11 931)	47 (33 to 60)
Taiwan (Province of China)	1 135 (953 to 1 352)	6 (5 to 7)	34 457 (32 129 to 36 744)	176 (165 to 187)	11 387 (8 064 to 14 648)	58 (40 to 75)
**Southeast Asia**	**30 143 (24 789 to 38 740)**	**7 (6 to 8)**	**684 245 (587 277 to 900 284)**	**166 (146 to 213)**	**287 411 (188 716 to 398 599)**	**70 (47 to 96)**
Cambodia	660 (458 to 1 093)	8 (5 to 12)	35 702 (13 812 to 95 730)	448 (184 to 1 141)	17 648 (3 237 to 40 047)	221 (44 to 488)
Indonesia	10 043 (8 596 to 11 662)	6 (5 to 7)	251 742 (223 150 to 307 850)	154 (139 to 185)	104 940 (72 274 to 138 184)	64 (45 to 84)
Laos	358 (229 to 683)	8 (5 to 16)	3 922 (3 562 to 4 431)	110 (102 to 122)	1 722 (1 197 to 2 183)	48 (35 to 61)
Malaysia	1 114 (943 to 1 309)	6 (5 to 7)	27 133 (25 411 to 29 200)	172 (163 to 184)	10 080 (7 093 to 12 736)	64 (45 to 81)
Maldives	11 (9 to 12)	6 (5 to 6)	237 (220 to 256)	140 (132 to 150)	91 (64 to 117)	54 (38 to 69)
Mauritius	54 (46 to 63)	5 (4 to 6)	1 485 (1 383 to 1 599)	145 (136 to 156)	511 (360 to 656)	50 (35 to 63)
Myanmar	2 068 (1 754 to 2 428)	5 (4 to 6)	58 768 (43 252 to 99 717)	151 (115 to 245)	27 082 (15 236 to 43 807)	69 (40 to 106)
Philippines	4 494 (3 422 to 6 255)	7 (5 to 10)	90 154 (71 360 to 135 233)	170 (139 to 246)	36 588 (21 704 to 54 829)	69 (43 to 101)
Sri Lanka	3 902 (1 758 to 8 973)	22 (10 to 49)	36 294 (29 970 to 50 872)	225 (190 to 306)	13 989 (8 794 to 20 879)	86 (57 to 124)
Seychelles	4 (4 to 5)	6 (5 to 7)	102 (93 to 114)	164 (152 to 181)	38 (27 to 49)	60 (42 to 77)
Thailand	3 833 (3 226 to 4 539)	7 (6 to 8)	95 839 (89 800 to 102 329)	185 (174 to 196)	35 582 (25 112 to 45 205)	68 (49 to 87)
Timor-Leste	159 (60 to 408)	20 (8 to 49)	3 343 (1 272 to 8 537)	479 (190 to 1 177)	1 628 (282 to 3 583)	235 (48 to 509)
Vietnam	3 401 (2 916 to 3 957)	5 (4 to 6)	78 574 (72 888 to 84 474)	134 (126 to 143)	30 720 (21 754 to 38 831)	52 (37 to 66)
**Oceania**	**420 (352 to 510)**	**6 (5 to 8)**	**6 943 (6 476 to 7 446)**	**123 (116 to 131)**	**2 859 (2 040 to 3 599)**	**50 (36 to 64)**
American Samoa	4 (3 to 4)	7 (6 to 9)	76 (71 to 82)	191 (180 to 204)	28 (20 to 35)	69 (49 to 88)
Federated States of Micronesia	6 (5 to 7)	6 (5 to 7)	106 (98 to 114)	134 (125 to 142)	44 (31 to 55)	55 (39 to 68)
Fiji	38 (32 to 44)	5 (4 to 6)	827 (768 to 893)	127 (119 to 136)	312 (219 to 396)	47 (33 to 60)
Guam	9 (7 to 10)	7 (6 to 8)	221 (206 to 237)	183 (172 to 194)	72 (51 to 93)	59 (42 to 77)
Kiribati	4 (3 to 4)	5 (4 to 6)	65 (60 to 69)	101 (95 to 108)	28 (20 to 35)	43 (31 to 54)
Marshall Islands	3 (2 to 3)	6 (5 to 7)	50 (46 to 54)	133 (124 to 141)	20 (14 to 25)	52 (37 to 66)
Northern Mariana Islands	4 (3 to 4)	8 (6 to 9)	93 (87 to 100)	219 (206 to 232)	31 (22 to 40)	71 (51 to 92)

Location	Incidence (95% UI)		Prevalence (95% UI)		YLDs (95% UI)	
	1990 counts	1990 age-standardised rates per 100,000	1990 counts	1990 age-standardised rates per 100,000	1990 counts	1990 age-standardised rates per 100,000
Papua New Guinea	280 (231 to 350)	7 (6 to 8)	4 118 (3 833 to 4 429)	116 (109 to 123)	1 741 (1 253 to 2 191)	49 (35 to 61)
Samoa	10 (8 to 11)	6 (5 to 7)	200 (186 to 215)	147 (138 to 157)	77 (55 to 99)	57 (41 to 72)
Solomon Islands	17 (14 to 20)	6 (5 to 7)	323 (297 to 353)	125 (116 to 135)	134 (96 to 169)	51 (36 to 64)
Tonga	6 (5 to 7)	6 (5 to 7)	119 (110 to 127)	148 (139 to 158)	45 (32 to 58)	56 (40 to 71)
Vanuatu	8 (7 to 10)	5 (4 to 6)	151 (140 to 164)	120 (112 to 129)	62 (44 to 78)	49 (35 to 62)
**North Africa and Middle East**	**39 022 (31 286 to 49 505)**	**11 (9 to 14)**	**1 349 057 (816 813 to 2 808 948)**	**430 (282 to 810)**	**597 913 (232 320 to 1 090 466)**	**188 (83 to 334)**
**North Africa and Middle East**	**39 022 (31 286 to 49 505)**	**11 (9 to 14)**	**1 349 057 (816 813 to 2 808 948)**	**430 (282 to 810)**	**597 913 (232 320 to 1 090 466)**	**188 (83 to 334)**
Afghanistan	1 910 (1 196 to 3 445)	14 (9 to 25)	200 949 (39 507 to 600 088)	1 622 (346 to 4 790)	106 737 (7 289 to 250 992)	850 (73 to 1 988)
Algeria	2 316 (1 959 to 2 727)	9 (7 to 10)	50 738 (46 797 to 55 133)	245 (229 to 263)	19 006 (13 542 to 24 115)	92 (65 to 117)
Bahrain	49 (42 to 59)	9 (8 to 11)	1 267 (1 183 to 1 359)	274 (257 to 292)	454 (319 to 584)	98 (69 to 125)
Egypt	3 894 (3 313 to 4 532)	6 (5 to 7)	92 794 (84 688 to 105 532)	189 (174 to 210)	37 240 (25 956 to 48 047)	75 (53 to 95)
Iran	11 293 (6 779 to 19 058)	21 (12 to 37)	300 592 (157 137 to 672 896)	584 (339 to 1 210)	126 700 (41 221 to 249 106)	238 (91 to 450)
Iraq	2 114 (1 650 to 2 906)	11 (9 to 15)	252 872 (80 384 to 722 959)	1 441 (508 to 4 132)	117 551 (12 031 to 265 507)	666 (84 to 1 548)
Jordan	310 (263 to 363)	9 (7 to 10)	6 512 (6 031 to 7 101)	253 (237 to 271)	2 324 (1 668 to 2 973)	90 (64 to 115)
Kuwait	602 (244 to 1 603)	27 (11 to 69)	5 788 (5 350 to 6 373)	322 (301 to 349)	1 868 (1 314 to 2 425)	103 (71 to 136)
Lebanon	1 111 (423 to 2 811)	37 (15 to 92)	60 229 (17 691 to 175 037)	2 158 (657 to 6 093)	26 925 (1 997 to 65 732)	927 (78 to 2 182)
Libya	370 (312 to 438)	8 (7 to 10)	11 323 (9 143 to 16 183)	301 (255 to 398)	4 304 (2 719 to 6 337)	111 (74 to 155)
Morocco	2 041 (1 729 to 2 398)	8 (6 to 9)	47 724 (43 606 to 54 137)	221 (204 to 244)	18 782 (13 443 to 24 390)	86 (61 to 111)
Palestine	281 (175 to 558)	11 (7 to 22)	13 607 (4 986 to 35 896)	705 (290 to 1 792)	6 020 (1 085 to 13 920)	301 (70 to 672)
Oman	197 (163 to 240)	10 (8 to 13)	4 250 (3 940 to 4 568)	296 (278 to 317)	1 495 (1 066 to 1 921)	104 (73 to 133)
Qatar	58 (49 to 70)	12 (10 to 14)	1 651 (1 539 to 1 761)	363 (341 to 386)	562 (398 to 721)	123 (87 to 159)
Saudi Arabia	1 844 (1 560 to 2 176)	11 (9 to 13)	39 682 (37 001 to 42 476)	317 (298 to 337)	14 221 (10 038 to 18 297)	113 (80 to 144)
Sudan	1 971 (1 470 to 2 970)	9 (7 to 14)	41 173 (30 370 to 68 710)	234 (184 to 351)	17 882 (9 960 to 28 470)	101 (62 to 152)
Syria	918 (779 to 1 075)	7 (6 to 8)	26 005 (19 190 to 43 677)	255 (201 to 401)	10 357 (5 788 to 16 631)	101 (59 to 159)
Tunisia	681 (576 to 798)	8 (7 to 10)	16 852 (15 667 to 18 174)	236 (220 to 253)	6 135 (4 331 to 7 940)	86 (60 to 110)
Turkey	5 758 (4 888 to 6 746)	10 (9 to 12)	142 726 (131 576 to 155 056)	294 (273 to 316)	53 449 (37 951 to 67 809)	109 (77 to 139)
United Arab Emirates	231 (193 to 275)	12 (10 to 14)	5 963 (5 507 to 6 375)	348 (324 to 370)	2 144 (1 518 to 2 758)	124 (87 to 161)
Yemen	1 050 (888 to 1 229)	8 (7 to 10)	25 443 (19 629 to 40 101)	262 (216 to 376)	11 318 (6 573 to 17 407)	115 (74 to 165)
**South Asia**	**117 189 (99 031 to 138 109)**	**12 (10 to 14)**	**1 988 618 (1 865 624 to 2 128 475)**	**213 (201 to 226)**	**837 245 (602 368 to 1 050 465)**	**89 (64 to 112)**
**South Asia**	**117 189 (99 031 to 138 109)**	**12 (10 to 14)**	**1 988 618 (1 865 624 to 2 128 475)**	**213 (201 to 226)**	**837 245 (602 368 to 1 050 465)**	**89 (64 to 112)**
Bangladesh	8 809 (7 481 to 10 311)	9 (8 to 10)	156 485 (144 946 to 169 570)	180 (169 to 193)	68 625 (49 053 to 86 595)	79 (56 to 98)
Bhutan	53 (45 to 63)	11 (9 to 13)	917 (858 to 986)	218 (204 to 233)	395 (283 to 497)	94 (67 to 117)
India	97 000 (81 856 to 114 695)	12 (10 to 15)	1 633 839 (1 532 078 to 1 749 156)	219 (207 to 233)	684 349 (487 896 to 852 830)	91 (65 to 116)
Nepal	1 744 (1 468 to 2 045)	10 (8 to 12)	28 439 (26 541 to 30 594)	187 (176 to 199)	12 161 (8 685 to 15 301)	80 (57 to 100)
Pakistan	9 582 (8 140 to 11 127)	9 (8 to 10)	168 937 (157 942 to 182 483)	196 (185 to 210)	69 808 (49 219 to 88 147)	81 (58 to 103)
**Sub-Saharan Africa**	**62 577 (44 733 to 100 053)**	**13 (9 to 19)**	**840 100 (718 645 to 1 087 391)**	**206 (181 to 257)**	**373 088 (243 208 to 504 507)**	**92 (63 to 122)**
**Southern sub-Saharan Africa**	**5 428 (4 334 to 7 142)**	**10 (8 to 14)**	**101 689 (93 822 to 110 791)**	**221 (207 to 239)**	**39 511 (27 940 to 50 852)**	**85 (60 to 108)**
Botswana	103 (87 to 122)	8 (6 to 9)	2 050 (1 899 to 2 203)	179 (168 to 190)	797 (559 to 1 014)	69 (49 to 89)
Lesotho	109 (91 to 128)	7 (6 to 8)	1 968 (1 823 to 2 107)	148 (139 to 157)	797 (565 to 1 013)	60 (42 to 75)
Namibia	104 (88 to 124)	7 (6 to 9)	1 951 (1 811 to 2 103)	166 (156 to 177)	804 (575 to 1 022)	68 (49 to 85)
South Africa	4 363 (3 414 to 5 968)	12 (9 to 16)	82 640 (75 874 to 91 280)	247 (229 to 269)	31 876 (22 761 to 41 141)	94 (68 to 120)
Swaziland	63 (53 to 75)	8 (6 to 9)	1 168 (1 083 to 1 248)	174 (164 to 184)	469 (333 to 597)	70 (50 to 88)
Zimbabwe	685 (581 to 796)	7 (6 to 8)	11 912 (11 044 to 12 859)	139 (131 to 148)	4 605 (3 259 to 5 896)	53 (38 to 68)
**Western sub-Saharan Africa**	**18 364 (15 298 to 22 224)**	**9 (8 to 11)**	**292 457 (269 831 to 315 590)**	**184 (171 to 196)**	**124 324 (89 107 to 156 742)**	**78 (56 to 98)**
Benin	419 (355 to 490)	8 (7 to 10)	7 070 (6 532 to 7 601)	177 (165 to 189)	3 049 (2 195 to 3 863)	76 (54 to 96)
Burkina Faso	778 (654 to 910)	9 (7 to 10)	11 472 (10 579 to 12 363)	161 (150 to 172)	5 024 (3 609 to 6 261)	70 (50 to 88)
Cameroon	990 (837 to 1 160)	8 (7 to 10)	17 521 (16 178 to 18 982)	182 (170 to 195)	7 391 (5 207 to 9 413)	77 (56 to 97)
Cape Verde	26 (22 to 31)	8 (6 to 9)	516 (477 to 557)	193 (181 to 207)	199 (143 to 252)	74 (52 to 94)

Location	Incidence (95% UI)		Prevalence (95% UI)		YLDs (95% UI)	
	1990 counts	1990 age-standardised rates per 100,000	1990 counts	1990 age-standardised rates per 100,000	1990 counts	1990 age-standardised rates per 100,000
Chad	768 (495 to 1 357)	13 (8 to 22)	12 059 (8 748 to 19 658)	244 (185 to 377)	5 605 (3 038 to 8 808)	110 (66 to 166)
Cote d'Ivoire	1 091 (925 to 1 279)	10 (8 to 11)	18 239 (16 907 to 19 571)	183 (172 to 195)	7 829 (5 638 to 9 880)	78 (56 to 99)
The Gambia	77 (65 to 90)	9 (7 to 10)	1 491 (1 309 to 1 853)	211 (188 to 256)	629 (436 to 840)	88 (62 to 115)
Ghana	1 111 (941 to 1 302)	8 (7 to 9)	19 462 (17 983 to 21 037)	164 (153 to 175)	8 102 (5 716 to 10 280)	68 (48 to 86)
Guinea	510 (432 to 596)	9 (7 to 10)	8 907 (8 246 to 9 604)	180 (168 to 193)	3 889 (2 777 to 4 889)	78 (57 to 99)
Guinea-Bissau	96 (81 to 111)	10 (8 to 11)	1 489 (1 385 to 1 595)	170 (160 to 181)	666 (477 to 834)	76 (54 to 95)
Liberia	1 213 (398 to 3 178)	52 (18 to 137)	3 382 (2 906 to 4 382)	191 (170 to 235)	1 489 (1 026 to 2 029)	84 (57 to 112)
Mali	832 (684 to 1 032)	9 (8 to 11)	11 283 (10 433 to 12 184)	164 (153 to 175)	4 944 (3 514 to 6 185)	71 (51 to 89)
Mauritania	244 (166 to 435)	12 (9 to 21)	3 112 (2 867 to 3 367)	192 (179 to 205)	1 316 (949 to 1 659)	81 (58 to 101)
Niger	756 (629 to 908)	9 (8 to 11)	10 742 (9 943 to 11 578)	173 (162 to 185)	4 737 (3 389 to 6 006)	76 (55 to 96)
Nigeria	8 176 (6 910 to 9 576)	9 (7 to 10)	143 938 (132 838 to 155 791)	189 (176 to 202)	59 186 (41 448 to 74 693)	77 (56 to 99)
Sao Tome and Principe	10 (9 to 12)	9 (7 to 10)	189 (175 to 204)	215 (201 to 229)	79 (56 to 100)	89 (64 to 113)
Senegal	637 (541 to 749)	9 (7 to 10)	10 770 (9 928 to 11 634)	181 (169 to 193)	4 568 (3 231 to 5 764)	77 (54 to 97)
Sierra Leone	325 (275 to 380)	9 (7 to 10)	5 583 (5 151 to 6 018)	174 (162 to 186)	2 395 (1 720 to 3 015)	74 (53 to 94)
Togo	304 (257 to 355)	8 (7 to 10)	5 225 (4 789 to 5 635)	173 (161 to 184)	2 225 (1 582 to 2 803)	73 (52 to 92)
**Eastern sub-Saharan Africa**	**33 436 (19 734 to 65 893)**	**18 (11 to 33)**	**359 311 (273 602 to 559 097)**	**227 (180 to 337)**	**169 262 (99 652 to 254 398)**	**104 (64 to 154)**
Burundi	495 (417 to 582)	9 (8 to 11)	6 881 (6 340 to 7 403)	150 (140 to 159)	3 057 (2 167 to 3 797)	66 (48 to 82)
Comoros	41 (34 to 48)	11 (9 to 13)	610 (569 to 653)	191 (180 to 203)	269 (194 to 336)	84 (60 to 107)
Djibouti	74 (57 to 109)	12 (9 to 17)	1 048 (972 to 1 127)	224 (210 to 238)	444 (319 to 559)	95 (68 to 119)
Eritrea	277 (236 to 324)	10 (8 to 11)	4 206 (3 887 to 4 536)	167 (156 to 178)	1 893 (1 363 to 2 375)	75 (53 to 93)
Ethiopia	18 110 (7 388 to 43 415)	36 (16 to 85)	107 469 (74 925 to 195 013)	254 (184 to 434)	51 709 (26 334 to 86 810)	123 (67 to 195)
Kenya	1 896 (1 609 to 2 215)	8 (7 to 10)	32 524 (30 042 to 35 014)	183 (172 to 195)	12 979 (9 304 to 16 457)	73 (52 to 92)
Madagascar	982 (827 to 1 150)	9 (7 to 10)	16 160 (14 917 to 17 386)	174 (163 to 185)	6 932 (4 945 to 8 674)	74 (53 to 94)
Malawi	802 (674 to 941)	9 (7 to 10)	11 930 (10 912 to 12 934)	153 (142 to 164)	5 140 (3 681 to 6 511)	66 (47 to 83)
Mozambique	1 559 (1 093 to 2 617)	11 (8 to 18)	45 723 (21 156 to 106 652)	379 (190 to 856)	22 846 (6 593 to 46 263)	190 (60 to 380)
Rwanda	1 417 (766 to 2 956)	19 (11 to 39)	9 797 (8 957 to 10 743)	168 (156 to 181)	4 326 (3 121 to 5 433)	74 (54 to 93)
Somalia	1 312 (697 to 2 874)	21 (12 to 43)	13 638 (9 909 to 23 383)	247 (189 to 392)	6 484 (3 615 to 10 537)	116 (71 to 177)
South Sudan	1 724 (764 to 4 038)	28 (13 to 64)	12 944 (10 348 to 19 142)	264 (219 to 370)	5 803 (3 564 to 8 597)	117 (76 to 165)
Tanzania	2 322 (1 963 to 2 703)	9 (8 to 11)	36 872 (33 981 to 39 711)	180 (168 to 192)	15 715 (11 147 to 19 627)	76 (55 to 96)
Uganda	1 632 (1 319 to 2 095)	9 (8 to 12)	47 763 (22 079 to 108 126)	315 (153 to 703)	22 813 (7 342 to 44 718)	149 (51 to 290)
Zambia	777 (656 to 907)	9 (8 to 11)	11 562 (10 607 to 12 481)	176 (163 to 187)	4 906 (3 541 to 6 159)	74 (53 to 93)
**Central sub-Saharan Africa**	**5 348 (4 286 to 7 008)**	**10 (8 to 13)**	**86 643 (72 235 to 119 351)**	**199 (171 to 262)**	**38 792 (25 688 to 55 484)**	**88 (59 to 119)**
Angola	1 765 (1 104 to 3 256)	16 (10 to 28)	30 655 (17 022 to 62 625)	322 (194 to 629)	14 526 (6 133 to 27 034)	154 (70 to 275)
Central African Republic	218 (184 to 255)	8 (6 to 9)	3 273 (3 022 to 3 539)	128 (119 to 138)	1 434 (1 040 to 1 805)	56 (41 to 70)
Congo	216 (182 to 252)	9 (8 to 11)	3 373 (3 133 to 3 635)	166 (155 to 177)	1 435 (1 039 to 1 800)	70 (50 to 88)
DR Congo	3 019 (2 555 to 3 493)	9 (8 to 11)	47 097 (43 704 to 50 677)	170 (159 to 181)	20 038 (14 384 to 25 347)	72 (52 to 90)
Equatorial Guinea	33 (28 to 39)	9 (8 to 11)	497 (462 to 532)	152 (142 to 162)	220 (160 to 276)	67 (48 to 85)
Gabon	97 (82 to 115)	11 (9 to 13)	1 748 (1 640 to 1 866)	218 (205 to 231)	715 (514 to 899)	89 (64 to 112)

Appendix Table 8: Incidence, prevalence, and YLDs for 2016 and percentage change of rates by age group for SCI

Age group	Incidence (95% UI)			Prevalence (95% UI)			YLDs (95% UI)		
	2016 counts	2016 rates per 100,000	Percentage change in rates between 1990 and 2016	2016 counts	2016 rates per 100,000	Percentage change in rates between 1990 and 2016	2016 counts	2016 rates per 100,000	Percentage change in rates between 1990 and 2016
Early Neonatal	41<br>(16 to 110)	2<br>(1 to 5)	26.1<br>(-14.2 to 76.4)	9<br>(6 to 14)	0<br>(0 to 1)	-13.5<br>(-25.0 to 9.7)	5<br>(3 to 7)	0<br>(0 to 0)	-18.7<br>(-32.7 to -5.2)
Late Neonatal	210<br>(108 to 468)	3<br>(1 to 6)	-0.8<br>(-25.3 to 33.7)	109<br>(81 to 142)	1<br>(1 to 2)	-19.0<br>(-23.8 to -11.5)	41<br>(29 to 55)	1<br>(0 to 1)	-25.9<br>(-31.1 to -20.3)
Post Neonatal	23 818<br>(19 573 to 28 619)	20<br>(17 to 25)	-21.8<br>(-24.7 to -18.0)	14 227<br>(11 871 to 16 743)	12<br>(10 to 14)	-3.5<br>(-6.1 to -0.5)	5 421<br>(3 975 to 6 849)	5<br>(3 to 6)	-13.0<br>(-15.6 to -10.7)
1 to 4	75 842<br>(57 551 to 104 764)	15<br>(11 to 21)	-0.4<br>(-8.8 to 19.7)	268 278<br>(229 239 to 328 599)	53<br>(45 to 65)	-7.3<br>(-14.0 to -3.2)	96 832<br>(65 475 to 130 154)	19<br>(13 to 26)	-16.9<br>(-20.2 to -13.3)
5 to 9	71 813<br>(54 098 to 97 346)	11<br>(8 to 15)	0.2<br>(-7.6 to 17.5)	677 497<br>(582 672 to 806 621)	106<br>(91 to 126)	-16.7<br>(-31.8 to -7.2)	241 122<br>(167 035 to 326 690)	38<br>(26 to 51)	-26.9<br>(-37.7 to -14.2)
10 to 14	65 062<br>(48 223 to 89 462)	11<br>(8 to 15)	-1.1<br>(-9.3 to 14.9)	974 402<br>(855 433 to 1 122 819)	159<br>(140 to 184)	-15.8<br>(-30.0 to -7.9)	347 204<br>(238 687 to 454 697)	57<br>(39 to 74)	-26.4<br>(-36.1 to -15.5)
15 to 19	64 615<br>(49 025 to 87 425)	11<br>(8 to 15)	-0.8<br>(-9.0 to 14.0)	1 264 911<br>(1 131 926 to 1 429 050)	214<br>(192 to 242)	-10.3<br>(-18.2 to -5.7)	448 491<br>(316 856 to 580 212)	76<br>(54 to 98)	-20.3<br>(-25.4 to -14.8)
20 to 24	68 781<br>(53 382 to 94 537)	11<br>(9 to 16)	-2.1<br>(-10.4 to 11.1)	1 627 658<br>(1 460 186 to 1 822 436)	271<br>(243 to 303)	-8.4<br>(-13.5 to -5.2)	580 190<br>(409 359 to 751 498)	97<br>(68 to 125)	-18.3<br>(-21.6 to -14.9)
25 to 29	64 778<br>(50 101 to 82 238)	11<br>(8 to 13)	-6.2<br>(-12.4 to 2.8)	2 008 895<br>(1 815 414 to 2 251 216)	327<br>(296 to 367)	-10.1<br>(-13.6 to -6.9)	714 781<br>(493 049 to 931 428)	116<br>(80 to 152)	-19.3<br>(-21.4 to -17.4)
30 to 34	58 701<br>(47 053 to 73 060)	10<br>(8 to 13)	-8.2<br>(-13.5 to -0.9)	2 229 369<br>(1 985 612 to 2 670 176)	397<br>(353 to 475)	-6.1<br>(-10.3 to 3.7)	799 021<br>(541 428 to 1 080 248)	142<br>(96 to 192)	-14.6<br>(-20.8 to -7.2)
35 to 39	52 641<br>(41 911 to 66 150)	10<br>(8 to 13)	-6.8<br>(-11.8 to -1.1)	2 313 769<br>(2 068 252 to 2 807 916)	459<br>(410 to 557)	-1.5<br>(-7.5 to 12.2)	832 239<br>(572 035 to 1 113 032)	165<br>(114 to 221)	-9.8<br>(-19.0 to 1.0)
40 to 44	51 721<br>(40 900 to 64 449)	11<br>(8 to 13)	-7.5<br>(-11.3 to -3.3)	2 417 534<br>(2 205 472 to 2 774 009)	497<br>(453 to 570)	-5.5<br>(-10.0 to 4.0)	861 798<br>(600 849 to 1 135 818)	177<br>(123 to 233)	-13.8<br>(-20.8 to -6.0)
45 to 49	52 431<br>(41 434 to 65 732)	11<br>(9 to 14)	-3.8<br>(-7.9 to 0.3)	2 491 324<br>(2 300 047 to 2 788 448)	540<br>(499 to 605)	-1.8<br>(-6.0 to 6.4)	881 733<br>(624 533 to 1 152 033)	191<br>(135 to 250)	-10.7<br>(-17.1 to -3.7)
50 to 54	52 627<br>(40 982 to 67 999)	13<br>(10 to 17)	0.1<br>(-4.1 to 4.6)	2 419 930<br>(2 243 641 to 2 693 499)	589<br>(546 to 655)	2.7<br>(-1.5 to 11.4)	851 024<br>(602 709 to 1 097 678)	207<br>(147 to 267)	-6.6<br>(-12.7 to 0.2)
55 to 59	47 002<br>(36 597 to 61 603)	14<br>(11 to 18)	4.3<br>(-0.1 to 8.7)	2 148 513<br>(1 993 797 to 2 356 200)	620<br>(575 to 680)	7.7<br>(3.9 to 14.9)	749 372<br>(533 325 to 972 143)	216<br>(154 to 281)	-2.0<br>(-7.4 to 3.8)
60 to 64	41 436<br>(32 659 to 53 657)	14<br>(11 to 18)	0.1<br>(-4.0 to 4.2)	1 899 966<br>(1 778 987 to 2 058 267)	633<br>(592 to 685)	7.9<br>(4.6 to 13.6)	654 739<br>(464 032 to 855 009)	218<br>(155 to 285)	-2.3<br>(-6.9 to 2.4)
65 to 69	33 674<br>(26 443 to 44 460)	15<br>(12 to 20)	0.8<br>(-3.3 to 4.8)	1 539 333<br>(1 430 495 to 1 657 183)	682<br>(633 to 734)	12.3<br>(9.3 to 17.0)	528 313<br>(374 121 to 678 082)	234<br>(166 to 300)	1.5<br>(-2.3 to 5.4)
70 to 74	27 285<br>(20 520 to 37 422)	17<br>(13 to 24)	2.1<br>(-3.2 to 7.6)	1 100 449<br>(1 014 574 to 1 187 150)	700<br>(645 to 755)	22.4<br>(19.2 to 26.7)	376 923<br>(268 167 to 485 511)	240<br>(171 to 309)	10.5<br>(6.8 to 14.1)
75 to 79	25 551<br>(19 145 to 34 312)	22<br>(16 to 29)	-8.3<br>(-14.7 to -2.2)	806 133<br>(734 135 to 891 218)	692<br>(630 to 765)	17.5<br>(14.1 to 21.4)	273 530<br>(191 726 to 354 153)	235<br>(164 to 304)	5.7<br>(2.1 to 9.4)
80 to 84	23 482<br>(17 352 to 32 192)	32<br>(24 to 44)	-13.2<br>(-18.9 to -6.5)	490 431<br>(429 150 to 561 018)	676<br>(591 to 773)	20.9<br>(16.1 to 26.3)	165 024<br>(117 344 to 215 233)	227<br>(162 to 297)	8.4<br>(4.5 to 12.0)
85 to 89	18 682<br>(13 299 to 26 188)	47<br>(33 to 66)	-13.6<br>(-20.9 to -7.1)	248 266<br>(206 691 to 295 478)	623<br>(519 to 742)	27.7<br>(21.7 to 34.1)	82 995<br>(57 336 to 109 506)	208<br>(144 to 275)	13.6<br>(9.4 to 18.2)
90 to 94	11 124<br>(7 820 to 15 781)	75<br>(53 to 107)	-0.8<br>(-7.6 to 5.6)	85 490<br>(64 083 to 110 013)	579<br>(434 to 745)	36.0<br>(28.8 to 43.9)	28 242<br>(19 222 to 38 405)	191<br>(130 to 260)	19.9<br>(14.9 to 26.5)
95 plus	3 635<br>(2 424 to 5 459)	110<br>(74 to 166)	9.9<br>(-0.1 to 19.2)	16 013<br>(11 106 to 22 813)	486<br>(337 to 692)	33.5<br>(27.3 to 41.1)	5 272<br>(3 362 to 7 364)	160<br>(102 to 223)	16.9<br>(11.8 to 23.2)

Appendix Table 9: YLDs for 2016 and percentage change of age-standardized rates by location for TBI			
Location	YLDs (95% UI)		
	2016 counts	2016 age-standardised rates per 100,000	Percentage change in age-standardised rates between 1990 and 2016
**Global**	**8 122 386 (6 025 047 to 10 376 022)**	**111 (82 to 141)**	**8.5 (7.6 to 9.3)**
**High SDI**	**1 215 736 (926 162 to 1 517 290)**	**95 (72 to 119)**	**-7.7 (-8.0 to -7.4)**
**High-middle SDI**	**2 023 554 (1 476 556 to 2 612 788)**	**157 (114 to 196)**	**-3.9 (-4.6 to -3.4)**
**Middle SDI**	**2 504 676 (1 872 290 to 3 160 845)**	**105 (77 to 133)**	**29.6 (29.2 to 30.0)**
**Low-middle SDI**	**2 029 121 (1 464 165 to 2 559 966)**	**111 (84 to 138)**	**17.0 (16.3 to 17.5)**
**Low SDI**	**536 314 (404 888 to 665 010)**	**100 (76 to 124)**	**3.8 (3.3 to 4.2)**
**High-income**	**1 024 854 (792 004 to 1 256 091)**	**78 (58 to 98)**	**-10.4 (-11.1 to -9.7)**
**High-income North America**	**364 482 (284 040 to 454 191)**	**85 (67 to 106)**	**-7.9 (-8.4 to -7.2)**
Canada	35 575 (27 392 to 43 969)	80 (62 to 99)	-10.4 (-11.9 to -9.1)
Greenland	39 (31 to 49)	73 (56 to 90)	-12.7 (-14.2 to -11.5)
United States	328 736 (250 176 to 401 975)	86 (65 to 106)	-7.5 (-8.1 to -6.7)
**Australasia**	**25 346 (19 297 to 31 558)**	**76 (57 to 93)**	**-13.4 (-14.8 to -12.0)**
Australia	21 297 (16 389 to 26 077)	76 (58 to 91)	-12.6 (-14.1 to -11.0)
New Zealand	4 049 (3 126 to 5 028)	77 (60 to 94)	-17.1 (-18.4 to -15.1)
**High-income Asia-Pacific**	**173 676 (132 098 to 214 091)**	**70 (53 to 88)**	**-14.9 (-15.7 to -14.1)**
Brunei	385 (292 to 480)	96 (71 to 120)	-20.3 (-21.0 to -19.6)
Japan	122 681 (94 271 to 153 513)	68 (53 to 83)	-14.1 (-15.1 to -13.2)
Singapore	3 420 (2 626 to 4 186)	74 (56 to 91)	-0.2 (-1.4 to 0.8)
South Korea	47 185 (35 246 to 57 791)	76 (56 to 94)	-18.7 (-19.3 to -18.1)
**Western Europe**	**424 043 (314 838 to 527 544)**	**78 (59 to 97)**	**-12.2 (-12.8 to -11.8)**
Andorra	81 (61 to 101)	80 (59 to 101)	6.7 (6.2 to 7.2)
Austria	9 346 (7 160 to 11 514)	84 (64 to 106)	-16.6 (-16.8 to -16.5)
Belgium	12 675 (9 856 to 15 542)	89 (68 to 110)	-10.0 (-11.9 to -8.0)
Cyprus	952 (733 to 1 164)	90 (68 to 112)	-7.7 (-8.3 to -6.6)
Denmark	5 548 (4 261 to 6 891)	79 (60 to 98)	-11.9 (-12.1 to -11.7)
Finland	6 187 (4 710 to 7 597)	87 (67 to 108)	-3.6 (-4.1 to -3.1)
France	65 494 (49 777 to 80 769)	81 (62 to 100)	-18.5 (-19.3 to -18.0)
Germany	82 143 (62 650 to 101 447)	76 (57 to 95)	-12.9 (-14.4 to -12.0)
Greece	12 769 (9 940 to 15 660)	91 (69 to 111)	-7.0 (-7.2 to -6.8)

Location	YLDs (95% UI)		
	2016 counts	2016 age-standardised rates per 100,000	Percentage change in age-standardised rates between 1990 and 2016
Iceland	284<br>(212 to 352)	76<br>(56 to 95)	-6.4<br>(-6.9 to -5.8)
Ireland	4 063<br>(3 077 to 4 995)	78<br>(59 to 98)	-6.4<br>(-8.2 to -5.2)
Israel	6 476<br>(5 010 to 7 995)	79<br>(61 to 97)	3.9<br>(1.9 to 6.9)
Italy	68 866<br>(51 075 to 84 665)	86<br>(63 to 108)	-9.9<br>(-10.3 to -9.4)
Luxembourg	564<br>(428 to 697)	81<br>(60 to 101)	-24.6<br>(-24.7 to -24.5)
Malta	401<br>(306 to 509)	77<br>(58 to 96)	-4.6<br>(-5.6 to -3.9)
Netherlands	15 541<br>(11 548 to 19 376)	73<br>(55 to 89)	0.4<br>(-0.7 to 1.2)
Norway	4 900<br>(3 701 to 5 984)	78<br>(61 to 94)	-3.6<br>(-3.8 to -3.5)
Portugal	10 051<br>(7 927 to 12 479)	73<br>(56 to 90)	-27.1<br>(-27.6 to -26.5)
Spain	47 251<br>(36 277 to 58 331)	80<br>(59 to 101)	-14.8<br>(-15.0 to -14.4)
Sweden	8 827<br>(6 827 to 10 833)	72<br>(55 to 89)	-5.8<br>(-7.2 to -4.9)
Switzerland	7 768<br>(5 895 to 9 668)	72<br>(55 to 90)	-27.8<br>(-28.5 to -27.4)
United Kingdom	53 367<br>(40 826 to 66 729)	68<br>(51 to 84)	-5.9<br>(-6.6 to -5.1)
**Southern Latin America**	**37 247<br>(29 218 to 45 481)**	**55<br>(43 to 68)**	**13.5<br>(12.5 to 14.9)**
Argentina	24 895<br>(19 375 to 30 506)	56<br>(43 to 68)	16.1<br>(14.6 to 17.8)
Chile	10 063<br>(7 698 to 12 528)	51<br>(39 to 63)	7.5<br>(6.8 to 8.6)
Uruguay	2 286<br>(1 738 to 2 784)	59<br>(46 to 73)	15.2<br>(14.6 to 15.7)
**Central Europe, Eastern Europe, and Central Asia**	**1 098 567<br>(822 219 to 1 387 531)**	**228<br>(166 to 288)**	**1.0<br>(0.8 to 1.1)**
**Eastern Europe**	**585 114<br>(429 634 to 748 230)**	**230<br>(169 to 294)**	**0.5<br>(-0.1 to 0.9)**
Belarus	29 480<br>(21 326 to 36 770)	254<br>(184 to 324)	15.3<br>(15.1 to 15.4)
Estonia	3 754<br>(2 780 to 4 697)	228<br>(166 to 292)	-11.7<br>(-12.5 to -11.1)
Latvia	5 712<br>(4 187 to 7 152)	227<br>(163 to 291)	-15.4<br>(-16.7 to -14.7)
Lithuania	9 403<br>(7 018 to 11 897)	254<br>(183 to 321)	-0.9<br>(-1.5 to -0.6)
Moldova	8 583<br>(6 163 to 10 894)	184<br>(132 to 237)	-11.7<br>(-12.7 to -11.1)
Russia	412 679<br>(307 291 to 515 549)	236<br>(174 to 300)	1.5<br>(0.9 to 1.9)
Ukraine	115 536<br>(85 455 to 146 376)	208<br>(148 to 260)	-3.9<br>(-4.3 to -3.7)
**Central Europe**	**383 486<br>(286 605 to 480 906)**	**265<br>(201 to 337)**	**5.3<br>(4.9 to 5.8)**
Albania	7 145<br>(5 200 to 9 105)	220<br>(162 to 282)	12.4<br>(11.6 to 12.9)
Bosnia and Herzegovina	11 058<br>(8 346 to 13 910)	235<br>(176 to 293)	50.7<br>(50.3 to 51.2)
Bulgaria	22 833<br>(16 936 to 29 093)	246<br>(177 to 314)	-0.7<br>(-1.4 to -0.3)

Location	YLDs (95% UI)		
	2016 counts	2016 age-standardised rates per 100,000	Percentage change in age-standardised rates between 1990 and 2016
Croatia	13 300<br>(9 657 to 16 773)	240<br>(176 to 308)	0.8<br>(-0.2 to 1.8)
Czech Republic	43 283<br>(30 743 to 54 413)	323<br>(231 to 408)	11.0<br>(9.9 to 11.7)
Hungary	32 173<br>(23 992 to 40 721)	253<br>(184 to 321)	-6.7<br>(-7.0 to -6.5)
Macedonia	5 430<br>(3 904 to 6 879)	226<br>(160 to 284)	18.3<br>(17.6 to 18.8)
Montenegro	1 841<br>(1 347 to 2 346)	253<br>(185 to 326)	12.1<br>(12.0 to 12.2)
Poland	131 118<br>(94 297 to 166 537)	273<br>(203 to 348)	9.1<br>(8.5 to 10.0)
Romania	63 022<br>(45 867 to 80 231)	259<br>(187 to 327)	-3.7<br>(-4.5 to -2.2)
Serbia	25 279<br>(18 635 to 32 486)	236<br>(174 to 300)	16.5<br>(15.7 to 17.7)
Slovakia	17 917<br>(13 220 to 22 915)	270<br>(194 to 347)	-1.0<br>(-2.6 to -0.0)
Slovenia	9 172<br>(6 866 to 11 462)	335<br>(241 to 421)	6.1<br>(5.7 to 6.9)
**Central Asia**	**128 875<br>(95 089 to 162 889)**	**156<br>(111 to 196)**	**3.3<br>(3.1 to 3.5)**
Armenia	5 890<br>(4 448 to 7 364)	174<br>(132 to 218)	-11.0<br>(-12.4 to -8.9)
Azerbaijan	14 870<br>(10 837 to 18 962)	148<br>(108 to 185)	1.6<br>(1.5 to 1.9)
Georgia	7 034<br>(5 007 to 9 043)	153<br>(111 to 198)	-3.3<br>(-4.4 to -2.5)
Kazakhstan	32 470<br>(22 797 to 41 824)	185<br>(129 to 236)	13.8<br>(13.3 to 14.7)
Kyrgyzstan	7 632<br>(5 501 to 9 708)	146<br>(108 to 185)	-8.3<br>(-8.7 to -7.6)
Mongolia	5 102<br>(3 539 to 6 581)	184<br>(130 to 240)	50.8<br>(49.1 to 51.9)
Tajikistan	9 305<br>(6 887 to 11 882)	135<br>(99 to 175)	-6.7<br>(-7.2 to -5.9)
Turkmenistan	7 012<br>(5 043 to 9 010)	141<br>(102 to 182)	4.5<br>(4.1 to 4.8)
Uzbekistan	39 501<br>(28 309 to 51 464)	142<br>(101 to 181)	6.6<br>(6.1 to 7.4)
**Latin America and Caribbean**	**547 886<br>(396 123 to 686 582)**	**101<br>(73 to 128)**	**3.7<br>(3.4 to 4.0)**
**Central Latin America**	**209 949<br>(149 787 to 268 238)**	**91<br>(65 to 115)**	**-4.3<br>(-4.7 to -3.9)**
Colombia	44 630<br>(31 710 to 57 041)	94<br>(68 to 119)	-1.2<br>(-2.8 to -0.2)
Costa Rica	4 960<br>(3 578 to 6 322)	101<br>(74 to 128)	23.0<br>(22.2 to 24.3)
El Salvador	5 562<br>(4 035 to 6 997)	98<br>(72 to 123)	5.7<br>(4.4 to 7.2)
Guatemala	10 842<br>(7 789 to 13 695)	87<br>(64 to 110)	17.9<br>(17.1 to 18.7)
Honduras	5 595<br>(4 182 to 6 987)	84<br>(64 to 105)	30.5<br>(29.1 to 32.8)
Mexico	98 185<br>(69 473 to 126 008)	84<br>(62 to 108)	-15.6<br>(-15.8 to -15.4)
Nicaragua	4 326<br>(3 155 to 5 508)	84<br>(61 to 108)	1.4<br>(-0.7 to 2.9)
Panama	3 790<br>(2 702 to 4 858)	101<br>(71 to 129)	21.5<br>(21.1 to 22.3)

Location	YLDs (95% UI)		
	2016 counts	2016 age-standardised rates per 100,000	Percentage change in age-standardised rates between 1990 and 2016
Venezuela	32 012<br>(23 853 to 40 893)	110<br>(79 to 139)	18.6<br>(18.1 to 19.6)
**Andean Latin America**	**49 821<br>(35 402 to 62 771)**	**94<br>(68 to 120)**	**11.6<br>(9.0 to 13.0)**
Bolivia	8 224<br>(5 718 to 10 632)	89<br>(64 to 115)	2.5<br>(0.9 to 3.4)
Ecuador	14 927<br>(10 679 to 19 228)	102<br>(75 to 131)	10.8<br>(10.5 to 11.0)
Peru	26 669<br>(18 562 to 34 420)	92<br>(66 to 119)	14.7<br>(10.8 to 16.9)
**Caribbean**	**46 874<br>(34 276 to 60 229)**	**103<br>(76 to 131)**	**27.5<br>(26.2 to 29.7)**
Antigua and Barbuda	88<br>(62 to 114)	95<br>(68 to 123)	17.3<br>(14.8 to 18.7)
The Bahamas	401<br>(297 to 521)	98<br>(70 to 125)	11.0<br>(10.8 to 11.1)
Barbados	293<br>(214 to 369)	89<br>(63 to 114)	22.2<br>(21.5 to 22.6)
Belize	294<br>(219 to 373)	97<br>(71 to 121)	28.5<br>(28.0 to 29.4)
Bermuda	77<br>(56 to 99)	108<br>(77 to 138)	5.8<br>(4.5 to 6.7)
Cuba	13 933<br>(9 777 to 17 507)	101<br>(72 to 129)	9.8<br>(9.3 to 10.1)
Dominica	68<br>(47 to 88)	91<br>(63 to 119)	34.8<br>(32.3 to 36.2)
Dominican Republic	9 641<br>(6 783 to 12 343)	101<br>(69 to 130)	32.0<br>(31.5 to 32.4)
Grenada	95<br>(67 to 123)	95<br>(71 to 120)	30.2<br>(29.3 to 31.6)
Guyana	612<br>(435 to 785)	86<br>(63 to 110)	17.3<br>(16.9 to 17.5)
Haiti	10 154<br>(6 807 to 13 434)	106<br>(74 to 137)	63.1<br>(56.6 to 66.7)
Jamaica	2 394<br>(1 707 to 3 058)	86<br>(62 to 111)	35.4<br>(33.2 to 39.0)
Puerto Rico	4 578<br>(3 307 to 5 913)	109<br>(80 to 137)	22.2<br>(21.6 to 23.2)
Saint Lucia	177<br>(130 to 225)	94<br>(67 to 119)	23.9<br>(22.3 to 27.1)
Saint Vincent and the Grenadines	102<br>(70 to 134)	94<br>(66 to 123)	29.1<br>(26.5 to 30.6)
Suriname	507<br>(375 to 651)	96<br>(70 to 120)	25.9<br>(23.8 to 27.2)
Trinidad and Tobago	1 431<br>(1 017 to 1 890)	98<br>(70 to 126)	32.7<br>(29.6 to 34.6)
Virgin Islands, U.S.	121<br>(90 to 150)	95<br>(68 to 122)	15.0<br>(14.5 to 15.9)
**Tropical Latin America**	**241 040<br>(173 317 to 308 813)**	**112<br>(81 to 142)**	**5.5<br>(5.2 to 6.0)**
Brazil	235 110<br>(166 808 to 303 358)	112<br>(81 to 143)	5.0<br>(4.7 to 5.5)
Paraguay	5 921<br>(4 198 to 7 608)	101<br>(71 to 130)	30.4<br>(29.4 to 30.9)
**Southeast Asia, East Asia, and Oceania**	**2 411 705<br>(1 651 377 to 3 043 854)**	**106<br>(77 to 135)**	**39.5<br>(38.9 to 40.0)**
**East Asia**	**1 804 562<br>(1 308 026 to 2 283 906)**	**110<br>(79 to 141)**	**40.1<br>(39.6 to 40.7)**
China	1 750 360<br>(1 244 743 to 2 261 305)	110<br>(79 to 140)	40.2<br>(39.7 to 40.7)

Location	YLDs (95% UI)		
	2016 counts	2016 age-standardised rates per 100,000	Percentage change in age-standardised rates between 1990 and 2016
North Korea	24 005 (17 353 to 30 695)	87 (64 to 109)	48.4 (47.0 to 49.2)
Taiwan (Province of China)	30 195 (21 162 to 38 776)	105 (75 to 137)	28.2 (28.0 to 28.4)
**Southeast Asia**	**598 401 (443 927 to 754 472)**	**95 (71 to 121)**	**38.4 (37.2 to 39.3)**
Cambodia	11 768 (8 417 to 15 123)	89 (67 to 114)	23.6 (17.9 to 32.7)
Indonesia	213 821 (154 591 to 268 851)	87 (63 to 112)	31.9 (31.4 to 32.4)
Laos	4 328 (3 078 to 5 687)	77 (55 to 97)	50.5 (49.4 to 51.8)
Malaysia	32 172 (22 748 to 40 873)	110 (81 to 144)	38.3 (36.6 to 39.5)
Maldives	241 (179 to 305)	74 (55 to 95)	19.0 (17.0 to 20.4)
Mauritius	1 276 (924 to 1 630)	90 (65 to 116)	47.1 (44.7 to 48.5)
Myanmar	45 307 (31 671 to 58 451)	87 (63 to 113)	68.9 (64.6 to 71.8)
Philippines	77 797 (56 554 to 98 225)	87 (66 to 109)	37.9 (37.1 to 39.2)
Sri Lanka	24 982 (18 435 to 31 472)	116 (87 to 148)	31.7 (30.6 to 33.1)
Seychelles	102 (78 to 129)	102 (75 to 129)	39.4 (38.4 to 41.1)
Thailand	95 805 (67 085 to 123 019)	121 (87 to 152)	31.9 (30.9 to 32.5)
Timor-Leste	870 (614 to 1 122)	102 (73 to 130)	34.0 (29.4 to 41.9)
Vietnam	88 658 (64 008 to 114 045)	95 (71 to 121)	53.3 (51.3 to 54.6)
**Oceania**	**7 734 (5 556 to 9 832)**	**82 (58 to 103)**	**35.6 (34.9 to 36.0)**
American Samoa	66 (48 to 83)	95 (68 to 121)	16.2 (14.8 to 17.0)
Federated States of Micronesia	69 (50 to 86)	80 (59 to 102)	29.3 (27.5 to 32.2)
Fiji	677 (505 to 864)	79 (56 to 99)	40.5 (39.7 to 40.9)
Guam	186 (132 to 238)	107 (78 to 135)	30.3 (30.0 to 30.6)
Kiribati	69 (50 to 88)	70 (50 to 90)	43.2 (40.8 to 44.7)
Marshall Islands	47 (34 to 61)	77 (57 to 95)	27.9 (26.2 to 31.0)
Northern Mariana Islands	114 (85 to 149)	104 (74 to 134)	10.4 (9.2 to 12.4)
Papua New Guinea	5 181 (3 722 to 6 502)	82 (60 to 106)	36.7 (36.0 to 37.2)
Samoa	140 (100 to 179)	86 (63 to 109)	32.5 (31.4 to 34.5)
Solomon Islands	368 (274 to 463)	79 (58 to 100)	34.8 (33.5 to 35.6)
Tonga	74 (53 to 94)	83 (61 to 105)	23.6 (22.5 to 25.5)
Vanuatu	179 (136 to 224)	79 (59 to 100)	43.0 (41.8 to 45.2)
**North Africa and Middle East**	**582 957 (440 793 to 716 231)**	**114 (86 to 143)**	**1.9 (1.7 to 2.2)**

Location	YLDs (95% UI)		
	2016 counts	2016 age-standardised rates per 100,000	Percentage change in age-standardised rates between 1990 and 2016
**North Africa and Middle East**	**582 957 (440 793 to 716 231)**	**114 (86 to 143)**	**1.9 (1.7 to 2.2)**
Afghanistan	31 415 (20 159 to 42 061)	133 (82 to 184)	-10.0 (-13.0 to -2.9)
Algeria	38 261 (27 921 to 47 981)	105 (79 to 131)	-0.5 (-1.0 to 0.2)
Bahrain	1 623 (1 165 to 2 085)	118 (86 to 150)	-1.7 (-2.3 to -1.3)
Egypt	72 253 (52 350 to 93 264)	89 (66 to 110)	22.9 (22.5 to 23.5)
Iran	103 580 (77 924 to 126 867)	136 (104 to 168)	-4.7 (-6.4 to -2.5)
Iraq	45 223 (31 681 to 60 387)	160 (108 to 210)	-7.7 (-10.2 to -3.5)
Jordan	6 020 (4 211 to 7 746)	94 (69 to 121)	-10.6 (-11.1 to -9.6)
Kuwait	5 074 (3 849 to 6 486)	135 (101 to 169)	-4.3 (-5.1 to -2.9)
Lebanon	9 036 (5 996 to 12 219)	158 (100 to 210)	-18.1 (-22.3 to -6.6)
Libya	6 894 (5 042 to 8 778)	118 (90 to 149)	5.8 (1.2 to 11.2)
Morocco	31 325 (22 804 to 39 602)	95 (68 to 122)	6.1 (4.7 to 6.8)
Palestine	3 815 (2 754 to 4 855)	104 (75 to 134)	-0.6 (-2.9 to 3.3)
Oman	6 317 (4 360 to 8 083)	148 (106 to 189)	0.7 (0.1 to 1.8)
Qatar	3 786 (2 691 to 4 842)	172 (125 to 222)	2.2 (1.9 to 2.6)
Saudi Arabia	36 592 (27 055 to 46 235)	128 (95 to 159)	-9.1 (-9.3 to -9.0)
Sudan	29 393 (22 197 to 37 197)	93 (70 to 116)	12.8 (11.7 to 14.6)
Syria	20 482 (13 453 to 27 453)	127 (87 to 166)	50.9 (29.5 to 70.3)
Tunisia	11 885 (8 731 to 15 385)	104 (76 to 134)	8.1 (6.2 to 9.4)
Turkey	82 604 (60 682 to 104 118)	105 (78 to 132)	-12.0 (-13.0 to -11.5)
United Arab Emirates	15 412 (10 959 to 19 976)	159 (114 to 204)	-2.9 (-4.6 to -1.9)
Yemen	21 162 (15 990 to 27 203)	101 (77 to 126)	7.5 (4.9 to 10.2)
**South Asia**	**1 821 795 (1 322 861 to 2 323 116)**	**121 (90 to 154)**	**16.0 (15.2 to 16.6)**
**South Asia**	**1 821 795 (1 322 861 to 2 323 116)**	**121 (90 to 154)**	**16.0 (15.2 to 16.6)**
Bangladesh	144 420 (104 911 to 185 476)	102 (74 to 128)	17.2 (15.8 to 18.6)
Bhutan	811 (612 to 1 023)	120 (90 to 149)	8.0 (7.5 to 8.9)
India	1 465 564 (1 077 785 to 1 842 837)	124 (93 to 154)	14.9 (14.1 to 15.5)
Nepal	27 634 (21 082 to 35 189)	112 (82 to 144)	22.6 (22.1 to 23.0)
Pakistan	183 186 (133 667 to 235 361)	118 (87 to 149)	25.5 (24.8 to 26.6)
**Sub-Saharan Africa**	**624 210 (459 720 to 785 906)**	**91 (67 to 115)**	**1.2 (0.9 to 1.6)**

Location	YLDs (95% UI)		
	2016 counts	2016 age-standardised rates per 100,000	Percentage change in age-standardised rates between 1990 and 2016
**Southern sub-Saharan Africa**	**62 135 (44 085 to 79 085)**	**93 (68 to 117)**	**-15.6 (-16.1 to -14.8)**
Botswana	1 907 (1 391 to 2 372)	98 (74 to 123)	14.7 (12.8 to 18.0)
Lesotho	1 336 (1 013 to 1 681)	76 (57 to 98)	6.2 (5.0 to 8.2)
Namibia	1 704 (1 241 to 2 199)	85 (61 to 108)	1.4 (0.4 to 1.9)
South Africa	48 294 (34 624 to 61 368)	99 (74 to 125)	-19.7 (-20.0 to -19.2)
Swaziland	935 (676 to 1 202)	92 (68 to 115)	5.0 (4.0 to 6.4)
Zimbabwe	7 835 (5 592 to 9 982)	68 (49 to 88)	0.5 (-1.5 to 4.5)
**Western sub-Saharan Africa**	**241 993 (178 590 to 312 721)**	**87 (64 to 110)**	**4.0 (3.7 to 4.2)**
Benin	7 041 (5 137 to 8 908)	90 (65 to 115)	8.4 (6.8 to 9.4)
Burkina Faso	10 398 (7 294 to 13 352)	83 (62 to 106)	7.7 (5.0 to 9.6)
Cameroon	14 307 (10 438 to 18 350)	84 (62 to 105)	1.3 (0.6 to 2.3)
Cape Verde	422 (301 to 549)	94 (67 to 118)	16.0 (14.1 to 17.1)
Chad	8 197 (5 916 to 10 572)	87 (62 to 109)	5.5 (3.2 to 7.1)
Cote d'Ivoire	14 816 (10 648 to 18 894)	90 (67 to 112)	-2.6 (-3.8 to -1.9)
The Gambia	1 097 (789 to 1 404)	81 (59 to 102)	-8.3 (-8.5 to -8.2)
Ghana	18 694 (13 462 to 23 706)	90 (68 to 113)	15.6 (14.4 to 16.6)
Guinea	7 328 (5 350 to 9 246)	79 (59 to 100)	-3.9 (-6.8 to -2.0)
Guinea-Bissau	1 164 (836 to 1 496)	83 (62 to 106)	-4.5 (-4.9 to -3.9)
Liberia	2 636 (1 961 to 3 281)	80 (61 to 101)	1.3 (0.1 to 2.7)
Mali	9 708 (6 838 to 12 279)	82 (60 to 103)	7.9 (5.2 to 9.5)
Mauritania	2 877 (2 104 to 3 735)	95 (67 to 120)	6.4 (5.5 to 7.0)
Niger	10 177 (7 130 to 13 193)	77 (54 to 99)	-4.2 (-5.8 to -3.2)
Nigeria	115 628 (81 196 to 149 329)	90 (64 to 116)	4.9 (4.6 to 5.3)
Sao Tome and Principe	142 (98 to 184)	104 (76 to 134)	6.0 (3.7 to 7.4)
Senegal	9 215 (6 772 to 11 826)	86 (63 to 109)	2.8 (1.8 to 3.3)
Sierra Leone	3 885 (2 905 to 4 904)	84 (64 to 104)	-1.1 (-1.9 to 0.3)
Togo	4 201 (3 019 to 5 412)	79 (56 to 101)	-1.6 (-2.1 to -0.7)
**Eastern sub-Saharan Africa**	**245 622 (181 337 to 312 153)**	**93 (69 to 118)**	**5.9 (5.2 to 6.7)**
Burundi	7 766 (5 584 to 10 004)	101 (75 to 125)	22.4 (22.1 to 22.9)
Comoros	562 (404 to 719)	98 (73 to 124)	-15.4 (-15.9 to -14.5)

Location	YLDs (95% UI)		
	2016 counts	2016 age-standardised rates per 100,000	Percentage change in age-standardised rates between 1990 and 2016
Djibouti	769<br>(555 to 982)	104<br>(76 to 133)	-1.7<br>(-2.0 to -1.2)
Eritrea	3 660<br>(2 641 to 4 733)	100<br>(75 to 128)	14.7<br>(13.5 to 16.5)
Ethiopia	67 782<br>(50 073 to 86 187)	96<br>(72 to 120)	5.7<br>(4.1 to 7.4)
Kenya	32 206<br>(23 074 to 41 242)	98<br>(70 to 126)	12.2<br>(11.8 to 12.5)
Madagascar	14 902<br>(10 847 to 18 873)	84<br>(63 to 107)	-1.8<br>(-2.1 to -1.5)
Malawi	8 470<br>(5 893 to 10 837)	70<br>(51 to 89)	-9.1<br>(-9.3 to -8.9)
Mozambique	17 171<br>(12 338 to 21 836)	90<br>(66 to 113)	3.4<br>(0.5 to 5.9)
Rwanda	9 487<br>(6 589 to 12 554)	114<br>(76 to 150)	17.8<br>(6.2 to 26.3)
Somalia	6 773<br>(4 865 to 8 567)	94<br>(69 to 118)	-2.9<br>(-5.4 to -0.7)
South Sudan	9 523<br>(7 074 to 12 129)	105<br>(79 to 131)	2.8<br>(1.7 to 4.6)
Tanzania	33 604<br>(23 477 to 44 186)	90<br>(64 to 114)	1.5<br>(0.8 to 1.8)
Uganda	22 364<br>(16 844 to 27 414)	89<br>(67 to 108)	11.5<br>(8.8 to 16.0)
Zambia	10 377<br>(7 644 to 13 413)	94<br>(69 to 118)	4.5<br>(4.1 to 5.3)
**Central sub-Saharan Africa**	**74 195<br>(56 171 to 93 632)**	**92<br>(70 to 116)**	**3.2<br>(2.8 to 3.4)**
Angola	18 508<br>(13 541 to 23 233)	113<br>(85 to 142)	7.5<br>(6.4 to 8.3)
Central African Republic	2 799<br>(2 035 to 3 531)	71<br>(52 to 91)	6.2<br>(5.2 to 6.9)
Congo	3 561<br>(2 645 to 4 442)	104<br>(77 to 128)	15.8<br>(15.6 to 16.3)
DR Congo	47 065<br>(35 505 to 59 167)	87<br>(66 to 109)	0.0<br>(-0.1 to 0.1)
Equatorial Guinea	766<br>(562 to 978)	117<br>(85 to 146)	38.5<br>(36.5 to 40.1)
Gabon	1 489<br>(1 060 to 1 887)	107<br>(77 to 136)	-3.1<br>(-3.9 to -2.4)

Appendix Table 10: YLDs for 2016 and percentage change of age-standardized rates by location for SCI			
Location	YLDs (95% UI)		
	2016 counts	2016 age-standardised rates per 100,000	Percentage change in age-standardised rates between 1990 and 2016
**Global**	**9 521 880 (6 699 959 to 12 448 857)**	**130 (90 to 170)**	**-10.0 (-13.3 to -7.0)**
**High SDI**	**2 757 053 (1 905 208 to 3 622 994)**	**228 (161 to 298)**	**-0.6 (-1.3 to 0.0)**
**High-middle SDI**	**1 731 772 (1 207 228 to 2 265 414)**	**134 (94 to 176)**	**-15.4 (-18.6 to -12.4)**
**Middle SDI**	**1 825 020 (1 280 772 to 2 376 430)**	**75 (53 to 98)**	**4.5 (-0.7 to 9.0)**
**Low-middle SDI**	**2 199 303 (1 538 056 to 2 986 060)**	**111 (76 to 149)**	**12.5 (8.3 to 16.3)**
**Low SDI**	**972 553 (529 268 to 1 525 018)**	**167 (85 to 263)**	**10.2 (3.7 to 14.2)**
**High-income**	**2 848 222 (1 982 040 to 3 731 454)**	**229 (159 to 303)**	**-1.4 (-2.1 to -0.6)**
**High-income North America**	**865 483 (601 530 to 1 141 832)**	**208 (145 to 274)**	**-6.0 (-7.0 to -5.0)**
Canada	95 472 (66 046 to 125 086)	222 (156 to 288)	1.1 (0.4 to 1.9)
Greenland	120 (84 to 158)	218 (154 to 286)	-11.0 (-16.0 to -6.3)
United States	769 600 (526 327 to 1 010 219)	207 (144 to 275)	-6.9 (-8.0 to -5.7)
**Australasia**	**70 773 (48 770 to 93 684)**	**220 (154 to 288)**	**4.2 (2.7 to 5.6)**
Australia	59 334 (40 953 to 77 251)	219 (152 to 289)	4.8 (3.4 to 6.2)
New Zealand	11 303 (7 832 to 15 081)	224 (155 to 296)	1.2 (-0.2 to 2.5)
**High-income Asia-Pacific**	**539 960 (376 562 to 703 358)**	**243 (168 to 320)**	**0.5 (-1.5 to 2.4)**
Brunei	1 185 (825 to 1 558)	271 (189 to 359)	-15.0 (-18.3 to -11.8)
Japan	384 348 (265 928 to 510 732)	244 (170 to 319)	5.5 (3.4 to 7.4)
Singapore	11 697 (8 066 to 15 370)	259 (180 to 341)	11.3 (8.0 to 14.1)
South Korea	141 913 (99 545 to 187 790)	239 (168 to 314)	-17.9 (-21.3 to -14.9)
**Western Europe**	**1 262 332 (881 721 to 1 662 432)**	**252 (174 to 331)**	**1.3 (0.2 to 2.3)**
Andorra	244 (170 to 321)	261 (181 to 346)	7.4 (7.0 to 7.8)
Austria	28 564 (19 596 to 37 360)	276 (190 to 362)	-4.7 (-6.0 to -3.5)
Belgium	35 457 (24 965 to 46 697)	268 (185 to 358)	4.6 (3.2 to 5.9)
Cyprus	2 667 (1 862 to 3 497)	261 (182 to 348)	-0.0 (-2.7 to 2.2)
Denmark	16 899 (11 583 to 22 444)	258 (181 to 341)	6.1 (4.8 to 7.3)
Finland	18 817 (13 038 to 24 595)	287 (197 to 381)	12.4 (11.5 to 13.4)
France	188 864 (129 727 to 248 764)	252 (176 to 332)	-4.6 (-5.6 to -3.5)
Germany	244 786 (170 335 to 322 606)	248 (170 to 324)	3.9 (2.3 to 5.5)
Greece	32 525 (22 416 to 42 927)	252 (176 to 332)	-3.2 (-3.7 to -2.7)

Location	YLDs (95% UI)		
	2016 counts	2016 age-standardised rates per 100,000	Percentage change in age-standardised rates between 1990 and 2016
Iceland	922 (640 to 1 200)	255 (177 to 335)	8.4 (7.1 to 9.6)
Ireland	14 187 (9 971 to 18 667)	283 (192 to 373)	14.3 (12.6 to 15.8)
Israel	22 838 (15 120 to 32 295)	282 (181 to 396)	18.5 (7.2 to 29.4)
Italy	192 772 (135 952 to 252 122)	264 (181 to 346)	1.7 (0.4 to 2.9)
Luxembourg	1 682 (1 179 to 2 223)	252 (176 to 331)	-12.7 (-14.0 to -11.6)
Malta	1 311 (909 to 1 726)	268 (186 to 353)	-0.1 (-1.5 to 0.8)
Netherlands	44 836 (31 617 to 59 023)	225 (158 to 296)	9.5 (8.6 to 10.5)
Norway	15 415 (10 729 to 20 220)	259 (179 to 339)	10.6 (9.4 to 11.6)
Portugal	27 056 (18 944 to 35 457)	215 (147 to 286)	-22.6 (-24.3 to -21.0)
Spain	136 539 (96 151 to 179 204)	248 (171 to 330)	2.2 (0.8 to 3.4)
Sweden	29 882 (20 887 to 39 339)	267 (185 to 353)	8.0 (7.3 to 8.6)
Switzerland	23 432 (16 224 to 30 661)	231 (160 to 301)	-23.1 (-24.1 to -21.9)
United Kingdom	179 945 (123 666 to 233 755)	245 (172 to 324)	3.0 (1.9 to 4.1)
**Southern Latin America**	**113 861 (80 684 to 149 355)**	**168 (118 to 221)**	**6.1 (2.5 to 9.3)**
Argentina	76 759 (54 378 to 98 743)	174 (120 to 225)	8.5 (4.5 to 11.7)
Chile	30 982 (21 372 to 40 580)	158 (110 to 209)	0.5 (-3.3 to 4.0)
Uruguay	5 989 (4 235 to 7 888)	162 (112 to 213)	9.5 (5.6 to 12.5)
**Central Europe, Eastern Europe, and Central Asia**	**717 886 (503 935 to 952 526)**	**156 (110 to 205)**	**-7.7 (-12.6 to -3.1)**
**Eastern Europe**	**358 790 (249 645 to 474 839)**	**151 (105 to 198)**	**-8.5 (-12.4 to -3.7)**
Belarus	18 189 (12 629 to 23 870)	166 (115 to 218)	4.8 (2.3 to 7.1)
Estonia	2 447 (1 701 to 3 243)	161 (112 to 212)	-8.6 (-10.6 to -6.7)
Latvia	3 459 (2 410 to 4 531)	151 (106 to 200)	-14.7 (-16.6 to -13.1)
Lithuania	5 621 (3 897 to 7 339)	166 (115 to 220)	-2.5 (-4.4 to -0.9)
Moldova	6 566 (4 591 to 8 631)	144 (101 to 187)	-11.4 (-15.9 to -6.6)
Russia	249 643 (173 130 to 327 305)	152 (107 to 200)	-9.0 (-13.4 to -3.4)
Ukraine	73 805 (52 052 to 96 707)	143 (99 to 190)	-8.8 (-12.9 to -4.2)
**Central Europe**	**241 103 (167 504 to 314 190)**	**178 (124 to 235)**	**0.0 (-5.3 to 5.5)**
Albania	5 170 (3 577 to 6 960)	164 (113 to 218)	6.1 (-4.8 to 19.4)
Bosnia and Herzegovina	11 023 (6 234 to 16 435)	244 (139 to 376)	80.6 (18.4 to 162.4)
Bulgaria	13 748 (9 579 to 18 202)	163 (114 to 213)	-7.2 (-9.3 to -5.3)

Location	YLDs (95% UI)		
	2016 counts	2016 age-standardised rates per 100,000	Percentage change in age-standardised rates between 1990 and 2016
Croatia	8 605 (5 541 to 12 296)	171 (113 to 244)	19.6 (-3.1 to 48.9)
Czech Republic	27 015 (18 967 to 35 868)	216 (151 to 283)	17.5 (15.1 to 19.7)
Hungary	18 910 (13 212 to 25 192)	163 (115 to 214)	-2.2 (-4.8 to -0.1)
Macedonia	3 605 (2 501 to 4 726)	154 (109 to 204)	9.1 (5.1 to 13.0)
Montenegro	1 160 (802 to 1 536)	166 (115 to 221)	6.8 (4.0 to 9.4)
Poland	78 749 (54 400 to 102 079)	174 (121 to 228)	3.6 (0.6 to 6.2)
Romania	38 039 (26 670 to 49 889)	168 (117 to 219)	-16.5 (-19.1 to -14.0)
Serbia	18 691 (11 832 to 26 005)	189 (124 to 274)	33.4 (6.7 to 65.8)
Slovakia	11 028 (7 677 to 14 512)	174 (122 to 227)	0.4 (-1.8 to 2.3)
Slovenia	5 496 (3 853 to 7 130)	217 (150 to 285)	14.5 (12.5 to 16.1)
**Central Asia**	**112 655 (79 348 to 148 518)**	**130 (91 to 170)**	**-2.2 (-7.8 to 4.0)**
Armenia	5 276 (3 409 to 7 362)	159 (104 to 224)	-25.0 (-30.9 to -16.1)
Azerbaijan	13 658 (9 293 to 18 315)	132 (90 to 176)	4.2 (-9.0 to 18.3)
Georgia	5 597 (3 746 to 7 587)	128 (88 to 171)	3.5 (-10.1 to 19.7)
Kazakhstan	22 843 (16 023 to 29 911)	128 (90 to 167)	-3.2 (-7.9 to 0.9)
Kyrgyzstan	6 541 (4 587 to 8 443)	117 (82 to 151)	-12.1 (-15.4 to -8.8)
Mongolia	4 174 (2 967 to 5 396)	140 (98 to 178)	23.8 (18.2 to 28.9)
Tajikistan	11 709 (7 497 to 17 336)	156 (99 to 225)	20.1 (-10.1 to 56.0)
Turkmenistan	5 979 (4 228 to 7 762)	112 (79 to 145)	-4.0 (-7.8 to -1.0)
Uzbekistan	33 912 (24 186 to 44 288)	115 (81 to 149)	-1.6 (-5.6 to 1.7)
**Latin America and Caribbean**	**414 777 (292 583 to 535 447)**	**73 (51 to 95)**	**-12.1 (-15.5 to -8.9)**
**Central Latin America**	**157 739 (108 606 to 206 337)**	**64 (45 to 85)**	**-18.1 (-21.2 to -15.2)**
Colombia	32 629 (22 582 to 42 642)	67 (46 to 88)	-8.1 (-14.1 to -2.3)
Costa Rica	3 150 (2 187 to 4 110)	63 (44 to 81)	13.2 (9.3 to 16.5)
El Salvador	5 912 (3 110 to 9 818)	102 (52 to 171)	-24.6 (-32.3 to -4.3)
Guatemala	10 391 (6 634 to 14 308)	77 (50 to 109)	-8.1 (-18.3 to 6.9)
Honduras	5 209 (3 623 to 6 745)	71 (49 to 92)	32.4 (15.5 to 49.3)
Mexico	69 571 (48 294 to 89 684)	56 (39 to 73)	-28.1 (-31.0 to -25.4)
Nicaragua	5 381 (2 921 to 8 899)	94 (50 to 151)	-22.3 (-30.2 to -3.9)
Panama	2 592 (1 788 to 3 368)	67 (46 to 87)	8.1 (3.2 to 13.0)

Location	YLDs (95% UI)		
	2016 counts	2016 age-standardised rates per 100,000	Percentage change in age-standardised rates between 1990 and 2016
Venezuela	21 184 (14 673 to 27 539)	70 (48 to 91)	6.7 (0.3 to 12.3)
**Andean Latin America**	**45 245 (31 884 to 58 724)**	**81 (57 to 104)**	**-6.4 (-10.8 to -2.2)**
Bolivia	7 781 (5 547 to 9 968)	78 (55 to 100)	-7.5 (-12.5 to -3.0)
Ecuador	11 870 (8 348 to 15 323)	77 (53 to 100)	-5.8 (-10.6 to -1.9)
Peru	24 899 (17 032 to 32 712)	81 (56 to 107)	-6.5 (-12.1 to -1.2)
**Caribbean**	**41 863 (28 988 to 56 203)**	**91 (63 to 123)**	**34.8 (12.7 to 59.8)**
Antigua and Barbuda	67 (46 to 89)	71 (50 to 93)	4.9 (0.2 to 9.2)
The Bahamas	284 (201 to 365)	69 (48 to 90)	7.2 (2.9 to 11.2)
Barbados	201 (139 to 263)	64 (45 to 85)	10.0 (5.4 to 13.8)
Belize	245 (173 to 315)	73 (51 to 94)	16.1 (11.1 to 21.1)
Bermuda	52 (36 to 68)	71 (50 to 94)	6.3 (3.0 to 9.2)
Cuba	9 121 (6 333 to 11 990)	70 (49 to 91)	8.1 (4.9 to 11.0)
Dominica	51 (36 to 67)	68 (47 to 89)	22.2 (17.1 to 27.3)
Dominican Republic	7 271 (5 101 to 9 525)	72 (51 to 94)	14.0 (8.3 to 19.3)
Grenada	77 (54 to 99)	73 (52 to 93)	15.0 (10.2 to 19.8)
Guyana	510 (363 to 654)	68 (48 to 87)	8.0 (4.2 to 11.3)
Haiti	15 834 (7 246 to 25 887)	153 (68 to 264)	155.6 (27.3 to 310.4)
Jamaica	1 877 (1 298 to 2 422)	66 (46 to 86)	20.4 (14.4 to 26.0)
Puerto Rico	2 843 (1 961 to 3 703)	70 (49 to 93)	21.8 (18.8 to 24.0)
Saint Lucia	131 (92 to 171)	69 (48 to 89)	12.0 (7.5 to 15.9)
Saint Vincent and the Grenadines	81 (57 to 104)	73 (51 to 93)	15.2 (11.5 to 18.4)
Suriname	409 (293 to 529)	75 (53 to 97)	11.1 (5.9 to 16.1)
Trinidad and Tobago	1 017 (711 to 1 311)	70 (49 to 91)	20.0 (15.4 to 24.0)
Virgin Islands, U.S.	75 (52 to 98)	64 (44 to 85)	2.6 (-2.1 to 7.5)
**Tropical Latin America**	**169 005 (119 576 to 217 697)**	**76 (54 to 98)**	**-13.5 (-16.2 to -11.1)**
Brazil	163 792 (114 740 to 212 481)	76 (53 to 99)	-13.9 (-16.9 to -11.4)
Paraguay	4 590 (3 221 to 5 957)	74 (52 to 95)	9.1 (4.9 to 12.6)
**Southeast Asia, East Asia, and Oceania**	**1 763 251 (1 234 707 to 2 294 799)**	**77 (54 to 100)**	**11.2 (6.4 to 16.0)**
**East Asia**	**1 142 058 (801 451 to 1 502 885)**	**70 (49 to 91)**	**3.8 (-2.1 to 8.8)**
China	1 110 046 (782 610 to 1 449 680)	70 (49 to 93)	3.6 (-2.4 to 8.7)

Location	YLDs (95% UI)		
	2016 counts	2016 age-standardised rates per 100,000	Percentage change in age-standardised rates between 1990 and 2016
North Korea	17 108 (12 125 to 22 063)	62 (44 to 79)	31.7 (26.8 to 36.8)
Taiwan (Province of China)	18 613 (13 044 to 24 465)	67 (47 to 89)	16.2 (11.8 to 20.0)
**Southeast Asia**	**553 175 (385 768 to 738 083)**	**85 (59 to 113)**	**22.0 (13.5 to 31.7)**
Cambodia	21 522 (8 584 to 41 875)	165 (58 to 331)	-25.2 (-34.0 to 35.5)
Indonesia	196 990 (137 687 to 256 769)	77 (54 to 100)	20.2 (10.8 to 31.3)
Laos	3 958 (2 786 to 5 007)	64 (46 to 82)	33.3 (27.1 to 39.3)
Malaysia	21 961 (15 500 to 28 337)	72 (50 to 93)	12.3 (7.5 to 16.9)
Maldives	194 (138 to 255)	56 (39 to 74)	4.1 (-3.1 to 11.4)
Mauritius	884 (614 to 1 148)	64 (45 to 82)	27.8 (22.6 to 32.4)
Myanmar	54 125 (35 770 to 78 598)	100 (65 to 140)	46.5 (15.1 to 98.9)
Philippines	75 028 (50 493 to 102 464)	80 (53 to 110)	16.9 (5.6 to 32.0)
Sri Lanka	33 706 (14 802 to 61 850)	155 (69 to 272)	80.9 (14.2 to 127.5)
Seychelles	72 (50 to 94)	71 (49 to 92)	17.2 (10.3 to 24.2)
Thailand	57 655 (40 081 to 75 186)	74 (52 to 97)	8.6 (3.0 to 13.9)
Timor-Leste	2 503 (568 to 5 363)	305 (61 to 658)	29.4 (22.2 to 34.9)
Vietnam	65 224 (45 681 to 83 562)	67 (47 to 87)	29.2 (22.4 to 35.7)
**Oceania**	**6 834 (4 926 to 8 715)**	**67 (48 to 85)**	**32.7 (26.0 to 39.4)**
American Samoa	58 (41 to 75)	77 (54 to 99)	11.7 (3.7 to 19.8)
Federated States of Micronesia	60 (43 to 77)	64 (45 to 81)	16.5 (8.9 to 23.9)
Fiji	550 (388 to 703)	62 (44 to 80)	30.9 (22.7 to 39.9)
Guam	127 (89 to 166)	73 (50 to 94)	22.1 (16.0 to 28.3)
Kiribati	67 (48 to 85)	63 (45 to 80)	46.0 (36.0 to 57.0)
Marshall Islands	40 (28 to 52)	59 (42 to 76)	13.1 (7.8 to 17.7)
Northern Mariana Islands	87 (61 to 114)	71 (49 to 93)	-0.4 (-6.7 to 5.8)
Papua New Guinea	4 727 (3 346 to 6 041)	67 (47 to 85)	38.4 (29.3 to 47.3)
Samoa	125 (88 to 160)	72 (51 to 91)	27.5 (18.6 to 37.1)
Solomon Islands	342 (243 to 432)	65 (47 to 82)	26.5 (20.0 to 33.1)
Tonga	62 (44 to 81)	65 (46 to 84)	15.9 (9.7 to 21.5)
Vanuatu	162 (115 to 206)	66 (47 to 84)	35.0 (27.6 to 42.1)
**North Africa and Middle East**	**951 760 (408 570 to 1 655 875)**	**177 (77 to 316)**	**-6.2 (-9.5 to -3.5)**

Location	YLDs (95% UI)		
	2016 counts	2016 age-standardised rates per 100,000	Percentage change in age-standardised rates between 1990 and 2016
**North Africa and Middle East**	**951 760 (408 570 to 1 655 875)**	**177 (77 to 316)**	**-6.2 (-9.5 to -3.5)**
Afghanistan	157 352 (20 009 to 350 606)	685 (72 to 1 553)	-19.4 (-22.6 to 8.3)
Algeria	35 490 (24 550 to 47 320)	92 (64 to 123)	0.3 (-11.0 to 14.2)
Bahrain	1 375 (946 to 1 819)	94 (66 to 123)	-3.5 (-9.2 to 2.0)
Egypt	69 146 (47 749 to 90 578)	80 (56 to 104)	6.1 (0.2 to 11.3)
Iran	137 861 (63 416 to 244 285)	168 (79 to 302)	-29.1 (-35.2 to -10.9)
Iraq	171 082 (26 420 to 370 211)	588 (82 to 1 327)	-11.7 (-16.0 to 15.2)
Jordan	5 260 (3 649 to 6 913)	76 (53 to 99)	-15.4 (-21.6 to -9.5)
Kuwait	4 258 (2 869 to 5 685)	104 (71 to 141)	0.5 (-6.5 to 6.9)
Lebanon	36 679 (4 611 to 85 918)	614 (74 to 1 434)	-33.8 (-36.8 to -2.6)
Libya	14 064 (4 217 to 27 745)	210 (70 to 415)	89.5 (-16.9 to 179.8)
Morocco	28 025 (19 749 to 36 319)	82 (58 to 107)	-4.4 (-9.2 to 0.1)
Palestine	8 891 (2 638 to 18 553)	245 (68 to 528)	-18.3 (-23.6 to 1.8)
Oman	4 321 (3 013 to 5 665)	92 (64 to 121)	-11.0 (-15.1 to -7.3)
Qatar	2 647 (1 838 to 3 451)	112 (77 to 147)	-9.3 (-13.5 to -5.6)
Saudi Arabia	26 489 (18 322 to 34 704)	86 (59 to 111)	-23.9 (-28.0 to -20.2)
Sudan	41 069 (22 518 to 65 097)	119 (67 to 188)	17.6 (4.8 to 25.0)
Syria	64 852 (11 329 to 142 264)	345 (69 to 753)	241.4 (2.7 to 383.7)
Tunisia	9 690 (6 745 to 12 697)	83 (58 to 110)	-2.6 (-8.3 to 2.9)
Turkey	70 163 (48 417 to 92 448)	87 (61 to 115)	-20.2 (-28.2 to -11.8)
United Arab Emirates	10 942 (7 737 to 14 421)	103 (74 to 135)	-16.9 (-22.1 to -12.0)
Yemen	31 385 (16 242 to 52 528)	129 (70 to 205)	12.5 (-6.6 to 26.3)
**South Asia**	**1 571 585 (1 117 660 to 1 983 433)**	**97 (70 to 122)**	**8.9 (5.9 to 11.7)**
**South Asia**	**1 571 585 (1 117 660 to 1 983 433)**	**97 (70 to 122)**	**8.9 (5.9 to 11.7)**
Bangladesh	133 337 (93 315 to 170 161)	88 (63 to 114)	12.2 (6.0 to 18.8)
Bhutan	744 (525 to 953)	100 (71 to 128)	7.0 (1.0 to 12.7)
India	1 231 516 (871 821 to 1 555 124)	97 (69 to 124)	6.4 (3.3 to 9.1)
Nepal	26 857 (18 796 to 34 464)	100 (70 to 130)	26.0 (13.9 to 39.4)
Pakistan	169 910 (119 539 to 217 211)	99 (71 to 127)	22.7 (14.9 to 30.3)
**Sub-Saharan Africa**	**753 423 (482 382 to 1 037 402)**	**98 (65 to 137)**	**6.7 (-3.3 to 15.2)**

Location	YLDs (95% UI)		
	2016 counts	2016 age-standardised rates per 100,000	Percentage change in age-standardised rates between 1990 and 2016
**Southern sub-Saharan Africa**	**51 886 (36 503 to 66 929)**	**71 (49 to 91)**	**-17.4 (-19.6 to -15.2)**
Botswana	1 501 (1 048 to 1 959)	68 (47 to 89)	-2.3 (-13.8 to 9.0)
Lesotho	1 117 (804 to 1 414)	52 (37 to 66)	-12.1 (-17.6 to -6.5)
Namibia	1 470 (1 057 to 1 879)	64 (46 to 81)	-5.8 (-13.4 to 1.0)
South Africa	38 606 (26 635 to 50 110)	73 (53 to 94)	-22.0 (-24.8 to -19.3)
Swaziland	739 (520 to 945)	61 (42 to 78)	-12.6 (-20.1 to -5.2)
Zimbabwe	7 873 (5 567 to 9 960)	56 (40 to 71)	4.5 (-1.9 to 12.4)
**Western sub-Saharan Africa**	**259 337 (182 989 to 329 057)**	**81 (58 to 104)**	**3.6 (-2.9 to 11.3)**
Benin	6 869 (4 849 to 8 787)	75 (52 to 95)	-1.4 (-4.3 to 1.5)
Burkina Faso	10 754 (7 740 to 13 565)	72 (51 to 90)	2.7 (-0.5 to 6.0)
Cameroon	14 495 (10 282 to 18 469)	71 (52 to 91)	-6.7 (-11.1 to -2.3)
Cape Verde	382 (275 to 490)	77 (55 to 99)	4.0 (-0.3 to 8.1)
Chad	11 329 (7 292 to 16 038)	107 (66 to 159)	-2.8 (-6.7 to 2.7)
Cote d'Ivoire	15 042 (10 546 to 19 107)	77 (54 to 97)	-2.2 (-6.7 to 2.4)
The Gambia	1 220 (870 to 1 573)	78 (55 to 100)	-11.7 (-16.0 to -6.9)
Ghana	17 868 (12 603 to 22 745)	75 (53 to 95)	9.9 (5.5 to 14.3)
Guinea	7 556 (5 443 to 9 569)	71 (50 to 90)	-10.0 (-13.8 to -5.6)
Guinea-Bissau	1 235 (894 to 1 599)	75 (54 to 97)	-0.8 (-10.0 to 9.7)
Liberia	5 408 (2 256 to 10 244)	153 (59 to 276)	82.7 (-11.7 to 172.8)
Mali	11 924 (7 695 to 17 156)	87 (59 to 123)	21.5 (-6.4 to 59.1)
Mauritania	2 869 (2 055 to 3 678)	84 (58 to 106)	3.4 (-2.3 to 8.4)
Niger	10 581 (7 526 to 13 487)	69 (50 to 87)	-9.1 (-13.0 to -5.5)
Nigeria	117 876 (82 025 to 150 287)	79 (56 to 102)	2.1 (-4.5 to 8.5)
Sao Tome and Principe	139 (100 to 177)	88 (63 to 112)	-0.9 (-5.3 to 3.3)
Senegal	9 649 (6 855 to 12 226)	77 (55 to 98)	0.7 (-3.3 to 4.9)
Sierra Leone	6 073 (3 220 to 9 765)	115 (60 to 193)	55.2 (-11.0 to 131.6)
Togo	4 280 (3 052 to 5 423)	68 (48 to 86)	-7.3 (-10.7 to -4.0)
**Eastern sub-Saharan Africa**	**350 907 (209 415 to 521 517)**	**118 (66 to 184)**	**13.0 (1.5 to 20.5)**
Burundi	11 920 (6 120 to 19 568)	138 (67 to 228)	108.0 (15.1 to 232.2)
Comoros	558 (401 to 707)	85 (60 to 108)	0.6 (-3.9 to 4.6)

Location	YLDs (95% UI)		
	2016 counts	2016 age-standardised rates per 100,000	Percentage change in age-standardised rates between 1990 and 2016
Djibouti	758 (539 to 964)	92 (65 to 119)	-3.2 (-10.9 to 5.3)
Eritrea	5 824 (3 158 to 9 429)	134 (72 to 210)	79.5 (5.4 to 170.4)
Ethiopia	96 912 (58 096 to 148 158)	127 (70 to 200)	3.3 (-0.9 to 9.0)
Kenya	31 854 (22 612 to 40 436)	82 (58 to 104)	12.4 (6.9 to 17.6)
Madagascar	14 990 (10 758 to 18 945)	72 (52 to 92)	-2.7 (-7.2 to 1.0)
Malawi	8 549 (6 104 to 10 886)	58 (41 to 73)	-12.1 (-16.9 to -7.6)
Mozambique	26 500 (13 359 to 44 834)	139 (59 to 255)	-26.8 (-33.8 to -1.1)
Rwanda	39 929 (6 739 to 86 101)	443 (67 to 994)	501.0 (-0.2 to 1 103.4)
Somalia	12 931 (5 814 to 23 149)	155 (69 to 276)	33.2 (-8.4 to 57.2)
South Sudan	16 457 (7 610 to 27 825)	161 (73 to 277)	38.2 (-12.1 to 72.7)
Tanzania	33 584 (23 959 to 42 161)	76 (54 to 96)	-0.7 (-4.4 to 3.0)
Uganda	32 728 (18 585 to 50 571)	126 (60 to 219)	-15.2 (-27.1 to 21.4)
Zambia	10 019 (7 206 to 12 621)	73 (51 to 93)	-1.7 (-6.8 to 3.6)
**Central sub-Saharan Africa**	**93 615 (59 215 to 139 817)**	**104 (63 to 154)**	**18.2 (1.4 to 29.8)**
Angola	25 149 (14 498 to 39 618)	147 (76 to 247)	-4.3 (-13.2 to 14.0)
Central African Republic	3 634 (2 243 to 5 411)	77 (48 to 113)	37.1 (-1.6 to 87.0)
Congo	4 899 (2 759 to 7 854)	128 (67 to 211)	81.8 (9.1 to 186.6)
DR Congo	56 711 (37 248 to 79 279)	91 (59 to 128)	27.3 (-2.5 to 62.3)
Equatorial Guinea	603 (426 to 787)	83 (58 to 108)	22.6 (9.5 to 34.4)
Gabon	1 314 (933 to 1 661)	85 (61 to 108)	-4.2 (-9.0 to 0.1)
